# Analysis of hepatic transcript profile and plasma lipid profile in early lactating dairy cows fed grape seed and grape marc meal extract

**DOI:** 10.1186/s12864-017-3638-1

**Published:** 2017-03-23

**Authors:** Denise K. Gessner, Anne Winkler, Christian Koch, Georg Dusel, Gerhard Liebisch, Robert Ringseis, Klaus Eder

**Affiliations:** 1 Institute of Animal Nutrition and Nutrition Physiology, Justus-Liebig-University Giessen, Heinrich-Buff-Ring 26-32, Giessen, 35392 Germany; 2 Educational and Research Centre for Animal Husbandry, Hofgut Neumuehle, Muenchweiler an der Alsenz, 67728 Germany; 3 Department Life Sciences and Engineering, University of Applied Sciences, Bingen am Rhein, 55411 Germany; 4 Institute of Clinical Chemistry and Laboratory Medicine, University Hospital of Regensburg, Franz-Josef-Strauss-Allee 11, Regensburg, 93053 Germany

**Keywords:** Transcriptomics, Lipidomics, Dairy cow, Early lactation, Liver, Polyphenols, Grape seed and grape marc meal extract, Endoplasmic reticulum stress, Inflammation

## Abstract

**Background:**

It was recently reported that dairy cows fed a polyphenol-rich grape seed and grape marc meal extract (GSGME) during the transition period had an increased milk yield, but the underlying reasons remained unclear. As polyphenols exert a broad spectrum of metabolic effects, we hypothesized that feeding of GSGME influences metabolic pathways in the liver which could account for the positive effects of GSGME in dairy cows. In order to identify these pathways, we performed genome-wide transcript profiling in the liver and lipid profiling in plasma of dairy cows fed GSGME during the transition period at 1 week postpartum.

**Results:**

Transcriptomic analysis of the liver revealed 207 differentially expressed transcripts, from which 156 were up- and 51 were down-regulated, between cows fed GSGME and control cows. Gene set enrichment analysis of the 155 up-regulated mRNAs showed that the most enriched gene ontology (GO) biological process terms were dealing with cell cycle regulation and the most enriched Kyoto Encyclopedia of Genes and Genomes pathways were p53 signaling and cell cycle. Functional analysis of the 43 down-regulated mRNAs revealed that a great part of these genes are involved in endoplasmic reticulum (ER) stress-induced unfolded protein response (UPR) and inflammatory processes. Accordingly, protein folding, response to unfolded protein, unfolded protein binding, chemokine activity and heat shock protein binding were identified as one of the most enriched GO biological process and molecular function terms assigned to the down-regulated genes. In line with the transcriptomics data the plasma concentrations of the acute phase proteins serum amyloid A (SAA) and haptoglobin were reduced in cows fed GSGME compared to control cows. Lipidomic analysis of plasma revealed no differences in the concentrations of individual species of major and minor lipid classes between cows fed GSGME and control cows.

**Conclusions:**

Analysis of hepatic transcript profile in cows fed GSGME during the transition period at 1 week postpartum indicates that polyphenol-rich feed components are able to inhibit ER stress-induced UPR and inflammatory processes, both of which are considered to contribute to liver-associated diseases and to impair milk performance in dairy cows, in the liver of dairy cows during early lactation.

**Electronic supplementary material:**

The online version of this article (doi:10.1186/s12864-017-3638-1) contains supplementary material, which is available to authorized users.

## Background

The transition period spanning the time period between week 3 prepartum and week 3 postpartum represents the most critical period in the productive life of high-yielding dairy cows. With the onset of lactation, commonly a pronounced negative energy balance (NEB) is emerging due to the fact that feed intake is limited in this phase while energy demand is strongly increasing by milk production. NEB leads to a strong lipolysis of triacylglycerols (TAG) in adipose tissue, leading to the release of a large amount of non-esterified fatty acids (NEFA) into the circulation [1]. Approximately one-third of the whole body NEFA-flux is taken up into the liver. As the capacity of the liver for β-oxidation of fatty acids is limited during this phase, a part of the NEFA is esterified to TAG. Thus, a pronounced NEB during early lactation undoubtedly is involved in the development of liver-associated diseases such as fatty liver and ketosis [2]. Newer studies have shown that, besides metabolic stress induced by NEB, the liver of early lactating cows is also exposed to diverse inflammatory challenges. The inflammatory challenges, which include microbial components, pro-inflammatory cytokines and reactive oxygen species, typically result from infectious diseases, like endometritis and mastitis, but also from gastrointestinal disorders, like subacute rumen acidosis and abomasal displacement [3–5]. Both infectious diseases and gastrointestinal disorders frequently occur during parturition or the beginning of lactation. Due to of this, transition dairy cows develop an inflammation-like condition in the liver [4, 6]. Although this inflammation is mostly of subclinical nature, it is of great impact for health and performance of cows during early lactation [7].

Recently, it has been found that metabolic and inflammatory stress induces stress of the endoplasmic reticulum (ER) in the liver of early lactating cows [8]. ER stress is defined as an imbalance between the folding capacity of the ER and the protein load. As a consequence, unfolded and misfolded proteins accumulate in the ER lumen, thereby, disturbing ER homeostasis [9]. It is known from studies in humans and rodents that this causes activation of an adaptive response, termed unfolded protein response (UPR). While the aim of the UPR is to rapidly restore ER function [9], chronic activation of the UPR, as observed in obese or diabetic rodent models or induced by application of chemical ER stress inducers, causes various hepatic symptoms similar to those observed in periparturient dairy cows. Therefore, it has been proposed that ER stress-induced UPR contributes to the pathophysiologic conditions commonly observed in the liver of periparturient cows, like fatty liver, ketosis or inflammation [10].

Polyphenols are members of a large family of plant-derived compounds classified as flavonoids and non-flavonoids. Numerous studies in humans and rodents have shown that polyphenols are exerting antioxidative, antiinflammatory, cardioprotective, cancer chemopreventive and neuroprotective properties [11, 12]. In a recent study, we investigated the hypothesis that feeding grape seed and grape marc meal extract (GSGME), an inexpensive byproduct of wine and grape juice processing rich in flavonoids, to dairy cows might attenuate inflammation and ER stress in the liver during the transition period [13]. In that study, cows fed GSGME during the transition period had an increased milk yield and had a reduced mRNA concentration of fibroblast-growth factor (FGF)-21, a stress hormone, in the liver at week 1 and week 3 postpartum. Relative mRNA concentrations of various hepatic genes of inflammation and ER stress in the liver were decreased by 20-50% in the cows fed GSGME in comparison to the control group. However, as mRNA concentrations of these genes were not statistically significant different between the two groups of cows, the effect of polyphenols on hepatic inflammation and ER stress remains unclear. As polyphenols are exerting a broad spectrum of metabolic effects [14–16], we hypothesized that feeding of GSGME might influence other metabolic pathways in the liver which could account for the positive effects of GSGME observed in cows during early lactation. In order to investigate this hypothesis, we used a genome-wide transcript profiling technique to explore changes in the hepatic transcriptome of cows supplemented with GSGME during the transition period. A main advantage of large-scale screening technologies like transcriptomics is that changes in the complete transcriptome can be assessed simultaneously, despite only small amounts of tissue, e.g. biopsy samples, being available. Using this technique in dairy cows has strongly increased understanding of the hepatic molecular adaptations occurring in the periparturient period [17–19]. Transcriptomics in combination with the analysis of selected blood metabolites and animal performance parameters facilitates to relate changes in the hepatic transcriptome to alterations of liver function during the periparturient period [17–19].

Recently, a gene-based mapping and pathway analysis of metabolic traits in dairy cows figured out that hepatic genes of glycerophospholipid metabolism (e.g., lysophosphatidylcholine acyltransferase 1) are closely linked to plasma concentrations of NEFA, β-hydroxybutyrate (BHBA) and glucose, three key factors of the metabolic status of dairy cows during early lactation [20]. Moreover, signaling lipids, such as ceramides, are implicated in pathways regulating inflammation [21, 22]. As polyphenols have been shown to exert pronounced effects on hepatic lipid metabolism, particularly under pathological conditions [23], we further aimed to find out whether feeding of GSGME could influence metabolism of glycerophospholipids and ceramides. Therefore, we performed a lipidomic analysis of plasma samples.

## Methods

### Animal experiment

For this investigation, we used liver and plasma samples collected at 1 week postpartum of an experiment with dairy cows [13]. At this time point, both metabolic and inflammatory stress markers, such as plasma NEFA, plasma BHBA and hepatic mRNA concentrations of acute phase proteins (APPs), were increased most compared to later sampling time points (week 3 and week 5) in this study and another study [13, 24]. In this experiment, 28 Holstein cows with an average parity number of 2.8 were used as experimental animals. The experiment was conducted at the Educational and Research Centre for Animal Husbandry Hofgut Neumühle in Rhineland-Palatinate (Münchweiler an der Alsenz, Germany); the experimental protocol was approved by the Provincial Government of Coblenz, Germany (23 177–07/G12–20–074). The cows were assigned into 2 experimental groups, either a control group (*n =* 14) or a group supplemented with GSGME (GSGME group; *n =* 14), each consisting of 10 multiparous and 4 primiparous cows and having a similar average parity number (control group: 2.8, GSGME group: 2.9). In the period between week 3 prepartum and calving, a total mixed ration (TMR) was fed which was calculated to meet the demand of net energy (NE) and crude protein (CP) requirement of a dry cow with a BW of 650 kg and an assumed dry matter intake (DMI) of 12 kg/d, according to the German Society of Nutrition Physiology [25]. After calving, all animals were offered a basal TMR calculated to meet the demand of net energy and CP requirement for producing 34 kg of milk, with an assumed daily DMI of 22 kg [13]. Feed components were collected fortnightly and analyzed according to the official methods of Verband der Deutschen Landwirtschaftlichen Untersuchungs- und Forschungsanstalten [26]. The analyzed chemical composition of the TMR offered during dry period and lactation was in average of control and GSGME group as follows (per kg DM): 6.5 and 6.8 MJ NE_L_, 140 and 166 g CP, 383 and 356 g neutral detergent fiber. More details on the analytical composition of the TMR have been published recently [13]. In the time period from 3 week before the expected calving date until week 9 postpartum, the basal TMR of the GSGME group was supplemented with 1% of GSGME (Antaox, Dr. Eckel, Niederzissen, Germany) based on DM content. The GSGME product used had a total flavonoid content of 52 mg gallic acid equivalents per gram. The TMR of the control group was supplemented with 1% of wheat bran for an energetic adjustment. Although the NE_L_ content of the GSGME used in this study was slightly lower (3.64 MJ NE_L_/kg DM [27]) than that of wheat bran (4.18 MJ NE_L_/kg DM) [28], the NE_L_ content of the TMR between the two groups was nearly identical due to the small proportion of GSGME and wheat bran, respectively, in the TMR [13].

### Blood samples and liver biopsies

Each cow was separated from the herd for blood sampling and liver biopsy procedure. Blood was taken from the vena caudalis at week 1 (day 7 postpartum ± 2 d) using ethylenediaminetetraacetic acid-coated collection tubes (S-Monovette, Sarstedt, Nümbrecht, Germany). Plasma was separated from blood cells by centrifugation, and the plasma samples were stored at −20 °C until analysis. Liver biopsies were taken after sampling of blood according to the protocol recently described [13] and immediately snap-frozen in liquid nitrogen and stored at −80 °C until further analysis.

### RNA isolation

Total RNA was isolated from liver samples using Trizol according to the manufacturer’s protocol and stored at −80 °C. Prior to sample processing at the Centre of Excellence for Fluorescent Bioanalytics (KFB) at the University of Regensburg, the concentration and integrity of RNA was analyzed using an Agilent 2100 Bioanalyzer (Agilent technologies, Böblingen, Germany). The total RNA concentrations, optical density A260/A280 ratios, RNA integrity number (RIN) values and starting total RNA amounts of all samples were 0.59 ± 0.08 μg/μL, 1.93 ± 0.03, 6.6 ± 0.5 and 3.8 ± 0.5 μg (mean ± SD, *n =* 12), respectively.

### Microarray hybridization

For microarray analysis, six RNA samples each of the control group (*n =* 6) and the GSGME group (*n =* 6) were selected. The six RNA samples of each group consisted of five samples randomly selected from the multiparous cows and one sample randomly selected from the primiparous cows. Both groups had a similar average parity number (control group: 2.5, GSGME group: 2.3). Total RNA samples were processed according to the GeneChip WT Plus Reagent Kit (Affymetrix, High Wycombe, UK). In brief, total RNA was transcribed to first strand and second strand complementary DNA (cDNA). Then, complementary RNA (cRNA) was synthesized and amplified by *in vitro*-transcription of the second-stranded cDNA template using T7 RNA polymerase. After purification of cRNA and assessing cRNA yield and quality, single-stranded (ss) cDNA was synthesized by reverse transcription of cRNA using 2^nd^-cycle primers. The ss cDNA was purified and checked again for yield and quality. The purified ss cDNA was fragmented and the fragmented cDNA labeled by terminal deoxynucleotidyl transferase using the Affymetrix proprietary DNA labeling reagent that is covalently linked to biotin. Finally, the labeled ss cDNA was hybridized to the Affymetrix GeneChip Bovine Gene 1.0 Sense Target array representing approximately 23,000 bovine transcripts. After hybridization arrays were washed and stained with the Affymetrix GeneChip Fluidics station 450. Finally, arrays were scanned with an Affymetrix GeneChip scanner 3000. The quality of hybridization was assessed in all samples following the manufacturer’s recommendations. The microarray data have been deposited in MIAME compliant format in the NCBI’s Gene Expression Omnibus public repository ([29]; GEO accession no. GSE86368).

### Microarray analysis

After scanning the microarrays, cell intensity files containing a single intensity value for each probe cell were computed from the image data with the Affymetrix GeneChip Command Console Software. Background correction and normalization of probe cell intensity data was performed with Affymetrix Expression Console software using the Robust Multichip Analysis (RMA) algorithm. This algorithm is a log scale multi-chip analysis approach fitting a robust linear model at the probe level to minimize the effect of probe-specific affinity differences. Expression levels of transcripts are measured using log transformed perfect match values, after carrying out a global background adjustment and across microarray normalization [30]. The microarrays were annotated using the Affymetrix BovGene-1_0-st-v1_Probeset_Release 36 annotation file. Transcripts were defined as differentially expressed when the fold-change (FC) between GSGME group and control group was > 1.3 or < −1.3 and the *P-*value of the unpaired Student’s t-test (two-tailed distribution, two-sample equal variance) for each transcript was < 0.05. False discovery rates (FDR) according to Benjamini-Hochberg multiple testing correction were also calculated. However, the FDR value was not applied as a cut off criterion, since the FDR-corrected *P-*values for all 23,000 transcripts were > 0.05.

### Bioinformatic prediction of mRNA targets of differentially expressed miRNAs

Bioinformatic prediction of mRNA targets for differentially regulated miRNAs was performed using TargetScan release version 7.1 (http://www.targetscan.org/vert_71/) for the species “cow”. TargetScan predicts biological targets of miRNAs by searching for the presence of conserved 6 to 8mer sites matching the seed region of each miRNA [31]. In mammals, predictions are ranked based on the predicted efficacy of targeting as calculated using cumulative weighted context++ scores of the sites [32]. A cumulative weighted context++ score < −0.20 was used as cut off criterion for predicting mRNAs targets.

### Gene set enrichment analysis

To extract biological meaning from the identified differentially expressed transcripts and predicted mRNA targets, gene set enrichment analysis (GSEA) with a modified Fisher’s exact test was performed in order to identify enriched Gene Ontology (GO) terms within GO categories (biological process, molecular function, cellular component) and enriched Kyoto Encyclopedia of Genes and Genomes (KEGG) pathways using the Database for Annotation, Visualization and Integrated Discovery (DAVID) 6.7 bioinformatic resource [33, 34]. GO terms and KEGG pathways were defined as enriched if the FDR-adjusted *P-*value according to the Benjamini-Hochberg correction was < 0.05. GSEA was performed separately for the up- and down-regulated mRNAs and predicted mRNAs, respectively. The rationale of performing GSEA separately for the up- and down-regulated transcripts and not for all differentially expressed transcripts together is that results from GSEA are better to interpret, i.e. based on this approach it is assumed that biological processes or molecular functions and pathways identified as enriched within up-regulated genes are probably activated, whereas those enriched with down-regulated genes are likely inhibited.

### Quantitative real-time polymerase chain reaction (qPCR) analysis

Microarray data of 25 differentially expressed mRNAs were validated by qPCR. For qPCR analysis, total RNA from all cows (*n =* 14 per group) was used to generate cDNA by reverse transcription. The cDNA was synthesized using a Mastermix containing 1.2 μg of total RNA, 100 pmol oligo(dT)18 primer (Eurofins MWG Operon, Ebersberg, Germany), 1.25 μL dNTP mix (10 mM, GeneCraft, Lüdinghausen, Germany), 5 μL 5× RT reaction buffer (Thermo Fisher Scientific, St. Leon-Rot, Deutschland) and 60 units M-MuLV Reverse Transcriptase (Thermo Fisher Scientific). The cDNA synthesis was carried out at 42 °C for 60 min and a final inactivating step at 70 °C for 10 min in a thermocycler (Biometra, Göttingen, Germany). The relative mRNA expression of genes was measured with a Rotor-Gene Q system (Qiagen, Hilden, Germany) using KAPA SYBR FAST qPCR Mastermix (Peqlab, Erlangen, Germany) and gene-specific primer pairs (Eurofins MWG Operon, Ebersberg, Germany) that were designed using Primer3 and BLAST. Primer characteristics of reference genes were recently published [13]. Primer characteristics of target genes are shown in Additional file 1: Table S1. Ct-values of reference and target genes were obtained using Rotor-Gene Q Software (Qiagen). For normalization of relative expression levels GeNorm normalization factor was calculated from the three most stable (beta-actin, peptidylprolyl isomerase A, ribosomal protein S9) out of six reference genes tested [35]. Raw Ct-values of reference genes were statistically analyzed to ensure that expression levels did not differ between groups. Raw Ct-values were transformed into relative expression values using the 2^-ΔCt^ equation for the calculation of the normalization factors. The highest relative value of each gene was set to 1. From these values, the normalization factor was calculated as the geometric mean of expression data of the three most stable reference genes. Ct-values of target genes were also transformed into relative expression values using the 2^-ΔCt^ equation and were normalized with the individual normalization factor resulting in relative gene quantities that were used for the statistical analysis. The mean normalized 2^-ΔCt^ ratios of the control group was set to 1.0 and the mean and SD of normalized 2^-ΔCt^ ratios of the GSGME group was scaled proportionally. PCR products were separated electrophoretically using a 1.5% agarose gel stained with GelRed nucleic acid gel stain (Biotium, Hayward, CA, USA) to confirm the expected size of the PCR products.

### Plasma concentration of acute phase proteins

Plasma concentrations of bovine haptoglobin (HP) and serum amyloid A (SAA) were analyzed using commercial ELISA Kits (CSB-E08585b, CSB-E08592b, Hölzel Diagnostika, Cologne, Germany). The ELISA procedure was performed based on the instructions provided by the manufacturer and absorbance read in a microplate reader (Infinite® 200, Tecan, Mainz, Germany). According to manufacturer’s information, the limits of detection were 7.8 μg HP/L plasma for the HP ELISA kit and 50 μg SAA/L plasma for the SAA kit. All samples were measured in duplicate. Intra-assay coefficients of variability (CV) were < 10% for each sample in both assays. The average of individual CV was 5.8% and 3.7% for the measurement of HP and SAA, respectively.

### Lipidomic analysis

Lipid extraction was carried out in the presence of non-naturally occurring lipid species as internal standards according to the protocol of Bligh and Dyer [36]. Determination of plasma lipid species was accomplished by means of direct flow injection electrospray ionization tandem mass spectrometry (ESI-MS/MS) in positive ion mode as described in [37, 38]. For phosphatidylcholine (PC), lysophosphatidylcholine (LPC), and sphingomyelin (SM) a precursor ion of *m/z* 184 was used [38, 39]. A fragment ion of *m/z* 264 was used to analyze spingosine based ceramides (Cer) and hexosylceramides (HexCer), while a fragment ion of *m/z* 369 was used for the analysis of free cholesterol (FC) and cholesteryl esters (CE) after selective derivatization of FC [38, 40]. Phosphatidylethanolamine species (PE) and phosphatidylinositol (PI) were analysed following neutral loss fragment of 141 and 277 Da, respectively [41, 42]. The analysis of PE-based plasmalogens (PE-P) with 16:0, 18:0 and 18:1 vinylether bonds was performed as described by Zemski-Berry [43]. Data analysis was performed with Mass Lynx software including the NeoLynx tool (Micromass) and results were exported to Excel and further processed by self-programmed Excel Macros [37]. Annotation of lipid species was carried out according to the LipidomicNet proposal for shorthand notation of lipid structures derived from mass spectrometry [44]. Glycerophospholipid species annotation was based on the assumption of even-numbered carbon chains only. Sphingomyelin species were assigned based on the assumption of a sphingoid base with 2 hydroxyl groups.

### Statistical analysis

Values presented in the text are means ± SD. All data were evaluated by Student’s t test using the Minitab statistical software (Release 13, Minitab Inc., State College, PA, USA). Multiple testing correction of microarray data was performed by Benjamini and Hochberg FDR.

## Results

### Identification of differentially expressed transcripts

To investigate the effect of GSGME on the transcriptome in the liver of dairy cows, we used a bovine microarray representing approximately 23,000 *Bos taurus* transcripts. Taking into account the criteria FC > 1.3 or FC < −1.3 and *P <* 0.05 a total of 207 transcripts were found to be differentially expressed in the liver between cows fed GSGME and control cows. Substantially more transcripts were up-regulated by GSGME (156), while only 51 transcripts were down-regulated by GSGME in the liver of cows. The up-regulated transcripts included 155 protein-coding transcripts (mRNAs) and 1 non-protein-coding miRNA, whereas the down-regulated transcripts included 43 mRNAs and 8 miRNAs. The 20 most strongly up- and down-regulated mRNAs are presented in Table 1 and Table 2, respectively. The FCs of the most strongly up-regulated mRNAs ranged between 2.91 and 1.90, while those of the most strongly down-regulated mRNAs ranged between −1.66 and −1.39. In Table 3 the differentially regulated miRNAs including FCs and *P-*values are shown.

**Table 1:** The 20 most strongly up-regulated mRNAs in the liver of cows fed grape seed and grape marc meal extract (GSGME) versus control cows at 1 week postpartum

Gene symbol	mRNA description	FC^a^	*P-*value
TOP2A	topoisomerase (DNA) II alpha 170 kDa	2.91	0.013
CDKN3	cyclin-dependent kinase inhibitor 3	2.64	0.010
ARHGAP11A	Rho GTPase activating protein 11A	2.61	0.029
STMN1	stathmin 1	2.57	0.015
ECT2	epithelial cell transforming sequence 2 oncogene	2.53	0.023
DEPDC1	DEP domain containing 1	2.51	0.015
CENPA	centromere protein A	2.49	0.006
CENPF	centromere protein F, 350/400 kDa (mitosin)	2.45	0.017
CKAP2	cytoskeleton associated protein 2	2.39	0.024
PRR11	proline rich 11	2.32	0.036
KIF11	kinesin family member 11	2.25	0.007
KIF20A	kinesin family member 20A	2.21	0.017
BUB1B	budding uninhibited by benzimidazoles 1 homolog beta	2.20	0.004
LOC618147	histone cluster 1, H2ai-like	2.17	0.002
KIF4A	kinesin family member 4A	2.13	0.018
LOC787465	histone H2B type 1-like	2.10	0.006
GAS2L3	growth arrest-specific 2 like 3	1.99	0.018
SMC4	structural maintenance of chromosomes 4	1.98	0.011
SMC2	structural maintenance of chromosomes 2	1.96	0.011
RRM2	ribonucleotide reductase M2	1.95	0.046
CASC5	cancer susceptibility candidate 5	1.93	0.029
ESCO2	establishment of cohesion 1 homolog 2	1.93	0.032
HIST2H2BF	histone cluster 2, H2bf	1.91	0.039
HELLS	helicase, lymphoid-specific	1.90	0.013

^a^The FC was calculated from the signal log ratios as follows: 2^Signal log ratio^ if signal log ratio ≥ 0 and (−1) × 2^–(Signal log ratio)^ if signal log ratio < 0. Signal log ratios were calculated from *n =* 6 microarrays per group

**Table 2:** The 20 most strongly down-regulated mRNAs in the liver of cows fed grape seed and grape marc meal extract (GSGME) versus control cows at 1 week postpartum

Gene symbol	mRNA description	FC^a^	*P-*value
GLCE	glucuronic acid epimerase	–1.66	0.036
TBATA	chromosome 28 open reading frame, human C10orf27	–1.60	0.011
MANF	mesencephalic astrocyte-derived neurotrophic factor	–1.59	0.014
XBP1	X-box binding protein 1, transcript variant 1	–1.59	0.003
LOC618817	olfactory receptor, family 6, subfamily B, member 2-like	–1.55	0.005
SAA4	serum amyloid A4, constitutive	–1.54	0.043
HSPA5	heat shock 70 kDa protein 5 (glucose-regulated protein, 78 kDa)	–1.51	0.005
GADD45B	growth arrest and DNA-damage-inducible, beta	–1.51	0.012
WWC1	WW and C2 domain containing 1, transcript variant 2	–1.48	0.037
LOC788587	olfactory receptor, family 4, subfamily D, member 11-like	–1.46	0.001
SOCS3	suppressor of cytokine signaling 3	–1.45	0.042
C15H11orf96	chromosome 15 open reading frame, human C11orf96	–1.44	0.018
PHLDA1	pleckstrin homology-like domain, family A, member 1	–1.43	0.012
SDF2L1	stromal cell-derived factor 2-like 1	–1.41	0.012
IRX3	iroquois homeobox 3	–1.40	0.001
LOC784679	peptidylprolyl isomerase A (cyclophilin A)-like	–1.39	0.023
HYOU1	hypoxia up-regulated 1, transcript variant 1	–1.39	0.018
ALX3	ALX homeobox 3	–1.39	0.010
CFHR2	complement factor H-related 2	–1.39	0.028
LOC520181	olfactory receptor 5-like	–1.39	0.007

^a^The FC was calculated from the signal log ratios as follows: 2^Signal log ratio^ if signal log ratio ≥ 0 and (−1) × 2^–(Signal log ratio)^ if signal log ratio < 0. Signal log ratios were calculated from *n =* 6 microarrays per group

**Table 3:** The most strongly differentially regulated (FC > 1.3 or FC < −1.3 and *P <* 0.05) miRNAs in the liver of cows fed grape seed and grape marc meal extract (GSGME) versus control cows at 1 week postpartum

Gene symbol	micro RNA description	FC^a^	*P-*value
MIR376C	microRNA mir-376c	1.40	0.027
MIR365-2	microRNA mir-365–2	–1.31	0.010
MIR2345	microRNA mir-2345	–1.33	0.007
MIR2403	microRNA mir-2403	–1.34	0.029
MIR2462	microRNA mir-2462	–1.39	0.006
MIR2359	microRNA mir-2359	–1.41	0.002
MIR2430	microRNA mir-2430	–1.51	0.004
MIR2461	microRNA mir-2461	–1.53	0.010
MIR365	microRNA mir-365	–1.56	0.003

^a^The FC was calculated from the signal log ratios as follows: 2^Signal log ratio^ if signal log ratio ≥ 0 and (−1) × 2^–(Signal log ratio)^ if signal log ratio < 0. Signal log ratios were calculated from *n =* 6 microarrays per group

### Validation of microarray data for selected differentially expressed protein-coding transcripts by qPCR

Validation of microarray data was carried out by qPCR analysis for 25 differentially regulated mRNAs. The transcripts to be validated by qPCR were randomly selected from the most strongly up- and down-regulated mRNAs. Since the number of transcripts up-regulated was higher than that down-regulated, we validated 14 up- and 11-down-regulated transcripts by qPCR. Table 4 shows that in the case of most mRNAs (19) the effect direction was the same between qPCR and microarray data, but the FCs from qPCR analysis were markedly lower than from microarray analysis. In the case of about half (9) of the differentially regulated mRNAs, qPCR analysis revealed a FC greater than the filter criterion for differential regulation in microarray analysis (>1.3 or < −1.3). In line with this, statistical analysis revealed that only 5 of these 9 mRNAs were differentially regulated according to qPCR analysis at a significance level of *P <* 0.05 (TUBB, PHLDA1) or at least *P <* 0.1 (KIF20A, SAA4, HYOU1). In the case of 15 mRNAs, the FC determined by qPCR analysis was below the filter criterion for differential regulation and the *P-*value was not significant (*P >* 0.05). In the case of one mRNA (HMMR) the effect direction determined by qPCR analysis (FC = −1.34) was contrary to that determined by microarray analysis (FC = 1.87. Regarding these partial inconsistencies between microarray and qPCR data (statistical results, effect size), three causative factors should be noted: 1) The number of biological replicates was different between microarray and qPCR analysis (*n =* 6 vs. *n =* 14) influencing the statistical power of the data. 2) For microarray analysis six cows (1 primiparous and 5 multiparous) were selected from the control group and the GSGME group each consisting of 4 primiparous and 10 multiparous cows. Due to this, the average parity number of the groups used for microarray analysis was slightly lower than of the groups used for qPCR. 3) The detection principle of transcript abundance differs between microarray and qPCR, i.e. for qPCR analysis a 100–250 bp sequence of the transcript is amplified by a single primer pair, whereas for microarray analysis up to 26 unique 25-mer probes are used for each transcript resulting in a high coverage across the entire transcript of up to 650 bp.

**Table 4:** Validation of microarray data for selected differentially expressed transcripts by qPCR

	Mean FC		*P-*value	
Gene symbol	Microarray	qPCR	Microarray	qPCR
STMN1	2.57	1.37	0.015	0.269
ECT2	2.53	1.45	0.023	0.246
CENPA	2.49	1.16	0.006	0.514
CENPF	2.45	1.07	0.017	0.788
CKAP2	2.39	1.20	0.023	0.557
PRR11	2.32	1.93	0.036	0.181
KIF20A	2.21	1.66	0.017	0.088
BUB1B	2.20	1.22	0.004	0.528
RRM2	1.95	1.56	0.046	0.263
ESCO2	1.93	–1.02	0.032	0.905
SPC25	1.88	–1.19	0.034	0.207
CCNA2	1.87	–1.07	0.030	0.762
HMMR	1.87	–1.34	0.018	0.100
TUBB	1.85	1.38	0.013	0.040
GLCE	–1.66	–1.2	0.036	0.195
MANF	–1.59	–1.23	0.014	0.235
SAA4	–1.54	–1.31	0.043	0.057
SOCS3	–1.45	–1.14	0.042	0.522
PHLDA1	–1.43	–1.34	0.012	0.006
HYOU1	–1.39	–1.33	0.018	0.081
DNAJB11	–1.37	–1.23	0.026	0.170
BAG3	–1.37	1.07	0.032	0.674
UAP1	–1.34	1.05	0.005	0.674
CCNL1	–1.33	–1.26	0.009	0.067
CXCL14	–1.31	–1.16	0.036	0.459

The microarray FC was calculated from the signal log ratios as follows: 2^Signal log ratio^ if signal log ratio ≥ 0 and (−1) × 2^–(Signal log ratio)^ if signal log ratio < 0. Signal log ratios were calculated from *n =* 6 microarrays per group. The qPCR FC was calculated analogously from normalized 2^-ΔCt^ ratios. Normalized 2^-ΔCt^ expression was calculated from *n =* 14 samples per group

### Identification of enriched annotation terms associated with the differentially expressed protein-coding transcripts

**Fig. 1:**
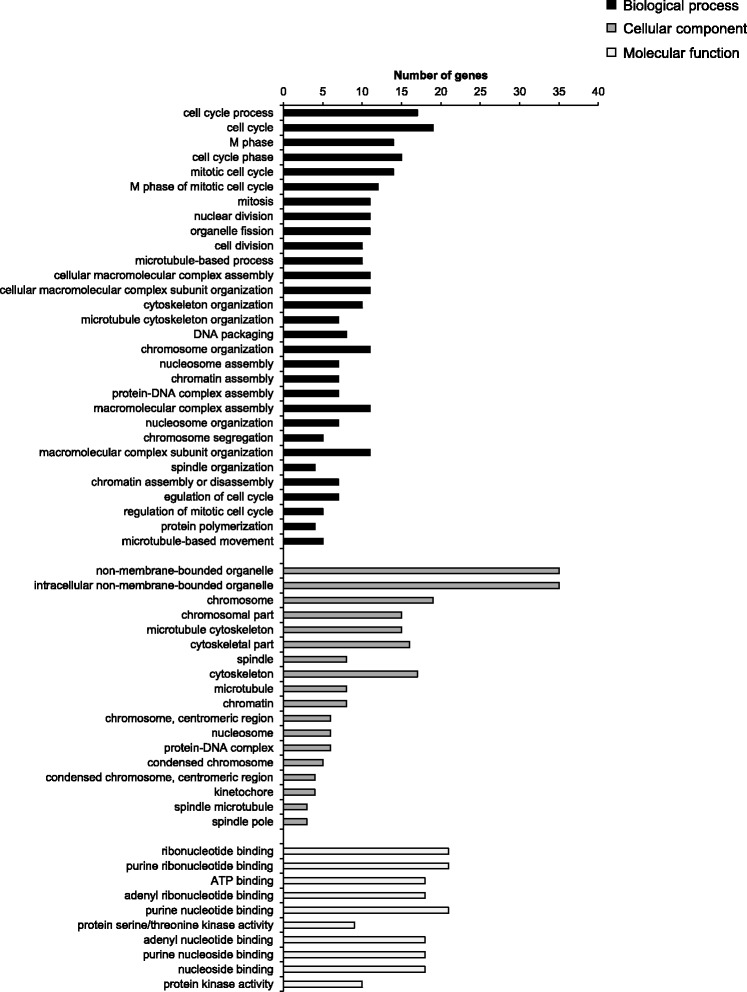
The most enriched gene ontology (GO) terms assigned to the up-regulated mRNAs including the number of genes. The GO terms were sorted by their enrichment *P-*values (EASE score) (top: lowest *P-*value, bottom: highest *P-*value) within the GO categories biological process, cellular component and molecular function. Only GO terms with FDR-adjusted *P-*values < 0.05 are shown

GSEA of the 155 up-regulated mRNAs showed that the GO terms with lowest FDR-adjusted *P-*values (most enriched) from all GO categories (biological process, cellular component, molecular function) were non-membrane-bounded organelle, intracellular non-membrane-bounded organelle, chromosome, cell cycle process, cell cycle, M phase, cell cycle phase, mitotic cell cycle, M phase of mitotic cell cycle, chromosomal part, microtubule cytoskeleton, mitosis, nuclear division, organelle fission, cytoskeletal part, cell division, spindle, microtubule-based process and cytoskeleton. Figure 1 shows the GO terms with FDR-adjusted *P-*values < 0.05 including the number of genes assigned to these terms separately for the GO categories biological process, cellular component and molecular function.

For the 43 down-regulated mRNAs GSEA revealed only three enriched GO terms with FDR-adjusted *P-*values < 0.05, namely ER lumen, ER part and ER. These GO terms belonged exclusively to the GO category cellular component.

### Identification of enriched regulatory pathways associated with the differentially expressed protein-coding transcripts

To identify regulatory pathways associated with the differentially expressed transcripts GSEA was performed using the KEGG database. The most enriched pathways with FDR-adjusted *P-*values < 0.05 identified from the 155 up-regulated mRNAs included pathways regulating systemic lupus erythematosus and cell cycle, while no enriched pathways FDR-adjusted *P-*values < 0.05 were identified from the 43 down-regulated mRNAs.

### Prediction of mRNA targets of the differentially expressed miRNAs and functional analysis

As described above, several miRNAs were identified as differentially expressed by microarray analysis of the cow livers. In order to identify further protein-coding transcripts that are influenced by feeding GSGME in the liver of cows, we performed bioinformatic target prediction for the 9 differentially regulated miRNAs. Considering a cumulative weighted context++ score < −0.20, a total of 185 target genes were identified for the up-regulated mir-376c, and 2,412 target genes for the highly conserved down-regulated miRNAs (mir-2345, mir-2403, mir-2462, mir-2359, mir-2430, mir-365). Data including gene names, total and 8mer, 7mer and 6mer sites and cumulative weighted context++ score are shown in Additional file 2: Table S2.

To elucidate the biological functions of the predicted target genes we carried out GSEA using GO category “biological process” and KEGG pathways separately for the targets identified for the up- and the down-regulated miRNAs. However, GSEA of the target genes of the up-regulated miRNA revealed no enriched biological process terms and KEGG pathways with FDR-adjusted *P-*values < 0.05.

GSEA of the target genes of the down-regulated miRNAs identified the following enriched GO biological process terms (FDR-adjusted *P-*value < 0.05): intracellular signaling cascade, positive regulation of macromolecule metabolic process, positive regulation of macromolecule biosynthetic process, positive regulation of biosynthetic process, positive regulation of cellular biosynthetic process and positive regulation of transcription. No enriched KEGG pathways with FDR-adjusted *P-*values < 0.05 could be identified by GSEA of target genes of the down-regulated miRNAs.

### Plasma concentrations of acute phase proteins

**Fig. 2:**
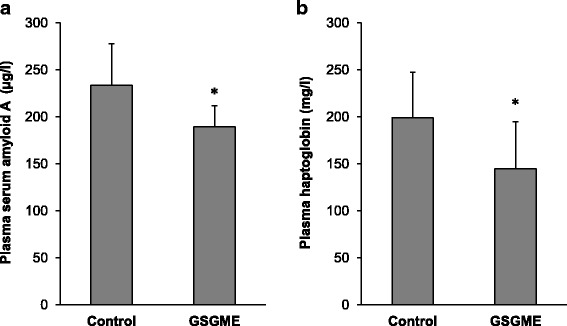
Plasma concentration of **a** serum amyloid A and **b** haptoglobin in cows fed grape seed and grape marc meal extract (GSGME) and control cows at 1 week postpartum. Bars are means ± SD for *n =* 14 cows per group. Asterisk denotes difference between cows fed GSGME and control cows (*P <* 0.05)

Plasma concentration of the positive APPs SAA and HP were decreased in cows fed the GSGME compared to the control group (*P <* 0.05; Fig. 2).

### Plasma lipid profile

Using lipidomic analysis, we were able to detect individual species of major (cholesterol, PC, SM, LPC) and minor (PE, PE plasmalogens, PI, ceramides) lipid classes in plasma samples of the cows. For all these lipid classes, there were no differences between the two groups of cows in the concentrations of any of the individual molecular species (*P >* 0.05, data are shown in Additional file 3: Tables S3-S9). Moreover, for all the lipids analyzed, the concentrations of species with no double bond (SFA), one double bond (MUFA) or two or more double bounds (PUFA) in the fatty acid moieties did not differ between the two groups of cows (*P >* 0.05, Table 5). In the PE plasmalogen fraction, there were moreover no differences in the concentrations of species with 16:0, 18:0 and 18:1 vinyl ether bonds between the two groups of cows (*P >* 0.05, Table 5).

**Table 5:** Plasma lipid profile of cows fed grape seed and grape marc meal extract (GSGME) versus control cows at 1 week postpartum

Lipid class	Group	Total	SFA	MUFA	PUFA
*Major lipid classes*					
CE, μM	Control	3070 ± 663	135 ± 38.8	206 ± 57.0	2730 ± 574.7
	GSGME	3079 ± 794	131 ± 32.4	204 ± 57.7	2743 ± 714.9
FC, μM	Control	411 ± 111	-	-	-
	GSGME	444 ± 124	-	-	-
LPC, μM	Control	77.0 ± 20.2	55.6 ± 13.9	9.35 ± 3.25	12.1 ± 4.05
	GSGME	80.7 ± 21.2	57.4 ± 15.0	10.0 ± 2.78	13.3 ± 4.18
PC, μM	Control	996 ± 285	22.8 ± 5.80	279 ± 91.8	598 ± 164
	GSGME	999 ± 293	22.6 ± 5.96	285 ± 87.0	594 ± 181
SM, μM	Control	169 ± 46.1	116 ± 27.5	44.4 ± 12.5	8.8 ± 6.0
	GSGME	170 ± 52.2	116 ± 33.1	44.2 ± 13.4	9.9 ± 6.0
*Minor lipid classes*					
Cer-d18:1, μM	Control	1.28 ± 0.24	1.11 ± 0.23	0.18 ± 0.04	-
	GSGME	1.28 ± 0.27	1.09 ± 0.23	0.19 ± 0.05	-
HexCer-d18:1, μM	Control	0.22 ± 0.05	0.11 ± 0.03	0.11 ± 0.02	-
	GSGME	0.23 ± 0.06	0.12 ± 0.04	0.12 ± 0.03	-
PI, μM	Control	9.65 ± 2.34	0.07 ± 0.01	2.02 ± 0.74	7.57 ± 1.68
	GSGME	9.58 ± 2.89	0.06 ± 0.02	2.08 ± 0.76	7.44 ± 2.18
PE, μM	Control	6.36 ± 1.96	0.19 ± 0.07	0.99 ± 0.30	4.97 ± 1.66
	GSGME	6.36 ± 1.64	0.17 ± 0.07	1.02 ± 0.25	4.95 ± 1.40
PE-P-16:0, μM	Control	6.73 ± 1.27	0.61 ± 0.05	1.01 ± 0.25	5.11 ± 1.06
	GSGME	6.99 ± 1.69	0.59 ± 0.10	1.09 ± 0.29	5.31 ± 1.37
PE-P-18:0, μM	Control	4.09 ± 0.39	0.57 ± 0.06	0.70 ± 0.10	2.82 ± 0.27
	GSGME	3.87 ± 0.52	0.51 ± 0.06	0.67 ± 0.11	2.69 ± 0.38
PE-P-18:1, μM	Control	4.48 ± 0.40	0.59 ± 0.04	0.77 ± 0.14	3.12 ± 0.29
	GSGME	4.41 ± 0.64	0.56 ± 0.08	0.79 ± 0.13	3.06 ± 0.50

Values are means ± SD for *n =* 14 cows per group

## Discussion

Recently, we observed that feeding GSGME to dairy cows from 3 week antepartum to 9 week postpartum increases milk yield and causes some beneficial changes in mRNA concentrations of hepatic genes, such as reduced mRNA concentration of FGF21, an indicator of metabolic and ER stress [13]. As the reasons underlying these effects could not be elucidated in the recent study, the aim of the present study was to identify changes in potentially critical signaling or metabolic pathways by using transcriptomic and lipidomic analyses. For this end, we considered liver and plasma samples obtained at 1 week postpartum regarding that metabolic and infectious stress in dairy cows is greatest at this early time after birth [6, 8, 17].

One striking finding of transcriptome analysis in the liver of cows was that within the limited number of protein-coding genes down-regulated by GSGME there was a large number of genes involved in ER stress-induced UPR, such as X-box binding protein 1 (XBP1), heat shock 70 kDa protein 5 (HSPA5)/GRP78, homocysteine inducible ER protein with ubiquitin like domain 1 (HERPUD1), DnaJ (Hsp40) homolog, subfamily C, member 5G (DNAJC5G), calreticulin (CALR), protein disulfide isomerase family A, member 4 (PDIA4), DnaJ (Hsp40) homolog, subfamily B, member 11 (DNAJB11), pleckstrin homology-like domain, family A, member 1 (PHLDA1)/TDAG51, protein phosphatase 1 regulatory subunit 3C (PPP1R3C), growth arrest and DNA damage inducible beta (GADD45B), BCL2-associated anthanogene 3 (BAG3), hypoxia up-regulated 1 (HYOU1) and mesencephalic astrocyte-derived neurotrophic factor (MANF). This is interesting because we have recently reported that ER stress-induced UPR occurs in the liver of dairy cows during early lactation [8] as evident from induction of XBP1, HSP5A, HERPUD1, DNAJC3, PDIA4, inositol-requiring enzyme 1 (IRE1), protein kinase (RNA)-like endoplasmic reticulum kinase (PERK), activating trascription factor 6 ATF6 (ATF6), ER degradation enhancing alpha-mannosidase-like protein 1 (EDEM1), ATF4, BCL2 antagonist/killer 1 (BAK1), BCL2 associated X, apoptosis regulator (BAX), caspase 3 (CASP3), CASP8, CASP9, CASP12, tryptophanyl-tRNA synthetase (WARS) and DNA damage inducible transcript 3 (DDIT3)/C/EBP homologous transcription factor protein (CHOP). In line with this, Loor [45] identified a large number of XBP1 target genes as up-regulated in the liver of dairy cows during the transition from late pregnancy to lactation. The significance of ER stress in the liver of dairy cows is its putative causative role in the development of liver-associated diseases in high-yielding dairy cows [10], which impairs metabolic function of the liver, overall health status, and productive and reproductive performance. UPR target genes encode proteins that mediate protective cellular responses aiming to reduce ER stress and restore ER homeostasis. Therefore, typical proteins encoded by UPR target genes, which were identified as down-regulated by GSGME, are chaperones (e.g. HSPA5, PDIA4, HYOU1, CALR) and co-chaperones (e.g. DNAJC5G, DNAJB1, BAG3), both of which are implicated in the refolding of proteins, and components of the ER-associated degradation (ERAD) machinery (e.g. HERPUD1). The ERAD machinery is involved in the clearance of misfolded proteins that cannot be refolded in the ER and, therefore, are retrotranslocated to the cytosol, where they become degraded by the proteasome after being ubiquitinated by E3 ubiquitin ligases [46]. Down-regulation of these UPR target genes by GSGME is likely mediated by the identified down-regulation of XBP1. The spliced (s) XPB1 is a critical transcriptional regulator of ER stress response by inducing genes that cope with ER stress factors (ER chaperones, ERAD components) and stimulating phospholipid biosynthesis which leads to an expansion of the ER membrane [9, 47, 48]. Transcriptional regulation of ER stress-responsive genes by sXBP1 and other ER stress-sensitive transcription factors is mediated by binding to ER stress-dependent regulatory promoter motifs [e.g. endoplasmic reticulum stress element (ERSE)]. Functional ERSEs regulated by XBP1 were reported for the MANF and GADD45B genes [49], both of which were identified as down-regulated transcripts in the liver of cows fed GSGME. While GADD45B is localized in the mitochondria and is an activator of pro-survival p38 mitogen-activated protein kinase signaling, MANF is located in the luminal side of the ER and is proposed to help to remove misfolded proteins from the ER by degradation and/or enhancing protein folding [50]. Another ER stress-inducible protein identified as down-regulated by GSGME is PHLDA1/TDAG51, which encodes a protein promoting apoptotic cell death. Apoptosis is induced as consequence of ER stress in the case that ER stress-induced damage is overwhelming and homeostasis cannot be restored [51, 52]. The large proportion of ER stress-induced UPR target genes of total down-regulated transcripts was also reflected by GSEA, according to which ER lumen, ER part and ER were identified as enriched GO annotation terms.

Noteworthy, the chemokine ligands C-X-C motif chemokine ligand 14 (CXCL14) and C-C motif chemokine ligand 3 like 1 (CCL3L1) were also identified as transcripts down-regulated in the liver of cows fed GSGME. These two chemokine ligands belong to a family of about 50 chemokines which as a common feature are key regulators of leukocyte chemotaxis, migration and function, thus playing fundamental roles both in physiological and pathological immune responses, including inflammatory processes [53]. Inflammation is also induced as a consequence of ER stress through IRE1-mediated activation of nuclear factor kappa B (NF-κB) [9, 54]. NF-κB plays a key role in regulating the transcription of a large set of genes involved in all aspects of inflammation (e.g. chemokines, proinflammatory cytokines, inflammatory enzymes, adhesion molecules and various receptors) [55]. Thus, the observed down-regulation of inflammatory chemokines in the liver of cows fed GSGME is not only an indicator of inhibition of hepatic inflammation but likely also of inhibition of ER stress by GSGME. In line with the assumption of an inhibition of hepatic inflammation by GSGME is a further finding of transcriptome analysis that the APP SAA4 was one of the genes down-regulated by GSGME. Hepatic synthesis of APPs, like SAA, HP, ceruloplasmin, and C-reactive protein, is greatly induced during systemic inflammation [7] triggered by pro-inflammatory cytokines [56]. In line with the view that high-yielding dairy cows suffer from systemic inflammation in the days after parturition, several studies have demonstrated that APPs are elevated in blood of cows during this phase, even in the absence of clinical signs of disease [7, 57, 58]. Thus, in order to substantiate our observation from transcriptome analysis that GSGME is able to attenuate the acute phase response of the liver, we determined the concentrations of SAA and HP in plasma of cows. In fact, the concentrations of both APPs were reduced in plasma of cows fed GSGME confirming our assumption that GSGME inhibits hepatic inflammation.

miRNAs were also identified as differentially regulated transcripts by GSGME in our transcriptome analysis and single miRNAs can regulate the expression of a large number of protein-coding target mRNAs, mainly at the posttranscriptional level. This is mediated by binding to complementary mRNA sequences, thereby causing their degradation or repression of protein translation, and, thus, inhibition of gene expression. Due to the great regulatory potential of miRNAs for regulating gene expression, we performed bioinformatic target prediction. Interestingly, the 185 target mRNAs predicted for mir-376c, which was up-regulated by GSGME, included several inflammatory chemokines, chemokine receptors, interleukins (ILs) and IL receptors [CCL15, CCL28, C-X9-C motif containing 4 (CMC4), CCR9, IL33, IL20RB]. Noteworthy, the target mRNAs predicted for mir-376c also included genes involved critically in the UPR including DDIT3/CHOP, eukaryotic translation initiation factor 2A (EIF2A) and the chaperone HSPD1. Although the predicted UPR target genes were not identical with the UPR target genes identified as differentially regulated, these findings strengthen our observation that GSGME causes down-regulation of ER stress target genes. Interestingly, CHOP is regulated by all branches of the UPR, in particular by ATF6, and is a powerful inducer of apoptosis during ER stress [59], while EIF2A encodes the initiator of protein translation eIF2α and inhibition of eIF2α phosphorylation in response to ER stress has long been known to be a cytoprotective mechanism, because inhibition of translation reduces global protein synthesis and thus work load of the ER [60]. Considering that mRNAs from up-regulated miRNAs are targeted for degradation and thus less transcribed, indicates that expression of genes involved in immune responses and critical genes of the UPR are inhibited by GSGME.

Nevertheless, we have recently reported that hepatic mRNA abundances of UPR target genes determined by qPCR analysis, such as ATF4, BAK1, BAX, CASP3, DDIT3, EDEM1, HSPA5, PDIA4 and XBP1, are not different between cows fed GSGME and control cows [13], because statistical evaluation of these data indicated no significant effect. Despite this, it was particularly striking that qPCR analyses showed a marked and consistent reduction in the mRNA abundances of all UPR target genes by 44% in average of all genes (variation between 25–65%). In addition, our recent study revealed that GSGME causes a strong and significant down-regulation of FGF21 in the liver of these cows. FGF21 is an important metabolic hormone regulating fatty acid oxidation and ketogenesis [61] and recent evidence indicated that FGF21 acts also as a stress hormone and is induced as a consequence of ER stress [62]. Thus, it is not surprising that FGF21 in the liver of dairy cows is dramatically induced during early lactation [63–65], because ER stress and various other stressors (negative energy balance, microbial pathogens) are present during the periparturient phase.

In connection with our results from transcriptome analysis, we are confident to postulate that GSGME is able to inhibit ER stress in the liver of dairy cows. Although we have no direct evidence for this, it is possible that attenuation of ER stress and inflammation was responsible for an increased utilization of energy and nutrients in these cows as reported recently [13]. Immune system activation is an energy-demanding process that necessitates a reallocation of nutrients and energy from dispensable functions such as growth and production [7]. It is well known that even subclinical inflammation increases the requirement of energy and amino acids, e.g. for the production of APPs and moreover has adverse effects on metabolism, e.g. by an increase of plasma cortisol [6, 66, 67]. The hypothesis that milk production is increased by an attenuation of inflammation has been confirmed in several studies in which supplementation of dairy cows with non-steroidal anti-inflammatory drugs during early lactation caused an increased milk yield [68–70]. The observed inhibition of ER stress and the parallel increase of milk yield by GSGME [13] is noteworthy regarding the relatively low amount of GSGME fed to the cows (1% of DM in the TMR). Other studies dealing with the effect of grape products in ruminants (cows or ewes) used markedly higher concentrations, such as 5 kg dried GM per cow and day [71], 10% grape residue silage of feed DM [72], and 300 g GS per ewe and day [73]. Despite the feeding of much higher amounts of grape products in these studies, only one study observed a slight improvement of milk yield compared to the control group [71]. However, none of these studies investigated the effect of grape products on ER stress and inflammatory signaling pathways, but on methanogenesis and intraruminal and total tract nutrient digestibility. Therefore, further studies dealing with the effects of grape products, with particular consideration of dose–response relationships, on ER stress and inflammatory pathways in the liver of high-yielding dairy cows during the transition period are required, in order to confirm potential beneficial effects of grape products on these pathways and to figure out the optimum supplementary dose.

A further striking observation from transcriptome analysis was that the most enriched GO terms associated with the genes up-regulated by GSGME are dealing with cell cycle regulation, such as M phase, cell cycle phase, mitotic cell phase, microtubule cytoskeleton, mitosis, nuclear and cell division. This is due to the fact that many of the proteins encoded by the up-regulated genes, like topoisomerase (DNA) II alpha (TOP2A), cyclin dependent kinase inhibitor 3 (CDKN3), stathmin 1 (STMN1), epithelial cell transforming 2 (ECT2), DEP domain containing 1 (DEPDC1), centromere protein A (CENPA), CENPF, CENPO, cytoskeleton associated protein 2 (CKAP2), kinesin family member 11 (KIF11), KIF20A, KIF4A, KIF20B, KIF15, BUB1B mitotic checkpoint serine/threonine kinase B (BUB1B), growth arrest specific 2 like 3 (GAS2L3), structural maintenance of chromosomes 4 (SMC4), SMC2, SPC25 NDC80 kinetochore complex component (SPC25), cyclin A2 (CCNA2), NDC80 kinetochore complex component (NUF2), β-tubulin (TUBB) and many others, have important biological functions within mitosis, cell cycle arrest, mitotic spindle organization, cytokinesis, mitotic chromosome condensation, metaphase/anaphase transition, chromosome organization, regulation of cyclin-dependent kinases and nucleosome assembly. For instance, KIF11, KIF20A, KIF4A, KIF20B and KIF15 encode proteins of the kinesin superfamily, a group of microtubule-dependent molecular motors. Proteins of the kinesin superfamily provide force for intracellular transport and cell division and are essential for mitosis and meiosis [74]. An important role during mitosis also plays topoisomerase IIα, encoded by the most strongly up-regulated gene TOP2A (2.9-fold), in resolving anaphase bridges between sister chromatids to ensure that daughter cells receive only one copy of each chromosome [75]. In this regard, centromer proteins, like CENPA, CENPF, CENPO, all of which were also up-regulated by GSGME, are localized to centromeric DNA, also called kinetochores, throughout the cell cycle and ensure correct chromosome attachment to the microtubules, equal segregation of sister chromatids, and their movement to the opposite poles [76]. Also in agreement with the observation that genes involved in mitosis and cell cycle are induced by GSGME is that several genes encoding histone proteins, which play a role for nucleosome assembly and thus affect chromatin structure, were up-regulated by GSGME. Although it is difficult to estimate the precise biological implication of an up-regulation of genes involved in mitosis or cell cycle regulation by GSGME in the context of early-lactating dairy cows, this effect might be explained, at least in part, by the well-described effects of different polyphenolic compounds contained in GSGME on cell cycle regulation and apoptosis, effects that are made responsible for the anti-cancer activities of many polyphenols [77]. For instance, quercetin [78], curcurmin [79], ellagic acid [80], epigallocatechin-3-gallate [81] and resveratrol [82] were found to induce the critical cell cycle regulator p53 and, subsequently, cell cycle arrest and apoptosis in different cancer cells. On the other hand, induction of p53-mediated cell cycle arrest by polyphenolic compounds in normal cells allows complete repair of DNA damage before continuing with cellular division through p53-induced formation of different DNA repair proteins, like mutL homolog 1 and Rad51 recombinase [83, 84]. Due to the central role of p53 for cell cycle regulation and the large number of up-regulated genes involved in this process, it was not surprising to identify cell cycle as an enriched KEGG pathway.

Besides their anti-inflammatory properties, pronounced effects of polyphenols on lipid metabolism have been reported. In rodent models, it has been shown that dietary polyphenols are able to lower plasma lipid concentrations and prevent the development of fatty liver by influencing several pathways of lipid metabolism, including inhibition of lipogenesis and activation of β-oxidation [23, 85]. In dairy cows, hepatic lipid metabolism is a physiological key aspect of health in dairy cows. It has been well established that disturbances of hepatic lipid metabolism, such as a low rate of β-oxidation and a limited capacity of the liver for the secretion of lipids into the blood are critical events in the development of fatty liver and ketosis [86, 87]. Recently, it has been observed that hepatic metabolism of glycerol- and ether phospholipids is closely linked to plasma concentrations of NEFA, BHBA and glucose, three key factors of the metabolic status of dairy cows during early lactation [20]. Imhashly et al. [88] recently showed, using lipidomic analysis of plasma, that concentrations of some unsaturated PC, LPC and SM species (such as PC 36:4, PC 36:5, PC 36:6, LPC 18:1, LPC 18:2, LPC 18:3, SM 39:1, SM 43:3) in dairy cows are continuously increasing after birth. A common feature of these phospholipids is their requirement for the secretion of hepatic TAG as very low-density lipoprotein particles. Thus, an increased formation and secretion of these phospholipids after birth has been regarded as a means of the liver to prevent accumulation of lipids [88]. In the present study, we observed that concentrations of all the individual phospholipids, and even their molecular species, in plasma of dairy cows in week 1 postpartum are not influenced by feeding GSGME. As the greatest part of plasma phospholipids is synthesized in the liver, this finding strongly suggests that phospholipid metabolism in the liver was not influenced by polyphenols from GSGME. The finding that fatty acid moieties of plasma phospholipids were also not changed in the group of cows supplemented with GSGME indicates that polyphenols also did not influence hepatic desaturation and elongation of fatty acids. This finding is of relevance as the fatty acid composition of phospholipids not only influences properties of cellular membranes [89], but certain phospholipid-bound fatty acids such as arachidonic acid are serving also as precursors for the synthesis of pro-inflammatory eicosanoids [90]. The finding that supplementation of GSGME did not influence the concentrations of free cholesterol and cholesterol esters indicates that polyphenols do not modify hepatic cholesterol metabolism. This finding agrees with our recent study which showed that GSGME does not influence hepatic cholesterol concentration [13]. Ceramide and ceramide-derived sphingolipids are structural components of membranes. In plasma, ceramides are transported as components of low-density lipoproteins of hepatic origin [91]. Ceramides are of physiological relevance as their plasma concentrations have been linked to insulin resistance, oxidative stress, and inflammation [22, 92–94], conditions which are commonly observed in dairy cows during the transition period. Recently, Rico et al. [91] have shown that overweight dairy cows have increased plasma concentrations of ceramides and these are closely linked with the progression of insulin resistance. These authors suggested that ceramides may have a fundamental role in the homeorhetic adaptation to early lactation in dairy cows. Our lipidomic analysis revealed that polyphenols from GSGME do not influence plasma concentrations and the molecular profile of ceramides in plasma. Thus, we conclude that beneficial effects of GSGME on inflammation and ER stress in the liver were independent of metabolism of ceramides.

## Conclusion

The present findings from transcriptome analysis of the liver of cows fed GSGME during the transition period at 1 week postpartum indicates that polyphenol-rich feed components, such as GSGME, are able to down-regulate a large set of genes involved in ER stress-induced UPR and inflammatory processes. The observation that GSGME induces specific miRNAs, which are known to bind and thus degrade mRNAs encoding proteins of the UPR and inflammation, indicates that at least some of the GSGME effects on the hepatic transcriptome of dairy cows are mediated by miRNA-mRNA interactions. In contrast, transcriptome analysis of the liver of these cows did not reveal alterations in the expression of genes involved in important metabolic pathways, such as lipid metabolism. This finding is in agreement with our results from plasma lipid profiling demonstrating no differences in the concentrations of individual species of major and minor lipid classes between cows fed GSGME and control cows. Considering that both ER stress and inflammatory processes are considered to contribute to liver-associated diseases, which frequently occur during early lactation in high-yielding dairy cows, and to impair milk performance in dairy cows, it is likely that inhibition of ER stress and inflammation is responsible for the recently observed increase in milk yield of dairy cows fed GSGME.

## Additional files

Additional file 1: Table S1. Characteristics of gene-specific primers used for qPCR. (DOCX 17 kb)

Additional file 2: Table S2. Predicted mRNAs of the highly conserved differentially regulated miRNAs including gene names, total and 8mer, 7mer and 6mer sites and cumulative weighted context++ score. (DOCX 298 kb)

Additional file 3: Tables S3-S9. Concentrations of various lipid species in plasma (μM) of cows fed grape seed and grape marc meal extract (GSGME) and control cows at 1 week postpartum. (DOCX 45 kb)

## Abbreviations

APP: Acute phase protein
ATF6: Activating trascription factor 6 ATF6
BAG3: BCL2-associated anthanogene 3
BAK1: BCL2 antagonist/killer 1
BAX: BCL2 associated X, apoptosis regulator
BHBA: β-hydroxybutyrate
BUB1B: BUB1B mitotic checkpoint serine/threonine kinase B
CALR: Calreticulin
CASP3: Caspase 3
CCL3L1: C-C motif chemokine ligand 3 like 1
CCNA2: Cyclin A2
CDKN3: Cyclin dependent kinase inhibitor 3
cDNA: Complementary DNA
CE: Cholesteryl esters
CENPA: Centromere protein A
Cer: Ceramides
CHOP: C/EBP homologous transcription factor protein
CKAP2: Cytoskeleton associated protein 2
CMC4: C-X9-C motif containing 4
CP: Crude protein
cRNA: Complementary RNA
CV: Coefficient of variability
CXCL14: Chemokine ligand 14
DAVID: Database for Annotation, Visualization and Integrated Discovery
DDIT3: DNA damage inducible transcript 3 (DDIT3)
DEPDC1: DEP domain containing 1
DMI: Dry matter intake
DNAJB11: DnaJ (Hsp40) homolog, subfamily B, member 11
DNAJC5G: DnaJ (Hsp40) homolog, subfamily C, member 5G
ECT2: Epithelial cell transforming 2
EDEM1: ER degradation enhancing alpha-mannosidase-like protein 1
EIF2A: Eukaryotic translation initiation factor 2A
ER: Endoplasmic reticulum
ERAD: ER-associated degradation
ERSE: Endoplasmic reticulum stress elements
ESI-MS/MS: electrospray ionization tandem mass spectrometry
FC: Fold-change
FC: Free cholesterol
FDR: False discovery rates
FGF-21: Fibroblast-growth factor-21
GADD45B: Growth arrest and DNA damage inducible beta
GAS2L3: Growth arrest specific 2 like 3
GEO: Gene expression omnibus
GO: Gene ontology
GSEA: Gene set enrichment analysis
GSGME: Grape seed and grape marc meal extract
HERPUD1: Homocysteine inducible ER protein with ubiquitin like domain 1
HexCer: Hexosylceramides
HP: Haptoglobin
HSPA5: Heat shock 70 kDa protein 5
HYOU1: Hypoxia up-regulated 1 (HYOU1)
IL: Interleukin
KEGG: Kyoto encyclopedia of genes and genomes
KFB: Centre of excellence for fluorescent bioanalytics
KIF11: Kinesin family member 11
LPC: Lysophosphatidylcholine
MANF: Mesencephalic astrocyte-derived neurotrophic factor
NE: Net energy
NEB: Negative energy balance
NEFA: Non-esterified fatty acids
NF-κB: Nuclear factor kappa B
NUF2: NDC80 kinetochore complex component
PC: Phosphatidylcholine
PDIA4: Protein disulfide isomerase family A, member 4
PE: Phosphatidylethanolamine
PE-P: PE-based plasmalogens
PERK: Protein kinase (RNA)-like endoplasmic reticulum kinase
PHLDA1: Pleckstrin homology-like domain, family A, member 1
PI: Phosphatidylinositol
PPP1R3C: Protein phosphatase 1 regulatory subunit 3C
qPCR: Quantitative real-time polymerase chain reaction
RIN: RNA integrity number
RMA: Robust multichip analysis
SAA: Serum amyloid A
SM: Sphingomyelin
SMC4: Structural maintenance of chromosomes 4
SPC25: SPC25 NDC80 kinetochore complex component (SPC25)
ss: Single-stranded
STMN1: Stathmin 1
TAG: Triacylglycerols
TMR: Total mixed ration
TPO2A: Topoisomerase (DNA) II alpha
TUBB: β-tubulin
UPR: Unfolded protein response
WARS: Tryptophanyl-tRNA synthetase
XBP1: X-box binding protein 1

## References

[1] Drackley JK (1999). Biology of dairy cows during the transition period: the final frontier?. J Dairy Sci.

[2] Drackley JK, Overton TR, Douglas GN (2001). Adaptations of glucose and long-chain fatty acid metabolism in liver of dairy cows during the periparturient period. J Dairy Sci.

[3] Plaizier JC, Krause DO, Gozho GN, McBride BW (2008). Subacute ruminal acidosis in dairy cows: the physiological causes, incidence and consequences. Vet J.

[4] Vels L, Rontved CM, Bjerring M, Ingvartsen KL (2009). Cytokine and acute phase protein gene expression in repeated liver biopsies of dairy cows with a lipopolysaccharide-induced mastitis. J Dairy Sci.

[5] Zebeli Q, Metzler-Zebeli BU (2012). Interplay between rumen digestive disorders and diet-induced inflammation in dairy cattle. Res Vet Sci..

[6] Bionaz M, Trevisi E, Calamari L, Librandi F, Ferrari A, Bertoni G (2007). Plasma paraoxonase, health, inflammatory conditions, and liver function in transition dairy cows. J Dairy Sci.

[7] Bradford BJ, Yuan K, Farney JK, Mamedova LK, Carpenter AJ (2015). Invited review: inflammation during the transition to lactation: New adventures with an old flame. J Dairy Sci.

[8] Gessner DK, Schlegel G, Ringseis R, Schwarz FJ, Eder K (2014). Up-regulation of endoplasmic reticulum stress induced genes of the unfolded protein response in the liver of periparturient dairy cows. BMC Vet Res.

[9] Cnop MF, Foufelle F, Velloso LA (2012). Endoplasmic reticulum stress, obesity and diabetes. Trends Mol Med.

[10] Ringseis R, Gessner DK, Eder K (2015). Molecular insights into the mechanisms of liver-associated diseases in early-lactating dairy cows: hypothetical role of endoplasmic reticulum stress. J Anim Physiol Anim Nutr (Berl).

[11] Xia EQ, Deng GF, Guo YJ, Li HB (2010). Biological activities of polyphenols from grapes. Int J Mol Sci.

[12] Landete JM (2012). Updated knowledge about polyphenols: functions, bioavailability, metabolism, and health. Crit Rev Food Sci Nutr.

[13] Gessner DK, Koch C, Romberg FJ, Winkler A, Dusel G, Herzog E, Most E, Eder K (2015). The effect of grape seed and grape marc meal extract on milk performance and the expression of genes of endoplasmic reticulum stress and inflammation in the liver of dairy cows in early lactation. J Dairy Sci.

[14] Romier B, Schneider YJ, Larondelle Y, During A (2009). Dietary polyphenols can modulate the intestinal inflammatory response. Nutr Rev.

[15] Vendrame S, Klimis-Zacas D (2015). Anti-inflammatory effect of anthocyanins via modulation of nuclear factor-κB and mitogen-activated protein kinase signaling cascades. Nutr Rev.

[16] Gessner DK, Ringseis R, Eder K (2016). Potential of plant polyphenols to combat oxidative stress and inflammatory processes in farm animals. J Anim Physiol Anim Nutr (Berl).

[17] Loor JJ, Dann HM, Everts RE, Oliveira R, Green CA, Guretzky NA, Rodriguez-Zas SL, Lewin HA, Drackley JK (2005). Temporal gene expression profiling of liver from periparturient dairy cows reveals complex adaptive mechanisms in hepatic function. Physiol Genomics.

[18] Loor JJ, Dann HM, Guretzky NA, Everts RE, Oliveira R, Green CA, Litherland NB, Rodriguez-Zas SL, Lewin HA, Drackley JK (2006). Plane of nutrition prepartum alters hepatic gene expression and function in dairy cows as assessed by longitudinal transcript and metabolic profiling. Physiol Genomics.

[19] Loor JJ, Everts RE, Bionaz M, Dann HM, Morin DE, Oliveira R, Rodriguez-Zas SL, Drackley JK, Lewin HA (2007). Nutrition-induced ketosis alters metabolic and signaling gene networks in liver of periparturient dairy cows. Physiol Genomics.

[20] Ha NT, Gross JJ, van Dorland A, Tetens J, Thaller G, Schlather M, Bruckmaier R, Simianer H (2015). Gene-based mapping and pathway analysis of metabolic traits in dairy cows. PLoS One.

[21] Hamada Y, Nagasaki H, Fujiya A, Seino Y, Shang QL, Suzuki T, Hashimoto H, Oiso Y (2014). Involvement of de novo ceramide synthesis in pro-inflammatory adipokine secretion and adipocyte-macrophage interaction. J Nutr Biochem.

[22] Gomez-Muñoz A, Presa N, Gomez-Larrauri A, Rivera IG, Trueba M, Ordoñez M (2016). Control of inflammatory responses by ceramide, sphingosine 1-phosphate and ceramide 1-phosphate. Prog Lipid Res.

[23] Aguirre L, Portillo MP, Hijona E, Bujanda L (2014). Effects of resveratrol and other polyphenols in hepatic steatosis. World J Gastroenterol.

[24] Gessner DK, Schlegel G, Keller J, Schwarz FJ, Ringseis R, Eder K (2013). Expression of target genes of nuclear factor E2-related factor 2 in the liver of dairy cows in the transition period and at different stages of lactation. J Dairy Sci.

[25] GfE (German Society of Nutrition Physiology) (2001). Empfehlungen zur Energie- und Nährstoffversorgung der Milchkühe und Aufzuchtrinder.

[26] VDLUFA (Verband Deutscher Landwirtschaftlicher Untersuchungs- und Forschungsanstalten) (2007). VDLUFA-Methodenbuch. Band III: Die chemische Untersuchung von Futtermitteln. Ergänzungslieferungen von 1983, 1988, 1992, 1997, 2004, 2006, 2007.

[27] Winkler A, Weber F, Ringseis R, Eder K, Dusel G (2015). Determination of polyphenol and crude nutrient content and nutrient digestibility of dried and ensiled white and red wine grape pomace cultivars. Arch Anim Nutr.

[28] Seker E (2002). The determination of the energy values of some ruminant feeds by using digestibility trial and gas test. Revue Méd Vét.

[29] Edgar R, Domrachev M, Lash AE (2002). Gene expression omnibus: NCBI gene expression and hybridization array data repository. Nucleic Acids Res.

[30] Irizarry RA, Hobbs B, Collin F, Beazer-Barclay YD, Antonellis KJ, Scherf U, Speed TP (2003). Exploration, normalization, and summaries of high density oligonucleotide array probe level data. Biostatistics.

[31] Lewis BP, Burge CB, Bartel DP (2005). Conserved seed pairing, often flanked by adenosines, indicates that thousands of human genes are microRNA targets. Cell.

[32] Agarwal V, Bell GW, Nam JW, Bartel DP (2015). Predicting effective microRNA target sites in mammalian mRNAs. Elife.

[33] da Huang W, Sherman BT, Lempicki RA (2009). Systematic and integrative analysis of large gene lists using DAVID bioinformatics resources. Nat Protoc.

[34] Huang DW, Sherman BT, Lempicki RA (2009). Bioinformatics enrichment tools: paths toward the comprehensive functional analysis of large gene lists. Nucleic Acids Res.

[35] Vandesompele J, De Preter K, Pattyn F, Poppe B, Van Roy N, De Paepe A, Speleman F (2002). Accurate normalization of real-time quantitative RT-PCR data by geometric averaging of multiple internal control genes. Genome Biol.

[36] Bligh EG, Dyer WJ (1959). A rapid method of total lipid extraction and purification. Can J Biochem Physiol.

[37] Liebisch G, Lieser B, Rathenberg J, Drobnik W, Schmitz G (2004). High-throughput quantification of phosphatidylcholine and sphingomyelin by electrospray ionization tandem mass spectrometry coupled with isotope correction algorithm. Biochim Biophys Acta.

[38] Liebisch G, Binder M, Schifferer R, Langmann T, Schulz B, Schmitz G (2006). High throughput quantification of cholesterol and cholesteryl ester by electrospray ionization tandem mass spectrometry (ESI-MS/MS). Biochim Biophys Acta.

[39] Liebisch G, Drobnik W, Lieser B, Schmitz G (2002). High-throughput quantification of lysophosphatidylcholine by electrospray ionization tandem mass spectrometry. Clin Chem.

[40] Liebisch G, Drobnik W, Reil M, Trumbach B, Arnecke R, Olgemoller B, Roscher A, Schmitz G (1999). Quantitative measurement of different ceramide species from crude cellular extracts by electrospray ionization tandem mass spectrometry (ESI-MS/MS). J Lipid Res.

[41] Brügger B, Erben G, Sandhoff R, Wieland FT, Lehmann WD (1997). Quantitative analysis of biological membrane lipids at the low picomole level by nano-electrospray ionization tandem mass spectrometry. Proc Natl Acad Sci U S A.

[42] Matyash V, Liebisch G, Kurzchalia TV, Shevchenko A, Schwudke D (2008). Lipid extraction by methyl-tertbutylether for high-throughput lipidomics. J Lipid Res.

[43] Zemski Berry KA, Murphy RC (2004). Electrospray ionization tandem mass spectrometry of glycerophosphoethanolamine plasmalogen phospholipids. J Am Soc Mass Spectrom.

[44] Liebisch G, Vizcaino JA, Kofeler H, Trotzmuller M, Griffiths WJ, Schmitz G, Spener F, Wakelam MJ (2013). Shorthand notation for lipid structures derived from mass spectrometry. J Lipid Res.

[45] Loor JJ (2010). Genomics of metabolic adaptations in the peripartal cow. Animal.

[46] Lemus L, Goder V (2014). Regulation of endoplasmic reticulum-associated protein degradation (ERAD) by ubiquitin. Cells.

[47] Marciniak SJ, Ron D (2006). Endoplasmic reticulum stress signaling in disease. Physiol Rev.

[48] Fu S, Watkins SM, Hotamisligil GS (2012). The role of endoplasmic reticulum in hepatic lipid homeostasis and stress signaling. Cell Metab.

[49] Mizobuchi N, Hoseki J, Kubota H, Toyokuni S, Nozaki J, Naitoh M, Koizumi A, Nagata K (2007). ARMET is a soluble ER protein induced by the unfolded protein response via ERSE-II element. Cell Struct Funct.

[50] Liu H, Tang X, Gong L (2015). Mesencephalic astrocyte-derived neurotrophic factor and cerebral dopamine neurotrophic factor: New endoplasmic reticulum stress response proteins. Eur J Pharmacol.

[51] Breckenridge DG, Germain M, Mathai JP, Nguyen M, Shore GC (2003). Regulation of apoptosis by endoplasmic reticulum pathways. Oncogene.

[52] Rutkowski DT, Kaufman RJ (2004). A trip to the ER: coping with stress. Trends Cell Biol.

[53] Proudfoot AE, Uguccioni M (2016). Modulation of chemokine responses: synergy and cooperativity. Front Immunol.

[54] Momoi T (2004). Caspases involved in ER stress-mediated cell death. J Chem Neuroanat.

[55] Barnes PJ, Karin M (1997). Nuclear factor-kappaB: a pivotal transcription factor in chronic inflammatory diseases. N Engl J Med.

[56] Venteclef N, Jakobsson T, Steffensen KR, Treuter E (2011). Metabolic nuclear receptor signaling and the inflammatory acute phase response. Trends Endocrinol Metab.

[57] Graugnard DE, Moyes KM, Trevisi E, Khan MJ, Keisler D, Drackley JK, Bertoni G, Loor JJ (2013). Liver lipid content and inflammometabolic indices in peripartal dairy cows are altered in response to prepartal energy intake and postpartal intramammary inflammatory challenge. J Dairy Sci.

[58] Akbar H, Grala TM, Vailati Riboni M, Cardoso FC, Verkerk G, McGowan J, Macdonald K, Webster J, Schutz K, Meier S, Matthews L, Roche JR, Loor JJ (2015). Body condition score at calving affects systemic and hepatic transcriptome indicators of inflammation and nutrient metabolism in grazing dairy cows. J Dairy Sci.

[59] Morishima N, Nakanishi K, Nakano A (2011). Activating transcription factor-6 (ATF6) mediates apoptosis with reduction of myeloid cell leukemia sequence 1 (Mcl-1) protein via induction of WW domain binding protein 1. J Biol Chem.

[60] Holcik M, Sonenberg N (2005). Translational control in stress and apoptosis. Nat Rev Mol Cell Biol.

[61] Inagaki T, Dutchak P, Zhao G, Ding X, Gautron L, Parameswara V, Li Y, Goetz R, Mohammadi M, Esser V, Elmquist JK, Gerard RD, Burgess SC, Hammer RE, Mangelsdorf DJ, Kliewer SA (2007). Endocrine regulation of the fasting response by PPARα-mediated induction of fibroblast growth factor 21. Cell Metab.

[62] Kim KH, Lee MS (2014). FGF21 as a stress hormone: the roles of FGF21 in stress adaptation and the treatment of metabolic diseases. Diabetes Metab J.

[63] Carriquiry M, Weber WJ, Fahrenkrug SC, Crooker BA (2009). Hepatic gene expression in multiparous Holstein cows treated with bovine somatotropin and fed n-3 fatty acids in early lactation. J Dairy Sci.

[64] Schoenberg KM, Giesy SL, Harvatine KJ, Waldron MR, Cheng C, Kharitonenkov A, Boisclair YR (2011). Plasma FGF21 is elevated by the intense lipid mobilization of lactation. Endocrinology.

[65] Schlegel G, Ringseis R, Keller J, Schwarz FJ, Windisch W, Eder K (2013). Expression of fibroblast growth factor 21 in the liver of dairy cows in the transition period and during lactation. J Anim Physiol Anim Nutr (Berl).

[66] Bertoni G, Trevisi E, Han X, Bionaz M (2008). Effects of inflammatory conditions on liver activity in puerperium period and consequences for performance in dairy cows. J Dairy Sci.

[67] Trevisi E, Bertoni G, Lombardelli R, Minuti A (2013). Relation of inflammation and liver function with the plasma cortisol response to adrenocorticotropin in early lactating dairy cows. J Dairy Sci.

[68] Bertoni G, Trevisi E, Piccioli-Cappelli F (2004). Effects of acetyl-salicylate used in post-calving of dairy cows. Vet Res Commun.

[69] Farney JK, Mamedova LK, Coetzee JF, Minton JE, Hollis LC, Bradford BJ (2013). Sodium salicylate treatment in early lactation increases whole-lactation milk and milk fat yield in mature dairy cows. J Dairy Sci.

[70] Carpenter AJ, Ylioja CM, Vargas CF, Mamedova LK, Mendonca LG, Coetzee JF, Hollis LC, Gehring R, Bradford BJ (2016). Hot topic: early postpartum treatment of commercial dairy cows with nonsteroidal antiinflammatory drugs increases whole-lactation milk yield. J Dairy Sci.

[71] Moate PJ, Williams SR, Torok VA, Hannah MC, Ribaux BE, Tavendale MH, Eckard RJ, Jacobs JL, Auldist MJ, Wales WJ (2014). Grape marc reduces methane emissions when fed to dairy cows. J Dairy Sci.

[72] Santos NW, Santos GTD, Silva-Kazama DC, Grande PA, Pintro PM, de Marchi FE, Jobim CC, Petit HV (2014). Production, composition and antioxidants in milk of dairy cows fed diets containing soybean oil and grape seed residue silage. Livest Sci.

[73] Nudda A, Correddu F, Marzano A, Battacone G, Nicolussi P, Bonelli P, Pulina G (2015). Effects of diets containing grape seed, linseed, or both on milk production traits, liver and kidney activities, and immunity of lactating dairy ewes. J Dairy Sci.

[74] Niwa S (2015). Kinesin superfamily proteins and the regulation of microtubule dynamics in morphogenesis. Anat Sci Int.

[75] Porter AC, Farr CJ (2004). Topoisomerase II: untangling its contribution at the centromere. Chromosome Res.

[76] Perpelescu M, Fukagawa T (2011). The ABCs of CENPs. Chromosoma.

[77] Mocanu MM, Nagy P, Szöllősi J (2015). Chemoprevention of breast cancer by dietary polyphenols. Molecules.

[78] López-Vélez M, Martínez-Martínez F, Del Valle-Ribes C (2003). The study of phenolic compounds as natural antioxidants in wine. Crit Rev Food Sci Nutr.

[79] Jee SH, Shen SC, Tseng CR, Chiu HC, Kuo ML (1998). Curcumin induces a p53-dependent apoptosis in human basal cell carcinoma cells. J Invest Dermatol.

[80] Mertens-Talcott SU, Bomser JA, Romero C, Talcott ST, Percival SS (2005). Ellagic acid potentiates the effect of quercetin on p21waf1/cip1, p53, and MAP-kinases without affecting intracellular generation of reactive oxygen species in vitro. J Nutr.

[81] Yamauchi R, Sasaki K, Yoshida K (2009). Identification of epigallocatechin-3-gallate in green tea polyphenols as a potent inducer of p53-dependent apoptosis in the human lung cancer cell line A549. Toxicol In Vitro.

[82] Hu YQ, Wang J, Wu JH (2016). Administration of resveratrol enhances cell-cycle arrest followed by apoptosis in DMBA-induced skin carcinogenesis in male Wistar rats. Eur Rev Med Pharmacol Sci.

[83] Chen J, Sadowski I (2005). Identification of the mismatch repair genes PMS2 and MLH1 as p53 target genes by using serial analysis of binding elements. Proc Natl Acad Sci U S A.

[84] Arias-Lopez C, Lazaro-Trueba I, Kerr P, Lord CJ, Dexter T, Iravani M, Ashworth A, Silva A (2006). p53 modulates homologous recombination by transcriptional regulation of the RAD51 gene. EMBO Rep.

[85] Bladé C, Arola L, Salvadó MJ (2010). Hypolipidemic effects of proanthocyanidins and their underlying biochemical and molecular mechanisms. Mol Nutr Food Res.

[86] Gruffat D, Durand D, Chilliard Y, Williams P, Bauchart D (1997). Hepatic gene expression of apolipoprotein B100 during early lactation in underfed, high producing dairy cows. J Dairy Sci.

[87] Hayirli A (2006). The role of exogenous insulin in the complex of hepatic lipidosis and ketosis associated with insulin resistance phenomenon in postpartum dairy cattle. Vet Res Commun.

[88] Imhasly S, Bieli C, Naegeli H, Nyström L, Ruetten M, Gerspach C (2015). Blood plasma lipidome profile of dairy cows during the transition period. BMC Vet Res.

[89] Bruni A, van Dijck PW, de Gier J (1975). The role of phospholipid acyl chains in the activation of mitochondrial ATPase complex. Biochim Biophys Acta.

[90] Wenzel SE (1997). Arachidonic acid metabolites: mediators of inflammation in asthma. Pharmacotherapy.

[91] Rico JE, Bandaru VV, Dorskind JM, Haughey NJ, McFadden JW (2015). Plasma ceramides are elevated in overweight Holstein dairy cows experiencing greater lipolysis and insulin resistance during the transition from late pregnancy to early lactation. J Dairy Sci.

[92] Bikman BT (2012). A role for sphingolipids in the pathophysiology of obesity-induced inflammation. Cell Mol Life Sci.

[93] Chavez JA, Siddique MM, Wang ST, Ching J, Shayman JA, Summers SA (2014). Ceramides and Glucosylceramides are independent antagonists of insulin signaling. J Biol Chem.

[94] Maceyka M, Spiegel S (2014). Sphingolipid metabolites in inflammatory disease. Nature.

