# Supplementary material for: Analysis of hepatic transcript profile and plasma lipid profile in early lactating dairy cows fed grape seed and grape marc meal extract
Source: BMC Genomics. 2017 Mar 23;18:253. doi: 10.1186/s12864-017-3638-1 (PMC5364584; doi:10.1186/s12864-017-3638-1)
Supplement: Supplementary file 1 — Characteristics of gene-specific primers used for qPCR. (DOCX 17 kb) [file 12864_2017_3638_MOESM1_ESM.docx]

**Table S1 Characteristics of gene-specific primers used for qPCR**

| Gene | Forward primer (from 5` to 3`)  Reverse primer (from 5` to 3`) | Product  size (bp) | NCBI  GenBank |
| --- | --- | --- | --- |
| *BAG3* | TCGTCCTCCTCCTCAGCCAG  TCTGGTACTCGCCCTGCTGA | 187 | NM_001082471 |
| *BUB1B* | AAGGGTAGGTTGCAGAAGGAGG  TGATCCGCCCCTGCCTTAAA | 200 | NM_001145173 |
| *CCNA2* | ATTTGCCGTCAGTTATCGCTGC  TTGACTGTTGTGCGTGCTGTG | 177 | NM_001075123 |
| *CCNL1* | ACCCGAGGATAGACAACAGGC  TGTTCCAGATCGACTCCGCC | 172 | NM_001206311 |
| *CENPA* | CTCCTCTCCTTACACGCCGG  TCAACCAGAGACTTGGGCGG | 124 | NM_001205380 |
| *CENPF* | ACCAGGCATCAGCCAAGTGT  AGGAACGCTGTTGACGGGAG | 168 | NM_001256586 |
| *CKAP2* | CGCAGCCACACTACGGTGAA  CAGGCCGGGTAACTGCTGAG | 151 | NM_001098032 |
| *CXCL14* | AAAGAGCCGAGCACAGCACA  GCTGTAGCGGATCTTGGGTCC | 186 | NM_001034410 |
| *DNAJB11* | ACCCCTCGTCAGCAAGACAG  CCTCGCCACGGGTTTGTTTC | 126 | NM_001034268 |
| *ECT2* | AGAGGGACCTGTGCTTGAAAAGG  AGAGTCTGCCAAGCTTGTACTGC | 131 | NM_001097573 |
| *ESCO2* | TCACCACCGATTTGTGGAGGG  ATAACTCGGGTCACGTGGCAG | 112 | NM_001101182 |
| *GLCE* | CCCAAGGGCTGCTTTATGGC  TCTCCAGAACCACAGACACGC | 178 | NM_174070 |
| *HMMR* | TGCCACACCACACACCAAGT  AGGTTATGAGCAGCGACTTCCC | 122 | NM_001206621 |
| *KIF20A* | GGAGGTCGTAGTCTCCCCCAT  GTAACAAGGGCCTGACCCTCA | 176 | NM_001046288 |
| *MANF* | CTGCCGTGAAGCAAGAGGCA  TCCACAGGGATGTGGTGGGA | 120 | NM_001101211 |
| *PHLDA1* | TCCAACTCGGCCTGAAGAGG  CTACTTGATTTGGTGTGGGGCG | 134 | NM_001105631 |
| *PRR11* | CCGTCTCTGCCACCGAGAAC  GTCTGGATGCTCACCACCCG | 157 | NM_001098137 |
| *RRM2* | GGGCTCAGCTTAGCGGACAA  CCACACTGGGGGCAGATAGC | 133 | NM_001244181 |
| *SAA4* | TCAAGGAGGCTGTGCAAGGG  GCAGCCTCGTAGTTCCCTCG | 122 | NM_001040505 |
| *SOCS3* | ATCCCTCTGGTGTTGAGCCG CCAGGAACTCCCGAATGGGC | 136 | NM_174466 |
| *SPC25* | CCCTAAGCATCCTGATCGCCC  TGCGAGGCACTCAAGACGAG | 100 | NM_001034230 |
| *STMN1* | CTCGGACGGAGCAGGGTTTT  TGAGGCACGCTTCTCCAGTTC | 122 | NM_001034790 |
| *TUBB* | GCCCCGTATACACTTTGAGGC  CGGTCCAGTTGTAGGTCGCT | 187 | NM_001046549 |
| *UAP1* | GCAATTCCCCGCAGTGCTAC  ACTGGATTGGCACATCATTGGC | 94 | NM_001046404 |
