# Supplementary material for: Analysis of hepatic transcript profile and plasma lipid profile in early lactating dairy cows fed grape seed and grape marc meal extract
Source: BMC Genomics. 2017 Mar 23;18:253. doi: 10.1186/s12864-017-3638-1 (PMC5364584; doi:10.1186/s12864-017-3638-1)
Supplement: Supplementary file 2 — Predicted mRNAs of the highly conserved differentially regulated miRNAs including gene names, total and 8mer, 7mer and 6mer sites and cumulative weighted context++ score. (DOCX 298 kb) [file 12864_2017_3638_MOESM2_ESM.docx]

**Table S2 Predicted mRNAs of the highly conserved differentially regulated miRNAs including gene names, total and 8mer, 7mer and 6mer sites and cumulative weighted context++ score.**

**Predicted target mRNAs of mir-376c**

| Ortholog of target gene | Representative transcript | Gene name | 3P-seq tags + 5 | Total sites | 8mer sites | 7mer-m8 sites | 7mer-A1 sites | 6mer sites | Representative miRNA | Cumulative weighted context++ score |
| --- | --- | --- | --- | --- | --- | --- | --- | --- | --- | --- |
| RP11-67H2.1 | ENST00000521500.2 | Uncharacterized protein | 5 | 2 | 1 | 1 | 0 | 0 | bta-miR-376c | -0.91 |
| CDR1as | CDR1as | circular RNA CDR1as | 1 | 1 | 0 | 1 | 0 | 0 | bta-miR-376c | -0.81 |
| ACMSD | ENST00000356140.5 | aminocarboxymuconate semialdehyde decarboxylase | 19 | 1 | 1 | 0 | 0 | 1 | bta-miR-376c | -0.76 |
| CMC4 | ENST00000369484.3 | C-x(9)-C motif containing 4 homolog (S. cerevisiae) | 87 | 1 | 1 | 0 | 0 | 0 | bta-miR-376c | -0.69 |
| EGFL6 | ENST00000380602.3 | EGF-like-domain, multiple 6 | 5 | 1 | 1 | 0 | 0 | 0 | bta-miR-376c | -0.59 |
| STMN1 | ENST00000455785.2 | stathmin 1 | 30059 | 1 | 1 | 0 | 0 | 0 | bta-miR-376c | -0.56 |
| AL354808.2 | ENST00000601204.1 | | 5 | 1 | 1 | 0 | 0 | 0 | bta-miR-376c | -0.56 |
| ENAM | ENST00000396073.3 | enamelin | 5 | 2 | 1 | 0 | 1 | 1 | bta-miR-376c | -0.54 |
| DAZ4 | ENST00000382290.3 | deleted in azoospermia 4 | 5 | 4 | 0 | 0 | 4 | 0 | bta-miR-376c | -0.54 |
| CTSL | ENST00000343150.5 | cathepsin L | 4520 | 1 | 1 | 0 | 0 | 0 | bta-miR-376c | -0.53 |
| C9orf153 | ENST00000339137.3 | chromosome 9 open reading frame 153 | 5 | 1 | 1 | 0 | 0 | 1 | bta-miR-376c | -0.53 |
| FUT9 | ENST00000302103.5 | fucosyltransferase 9 (alpha (1,3) fucosyltransferase) | 5 | 3 | 1 | 2 | 0 | 4 | bta-miR-376c | -0.51 |
| ARG1 | ENST00000368087.3 | arginase 1 | 5 | 1 | 1 | 0 | 0 | 0 | bta-miR-376c | -0.51 |
| MT-ND4L | ENST00000361335.1 | mitochondrially encoded NADH dehydrogenase 4L | 8219 | 1 | 1 | 0 | 0 | 2 | bta-miR-376c | -0.5 |
| C1GALT1C1 | ENST00000304661.5 | C1GALT1-specific chaperone 1 | 645 | 1 | 1 | 0 | 0 | 0 | bta-miR-376c | -0.5 |
| CLLU1 | ENST00000378485.1 | chronic lymphocytic leukemia up-regulated 1 | 5 | 2 | 0 | 2 | 0 | 0 | bta-miR-376c | -0.49 |
| DLX4 | ENST00000240306.3 | distal-less homeobox 4 | 74 | 1 | 1 | 0 | 0 | 1 | bta-miR-376c | -0.47 |
| FGF1 | ENST00000360966.5 | fibroblast growth factor 1 (acidic) | 88 | 2 | 1 | 0 | 1 | 1 | bta-miR-376c | -0.47 |
| DTD2 | ENST00000310850.4 | D-tyrosyl-tRNA deacylase 2 (putative) | 924 | 1 | 1 | 0 | 0 | 0 | bta-miR-376c | -0.43 |
| FOLR3 | ENST00000445078.2 | folate receptor 3 (gamma) | 5 | 1 | 0 | 1 | 0 | 0 | bta-miR-376c | -0.43 |
| IRF2BP2 | ENST00000366610.3 | interferon regulatory factor 2 binding protein 2 | 8690 | 1 | 1 | 0 | 0 | 0 | bta-miR-376c | -0.42 |
| IFT88 | ENST00000319980.6 | intraflagellar transport 88 homolog (Chlamydomonas) | 48 | 1 | 1 | 0 | 0 | 0 | bta-miR-376c | -0.42 |
| CCDC59 | ENST00000256151.7 | coiled-coil domain containing 59 | 140 | 1 | 1 | 0 | 0 | 0 | bta-miR-376c | -0.42 |
| NHLH2 | ENST00000320238.3 | nescient helix loop helix 2 | 16 | 2 | 1 | 0 | 1 | 0 | bta-miR-376c | -0.41 |
| SLC16A14 | ENST00000295190.4 | solute carrier family 16, member 14 | 34 | 3 | 1 | 0 | 2 | 0 | bta-miR-376c | -0.41 |
| NINL | ENST00000278886.6 | ninein-like | 121 | 1 | 1 | 0 | 0 | 1 | bta-miR-376c | -0.4 |
| ACADL | ENST00000233710.3 | acyl-CoA dehydrogenase, long chain | 5 | 1 | 1 | 0 | 0 | 0 | bta-miR-376c | -0.4 |
| NMUR2 | ENST00000255262.3 | neuromedin U receptor 2 | 5 | 1 | 1 | 0 | 0 | 0 | bta-miR-376c | -0.4 |
| MAGEA9B | ENST00000243314.5 | melanoma antigen family A, 9B | 5 | 1 | 1 | 0 | 0 | 0 | bta-miR-376c | -0.39 |
| CYGB | ENST00000293230.5 | cytoglobin | 10 | 1 | 1 | 0 | 0 | 0 | bta-miR-376c | -0.39 |
| SERINC4 | ENST00000299969.6 | serine incorporator 4 | 9 | 1 | 1 | 0 | 0 | 0 | bta-miR-376c | -0.39 |
| SPARCL1 | ENST00000282470.6 | SPARC-like 1 (hevin) | 5 | 1 | 1 | 0 | 0 | 0 | bta-miR-376c | -0.39 |
| PTPN22 | ENST00000460620.1 | protein tyrosine phosphatase, non-receptor type 22 (lymphoid) | 5 | 1 | 1 | 0 | 0 | 0 | bta-miR-376c | -0.39 |
| NCAPD3 | ENST00000534548.2 | non-SMC condensin II complex, subunit D3 | 990 | 1 | 1 | 0 | 0 | 0 | bta-miR-376c | -0.39 |
| RAB39A | ENST00000320578.2 | RAB39A, member RAS oncogene family | 15 | 1 | 1 | 0 | 0 | 0 | bta-miR-376c | -0.39 |
| ZNF197 | ENST00000383745.2 | zinc finger protein 197 | 53 | 1 | 1 | 0 | 0 | 0 | bta-miR-376c | -0.38 |
| ETV1 | ENST00000430479.1 | ets variant 1 | 160 | 2 | 1 | 1 | 0 | 1 | bta-miR-376c | -0.38 |
| LRRTM4 | ENST00000409088.3 | leucine rich repeat transmembrane neuronal 4 | 5 | 1 | 1 | 0 | 0 | 0 | bta-miR-376c | -0.38 |
| C5orf27 | ENST00000436592.1 | chromosome 5 open reading frame 27 | 5 | 1 | 1 | 0 | 0 | 0 | bta-miR-376c | -0.37 |
| TMTC4 | ENST00000376234.3 | transmembrane and tetratricopeptide repeat containing 4 | 37 | 2 | 1 | 1 | 0 | 0 | bta-miR-376c | -0.37 |
| MAGI2 | ENST00000419488.1 | membrane associated guanylate kinase, WW and PDZ domain containing 2 | 43 | 1 | 1 | 0 | 0 | 0 | bta-miR-376c | -0.37 |
| OR2B2 | ENST00000303324.2 | olfactory receptor, family 2, subfamily B, member 2 | 5 | 1 | 0 | 0 | 1 | 0 | bta-miR-376c | -0.36 |
| GFRA1 | ENST00000439649.3 | GDNF family receptor alpha 1 | 69 | 3 | 2 | 1 | 0 | 0 | bta-miR-376c | -0.36 |
| AC008964.1 | ENST00000593965.1 | | 5 | 2 | 0 | 1 | 1 | 0 | bta-miR-376c | -0.36 |
| HSD17B6 | ENST00000554643.1 | hydroxysteroid (17-beta) dehydrogenase 6 | 25 | 1 | 1 | 0 | 0 | 0 | bta-miR-376c | -0.36 |
| RP11-664D7.4 | ENST00000512294.3 | HCG1787533; Uncharacterized protein | 14 | 1 | 0 | 1 | 0 | 0 | bta-miR-376c | -0.36 |
| UBFD1 | ENST00000395878.3 | ubiquitin family domain containing 1 | 6826 | 1 | 1 | 0 | 0 | 0 | bta-miR-376c | -0.35 |
| SCRG1 | ENST00000296506.3 | stimulator of chondrogenesis 1 | 5 | 1 | 1 | 0 | 0 | 1 | bta-miR-376c | -0.35 |
| DGCR6 | ENST00000608842.1 | DiGeorge syndrome critical region gene 6 | 5 | 2 | 0 | 1 | 1 | 0 | bta-miR-376c | -0.35 |
| MAMDC2 | ENST00000377182.4 | MAM domain containing 2 | 365 | 1 | 1 | 0 | 0 | 0 | bta-miR-376c | -0.35 |
| PPP3R1 | ENST00000234310.3 | protein phosphatase 3, regulatory subunit B, alpha | 117 | 1 | 1 | 0 | 0 | 0 | bta-miR-376c | -0.35 |
| GCSAML | ENST00000366488.4 | germinal center-associated, signaling and motility-like | 5 | 1 | 1 | 0 | 0 | 0 | bta-miR-376c | -0.34 |
| HSPD1 | ENST00000388968.3 | heat shock 60kDa protein 1 (chaperonin) | 42 | 1 | 1 | 0 | 0 | 0 | bta-miR-376c | -0.34 |
| SPATA31A6 | ENST00000332857.6 | SPATA31 subfamily A, member 6 | 5 | 1 | 1 | 0 | 0 | 0 | bta-miR-376c | -0.34 |
| RP11-116D17.1 | ENST00000547948.1 | HCG2038717; Uncharacterized protein | 5 | 1 | 0 | 0 | 1 | 0 | bta-miR-376c | -0.34 |
| NKX3-1 | ENST00000380871.4 | NK3 homeobox 1 | 240 | 3 | 2 | 1 | 0 | 0 | bta-miR-376c | -0.34 |
| RNF7 | ENST00000273480.3 | ring finger protein 7 | 377 | 1 | 1 | 0 | 0 | 0 | bta-miR-376c | -0.34 |
| SPATA31A3 | ENST00000356699.5 | SPATA31 subfamily A, member 3 | 5 | 1 | 1 | 0 | 0 | 0 | bta-miR-376c | -0.34 |
| SPATA31A1 | ENST00000377647.3 | SPATA31 subfamily A, member 1 | 5 | 1 | 1 | 0 | 0 | 0 | bta-miR-376c | -0.34 |
| ARID2 | ENST00000457135.1 | AT rich interactive domain 2 (ARID, RFX-like) | 891 | 1 | 1 | 0 | 0 | 2 | bta-miR-376c | -0.34 |
| DDIT3 | ENST00000547303.1 | DNA-damage-inducible transcript 3 | 624 | 1 | 0 | 1 | 0 | 0 | bta-miR-376c | -0.34 |
| SPATA31A7 | ENST00000355045.2 | SPATA31 subfamily A, member 7 | 5 | 1 | 1 | 0 | 0 | 0 | bta-miR-376c | -0.33 |
| SPATA31A5 | ENST00000377621.1 | SPATA31 subfamily A, member 5 | 5 | 1 | 1 | 0 | 0 | 0 | bta-miR-376c | -0.33 |
| SPATA31A4 | ENST00000429767.1 | SPATA31 subfamily A, member 4 | 5 | 1 | 1 | 0 | 0 | 0 | bta-miR-376c | -0.33 |
| CXXC4 | ENST00000394767.2 | CXXC finger protein 4 | 121 | 2 | 1 | 1 | 0 | 1 | bta-miR-376c | -0.33 |
| SPATA31A2 | ENST00000456183.2 | SPATA31 subfamily A, member 2 | 5 | 1 | 1 | 0 | 0 | 0 | bta-miR-376c | -0.33 |
| ABCG2 | ENST00000515655.1 | ATP-binding cassette, sub-family G (WHITE), member 2 | 20 | 1 | 1 | 0 | 0 | 0 | bta-miR-376c | -0.33 |
| TPD52L3 | ENST00000344545.5 | tumor protein D52-like 3 | 5 | 1 | 1 | 0 | 0 | 0 | bta-miR-376c | -0.33 |
| NECAB1 | ENST00000417640.2 | N-terminal EF-hand calcium binding protein 1 | 5 | 1 | 0 | 1 | 0 | 1 | bta-miR-376c | -0.33 |
| EDNRA | ENST00000339690.5 | endothelin receptor type A | 35 | 2 | 1 | 1 | 0 | 1 | bta-miR-376c | -0.32 |
| SYNJ2 | ENST00000449859.2 | synaptojanin 2 | 369 | 1 | 1 | 0 | 0 | 0 | bta-miR-376c | -0.32 |
| LPCAT2 | ENST00000262134.5 | lysophosphatidylcholine acyltransferase 2 | 360 | 2 | 2 | 0 | 0 | 0 | bta-miR-376c | -0.32 |
| VPS52 | ENST00000482399.1 | vacuolar protein sorting 52 homolog (S. cerevisiae) | 124 | 1 | 1 | 0 | 0 | 1 | bta-miR-376c | -0.32 |
| AICDA | ENST00000229335.6 | activation-induced cytidine deaminase | 5 | 1 | 1 | 0 | 0 | 1 | bta-miR-376c | -0.32 |
| NKRF | ENST00000371527.1 | NFKB repressing factor | 607 | 1 | 1 | 0 | 0 | 0 | bta-miR-376c | -0.31 |
| MOB4 | ENST00000233892.4 | MOB family member 4, phocein | 123 | 1 | 1 | 0 | 0 | 1 | bta-miR-376c | -0.31 |
| GAS2 | ENST00000454584.2 | growth arrest-specific 2 | 13 | 1 | 1 | 0 | 0 | 0 | bta-miR-376c | -0.31 |
| MCF2 | ENST00000520602.1 | MCF.2 cell line derived transforming sequence | 5 | 1 | 1 | 0 | 0 | 0 | bta-miR-376c | -0.31 |
| CXorf66 | ENST00000370540.1 | chromosome X open reading frame 66 | 5 | 1 | 0 | 1 | 0 | 0 | bta-miR-376c | -0.31 |
| CAPN2 | ENST00000295006.5 | calpain 2, (m/II) large subunit | 1090 | 1 | 1 | 0 | 0 | 0 | bta-miR-376c | -0.31 |
| CYP2C18 | ENST00000285979.6 | cytochrome P450, family 2, subfamily C, polypeptide 18 | 5 | 1 | 1 | 0 | 0 | 1 | bta-miR-376c | -0.3 |
| TTC37 | ENST00000358746.2 | tetratricopeptide repeat domain 37 | 296 | 1 | 1 | 0 | 0 | 0 | bta-miR-376c | -0.3 |
| HECA | ENST00000367658.2 | headcase homolog (Drosophila) | 783 | 1 | 1 | 0 | 0 | 0 | bta-miR-376c | -0.3 |
| ZRSR1 | ENST00000391338.1 | zinc finger (CCCH type), RNA-binding motif and serine/arginine rich 1 | 17 | 1 | 0 | 1 | 0 | 0 | bta-miR-376c | -0.3 |
| EIF2A | ENST00000460851.1 | eukaryotic translation initiation factor 2A, 65kDa | 3166 | 1 | 0 | 1 | 0 | 0 | bta-miR-376c | -0.3 |
| FUT11 | ENST00000394790.1 | fucosyltransferase 11 (alpha (1,3) fucosyltransferase) | 622 | 2 | 1 | 1 | 0 | 0 | bta-miR-376c | -0.29 |
| SCAMP4 | ENST00000316097.8 | secretory carrier membrane protein 4 | 223 | 1 | 0 | 1 | 0 | 0 | bta-miR-376c | -0.29 |
| BLOC1S6 | ENST00000220531.3 | biogenesis of lysosomal organelles complex-1, subunit 6, pallidin | 1754 | 1 | 1 | 0 | 0 | 1 | bta-miR-376c | -0.29 |
| RP11-293M10.1 | ENST00000553510.1 | Uncharacterized protein | 5 | 1 | 0 | 1 | 0 | 0 | bta-miR-376c | -0.29 |
| MDFIC | ENST00000393486.1 | MyoD family inhibitor domain containing | 601 | 3 | 1 | 2 | 0 | 0 | bta-miR-376c | -0.29 |
| CCL15 | ENST00000354059.4 | chemokine (C-C motif) ligand 15 | 135 | 1 | 0 | 0 | 1 | 0 | bta-miR-376c | -0.28 |
| CAMK2N1 | ENST00000375078.3 | calcium/calmodulin-dependent protein kinase II inhibitor 1 | 6631 | 1 | 0 | 1 | 0 | 0 | bta-miR-376c | -0.28 |
| PAX3 | ENST00000350526.4 | paired box 3 | 7 | 1 | 1 | 0 | 0 | 0 | bta-miR-376c | -0.28 |
| RP1-228P16.5 | ENST00000547602.1 | Uncharacterized protein | 5 | 1 | 0 | 1 | 0 | 0 | bta-miR-376c | -0.28 |
| BLZF1 | ENST00000367808.3 | basic leucine zipper nuclear factor 1 | 73 | 1 | 1 | 0 | 0 | 0 | bta-miR-376c | -0.28 |
| PKIA | ENST00000396418.2 | protein kinase (cAMP-dependent, catalytic) inhibitor alpha | 542 | 3 | 0 | 2 | 1 | 4 | bta-miR-376c | -0.28 |
| HSPE1-MOB4 | ENST00000604458.1 | HSPE1-MOB4 readthrough | 123 | 1 | 1 | 0 | 0 | 1 | bta-miR-376c | -0.28 |
| PCDHB3 | ENST00000231130.2 | protocadherin beta 3 | 5 | 1 | 1 | 0 | 0 | 0 | bta-miR-376c | -0.28 |
| C12orf66 | ENST00000544871.1 | chromosome 12 open reading frame 66 | 98 | 1 | 1 | 0 | 0 | 0 | bta-miR-376c | -0.28 |
| OR51E2 | ENST00000396950.3 | olfactory receptor, family 51, subfamily E, member 2 | 5 | 1 | 1 | 0 | 0 | 0 | bta-miR-376c | -0.27 |
| ATG12 | ENST00000500945.2 | autophagy related 12 | 1822 | 1 | 0 | 1 | 0 | 0 | bta-miR-376c | -0.27 |
| LGR5 | ENST00000266674.5 | leucine-rich repeat containing G protein-coupled receptor 5 | 137 | 1 | 1 | 0 | 0 | 0 | bta-miR-376c | -0.27 |
| FAM102B | ENST00000370035.3 | family with sequence similarity 102, member B | 392 | 1 | 1 | 0 | 0 | 0 | bta-miR-376c | -0.27 |
| TMF1 | ENST00000398559.2 | TATA element modulatory factor 1 | 349 | 1 | 1 | 0 | 0 | 3 | bta-miR-376c | -0.27 |
| PDGFD | ENST00000393158.2 | platelet derived growth factor D | 52 | 1 | 1 | 0 | 0 | 0 | bta-miR-376c | -0.27 |
| KCTD1 | ENST00000579973.1 | potassium channel tetramerization domain containing 1 | 33 | 1 | 1 | 0 | 0 | 0 | bta-miR-376c | -0.27 |
| C11orf87 | ENST00000327419.6 | chromosome 11 open reading frame 87 | 5 | 2 | 2 | 0 | 0 | 1 | bta-miR-376c | -0.27 |
| C1orf21 | ENST00000235307.6 | chromosome 1 open reading frame 21 | 935 | 3 | 1 | 0 | 2 | 0 | bta-miR-376c | -0.27 |
| RP11-455G16.1 | ENST00000326780.3 | Uncharacterized protein | 5 | 1 | 0 | 1 | 0 | 0 | bta-miR-376c | -0.26 |
| KRBOX4 | ENST00000360017.5 | KRAB box domain containing 4 | 162 | 1 | 0 | 0 | 1 | 2 | bta-miR-376c | -0.26 |
| BIVM | ENST00000448849.2 | basic, immunoglobulin-like variable motif containing | 440 | 1 | 0 | 1 | 0 | 2 | bta-miR-376c | -0.26 |
| C15orf32 | ENST00000556865.1 | chromosome 15 open reading frame 32 | 5 | 1 | 0 | 0 | 1 | 0 | bta-miR-376c | -0.26 |
| CLDN1 | ENST00000295522.3 | claudin 1 | 153 | 3 | 0 | 2 | 1 | 0 | bta-miR-376c | -0.26 |
| ELK3 | ENST00000228741.3 | ELK3, ETS-domain protein (SRF accessory protein 2) | 2719 | 1 | 1 | 0 | 0 | 0 | bta-miR-376c | -0.25 |
| FAM111A | ENST00000528737.1 | family with sequence similarity 111, member A | 5 | 1 | 0 | 1 | 0 | 0 | bta-miR-376c | -0.25 |
| LYRM5 | ENST00000556402.1 | LYR motif containing 5 | 40 | 2 | 0 | 1 | 1 | 0 | bta-miR-376c | -0.25 |
| ECT2L | ENST00000423192.1 | epithelial cell transforming sequence 2 oncogene-like | 5 | 1 | 1 | 0 | 0 | 0 | bta-miR-376c | -0.25 |
| KCNJ14 | ENST00000391884.1 | potassium inwardly-rectifying channel, subfamily J, member 14 | 29 | 1 | 1 | 0 | 0 | 0 | bta-miR-376c | -0.25 |
| IL33 | ENST00000381434.3 | interleukin 33 | 8 | 1 | 0 | 1 | 0 | 0 | bta-miR-376c | -0.25 |
| ADAM12 | ENST00000368679.4 | ADAM metallopeptidase domain 12 | 758 | 2 | 1 | 1 | 0 | 0 | bta-miR-376c | -0.25 |
| MBIP | ENST00000416007.4 | MAP3K12 binding inhibitory protein 1 | 300 | 1 | 0 | 1 | 0 | 0 | bta-miR-376c | -0.25 |
| SLC7A14 | ENST00000231706.5 | solute carrier family 7, member 14 | 5 | 5 | 1 | 4 | 0 | 1 | bta-miR-376c | -0.24 |
| UBE2W | ENST00000517608.1 | ubiquitin-conjugating enzyme E2W (putative) | 102 | 2 | 1 | 0 | 1 | 1 | bta-miR-376c | -0.24 |
| HLA-DQA1 | ENST00000343139.5 | major histocompatibility complex, class II, DQ alpha 1 | 7 | 1 | 0 | 1 | 0 | 0 | bta-miR-376c | -0.24 |
| ZMAT3 | ENST00000311417.2 | zinc finger, matrin-type 3 | 459 | 2 | 1 | 0 | 1 | 0 | bta-miR-376c | -0.24 |
| DMP1 | ENST00000339673.6 | dentin matrix acidic phosphoprotein 1 | 5 | 1 | 0 | 1 | 0 | 0 | bta-miR-376c | -0.24 |
| RNF144B | ENST00000259939.3 | ring finger protein 144B | 17 | 2 | 1 | 1 | 0 | 0 | bta-miR-376c | -0.24 |
| C10orf111 | ENST00000378207.3 | chromosome 10 open reading frame 111 | 5 | 1 | 0 | 1 | 0 | 0 | bta-miR-376c | -0.24 |
| TTC6 | ENST00000476979.1 | tetratricopeptide repeat domain 6 | 5 | 1 | 0 | 1 | 0 | 0 | bta-miR-376c | -0.24 |
| MIER1 | ENST00000357692.2 | mesoderm induction early response 1, transcriptional regulator | 950 | 1 | 1 | 0 | 0 | 3 | bta-miR-376c | -0.24 |
| CCDC152 | ENST00000361970.5 | coiled-coil domain containing 152 | 5 | 1 | 1 | 0 | 0 | 0 | bta-miR-376c | -0.24 |
| PSPH | ENST00000275605.3 | phosphoserine phosphatase | 3231 | 1 | 0 | 0 | 1 | 1 | bta-miR-376c | -0.24 |
| SLC15A2 | ENST00000489711.1 | solute carrier family 15 (oligopeptide transporter), member 2 | 21 | 1 | 1 | 0 | 0 | 0 | bta-miR-376c | -0.24 |
| SUMO1 | ENST00000392246.2 | small ubiquitin-like modifier 1 | 180 | 1 | 0 | 1 | 0 | 0 | bta-miR-376c | -0.24 |
| CRISPLD1 | ENST00000262207.4 | cysteine-rich secretory protein LCCL domain containing 1 | 358 | 1 | 1 | 0 | 0 | 1 | bta-miR-376c | -0.24 |
| ASAH2B | ENST00000374007.1 | N-acylsphingosine amidohydrolase (non-lysosomal ceramidase) 2B | 5 | 2 | 0 | 1 | 1 | 0 | bta-miR-376c | -0.23 |
| KRTAP9-7 | ENST00000391354.1 | keratin associated protein 9-7 | 5 | 1 | 0 | 1 | 0 | 0 | bta-miR-376c | -0.23 |
| SALL1 | ENST00000566102.1 | sal-like 1 (Drosophila) | 95 | 1 | 0 | 1 | 0 | 0 | bta-miR-376c | -0.23 |
| PLXNC1 | ENST00000258526.4 | plexin C1 | 47 | 1 | 1 | 0 | 0 | 0 | bta-miR-376c | -0.23 |
| C7orf76 | ENST00000356686.1 | chromosome 7 open reading frame 76 | 5 | 1 | 0 | 1 | 0 | 0 | bta-miR-376c | -0.23 |
| MTFR1 | ENST00000458689.2 | mitochondrial fission regulator 1 | 1051 | 1 | 1 | 0 | 0 | 1 | bta-miR-376c | -0.23 |
| HTR7 | ENST00000371719.2 | 5-hydroxytryptamine (serotonin) receptor 7, adenylate cyclase-coupled | 12 | 2 | 0 | 2 | 0 | 0 | bta-miR-376c | -0.23 |
| MXRA7 | ENST00000355797.3 | matrix-remodelling associated 7 | 421 | 1 | 1 | 0 | 0 | 1 | bta-miR-376c | -0.23 |
| CHRNA3 | ENST00000326828.5 | cholinergic receptor, nicotinic, alpha 3 (neuronal) | 20 | 1 | 0 | 1 | 0 | 1 | bta-miR-376c | -0.23 |
| NT5C3A | ENST00000610140.1 | 5'-nucleotidase, cytosolic IIIA | 165 | 1 | 0 | 1 | 0 | 0 | bta-miR-376c | -0.23 |
| CBWD5 | ENST00000377384.1 | COBW domain containing 5 | 5 | 1 | 0 | 1 | 0 | 0 | bta-miR-376c | -0.22 |
| ANKRD30B | ENST00000358984.4 | ankyrin repeat domain 30B | 0 | 1 | 0 | 1 | 0 | 0 | bta-miR-376c | -0.22 |
| CHRNA9 | ENST00000310169.2 | cholinergic receptor, nicotinic, alpha 9 (neuronal) | 20 | 1 | 0 | 1 | 0 | 0 | bta-miR-376c | -0.22 |
| SYCP2 | ENST00000357552.3 | synaptonemal complex protein 2 | 28 | 1 | 0 | 0 | 1 | 0 | bta-miR-376c | -0.22 |
| GABPA | ENST00000354828.3 | GA binding protein transcription factor, alpha subunit 60kDa | 8 | 1 | 1 | 0 | 0 | 0 | bta-miR-376c | -0.22 |
| SGMS1 | ENST00000361781.2 | sphingomyelin synthase 1 | 100 | 1 | 0 | 1 | 0 | 1 | bta-miR-376c | -0.22 |
| FKBP5 | ENST00000536438.1 | FK506 binding protein 5 | 32 | 1 | 1 | 0 | 0 | 0 | bta-miR-376c | -0.22 |
| IDNK | ENST00000405990.3 | idnK, gluconokinase homolog (E. coli) | 137 | 1 | 0 | 0 | 1 | 0 | bta-miR-376c | -0.22 |
| SOCS6 | ENST00000397942.3 | suppressor of cytokine signaling 6 | 140 | 3 | 1 | 0 | 2 | 2 | bta-miR-376c | -0.22 |
| CCR9 | ENST00000357632.2 | chemokine (C-C motif) receptor 9 | 5 | 1 | 0 | 1 | 0 | 0 | bta-miR-376c | -0.22 |
| H2BFM | ENST00000355016.3 | H2B histone family, member M | 5 | 1 | 0 | 1 | 0 | 0 | bta-miR-376c | -0.22 |
| ZNF383 | ENST00000352998.3 | zinc finger protein 383 | 147 | 2 | 1 | 0 | 1 | 0 | bta-miR-376c | -0.22 |
| FAM72A | ENST00000367128.3 | family with sequence similarity 72, member A | 5 | 1 | 0 | 1 | 0 | 1 | bta-miR-376c | -0.22 |
| LDHAL6A | ENST00000280706.2 | lactate dehydrogenase A-like 6A | 5 | 1 | 0 | 1 | 0 | 0 | bta-miR-376c | -0.22 |
| MYO3B | ENST00000334231.6 | myosin IIIB | 5 | 1 | 0 | 1 | 0 | 0 | bta-miR-376c | -0.22 |
| EPS15 | ENST00000371730.2 | epidermal growth factor receptor pathway substrate 15 | 325 | 1 | 1 | 0 | 0 | 1 | bta-miR-376c | -0.22 |
| CHGB | ENST00000378961.4 | chromogranin B (secretogranin 1) | 57 | 1 | 0 | 0 | 1 | 1 | bta-miR-376c | -0.22 |
| ATPIF1 | ENST00000465645.1 | ATPase inhibitory factor 1 | 15846 | 1 | 1 | 0 | 0 | 1 | bta-miR-376c | -0.22 |
| AL589765.1 | ENST00000442233.2 | Uncharacterized protein; cDNA FLJ36032 fis, clone TESTI2017069 | 5 | 1 | 0 | 1 | 0 | 0 | bta-miR-376c | -0.22 |
| RP11-204N11.1 | ENST00000555187.1 | Uncharacterized protein | 5 | 2 | 0 | 1 | 1 | 0 | bta-miR-376c | -0.22 |
| TTLL3 | ENST00000383827.1 | tubulin tyrosine ligase-like family, member 3 | 10 | 1 | 1 | 0 | 0 | 0 | bta-miR-376c | -0.21 |
| NID1 | ENST00000366595.3 | nidogen 1 | 303 | 2 | 1 | 0 | 1 | 1 | bta-miR-376c | -0.21 |
| FTCDNL1 | ENST00000420922.2 | formiminotransferase cyclodeaminase N-terminal like | 5 | 1 | 0 | 1 | 0 | 0 | bta-miR-376c | -0.21 |
| POC1B | ENST00000378528.2 | POC1 centriolar protein B | 61 | 1 | 1 | 0 | 0 | 0 | bta-miR-376c | -0.21 |
| ODF2L | ENST00000359242.3 | outer dense fiber of sperm tails 2-like | 164 | 1 | 0 | 0 | 1 | 0 | bta-miR-376c | -0.21 |
| RGS21 | ENST00000417209.2 | regulator of G-protein signaling 21 | 5 | 1 | 0 | 1 | 0 | 0 | bta-miR-376c | -0.21 |
| IL20RB | ENST00000329582.4 | interleukin 20 receptor beta | 16 | 1 | 0 | 1 | 0 | 0 | bta-miR-376c | -0.21 |
| KIAA1143 | ENST00000296121.4 | KIAA1143 | 5 | 1 | 0 | 0 | 1 | 1 | bta-miR-376c | -0.21 |
| C4BPA | ENST00000367070.3 | complement component 4 binding protein, alpha | 11 | 1 | 1 | 0 | 0 | 0 | bta-miR-376c | -0.21 |
| SLC44A2 | ENST00000335757.5 | solute carrier family 44 (choline transporter), member 2 | 720 | 1 | 1 | 0 | 0 | 0 | bta-miR-376c | -0.21 |
| GCH1 | ENST00000491895.2 | GTP cyclohydrolase 1 | 502 | 1 | 0 | 1 | 0 | 1 | bta-miR-376c | -0.21 |
| CCL28 | ENST00000361115.4 | chemokine (C-C motif) ligand 28 | 5 | 1 | 0 | 1 | 0 | 0 | bta-miR-376c | -0.21 |
| CYP4F2 | ENST00000221700.6 | cytochrome P450, family 4, subfamily F, polypeptide 2 | 5 | 1 | 0 | 1 | 0 | 2 | bta-miR-376c | -0.21 |
| KCNJ16 | ENST00000589377.1 | potassium inwardly-rectifying channel, subfamily J, member 16 | 5 | 2 | 1 | 0 | 1 | 0 | bta-miR-376c | -0.21 |
| GLIPR1 | ENST00000266659.3 | GLI pathogenesis-related 1 | 623 | 1 | 1 | 0 | 0 | 1 | bta-miR-376c | -0.21 |
| C5orf54 | ENST00000408953.3 | chromosome 5 open reading frame 54 | 15 | 1 | 1 | 0 | 0 | 0 | bta-miR-376c | -0.21 |
| IGF2BP2 | ENST00000382199.2 | insulin-like growth factor 2 mRNA binding protein 2 | 5 | 1 | 0 | 1 | 0 | 0 | bta-miR-376c | -0.21 |
| ZNF345 | ENST00000589046.1 | zinc finger protein 345 | 24 | 1 | 0 | 0 | 1 | 0 | bta-miR-376c | -0.21 |
| ARL4A | ENST00000396663.1 | ADP-ribosylation factor-like 4A | 213 | 1 | 1 | 0 | 0 | 0 | bta-miR-376c | -0.21 |
| PTHLH | ENST00000395872.1 | parathyroid hormone-like hormone | 32 | 1 | 0 | 0 | 1 | 0 | bta-miR-376c | -0.21 |

**Predicted target mRNAs of mir-2345**

| Ortholog of target gene | Representative transcript | Gene name | 3P-seq tags + 5 | Total sites | 8mer sites | 7mer-m8 sites | 7mer-A1 sites | 6mer sites | Representative miRNA | Cumulative weighted context++ score |
| --- | --- | --- | --- | --- | --- | --- | --- | --- | --- | --- |
| PRR26 | ENST00000381489.5 | proline rich 26 | 5 | 3 | 1 | 2 | 0 | 2 | bta-miR-2345 | -1.7 |
| MYLK | ENST00000360772.3 | myosin light chain kinase | 2964 | 1* | 0 | 0 | 0 | 2 | bta-miR-2345 | -1 |
| CELF2 | ENST00000315874.4 | CUGBP, Elav-like family member 2 | 56 | 1* | 0 | 0 | 0 | 4 | bta-miR-2345 | -1 |
| RLIM | ENST00000332687.6 | ring finger protein, LIM domain interacting | 662 | 1* | 0 | 0 | 0 | 2 | bta-miR-2345 | -1 |
| SLC10A7 | ENST00000264986.3 | solute carrier family 10, member 7 | 77 | 3 | 2 | 0 | 1 | 1 | bta-miR-2345 | -0.79 |
| FAM155A | ENST00000375915.2 | family with sequence similarity 155, member A | 118 | 2 | 2 | 0 | 0 | 0 | bta-miR-2390 | -0.78 |
| MMP20 | ENST00000260228.2 | matrix metallopeptidase 20 | 5 | 2 | 2 | 0 | 0 | 0 | bta-miR-2345 | -0.76 |
| C4orf6 | ENST00000195455.2 | chromosome 4 open reading frame 6 | 5 | 1 | 1 | 0 | 0 | 0 | bta-miR-2345 | -0.71 |
| RP11-127H5.1 | ENST00000521923.1 | Uncharacterized protein | 5 | 1 | 1 | 0 | 0 | 0 | bta-miR-2345 | -0.7 |
| AJAP1 | ENST00000378191.4 | adherens junctions associated protein 1 | 5 | 3 | 2 | 0 | 1 | 2 | bta-miR-2390 | -0.69 |
| POU5F1B | ENST00000465342.2 | POU class 5 homeobox 1B | 5 | 2 | 0 | 1 | 1 | 0 | bta-miR-2345 | -0.63 |
| UCMA | ENST00000378681.3 | upper zone of growth plate and cartilage matrix associated | 5 | 2 | 2 | 0 | 0 | 0 | bta-miR-2390 | -0.62 |
| MCEE | ENST00000244217.5 | methylmalonyl CoA epimerase | 34 | 1 | 1 | 0 | 0 | 0 | bta-miR-2390 | -0.6 |
| ELAVL4 | ENST00000371824.1 | ELAV like neuron-specific RNA binding protein 4 | 5 | 3 | 2 | 0 | 1 | 0 | bta-miR-2390 | -0.59 |
| SCRG1 | ENST00000296506.3 | stimulator of chondrogenesis 1 | 5 | 2 | 1 | 1 | 0 | 0 | bta-miR-2390 | -0.58 |
| EMC4 | ENST00000267750.4 | ER membrane protein complex subunit 4 | 3844 | 1 | 1 | 0 | 0 | 0 | bta-miR-2390 | -0.56 |
| LHX1 | ENST00000254457.5 | LIM homeobox 1 | 15 | 2 | 1 | 0 | 1 | 1 | bta-miR-2390 | -0.55 |
| CREBRF | ENST00000540014.1 | CREB3 regulatory factor | 24 | 4 | 3 | 0 | 1 | 2 | bta-miR-2345 | -0.53 |
| SAMD12 | ENST00000409003.4 | sterile alpha motif domain containing 12 | 57 | 4 | 0 | 2 | 2 | 2 | bta-miR-2390 | -0.53 |
| RORB | ENST00000376896.3 | RAR-related orphan receptor B | 5 | 4 | 1 | 1 | 2 | 3 | bta-miR-2390 | -0.53 |
| ERP27 | ENST00000266397.2 | endoplasmic reticulum protein 27 | 5 | 2 | 1 | 0 | 1 | 0 | bta-miR-2390 | -0.53 |
| RP11-362K2.2 | ENST00000546977.1 | Protein LOC100506869 | 5 | 1 | 1 | 0 | 0 | 0 | bta-miR-2345 | -0.53 |
| DNASE1L1 | ENST00000369809.1 | deoxyribonuclease I-like 1 | 16 | 3 | 2 | 1 | 0 | 1 | bta-miR-2390 | -0.53 |
| AC015987.2 | ENST00000416501.1 | | 5 | 1 | 1 | 0 | 0 | 0 | bta-miR-2390 | -0.53 |
| NRXN1 | ENST00000342183.5 | neurexin 1 | 5 | 4 | 2 | 0 | 2 | 2 | bta-miR-2390 | -0.53 |
| NCK2 | ENST00000451463.2 | NCK adaptor protein 2 | 178 | 1 | 1 | 0 | 0 | 0 | bta-miR-2390 | -0.52 |
| IL12B | ENST00000231228.2 | interleukin 12B (natural killer cell stimulatory factor 2, cytotoxic lymphocyte maturation factor 2, p40) | 5 | 3 | 2 | 1 | 0 | 2 | bta-miR-2345 | -0.52 |
| PSMC2 | ENST00000435765.1 | proteasome (prosome, macropain) 26S subunit, ATPase, 2 | 1710 | 1 | 1 | 0 | 0 | 2 | bta-miR-2345 | -0.52 |
| ZNF121 | ENST00000586602.1 | zinc finger protein 121 | 5 | 1 | 1 | 0 | 0 | 0 | bta-miR-2345 | -0.52 |
| IFI44 | ENST00000370747.4 | interferon-induced protein 44 | 6 | 1 | 1 | 0 | 0 | 0 | bta-miR-2345 | -0.51 |
| ANKRD49 | ENST00000544253.1 | ankyrin repeat domain 49 | 32 | 2 | 1 | 1 | 0 | 0 | bta-miR-2390 | -0.51 |
| CLVS1 | ENST00000518592.1 | clavesin 1 | 5 | 1 | 1 | 0 | 0 | 3 | bta-miR-2390 | -0.5 |
| MAB21L2 | ENST00000317605.4 | mab-21-like 2 (C. elegans) | 63 | 3 | 1 | 1 | 1 | 0 | bta-miR-2390 | -0.5 |
| DHRS4L2 | ENST00000335125.6 | dehydrogenase/reductase (SDR family) member 4 like 2 | 5 | 1 | 1 | 0 | 0 | 0 | bta-miR-2390 | -0.49 |
| IGF2BP2 | ENST00000382199.2 | insulin-like growth factor 2 mRNA binding protein 2 | 5 | 4 | 4 | 0 | 0 | 0 | bta-miR-2390 | -0.49 |
| RP4-758J18.2 | ENST00000444362.1 | HCG20425, isoform CRA_a; Uncharacterized protein; cDNA FLJ53815 | 283 | 1 | 0 | 0 | 1 | 2 | bta-miR-2345 | -0.49 |
| DTD1 | ENST00000377452.3 | D-tyrosyl-tRNA deacylase 1 | 58 | 1 | 1 | 0 | 0 | 0 | bta-miR-2390 | -0.48 |
| ARCN1 | ENST00000534182.2 | archain 1 | 1139 | 2 | 1 | 0 | 1 | 0 | bta-miR-2390 | -0.48 |
| DZIP1 | ENST00000347108.3 | DAZ interacting zinc finger protein 1 | 360 | 2 | 1 | 1 | 0 | 0 | bta-miR-2390 | -0.48 |
| AAED1 | ENST00000375234.3 | AhpC/TSA antioxidant enzyme domain containing 1 | 5 | 3 | 0 | 0 | 3 | 0 | bta-miR-2345 | -0.47 |
| CWH43 | ENST00000226432.4 | cell wall biogenesis 43 C-terminal homolog (S. cerevisiae) | 5 | 1 | 1 | 0 | 0 | 0 | bta-miR-2345 | -0.47 |
| C15orf56 | ENST00000319503.3 | chromosome 15 open reading frame 56 | 5 | 1 | 1 | 0 | 0 | 0 | bta-miR-2345 | -0.46 |
| KIF20A | ENST00000394894.3 | kinesin family member 20A | 532 | 3 | 1 | 1 | 1 | 0 | bta-miR-2345 | -0.46 |
| HOXC9 | ENST00000303450.4 | homeobox C9 | 248 | 1 | 1 | 0 | 0 | 1 | bta-miR-2345 | -0.46 |
| RBFOX3 | ENST00000583458.1 | RNA binding protein, fox-1 homolog (C. elegans) 3 | 5 | 1 | 1 | 0 | 0 | 0 | bta-miR-2390 | -0.45 |
| KB-1980E6.3 | ENST00000523572.1 | Uncharacterized protein | 5 | 2 | 0 | 0 | 2 | 0 | bta-miR-2345 | -0.45 |
| GRB2 | ENST00000392563.1 | growth factor receptor-bound protein 2 | 2660 | 1 | 1 | 0 | 0 | 0 | bta-miR-2390 | -0.45 |
| MYL1 | ENST00000341685.4 | myosin, light chain 1, alkali; skeletal, fast | 5 | 1 | 1 | 0 | 0 | 0 | bta-miR-2345 | -0.45 |
| PINX1 | ENST00000426190.2 | PIN2/TERF1 interacting, telomerase inhibitor 1 | 224 | 1 | 1 | 0 | 0 | 0 | bta-miR-2345 | -0.45 |
| TOMM5 | ENST00000377773.5 | translocase of outer mitochondrial membrane 5 homolog (yeast) | 12658 | 1 | 1 | 0 | 0 | 0 | bta-miR-2345 | -0.45 |
| MGAM | ENST00000549489.2 | maltase-glucoamylase (alpha-glucosidase) | 5 | 2 | 2 | 0 | 0 | 1 | bta-miR-2390 | -0.45 |
| ARL8B | ENST00000419534.2 | ADP-ribosylation factor-like 8B | 664 | 2 | 1 | 1 | 0 | 0 | bta-miR-2345 | -0.44 |
| ATP6V0E2 | ENST00000425642.2 | ATPase, H+ transporting V0 subunit e2 | 85 | 1 | 1 | 0 | 0 | 0 | bta-miR-2390 | -0.44 |
| TMEM17 | ENST00000335390.5 | transmembrane protein 17 | 10 | 1 | 1 | 0 | 0 | 1 | bta-miR-2345 | -0.44 |
| URM1 | ENST00000372850.1 | ubiquitin related modifier 1 | 345 | 1 | 1 | 0 | 0 | 0 | bta-miR-2390 | -0.44 |
| KRIT1 | ENST00000394507.1 | KRIT1, ankyrin repeat containing | 574 | 2 | 2 | 0 | 0 | 1 | bta-miR-2345 | -0.44 |
| ENPP5 | ENST00000371383.2 | ectonucleotide pyrophosphatase/phosphodiesterase 5 (putative) | 15 | 1 | 1 | 0 | 0 | 1 | bta-miR-2390 | -0.43 |
| TMEM64 | ENST00000458549.2 | transmembrane protein 64 | 200 | 2 | 1 | 1 | 0 | 1 | bta-miR-2390 | -0.43 |
| MSTO1 | ENST00000245564.2 | misato 1, mitochondrial distribution and morphology regulator | 136 | 2 | 0 | 2 | 0 | 0 | bta-miR-2345 | -0.43 |
| KBTBD4 | ENST00000526005.1 | kelch repeat and BTB (POZ) domain containing 4 | 7 | 1 | 1 | 0 | 0 | 0 | bta-miR-2390 | -0.43 |
| TSPYL6 | ENST00000317802.7 | TSPY-like 6 | 5 | 1 | 1 | 0 | 0 | 0 | bta-miR-2390 | -0.43 |
| DRD5 | ENST00000304374.2 | dopamine receptor D5 | 5 | 2 | 1 | 0 | 1 | 0 | bta-miR-2345 | -0.43 |
| CTSE | ENST00000358184.2 | cathepsin E | 42 | 1 | 1 | 0 | 0 | 0 | bta-miR-2345 | -0.42 |
| LSAMP | ENST00000490035.2 | limbic system-associated membrane protein | 19 | 2 | 2 | 0 | 0 | 0 | bta-miR-2390 | -0.42 |
| AC120194.1 | ENST00000391684.1 | | 5 | 1 | 0 | 1 | 0 | 0 | bta-miR-2345 | -0.42 |
| PIM1 | ENST00000373509.5 | pim-1 oncogene | 2041 | 1 | 1 | 0 | 0 | 1 | bta-miR-2390 | -0.42 |
| C20orf173 | ENST00000246199.2 | chromosome 20 open reading frame 173 | 5 | 2 | 0 | 2 | 0 | 0 | bta-miR-2390 | -0.42 |
| RP11-382J12.1 | ENST00000499227.2 | Uncharacterized protein | 5 | 1 | 1 | 0 | 0 | 0 | bta-miR-2345 | -0.42 |
| ABHD17B | ENST00000333421.6 | abhydrolase domain containing 17B | 104 | 1 | 1 | 0 | 0 | 1 | bta-miR-2345 | -0.42 |
| C17orf58 | ENST00000536693.1 | chromosome 17 open reading frame 58 | 48 | 1 | 0 | 1 | 0 | 0 | bta-miR-2345 | -0.42 |
| ZFY | ENST00000383052.1 | zinc finger protein, Y-linked | 5 | 2 | 0 | 2 | 0 | 1 | bta-miR-2345 | -0.42 |
| GABRB1 | ENST00000295454.3 | gamma-aminobutyric acid (GABA) A receptor, beta 1 | 5 | 5 | 1 | 2 | 2 | 1 | bta-miR-2390 | -0.42 |
| TYW1B | ENST00000343721.5 | tRNA-yW synthesizing protein 1 homolog B (S. cerevisiae) | 5 | 2 | 0 | 0 | 2 | 0 | bta-miR-2345 | -0.41 |
| TIFAB | ENST00000537858.1 | TRAF-interacting protein with forkhead-associated domain, family member B | 5 | 1 | 1 | 0 | 0 | 2 | bta-miR-2390 | -0.41 |
| EIF3I | ENST00000373586.1 | eukaryotic translation initiation factor 3, subunit I | 236 | 1 | 1 | 0 | 0 | 0 | bta-miR-2390 | -0.41 |
| SEP15 | ENST00000401030.3 | Homo sapiens 15 kDa selenoprotein (SEP15), transcript variant 2, mRNA. | 2047 | 1 | 1 | 0 | 0 | 0 | bta-miR-2345 | -0.41 |
| H2AFY2 | ENST00000373255.4 | H2A histone family, member Y2 | 46 | 1 | 1 | 0 | 0 | 0 | bta-miR-2390 | -0.41 |
| C1QC | ENST00000374640.4 | complement component 1, q subcomponent, C chain | 5 | 1 | 1 | 0 | 0 | 0 | bta-miR-2345 | -0.41 |
| AL627171.2 | ENST00000595378.1 | HCG1786899; PRO2610; Uncharacterized protein | 5 | 1 | 0 | 1 | 0 | 2 | bta-miR-2390 | -0.41 |
| ARHGEF38 | ENST00000420470.2 | Rho guanine nucleotide exchange factor (GEF) 38 | 5 | 2 | 1 | 1 | 0 | 0 | bta-miR-2345 | -0.4 |
| RNF128 | ENST00000324342.3 | ring finger protein 128, E3 ubiquitin protein ligase | 17 | 2 | 1 | 0 | 1 | 0 | bta-miR-2345 | -0.4 |
| TENM2 | ENST00000519204.1 | teneurin transmembrane protein 2 | 162 | 5* | 1 | 2 | 1 | 1 | bta-miR-2390 | -0.4 |
| ARV1 | ENST00000310256.2 | ARV1 homolog (S. cerevisiae) | 705 | 1 | 1 | 0 | 0 | 0 | bta-miR-2390 | -0.4 |
| PYURF | ENST00000273968.4 | PIGY upstream reading frame | 103 | 1 | 0 | 1 | 0 | 0 | bta-miR-2390 | -0.4 |
| KSR2 | ENST00000425217.1 | kinase suppressor of ras 2 | 22 | 5 | 0 | 2 | 3 | 2 | bta-miR-2390 | -0.4 |
| ATP6V0D2 | ENST00000285393.3 | ATPase, H+ transporting, lysosomal 38kDa, V0 subunit d2 | 5 | 1 | 1 | 0 | 0 | 0 | bta-miR-2345 | -0.4 |
| SPOP | ENST00000393328.2 | speckle-type POZ protein | 883 | 1 | 1 | 0 | 0 | 0 | bta-miR-2390 | -0.4 |
| HOXA5 | ENST00000222726.3 | homeobox A5 | 63 | 1 | 1 | 0 | 0 | 1 | bta-miR-2345 | -0.39 |
| UROC1 | ENST00000290868.2 | urocanate hydratase 1 | 5 | 3 | 1 | 2 | 0 | 0 | bta-miR-2390 | -0.39 |
| INSL5 | ENST00000304526.2 | insulin-like 5 | 5 | 1 | 1 | 0 | 0 | 0 | bta-miR-2345 | -0.39 |
| CELF5 | ENST00000292672.2 | CUGBP, Elav-like family member 5 | 5 | 1 | 1 | 0 | 0 | 0 | bta-miR-2390 | -0.39 |
| TMEFF2 | ENST00000392314.1 | transmembrane protein with EGF-like and two follistatin-like domains 2 | 179 | 1 | 0 | 1 | 0 | 2 | bta-miR-2390 | -0.39 |
| SLC9A3R1 | ENST00000262613.5 | solute carrier family 9, subfamily A (NHE3, cation proton antiporter 3), member 3 regulator 1 | 2939 | 1 | 1 | 0 | 0 | 1 | bta-miR-2345 | -0.39 |
| TRIM42 | ENST00000286349.3 | tripartite motif containing 42 | 5 | 1 | 1 | 0 | 0 | 0 | bta-miR-2345 | -0.39 |
| FAM168B | ENST00000409185.1 | family with sequence similarity 168, member B | 122 | 1 | 1 | 0 | 0 | 1 | bta-miR-2390 | -0.38 |
| ACBD7 | ENST00000356189.5 | acyl-CoA binding domain containing 7 | 34 | 2 | 1 | 0 | 1 | 0 | bta-miR-2345 | -0.38 |
| CAGE1 | ENST00000379918.4 | cancer antigen 1 | 5 | 1 | 1 | 0 | 0 | 0 | bta-miR-2345 | -0.38 |
| C5orf20 | ENST00000503143.2 | chromosome 5 open reading frame 20 | 5 | 1 | 1 | 0 | 0 | 0 | bta-miR-2390 | -0.38 |
| TIAF1 | ENST00000408971.2 | TGFB1-induced anti-apoptotic factor 1 | 152 | 1 | 1 | 0 | 0 | 1 | bta-miR-2390 | -0.37 |
| HNRNPUL1 | ENST00000352456.3 | heterogeneous nuclear ribonucleoprotein U-like 1 | 11882 | 2 | 1 | 1 | 0 | 1 | bta-miR-2390 | -0.37 |
| SPATA2L | ENST00000289805.5 | spermatogenesis associated 2-like | 10 | 1 | 1 | 0 | 0 | 0 | bta-miR-2390 | -0.37 |
| CCDC102B | ENST00000319445.6 | coiled-coil domain containing 102B | 5 | 1 | 1 | 0 | 0 | 0 | bta-miR-2345 | -0.37 |
| RBP5 | ENST00000266560.3 | retinol binding protein 5, cellular | 256 | 1 | 1 | 0 | 0 | 0 | bta-miR-2345 | -0.37 |
| GYPA | ENST00000360771.4 | glycophorin A (MNS blood group) | 5 | 2 | 0 | 1 | 1 | 0 | bta-miR-2345 | -0.37 |
| CTXN2 | ENST00000417307.2 | cortexin 2 | 5 | 1 | 1 | 0 | 0 | 1 | bta-miR-2390 | -0.37 |
| PLEKHS1 | ENST00000354462.3 | pleckstrin homology domain containing, family S member 1 | 7 | 4 | 1 | 1 | 2 | 0 | bta-miR-2390 | -0.37 |
| CNOT4 | ENST00000428680.2 | CCR4-NOT transcription complex, subunit 4 | 30 | 2 | 1 | 0 | 1 | 2 | bta-miR-2345 | -0.37 |
| KLF7 | ENST00000309446.6 | Kruppel-like factor 7 (ubiquitous) | 737 | 2 | 1 | 1 | 0 | 0 | bta-miR-2390 | -0.36 |
| WDR20 | ENST00000335263.5 | WD repeat domain 20 | 288 | 1 | 1 | 0 | 0 | 0 | bta-miR-2345 | -0.36 |
| NKAIN2 | ENST00000368417.1 | Na+/K+ transporting ATPase interacting 2 | 34 | 1 | 1 | 0 | 0 | 0 | bta-miR-2390 | -0.36 |
| USP10 | ENST00000219473.7 | ubiquitin specific peptidase 10 | 66 | 1 | 1 | 0 | 0 | 0 | bta-miR-2345 | -0.36 |
| ZNF764 | ENST00000252797.2 | zinc finger protein 764 | 59 | 1 | 1 | 0 | 0 | 0 | bta-miR-2345 | -0.36 |
| CCNE1 | ENST00000262643.3 | cyclin E1 | 954 | 1 | 1 | 0 | 0 | 0 | bta-miR-2390 | -0.36 |
| HOXC4 | ENST00000609810.1 | homeobox C4 | 508 | 1 | 1 | 0 | 0 | 0 | bta-miR-2345 | -0.36 |
| HOXC4 | ENST00000430889.2 | Homeobox protein Hox-C4 | 508 | 1 | 1 | 0 | 0 | 0 | bta-miR-2345 | -0.36 |
| KCNV2 | ENST00000382082.3 | potassium channel, subfamily V, member 2 | 5 | 1 | 1 | 0 | 0 | 0 | bta-miR-2345 | -0.36 |
| PAFAH1B1 | ENST00000397195.5 | platelet-activating factor acetylhydrolase 1b, regulatory subunit 1 (45kDa) | 1570 | 2 | 1 | 1 | 0 | 1 | bta-miR-2390 | -0.36 |
| CCL18 | ENST00000004921.3 | chemokine (C-C motif) ligand 18 (pulmonary and activation-regulated) | 5 | 1 | 1 | 0 | 0 | 0 | bta-miR-2345 | -0.36 |
| CPNE4 | ENST00000512055.1 | copine IV | 5 | 2 | 1 | 0 | 1 | 0 | bta-miR-2345 | -0.36 |
| VAPB | ENST00000475243.1 | VAMP (vesicle-associated membrane protein)-associated protein B and C | 391 | 2 | 2 | 0 | 0 | 1 | bta-miR-2390 | -0.35 |
| MDH1B | ENST00000374412.3 | malate dehydrogenase 1B, NAD (soluble) | 20 | 1 | 1 | 0 | 0 | 0 | bta-miR-2390 | -0.35 |
| RP11-105C20.2 | ENST00000564533.1 | Uncharacterized protein | 5 | 1 | 0 | 0 | 1 | 1 | bta-miR-2345 | -0.35 |
| MYB | ENST00000367814.4 | v-myb avian myeloblastosis viral oncogene homolog | 39 | 2 | 2 | 0 | 0 | 1 | bta-miR-2345 | -0.35 |
| DMC1 | ENST00000216024.2 | DNA meiotic recombinase 1 | 9 | 1 | 1 | 0 | 0 | 0 | bta-miR-2345 | -0.34 |
| GABBR2 | ENST00000259455.2 | gamma-aminobutyric acid (GABA) B receptor, 2 | 214 | 1 | 1 | 0 | 0 | 1 | bta-miR-2390 | -0.34 |
| HMGA2 | ENST00000403681.2 | high mobility group AT-hook 2 | 8021 | 2 | 1 | 0 | 1 | 1 | bta-miR-2390 | -0.34 |
| CYTH4 | ENST00000248901.6 | cytohesin 4 | 5 | 2 | 1 | 1 | 0 | 0 | bta-miR-2390 | -0.34 |
| ZFHX3 | ENST00000268489.5 | zinc finger homeobox 3 | 57 | 1 | 1 | 0 | 0 | 2 | bta-miR-2390 | -0.34 |
| PARPBP | ENST00000378128.3 | PARP1 binding protein | 456 | 2 | 1 | 0 | 1 | 0 | bta-miR-2390 | -0.34 |
| ZNF662 | ENST00000541208.1 | zinc finger protein 662 | 11 | 1 | 1 | 0 | 0 | 0 | bta-miR-2390 | -0.34 |
| FAM43B | ENST00000332947.4 | family with sequence similarity 43, member B | 17 | 1 | 0 | 1 | 0 | 0 | bta-miR-2390 | -0.34 |
| TLX1 | ENST00000467928.2 | T-cell leukemia homeobox 1 | 47 | 2 | 1 | 1 | 0 | 0 | bta-miR-2390 | -0.33 |
| RP11-67H2.1 | ENST00000521500.2 | Uncharacterized protein | 5 | 1 | 0 | 0 | 1 | 2 | bta-miR-2345 | -0.33 |
| INHBA | ENST00000242208.4 | inhibin, beta A | 1260 | 3 | 3 | 0 | 0 | 1 | bta-miR-2390 | -0.33 |
| A1CF | ENST00000374001.2 | APOBEC1 complementation factor | 80 | 1 | 1 | 0 | 0 | 0 | bta-miR-2345 | -0.33 |
| OR6C65 | ENST00000379665.2 | olfactory receptor, family 6, subfamily C, member 65 | 5 | 1 | 0 | 0 | 1 | 0 | bta-miR-2345 | -0.33 |
| TMEM239 | ENST00000361033.1 | transmembrane protein 239 | 5 | 1 | 1 | 0 | 0 | 0 | bta-miR-2345 | -0.33 |
| MLKL | ENST00000306247.7 | mixed lineage kinase domain-like | 44 | 1 | 1 | 0 | 0 | 0 | bta-miR-2345 | -0.33 |
| RP11-169F17.1 | ENST00000581862.1 | Protein LOC400655 | 5 | 2 | 0 | 0 | 2 | 0 | bta-miR-2390 | -0.33 |
| SGPP2 | ENST00000321276.7 | sphingosine-1-phosphate phosphatase 2 | 182 | 3 | 2 | 0 | 1 | 0 | bta-miR-2390 | -0.33 |
| B3GNT5 | ENST00000326505.3 | UDP-GlcNAc:betaGal beta-1,3-N-acetylglucosaminyltransferase 5 | 57 | 2 | 1 | 0 | 1 | 1 | bta-miR-2390 | -0.33 |
| CALM1 | ENST00000356978.4 | calmodulin 1 (phosphorylase kinase, delta) | 4046 | 1 | 1 | 0 | 0 | 0 | bta-miR-2390 | -0.32 |
| RP11-147C23.1 | ENST00000413825.2 | Uncharacterized protein | 5 | 1 | 0 | 0 | 1 | 0 | bta-miR-2390 | -0.32 |
| EPAS1 | ENST00000263734.3 | endothelial PAS domain protein 1 | 3689 | 2 | 2 | 0 | 0 | 0 | bta-miR-2345 | -0.32 |
| TMEM126A | ENST00000304511.2 | transmembrane protein 126A | 18 | 1 | 0 | 0 | 1 | 0 | bta-miR-2345 | -0.32 |
| C16orf93 | ENST00000545825.1 | chromosome 16 open reading frame 93 | 38 | 1 | 0 | 1 | 0 | 0 | bta-miR-2345 | -0.32 |
| TMEM229A | ENST00000455783.1 | transmembrane protein 229A | 5 | 1 | 1 | 0 | 0 | 1 | bta-miR-2345 | -0.32 |
| AP1S2 | ENST00000329235.2 | adaptor-related protein complex 1, sigma 2 subunit | 357 | 2 | 0 | 1 | 1 | 3 | bta-miR-2390 | -0.31 |
| MAPK6 | ENST00000261845.5 | mitogen-activated protein kinase 6 | 24 | 1 | 1 | 0 | 0 | 0 | bta-miR-2345 | -0.31 |
| VSNL1 | ENST00000406397.1 | visinin-like 1 | 89 | 2 | 0 | 2 | 0 | 1 | bta-miR-2345 | -0.31 |
| UGT2A1 | ENST00000503640.1 | UDP glucuronosyltransferase 2 family, polypeptide A1, complex locus | 5 | 3 | 0 | 0 | 3 | 0 | bta-miR-2345 | -0.31 |
| AMELY | ENST00000215479.5 | amelogenin, Y-linked | 5 | 1 | 0 | 0 | 1 | 1 | bta-miR-2345 | -0.31 |
| HAS2 | ENST00000303924.4 | hyaluronan synthase 2 | 398 | 2 | 2 | 0 | 0 | 0 | bta-miR-2345 | -0.31 |
| TRIM64B | ENST00000329862.6 | tripartite motif containing 64B | 5 | 2 | 1 | 0 | 1 | 1 | bta-miR-2345 | -0.31 |
| UGT2A2 | ENST00000457664.2 | UDP glucuronosyltransferase 2 family, polypeptide A2 | 5 | 3 | 0 | 0 | 3 | 0 | bta-miR-2345 | -0.31 |
| FOXP1 | ENST00000318789.4 | forkhead box P1 | 264 | 1 | 1 | 0 | 0 | 1 | bta-miR-2390 | -0.31 |
| AMELX | ENST00000380714.3 | amelogenin, X-linked | 5 | 1 | 0 | 0 | 1 | 1 | bta-miR-2345 | -0.31 |
| LINC00632 | ENST00000370535.3 | long intergenic non-protein coding RNA 632 | 5 | 1 | 0 | 1 | 0 | 0 | bta-miR-2390 | -0.31 |
| MEIS2 | ENST00000397624.3 | Meis homeobox 2 | 63 | 3 | 1 | 0 | 2 | 0 | bta-miR-2390 | -0.31 |
| TRIQK | ENST00000521988.1 | triple QxxK/R motif containing | 210 | 1 | 1 | 0 | 0 | 0 | bta-miR-2345 | -0.3 |
| BRD4 | ENST00000263377.2 | bromodomain containing 4 | 849 | 2 | 2 | 0 | 0 | 1 | bta-miR-2390 | -0.3 |
| MICU1 | ENST00000361114.5 | mitochondrial calcium uptake 1 | 325 | 1 | 1 | 0 | 0 | 0 | bta-miR-2390 | -0.3 |
| KCNN3 | ENST00000271915.4 | potassium intermediate/small conductance calcium-activated channel, subfamily N, member 3 | 5 | 2 | 0 | 1 | 1 | 1 | bta-miR-2345 | -0.3 |
| MAFA | ENST00000333480.2 | v-maf avian musculoaponeurotic fibrosarcoma oncogene homolog A | 7 | 1 | 1 | 0 | 0 | 0 | bta-miR-2390 | -0.3 |
| PTPRD | ENST00000381196.4 | protein tyrosine phosphatase, receptor type, D | 101 | 4 | 2 | 1 | 1 | 1 | bta-miR-2390 | -0.3 |
| SH3BGRL | ENST00000373212.5 | SH3 domain binding glutamic acid-rich protein like | 2034 | 1 | 1 | 0 | 0 | 0 | bta-miR-2390 | -0.3 |
| ELAVL3 | ENST00000359227.3 | ELAV like neuron-specific RNA binding protein 3 | 5 | 3 | 0 | 0 | 3 | 0 | bta-miR-2390 | -0.3 |
| IGF2 | ENST00000381395.1 | insulin-like growth factor 2 (somatomedin A) | 1371 | 1 | 0 | 1 | 0 | 1 | bta-miR-2390 | -0.3 |
| HFE | ENST00000357618.5 | hemochromatosis | 563 | 1 | 1 | 0 | 0 | 0 | bta-miR-2345 | -0.3 |
| IRF2BPL | ENST00000238647.3 | interferon regulatory factor 2 binding protein-like | 2145 | 1 | 1 | 0 | 0 | 0 | bta-miR-2390 | -0.3 |
| SPECC1 | ENST00000395530.2 | sperm antigen with calponin homology and coiled-coil domains 1 | 128 | 4 | 1 | 0 | 3 | 0 | bta-miR-2345 | -0.3 |
| LATS2 | ENST00000382592.4 | large tumor suppressor kinase 2 | 464 | 2 | 1 | 1 | 0 | 0 | bta-miR-2345 | -0.3 |
| C1orf213 | ENST00000335648.3 | chromosome 1 open reading frame 213 | 49 | 1 | 1 | 0 | 0 | 0 | bta-miR-2390 | -0.3 |
| PPM1D | ENST00000305921.3 | protein phosphatase, Mg2+/Mn2+ dependent, 1D | 896 | 1 | 1 | 0 | 0 | 1 | bta-miR-2345 | -0.3 |
| DNMT3A | ENST00000380746.4 | DNA (cytosine-5-)-methyltransferase 3 alpha | 221 | 2 | 0 | 1 | 1 | 2 | bta-miR-2390 | -0.3 |
| PLCXD3 | ENST00000377801.3 | phosphatidylinositol-specific phospholipase C, X domain containing 3 | 81 | 2 | 1 | 0 | 1 | 2 | bta-miR-2390 | -0.3 |
| ARHGEF9 | ENST00000253401.6 | Cdc42 guanine nucleotide exchange factor (GEF) 9 | 13 | 3 | 1 | 0 | 2 | 0 | bta-miR-2345 | -0.3 |
| CXCL14 | ENST00000337225.5 | chemokine (C-X-C motif) ligand 14 | 5 | 2 | 0 | 0 | 2 | 0 | bta-miR-2345 | -0.3 |
| IFI16 | ENST00000368131.4 | interferon, gamma-inducible protein 16 | 25 | 1 | 0 | 1 | 0 | 0 | bta-miR-2390 | -0.29 |
| NAV3 | ENST00000228327.6 | neuron navigator 3 | 277 | 1 | 1 | 0 | 0 | 0 | bta-miR-2390 | -0.29 |
| RAB27A | ENST00000396307.2 | RAB27A, member RAS oncogene family | 181 | 3 | 0 | 1 | 2 | 0 | bta-miR-2345 | -0.29 |
| BCL7B | ENST00000223368.2 | B-cell CLL/lymphoma 7B | 4037 | 1 | 1 | 0 | 0 | 0 | bta-miR-2390 | -0.29 |
| ARPP21 | ENST00000428373.1 | cAMP-regulated phosphoprotein, 21kDa | 5 | 2 | 0 | 1 | 1 | 0 | bta-miR-2345 | -0.29 |
| GORASP1 | ENST00000319283.3 | golgi reassembly stacking protein 1, 65kDa | 102 | 1 | 0 | 0 | 1 | 0 | bta-miR-2345 | -0.29 |
| KCNIP4 | ENST00000382148.3 | Kv channel interacting protein 4 | 5 | 1 | 1 | 0 | 0 | 0 | bta-miR-2390 | -0.29 |
| TMEM170A | ENST00000357613.4 | transmembrane protein 170A | 70 | 1 | 1 | 0 | 0 | 1 | bta-miR-2345 | -0.29 |
| ARIH1 | ENST00000379887.4 | ariadne RBR E3 ubiquitin protein ligase 1 | 671 | 2 | 1 | 0 | 1 | 0 | bta-miR-2390 | -0.29 |
| ZFX | ENST00000539115.1 | zinc finger protein, X-linked | 583 | 1 | 0 | 1 | 0 | 1 | bta-miR-2345 | -0.29 |
| PSMB6 | ENST00000270586.3 | proteasome (prosome, macropain) subunit, beta type, 6 | 23086 | 1 | 0 | 0 | 1 | 0 | bta-miR-2390 | -0.28 |
| ITGAM | ENST00000544665.3 | integrin, alpha M (complement component 3 receptor 3 subunit) | 10 | 2 | 1 | 0 | 1 | 0 | bta-miR-2390 | -0.28 |
| SSBP4 | ENST00000348495.6 | single stranded DNA binding protein 4 | 510 | 1 | 0 | 1 | 0 | 0 | bta-miR-2390 | -0.28 |
| PPP1R1C | ENST00000409137.3 | protein phosphatase 1, regulatory (inhibitor) subunit 1C | 5 | 2 | 0 | 0 | 2 | 0 | bta-miR-2345 | -0.28 |
| MZT1 | ENST00000377818.3 | mitotic spindle organizing protein 1 | 157 | 1 | 0 | 1 | 0 | 0 | bta-miR-2345 | -0.28 |
| AMZ1 | ENST00000312371.4 | archaelysin family metallopeptidase 1 | 5 | 1 | 0 | 1 | 0 | 0 | bta-miR-2345 | -0.28 |
| DTHD1 | ENST00000456874.2 | death domain containing 1 | 5 | 2 | 2 | 0 | 0 | 0 | bta-miR-2345 | -0.28 |
| RAN | ENST00000543796.1 | RAN, member RAS oncogene family | 1418 | 2 | 2 | 0 | 0 | 0 | bta-miR-2345 | -0.28 |
| PSTK | ENST00000405485.1 | phosphoseryl-tRNA kinase | 530 | 1 | 1 | 0 | 0 | 0 | bta-miR-2390 | -0.28 |
| PITX2 | ENST00000306732.3 | paired-like homeodomain 2 | 75 | 1 | 1 | 0 | 0 | 0 | bta-miR-2345 | -0.28 |
| TLE1 | ENST00000376499.3 | transducin-like enhancer of split 1 (E(sp1) homolog, Drosophila) | 1996 | 2 | 0 | 0 | 2 | 0 | bta-miR-2390 | -0.28 |
| BMP4 | ENST00000245451.4 | bone morphogenetic protein 4 | 24 | 1 | 0 | 1 | 0 | 0 | bta-miR-2390 | -0.28 |
| TTLL9 | ENST00000375921.2 | tubulin tyrosine ligase-like family, member 9 | 5 | 1 | 1 | 0 | 0 | 0 | bta-miR-2345 | -0.27 |
| RASGRP1 | ENST00000310803.5 | RAS guanyl releasing protein 1 (calcium and DAG-regulated) | 7 | 4 | 0 | 0 | 4 | 2 | bta-miR-2345 | -0.27 |
| DLGAP4 | ENST00000373913.3 | discs, large (Drosophila) homolog-associated protein 4 | 1188 | 1 | 1 | 0 | 0 | 0 | bta-miR-2390 | -0.27 |
| SLITRK3 | ENST00000475390.1 | SLIT and NTRK-like family, member 3 | 5 | 1 | 1 | 0 | 0 | 1 | bta-miR-2390 | -0.27 |
| PHLDA2 | ENST00000314222.4 | pleckstrin homology-like domain, family A, member 2 | 755 | 1 | 0 | 1 | 0 | 0 | bta-miR-2345 | -0.27 |
| ZNF616 | ENST00000330123.5 | zinc finger protein 616 | 45 | 1 | 1 | 0 | 0 | 0 | bta-miR-2390 | -0.27 |
| DOC2A | ENST00000350119.4 | double C2-like domains, alpha | 10 | 1 | 0 | 1 | 0 | 1 | bta-miR-2390 | -0.27 |
| ZNF385D | ENST00000281523.2 | zinc finger protein 385D | 5 | 1 | 1 | 0 | 0 | 0 | bta-miR-2390 | -0.27 |
| MS4A14 | ENST00000395001.1 | membrane-spanning 4-domains, subfamily A, member 14 | 5 | 1 | 0 | 1 | 0 | 1 | bta-miR-2390 | -0.27 |
| LIN28B | ENST00000345080.4 | lin-28 homolog B (C. elegans) | 118 | 3 | 1 | 1 | 1 | 0 | bta-miR-2390 | -0.27 |
| UBQLN2 | ENST00000338222.5 | ubiquilin 2 | 70 | 1 | 1 | 0 | 0 | 0 | bta-miR-2390 | -0.27 |
| RABEP1 | ENST00000262477.6 | rabaptin, RAB GTPase binding effector protein 1 | 1163 | 1 | 1 | 0 | 0 | 0 | bta-miR-2345 | -0.27 |
| CHN1 | ENST00000409900.3 | chimerin 1 | 344 | 1 | 1 | 0 | 0 | 0 | bta-miR-2390 | -0.27 |
| KLK6 | ENST00000376851.3 | kallikrein-related peptidase 6 | 5 | 1 | 0 | 1 | 0 | 1 | bta-miR-2390 | -0.27 |
| MAPRE2 | ENST00000436190.2 | microtubule-associated protein, RP/EB family, member 2 | 361 | 3 | 0 | 2 | 1 | 0 | bta-miR-2390 | -0.27 |
| BRINP2 | ENST00000361539.4 | bone morphogenetic protein/retinoic acid inducible neural-specific 2 | 5 | 1 | 1 | 0 | 0 | 0 | bta-miR-2390 | -0.27 |
| IQUB | ENST00000466202.1 | IQ motif and ubiquitin domain containing | 5 | 1 | 0 | 1 | 0 | 0 | bta-miR-2345 | -0.27 |
| CTD-2228K2.5 | ENST00000342584.3 | Uncharacterized protein | 199 | 1 | 0 | 0 | 1 | 0 | bta-miR-2345 | -0.27 |
| MFSD6 | ENST00000392328.1 | major facilitator superfamily domain containing 6 | 47 | 3 | 0 | 0 | 3 | 0 | bta-miR-2390 | -0.27 |
| LAMP3 | ENST00000265598.3 | lysosomal-associated membrane protein 3 | 11 | 1 | 1 | 0 | 0 | 0 | bta-miR-2390 | -0.27 |
| MTF1 | ENST00000373036.4 | metal-regulatory transcription factor 1 | 98 | 3* | 0 | 0 | 2 | 1 | bta-miR-2390 | -0.27 |
| PBX3 | ENST00000342287.5 | pre-B-cell leukemia homeobox 3 | 1142 | 2 | 1 | 1 | 0 | 0 | bta-miR-2345 | -0.26 |
| RBFOX2 | ENST00000449924.2 | RNA binding protein, fox-1 homolog (C. elegans) 2 | 262 | 1 | 0 | 0 | 1 | 0 | bta-miR-2390 | -0.26 |
| AL117190.3 | ENST00000599197.1 | Esophagus cancer-related gene-2 interaction susceptibility protein; Uncharacterized protein | 30 | 1 | 1 | 0 | 0 | 1 | bta-miR-2390 | -0.26 |
| SMIM11 | ENST00000399299.1 | small integral membrane protein 11 | 108 | 1 | 0 | 0 | 1 | 0 | bta-miR-2345 | -0.26 |
| TPH1 | ENST00000250018.2 | tryptophan hydroxylase 1 | 12 | 2 | 0 | 1 | 1 | 0 | bta-miR-2345 | -0.26 |
| SLC16A14 | ENST00000295190.4 | solute carrier family 16, member 14 | 34 | 2 | 1 | 1 | 0 | 0 | bta-miR-2345 | -0.26 |
| SLC35F5 | ENST00000245680.2 | solute carrier family 35, member F5 | 136 | 1 | 1 | 0 | 0 | 0 | bta-miR-2390 | -0.26 |
| DCX | ENST00000356915.2 | doublecortin | 5 | 2 | 0 | 1 | 1 | 1 | bta-miR-2345 | -0.26 |
| CDK2 | ENST00000266970.4 | cyclin-dependent kinase 2 | 6789 | 1 | 1 | 0 | 0 | 0 | bta-miR-2345 | -0.26 |
| PLEKHH3 | ENST00000591022.1 | pleckstrin homology domain containing, family H (with MyTH4 domain) member 3 | 5 | 1 | 0 | 0 | 1 | 0 | bta-miR-2390 | -0.26 |
| PROX2 | ENST00000556084.2 | prospero homeobox 2 | 5 | 3 | 0 | 1 | 2 | 3 | bta-miR-2345 | -0.26 |
| FAM120A | ENST00000333936.5 | family with sequence similarity 120A | 57 | 2 | 1 | 0 | 1 | 1 | bta-miR-2345 | -0.26 |
| ZBTB4 | ENST00000380599.4 | zinc finger and BTB domain containing 4 | 1039 | 2 | 2 | 0 | 0 | 0 | bta-miR-2345 | -0.26 |
| KALRN | ENST00000393496.1 | kalirin, RhoGEF kinase | 83 | 1 | 0 | 0 | 1 | 0 | bta-miR-2390 | -0.26 |
| JPH4 | ENST00000356300.4 | junctophilin 4 | 5 | 3 | 1 | 2 | 0 | 0 | bta-miR-2345 | -0.26 |
| TMED7-TICAM2 | ENST00000333314.3 | TMED7-TICAM2 readthrough | 202 | 2 | 1 | 1 | 0 | 0 | bta-miR-2390 | -0.26 |
| SDK2 | ENST00000392650.3 | sidekick cell adhesion molecule 2 | 8 | 1 | 0 | 0 | 1 | 0 | bta-miR-2390 | -0.26 |
| CLLU1 | ENST00000378485.1 | chronic lymphocytic leukemia up-regulated 1 | 5 | 1 | 0 | 1 | 0 | 0 | bta-miR-2345 | -0.26 |
| PROCR | ENST00000216968.4 | protein C receptor, endothelial | 213 | 1 | 0 | 1 | 0 | 0 | bta-miR-2390 | -0.26 |
| ACAA1 | ENST00000333167.8 | acetyl-CoA acyltransferase 1 | 3539 | 1 | 1 | 0 | 0 | 0 | bta-miR-2345 | -0.26 |
| AC002451.1 | ENST00000601424.1 | Protein LOC100996577 | 5 | 2 | 0 | 0 | 2 | 0 | bta-miR-2345 | -0.26 |
| SCML1 | ENST00000380045.3 | sex comb on midleg-like 1 (Drosophila) | 722 | 2 | 0 | 1 | 1 | 0 | bta-miR-2345 | -0.26 |
| CAST | ENST00000395812.2 | calpastatin | 4674 | 1 | 1 | 0 | 0 | 0 | bta-miR-2345 | -0.26 |
| CD82 | ENST00000227155.4 | CD82 molecule | 1117 | 1 | 0 | 1 | 0 | 0 | bta-miR-2390 | -0.26 |
| MURC | ENST00000307584.5 | muscle-related coiled-coil protein | 75 | 2 | 1 | 0 | 1 | 0 | bta-miR-2390 | -0.25 |
| NMT2 | ENST00000378165.4 | N-myristoyltransferase 2 | 318 | 1 | 1 | 0 | 0 | 0 | bta-miR-2345 | -0.25 |
| IL1A | ENST00000263339.3 | interleukin 1, alpha | 5 | 2 | 0 | 1 | 1 | 0 | bta-miR-2345 | -0.25 |
| CHST4 | ENST00000338482.5 | carbohydrate (N-acetylglucosamine 6-O) sulfotransferase 4 | 15 | 1 | 0 | 0 | 1 | 0 | bta-miR-2390 | -0.25 |
| PHF17 | ENST00000226319.6 | PHD finger protein 17 | 1393 | 5 | 2 | 0 | 3 | 1 | bta-miR-2390 | -0.25 |
| ZFP1 | ENST00000464850.1 | ZFP1 zinc finger protein | 32 | 3 | 1 | 1 | 1 | 0 | bta-miR-2390 | -0.25 |
| MAN1C1 | ENST00000374332.4 | mannosidase, alpha, class 1C, member 1 | 60 | 3 | 1 | 1 | 1 | 0 | bta-miR-2390 | -0.25 |
| KCNIP3 | ENST00000295225.5 | Kv channel interacting protein 3, calsenilin | 555 | 1 | 1 | 0 | 0 | 0 | bta-miR-2390 | -0.25 |
| TMEM40 | ENST00000314124.7 | transmembrane protein 40 | 5 | 1 | 0 | 0 | 1 | 1 | bta-miR-2390 | -0.25 |
| MYO3B | ENST00000334231.6 | myosin IIIB | 5 | 4 | 1 | 0 | 3 | 0 | bta-miR-2345 | -0.25 |
| TNFSF10 | ENST00000420541.2 | tumor necrosis factor (ligand) superfamily, member 10 | 28 | 1 | 0 | 0 | 1 | 1 | bta-miR-2390 | -0.25 |
| C1orf63 | ENST00000243189.7 | chromosome 1 open reading frame 63 | 914 | 1 | 0 | 0 | 1 | 0 | bta-miR-2390 | -0.25 |
| LYRM2 | ENST00000520318.1 | LYR motif containing 2 | 305 | 3 | 1 | 2 | 0 | 1 | bta-miR-2345 | -0.25 |
| WNT2B | ENST00000369686.5 | wingless-type MMTV integration site family, member 2B | 5 | 3 | 1 | 0 | 2 | 2 | bta-miR-2390 | -0.25 |
| RSU1 | ENST00000377921.3 | Ras suppressor protein 1 | 10596 | 1 | 0 | 1 | 0 | 1 | bta-miR-2345 | -0.25 |
| NRAS | ENST00000369535.4 | neuroblastoma RAS viral (v-ras) oncogene homolog | 551 | 1 | 1 | 0 | 0 | 0 | bta-miR-2345 | -0.25 |
| TCTEX1D1 | ENST00000282670.2 | Tctex1 domain containing 1 | 20 | 2 | 0 | 0 | 2 | 2 | bta-miR-2345 | -0.24 |
| LCOR | ENST00000371103.3 | ligand dependent nuclear receptor corepressor | 94 | 4 | 0 | 2 | 2 | 0 | bta-miR-2345 | -0.24 |
| SPIN3 | ENST00000374919.3 | spindlin family, member 3 | 12 | 1 | 1 | 0 | 0 | 1 | bta-miR-2345 | -0.24 |
| LAMP5 | ENST00000246070.2 | lysosomal-associated membrane protein family, member 5 | 26 | 1 | 1 | 0 | 0 | 0 | bta-miR-2345 | -0.24 |
| NEUROD1 | ENST00000295108.3 | neuronal differentiation 1 | 5 | 2 | 1 | 0 | 1 | 0 | bta-miR-2390 | -0.24 |
| TPT1 | ENST00000379056.1 | tumor protein, translationally-controlled 1 | 2856 | 1 | 0 | 1 | 0 | 0 | bta-miR-2390 | -0.24 |
| OR5H14 | ENST00000437310.1 | olfactory receptor, family 5, subfamily H, member 14 | 5 | 1 | 0 | 0 | 1 | 0 | bta-miR-2390 | -0.24 |
| C1orf174 | ENST00000361605.3 | chromosome 1 open reading frame 174 | 10 | 1 | 0 | 0 | 1 | 0 | bta-miR-2345 | -0.24 |
| FOXO1 | ENST00000379561.5 | forkhead box O1 | 480 | 1 | 1 | 0 | 0 | 2 | bta-miR-2390 | -0.24 |
| HNRNPA1 | ENST00000546500.1 | heterogeneous nuclear ribonucleoprotein A1 | 2079 | 2 | 0 | 1 | 1 | 0 | bta-miR-2345 | -0.24 |
| MOB4 | ENST00000233892.4 | MOB family member 4, phocein | 123 | 2 | 1 | 0 | 1 | 0 | bta-miR-2345 | -0.24 |
| ZKSCAN3 | ENST00000377255.3 | zinc finger with KRAB and SCAN domains 3 | 63 | 3 | 0 | 0 | 3 | 0 | bta-miR-2390 | -0.24 |
| ELOVL6 | ENST00000394607.3 | ELOVL fatty acid elongase 6 | 901 | 2 | 1 | 1 | 0 | 2 | bta-miR-2390 | -0.24 |
| TNR | ENST00000367674.2 | tenascin R | 5 | 2 | 0 | 0 | 2 | 0 | bta-miR-2345 | -0.24 |
| AC090186.1 | ENST00000415643.1 | Uncharacterized protein | 5 | 1 | 0 | 0 | 1 | 0 | bta-miR-2345 | -0.24 |
| GPR6 | ENST00000275169.3 | G protein-coupled receptor 6 | 5 | 1 | 0 | 0 | 1 | 1 | bta-miR-2390 | -0.24 |
| FCGR2A | ENST00000271450.6 | Fc fragment of IgG, low affinity IIa, receptor (CD32) | 5 | 1 | 0 | 0 | 1 | 0 | bta-miR-2345 | -0.24 |
| KLF5 | ENST00000377687.4 | Kruppel-like factor 5 (intestinal) | 161 | 2 | 0 | 1 | 1 | 0 | bta-miR-2345 | -0.24 |
| TMPRSS4 | ENST00000534111.1 | transmembrane protease, serine 4 | 5 | 1 | 1 | 0 | 0 | 0 | bta-miR-2345 | -0.24 |
| NRXN3 | ENST00000281127.7 | neurexin 3 | 14 | 2 | 1 | 1 | 0 | 1 | bta-miR-2390 | -0.24 |
| TLR9 | ENST00000360658.2 | toll-like receptor 9 | 5 | 1 | 1 | 0 | 0 | 0 | bta-miR-2345 | -0.24 |
| ACOX1 | ENST00000293217.5 | acyl-CoA oxidase 1, palmitoyl | 287 | 2 | 1 | 1 | 0 | 0 | bta-miR-2390 | -0.24 |
| VSIG4 | ENST00000455586.2 | V-set and immunoglobulin domain containing 4 | 5 | 1 | 1 | 0 | 0 | 0 | bta-miR-2345 | -0.24 |
| NHS | ENST00000380060.3 | Nance-Horan syndrome (congenital cataracts and dental anomalies) | 72 | 2 | 1 | 0 | 1 | 1 | bta-miR-2390 | -0.24 |
| PRMT3 | ENST00000331079.6 | protein arginine methyltransferase 3 | 120 | 2 | 2 | 0 | 0 | 1 | bta-miR-2390 | -0.24 |
| HIST1H2AI | ENST00000358739.3 | histone cluster 1, H2ai | 11 | 1 | 1 | 0 | 0 | 0 | bta-miR-2390 | -0.24 |
| KCNT2 | ENST00000367433.5 | potassium channel, subfamily T, member 2 | 5 | 2 | 0 | 0 | 2 | 2 | bta-miR-2345 | -0.24 |
| FGF7 | ENST00000267843.4 | fibroblast growth factor 7 | 5 | 2 | 0 | 0 | 2 | 2 | bta-miR-2390 | -0.24 |
| TBX15 | ENST00000207157.3 | T-box 15 | 14 | 3 | 0 | 0 | 3 | 0 | bta-miR-2390 | -0.24 |
| GJD3 | ENST00000578689.1 | gap junction protein, delta 3, 31.9kDa | 0 | 1 | 1 | 0 | 0 | 0 | bta-miR-2390 | -0.24 |
| MRGPRX2 | ENST00000329773.2 | MAS-related GPR, member X2 | 5 | 1 | 0 | 1 | 0 | 0 | bta-miR-2345 | -0.24 |
| LCMT2 | ENST00000567039.1 | leucine carboxyl methyltransferase 2 | 274 | 1 | 0 | 0 | 1 | 2 | bta-miR-2345 | -0.24 |
| TMEM106C | ENST00000429772.2 | transmembrane protein 106C | 27951 | 1 | 0 | 1 | 0 | 0 | bta-miR-2345 | -0.23 |
| MLEC | ENST00000228506.3 | malectin | 12084 | 1 | 0 | 1 | 0 | 0 | bta-miR-2390 | -0.23 |
| C7orf41 | ENST00000324453.8 | chromosome 7 open reading frame 41 | 235 | 2 | 1 | 1 | 0 | 1 | bta-miR-2345 | -0.23 |
| BRS3 | ENST00000370648.3 | bombesin-like receptor 3 | 5 | 1 | 0 | 1 | 0 | 0 | bta-miR-2345 | -0.23 |
| TMEM150C | ENST00000515780.2 | transmembrane protein 150C | 8 | 2 | 0 | 1 | 1 | 1 | bta-miR-2345 | -0.23 |
| TFEC | ENST00000265440.7 | transcription factor EC | 5 | 3 | 1 | 0 | 2 | 4 | bta-miR-2345 | -0.23 |
| RPL7L1 | ENST00000493763.1 | ribosomal protein L7-like 1 | 824 | 1 | 1 | 0 | 0 | 0 | bta-miR-2390 | -0.23 |
| TLR9 | ENST00000494383.1 | TLR9 | 5 | 1 | 1 | 0 | 0 | 0 | bta-miR-2345 | -0.23 |
| ZNF280B | ENST00000360412.2 | zinc finger protein 280B | 7 | 2 | 1 | 1 | 0 | 0 | bta-miR-2345 | -0.23 |
| KDELR3 | ENST00000216014.4 | KDEL (Lys-Asp-Glu-Leu) endoplasmic reticulum protein retention receptor 3 | 329 | 1 | 0 | 0 | 1 | 1 | bta-miR-2390 | -0.23 |
| GUCD1 | ENST00000447813.2 | guanylyl cyclase domain containing 1 | 2990 | 1 | 1 | 0 | 0 | 0 | bta-miR-2390 | -0.23 |
| LIN7C | ENST00000278193.2 | lin-7 homolog C (C. elegans) | 1701 | 1 | 0 | 1 | 0 | 1 | bta-miR-2390 | -0.23 |
| COPS7A | ENST00000534947.1 | COP9 signalosome subunit 7A | 124 | 1 | 1 | 0 | 0 | 0 | bta-miR-2390 | -0.23 |
| DNAJB5 | ENST00000545841.1 | DnaJ (Hsp40) homolog, subfamily B, member 5 | 379 | 1 | 1 | 0 | 0 | 0 | bta-miR-2345 | -0.23 |
| ST6GALNAC3 | ENST00000328299.3 | ST6 (alpha-N-acetyl-neuraminyl-2,3-beta-galactosyl-1,3)-N-acetylgalactosaminide alpha-2,6-sialyltransferase 3 | 41 | 2 | 0 | 1 | 1 | 3 | bta-miR-2390 | -0.23 |
| CSNK1A1 | ENST00000261798.5 | casein kinase 1, alpha 1 | 769 | 1 | 1 | 0 | 0 | 2 | bta-miR-2345 | -0.23 |
| ZBTB25 | ENST00000608382.1 | zinc finger and BTB domain containing 25 | 149 | 3 | 1 | 0 | 2 | 0 | bta-miR-2345 | -0.23 |
| HIST1H2AC | ENST00000602637.1 | histone cluster 1, H2ac | 1665 | 1 | 1 | 0 | 0 | 1 | bta-miR-2345 | -0.23 |
| NPFFR2 | ENST00000308744.6 | neuropeptide FF receptor 2 | 8 | 1 | 0 | 0 | 1 | 0 | bta-miR-2345 | -0.23 |
| C5orf51 | ENST00000381647.2 | chromosome 5 open reading frame 51 | 124 | 2 | 0 | 2 | 0 | 0 | bta-miR-2390 | -0.23 |
| FRMD4A | ENST00000358621.4 | FERM domain containing 4A | 192 | 1 | 1 | 0 | 0 | 2 | bta-miR-2345 | -0.23 |
| SRP19 | ENST00000282999.3 | signal recognition particle 19kDa | 243 | 1 | 0 | 0 | 1 | 0 | bta-miR-2390 | -0.23 |
| CHD1 | ENST00000284049.3 | chromodomain helicase DNA binding protein 1 | 35 | 1 | 1 | 0 | 0 | 0 | bta-miR-2390 | -0.23 |
| AADACL4 | ENST00000376221.1 | arylacetamide deacetylase-like 4 | 5 | 1 | 0 | 1 | 0 | 0 | bta-miR-2390 | -0.23 |
| TSEN15 | ENST00000533373.1 | TSEN15 tRNA splicing endonuclease subunit | 30 | 1 | 0 | 1 | 0 | 0 | bta-miR-2345 | -0.23 |
| NXPE4 | ENST00000424261.2 | neurexophilin and PC-esterase domain family, member 4 | 5 | 1 | 0 | 0 | 1 | 0 | bta-miR-2390 | -0.23 |
| PLGRKT | ENST00000223864.2 | plasminogen receptor, C-terminal lysine transmembrane protein | 821 | 2 | 0 | 1 | 1 | 2 | bta-miR-2345 | -0.22 |
| TMED8 | ENST00000216468.7 | transmembrane emp24 protein transport domain containing 8 | 53 | 2 | 1 | 0 | 1 | 0 | bta-miR-2390 | -0.22 |
| ARL13B | ENST00000535334.1 | ADP-ribosylation factor-like 13B | 23 | 1 | 0 | 1 | 0 | 1 | bta-miR-2390 | -0.22 |
| TXN | ENST00000374515.5 | thioredoxin | 7991 | 1 | 0 | 0 | 1 | 0 | bta-miR-2345 | -0.22 |
| C20orf85 | ENST00000371168.3 | chromosome 20 open reading frame 85 | 5 | 1 | 0 | 0 | 1 | 1 | bta-miR-2390 | -0.22 |
| GABRB3 | ENST00000311550.5 | gamma-aminobutyric acid (GABA) A receptor, beta 3 | 267 | 1 | 1 | 0 | 0 | 2 | bta-miR-2390 | -0.22 |
| C2orf83 | ENST00000409066.1 | chromosome 2 open reading frame 83 | 5 | 1 | 0 | 0 | 1 | 0 | bta-miR-2390 | -0.22 |
| MYH10 | ENST00000360416.3 | myosin, heavy chain 10, non-muscle | 134 | 2 | 0 | 0 | 2 | 0 | bta-miR-2345 | -0.22 |
| ZNF618 | ENST00000288466.7 | zinc finger protein 618 | 778 | 2 | 1 | 1 | 0 | 1 | bta-miR-2390 | -0.22 |
| SYNJ1 | ENST00000357345.3 | synaptojanin 1 | 143 | 2 | 2 | 0 | 0 | 0 | bta-miR-2390 | -0.22 |
| TGM5 | ENST00000220420.5 | transglutaminase 5 | 5 | 1 | 1 | 0 | 0 | 0 | bta-miR-2390 | -0.22 |
| OR11A1 | ENST00000377148.1 | olfactory receptor, family 11, subfamily A, member 1 | 5 | 2 | 0 | 0 | 2 | 0 | bta-miR-2390 | -0.22 |
| HDX | ENST00000297977.5 | highly divergent homeobox | 20 | 3 | 1 | 1 | 1 | 2 | bta-miR-2390 | -0.22 |
| CHEK2 | ENST00000382566.1 | checkpoint kinase 2 | 5 | 1 | 0 | 1 | 0 | 0 | bta-miR-2390 | -0.22 |
| RTN1 | ENST00000395090.1 | reticulon 1 | 80 | 1 | 0 | 1 | 0 | 0 | bta-miR-2390 | -0.22 |
| SLC1A2 | ENST00000278379.3 | solute carrier family 1 (glial high affinity glutamate transporter), member 2 | 5 | 4 | 0 | 2 | 2 | 4 | bta-miR-2390 | -0.22 |
| CDH20 | ENST00000262717.4 | cadherin 20, type 2 | 5 | 2 | 1 | 0 | 1 | 0 | bta-miR-2390 | -0.22 |
| RSAD2 | ENST00000382040.3 | radical S-adenosyl methionine domain containing 2 | 5 | 1 | 1 | 0 | 0 | 0 | bta-miR-2345 | -0.22 |
| EBF4 | ENST00000380648.4 | early B-cell factor 4 | 1194 | 2 | 0 | 0 | 2 | 1 | bta-miR-2390 | -0.22 |
| IQCG | ENST00000265239.6 | IQ motif containing G | 22 | 1 | 0 | 1 | 0 | 0 | bta-miR-2390 | -0.22 |
| TDRD6 | ENST00000544460.1 | tudor domain containing 6 | 65 | 1 | 0 | 0 | 1 | 0 | bta-miR-2345 | -0.22 |
| IL1RN | ENST00000361779.3 | interleukin 1 receptor antagonist | 19 | 1 | 1 | 0 | 0 | 0 | bta-miR-2345 | -0.22 |
| CELF4 | ENST00000334919.5 | CUGBP, Elav-like family member 4 | 5 | 1 | 1 | 0 | 0 | 0 | bta-miR-2390 | -0.22 |
| GRID1 | ENST00000327946.7 | glutamate receptor, ionotropic, delta 1 | 45 | 1 | 0 | 0 | 1 | 0 | bta-miR-2390 | -0.22 |
| TEX9 | ENST00000558083.2 | testis expressed 9 | 10 | 2* | 0 | 0 | 1 | 0 | bta-miR-2390 | -0.22 |
| PCDHA13 | ENST00000289272.2 | protocadherin alpha 13 | 86 | 1 | 0 | 1 | 0 | 2 | bta-miR-2390 | -0.22 |
| PTAR1 | ENST00000377200.5 | protein prenyltransferase alpha subunit repeat containing 1 | 161 | 2 | 2 | 0 | 0 | 2 | bta-miR-2390 | -0.22 |
| ANGPTL6 | ENST00000589181.1 | angiopoietin-like 6 | 5 | 1 | 0 | 0 | 1 | 0 | bta-miR-2345 | -0.22 |
| PAPPA2 | ENST00000367662.3 | pappalysin 2 | 5 | 1 | 1 | 0 | 0 | 0 | bta-miR-2390 | -0.21 |
| PTP4A2 | ENST00000602725.1 | protein tyrosine phosphatase type IVA, member 2 | 5 | 2 | 0 | 0 | 2 | 1 | bta-miR-2390 | -0.21 |
| PORCN | ENST00000355961.4 | porcupine homolog (Drosophila) | 35 | 1 | 0 | 0 | 1 | 0 | bta-miR-2390 | -0.21 |
| WT1 | ENST00000379079.2 | Wilms tumor 1 | 29 | 1 | 1 | 0 | 0 | 0 | bta-miR-2345 | -0.21 |
| KCND2 | ENST00000331113.4 | potassium voltage-gated channel, Shal-related subfamily, member 2 | 5 | 2 | 0 | 1 | 1 | 1 | bta-miR-2390 | -0.21 |
| CMBL | ENST00000296658.3 | carboxymethylenebutenolidase homolog (Pseudomonas) | 2602 | 1 | 0 | 0 | 1 | 0 | bta-miR-2390 | -0.21 |
| DAND5 | ENST00000585548.1 | DAN domain family member 5, BMP antagonist | 11 | 1 | 0 | 1 | 0 | 0 | bta-miR-2390 | -0.21 |
| LRRTM4 | ENST00000409088.3 | leucine rich repeat transmembrane neuronal 4 | 5 | 1 | 1 | 0 | 0 | 0 | bta-miR-2390 | -0.21 |
| ISCU | ENST00000338291.4 | iron-sulfur cluster assembly enzyme | 182 | 1 | 0 | 1 | 0 | 0 | bta-miR-2345 | -0.21 |
| HIST2H2BF | ENST00000545683.1 | histone cluster 2, H2bf | 5 | 1 | 0 | 1 | 0 | 0 | bta-miR-2345 | -0.21 |
| WBP1L | ENST00000369889.4 | WW domain binding protein 1-like | 5 | 1 | 1 | 0 | 0 | 0 | bta-miR-2390 | -0.21 |
| GLS2 | ENST00000311966.4 | glutaminase 2 (liver, mitochondrial) | 10 | 1 | 0 | 0 | 1 | 0 | bta-miR-2345 | -0.21 |
| ZPBP | ENST00000046087.2 | zona pellucida binding protein | 5 | 1 | 0 | 0 | 1 | 0 | bta-miR-2345 | -0.21 |
| TMEM19 | ENST00000266673.5 | transmembrane protein 19 | 1378 | 1 | 1 | 0 | 0 | 1 | bta-miR-2345 | -0.21 |
| CCDC39 | ENST00000273654.4 | coiled-coil domain containing 39 | 5 | 2 | 0 | 1 | 1 | 0 | bta-miR-2345 | -0.21 |
| WDR6 | ENST00000395474.3 | WD repeat domain 6 | 3435 | 1 | 0 | 1 | 0 | 0 | bta-miR-2390 | -0.21 |
| SOX12 | ENST00000342665.2 | SRY (sex determining region Y)-box 12 | 6341 | 1 | 1 | 0 | 0 | 1 | bta-miR-2390 | -0.21 |
| NUP62CL | ENST00000372461.3 | nucleoporin 62kDa C-terminal like | 73 | 1 | 0 | 0 | 1 | 1 | bta-miR-2345 | -0.21 |
| LRRC8C | ENST00000370454.4 | leucine rich repeat containing 8 family, member C | 544 | 2 | 0 | 0 | 2 | 0 | bta-miR-2390 | -0.21 |
| LAMP2 | ENST00000371335.4 | lysosomal-associated membrane protein 2 | 3095 | 1 | 1 | 0 | 0 | 0 | bta-miR-2390 | -0.21 |
| MAP4K3 | ENST00000263881.3 | mitogen-activated protein kinase kinase kinase kinase 3 | 796 | 2 | 0 | 1 | 1 | 0 | bta-miR-2345 | -0.21 |
| CDC26 | ENST00000374206.3 | cell division cycle 26 | 8 | 1 | 0 | 0 | 1 | 0 | bta-miR-2345 | -0.21 |
| FBXO36 | ENST00000373652.3 | F-box protein 36 | 5 | 1 | 0 | 0 | 1 | 0 | bta-miR-2345 | -0.21 |
| KDM4C | ENST00000381309.3 | lysine (K)-specific demethylase 4C | 160 | 1 | 0 | 0 | 1 | 0 | bta-miR-2390 | -0.21 |
| ZBTB2 | ENST00000325144.4 | zinc finger and BTB domain containing 2 | 832 | 1 | 0 | 0 | 1 | 0 | bta-miR-2390 | -0.21 |
| EMP1 | ENST00000256951.5 | epithelial membrane protein 1 | 814 | 1 | 1 | 0 | 0 | 0 | bta-miR-2345 | -0.21 |
| FKBP1B | ENST00000380991.4 | FK506 binding protein 1B, 12.6 kDa | 232 | 1 | 0 | 1 | 0 | 0 | bta-miR-2345 | -0.21 |
| KLF12 | ENST00000377669.2 | Kruppel-like factor 12 | 251 | 6 | 0 | 4 | 2 | 1 | bta-miR-2390 | -0.21 |
| HSPE1-MOB4 | ENST00000604458.1 | HSPE1-MOB4 readthrough | 123 | 2 | 1 | 0 | 1 | 0 | bta-miR-2345 | -0.21 |

**Predicted target mRNAs of mir-2403**

| Ortholog of target gene | Representative transcript | Gene name | 3P-seq tags + 5 | Total sites | 8mer sites | 7mer-m8 sites | 7mer-A1 sites | 6mer sites | Representative miRNA | Cumulative weighted context++ score |
| --- | --- | --- | --- | --- | --- | --- | --- | --- | --- | --- |
| C21orf2 | ENST00000339818.4 | chromosome 21 open reading frame 2 | 205 | 2 | 2 | 0 | 0 | 0 | bta-miR-2403 | -1.37 |
| RNF146 | ENST00000356799.2 | ring finger protein 146 | 1504 | 1 | 1 | 0 | 0 | 0 | bta-miR-425-3p | -1.06 |
| ACOXL | ENST00000340561.4 | acyl-CoA oxidase-like | 5 | 1 | 1 | 0 | 0 | 0 | bta-miR-425-3p | -0.97 |
| OR6K2 | ENST00000359610.2 | olfactory receptor, family 6, subfamily K, member 2 | 5 | 1 | 1 | 0 | 0 | 0 | bta-miR-425-3p | -0.94 |
| KAAG1 | ENST00000274766.1 | kidney associated antigen 1 | 5 | 1 | 1 | 0 | 0 | 0 | bta-miR-2403 | -0.94 |
| RP11-1102P16.1 | ENST00000523987.1 | Uncharacterized protein | 5 | 1 | 1 | 0 | 0 | 0 | bta-miR-425-3p | -0.92 |
| FAM19A3 | ENST00000361886.3 | family with sequence similarity 19 (chemokine (C-C motif)-like), member A3 | 5 | 1 | 1 | 0 | 0 | 0 | bta-miR-2403 | -0.91 |
| C9orf116 | ENST00000371789.3 | chromosome 9 open reading frame 116 | 206 | 1 | 1 | 0 | 0 | 0 | bta-miR-2403 | -0.91 |
| MYBPC2 | ENST00000357701.5 | myosin binding protein C, fast type | 25 | 1 | 1 | 0 | 0 | 0 | bta-miR-425-3p | -0.9 |
| ATP6V1G2 | ENST00000376151.4 | ATPase, H+ transporting, lysosomal 13kDa, V1 subunit G2 | 16 | 1 | 1 | 0 | 0 | 0 | bta-miR-2403 | -0.82 |
| ASH2L | ENST00000343823.6 | ash2 (absent, small, or homeotic)-like (Drosophila) | 140 | 1 | 1 | 0 | 0 | 0 | bta-miR-2403 | -0.81 |
| OTX1 | ENST00000366671.3 | orthodenticle homeobox 1 | 11 | 1 | 1 | 0 | 0 | 0 | bta-miR-425-3p | -0.8 |
| TMEM100 | ENST00000424486.2 | transmembrane protein 100 | 86 | 1 | 1 | 0 | 0 | 0 | bta-miR-2403 | -0.78 |
| BOC | ENST00000485230.1 | BOC cell adhesion associated, oncogene regulated | 159 | 1 | 1 | 0 | 0 | 0 | bta-miR-2403 | -0.77 |
| RNF224 | ENST00000445101.2 | ring finger protein 224 | 5 | 1 | 1 | 0 | 0 | 0 | bta-miR-425-3p | -0.74 |
| HPRT1 | ENST00000298556.7 | hypoxanthine phosphoribosyltransferase 1 | 490 | 1 | 1 | 0 | 0 | 0 | bta-miR-2403 | -0.73 |
| FGF5 | ENST00000456523.3 | fibroblast growth factor 5 | 1478 | 1 | 1 | 0 | 0 | 0 | bta-miR-2403 | -0.72 |
| TMEM199 | ENST00000292114.3 | transmembrane protein 199 | 739 | 1 | 1 | 0 | 0 | 0 | bta-miR-425-3p | -0.71 |
| DHRS4 | ENST00000558263.1 | dehydrogenase/reductase (SDR family) member 4 | 5 | 1 | 1 | 0 | 0 | 0 | bta-miR-2403 | -0.71 |
| ANGEL2 | ENST00000535388.1 | angel homolog 2 (Drosophila) | 218 | 1 | 1 | 0 | 0 | 0 | bta-miR-425-3p | -0.7 |
| RNF182 | ENST00000488300.1 | ring finger protein 182 | 1122 | 1 | 1 | 0 | 0 | 0 | bta-miR-425-3p | -0.7 |
| SEC31A | ENST00000348405.4 | SEC31 homolog A (S. cerevisiae) | 44 | 1 | 1 | 0 | 0 | 0 | bta-miR-2403 | -0.69 |
| DHRS4L2 | ENST00000335125.6 | dehydrogenase/reductase (SDR family) member 4 like 2 | 5 | 1 | 1 | 0 | 0 | 0 | bta-miR-2403 | -0.66 |
| CHEK2 | ENST00000382566.1 | checkpoint kinase 2 | 5 | 1 | 1 | 0 | 0 | 0 | bta-miR-425-3p | -0.65 |
| ZNF143 | ENST00000396602.2 | zinc finger protein 143 | 380 | 1 | 1 | 0 | 0 | 0 | bta-miR-425-3p | -0.65 |
| PAPD7 | ENST00000230859.6 | PAP associated domain containing 7 | 3195 | 3 | 0 | 2 | 1 | 1 | bta-miR-425-3p | -0.64 |
| TRPV2 | ENST00000338560.7 | transient receptor potential cation channel, subfamily V, member 2 | 22341 | 2 | 0 | 1 | 1 | 1 | bta-miR-425-3p | -0.62 |
| LIMA1 | ENST00000552491.1 | LIM domain and actin binding 1 | 780 | 1 | 1 | 0 | 0 | 0 | bta-miR-425-3p | -0.62 |
| SUMF1 | ENST00000272902.5 | sulfatase modifying factor 1 | 1341 | 1 | 1 | 0 | 0 | 0 | bta-miR-2403 | -0.6 |
| P2RY13 | ENST00000325602.5 | purinergic receptor P2Y, G-protein coupled, 13 | 5 | 1 | 1 | 0 | 0 | 0 | bta-miR-2403 | -0.6 |
| ATG9B | ENST00000605938.1 | autophagy related 9B | 5 | 1 | 1 | 0 | 0 | 0 | bta-miR-2403 | -0.59 |
| DCTD | ENST00000357067.3 | dCMP deaminase | 747 | 2 | 0 | 1 | 1 | 0 | bta-miR-425-3p | -0.59 |
| LYL1 | ENST00000264824.4 | lymphoblastic leukemia derived sequence 1 | 39 | 2 | 0 | 0 | 2 | 0 | bta-miR-425-3p | -0.59 |
| PALLD | ENST00000261509.6 | palladin, cytoskeletal associated protein | 4423 | 1 | 1 | 0 | 0 | 0 | bta-miR-2403 | -0.59 |
| ZMAT2 | ENST00000274712.3 | zinc finger, matrin-type 2 | 1926 | 1 | 1 | 0 | 0 | 0 | bta-miR-2403 | -0.59 |
| KCTD15 | ENST00000284006.6 | potassium channel tetramerization domain containing 15 | 2483 | 1 | 1 | 0 | 0 | 1 | bta-miR-425-3p | -0.58 |
| ZNHIT6 | ENST00000431532.2 | zinc finger, HIT-type containing 6 | 201 | 1 | 1 | 0 | 0 | 0 | bta-miR-2403 | -0.58 |
| RNF122 | ENST00000256257.1 | ring finger protein 122 | 16 | 2 | 0 | 1 | 1 | 0 | bta-miR-425-3p | -0.58 |
| ATG16L1 | ENST00000392017.4 | autophagy related 16-like 1 (S. cerevisiae) | 552 | 1 | 1 | 0 | 0 | 1 | bta-miR-2403 | -0.58 |
| XYLT1 | ENST00000261381.6 | xylosyltransferase I | 159 | 3 | 0 | 0 | 3 | 0 | bta-miR-2403 | -0.58 |
| MUC15 | ENST00000455601.2 | mucin 15, cell surface associated | 7 | 1 | 1 | 0 | 0 | 0 | bta-miR-2403 | -0.57 |
| ISM1 | ENST00000262487.4 | isthmin 1, angiogenesis inhibitor | 9 | 2 | 0 | 1 | 1 | 0 | bta-miR-2403 | -0.57 |
| SSBP3 | ENST00000417664.2 | single stranded DNA binding protein 3 | 1474 | 1 | 1 | 0 | 0 | 0 | bta-miR-2403 | -0.56 |
| SLC1A4 | ENST00000234256.3 | solute carrier family 1 (glutamate/neutral amino acid transporter), member 4 | 380 | 1 | 1 | 0 | 0 | 1 | bta-miR-425-3p | -0.56 |
| TNFSF8 | ENST00000223795.2 | tumor necrosis factor (ligand) superfamily, member 8 | 5 | 1 | 1 | 0 | 0 | 1 | bta-miR-2403 | -0.56 |
| PPP1R13B | ENST00000423488.2 | protein phosphatase 1, regulatory subunit 13B | 105 | 2 | 0 | 2 | 0 | 0 | bta-miR-2403 | -0.55 |
| FJX1 | ENST00000317811.4 | four jointed box 1 (Drosophila) | 823 | 1 | 1 | 0 | 0 | 0 | bta-miR-2403 | -0.55 |
| CYB561A3 | ENST00000294072.4 | cytochrome b561 family, member A3 | 470 | 1 | 1 | 0 | 0 | 0 | bta-miR-425-3p | -0.54 |
| AL031666.2 | ENST00000599904.1 | HCG2018772; Uncharacterized protein; cDNA FLJ31609 fis, clone NT2RI2002852 | 5 | 1 | 1 | 0 | 0 | 0 | bta-miR-425-3p | -0.54 |
| TRIM39 | ENST00000396551.3 | tripartite motif containing 39 | 840 | 1 | 1 | 0 | 0 | 0 | bta-miR-425-3p | -0.54 |
| SH3BP5L | ENST00000366472.5 | SH3-binding domain protein 5-like | 379 | 1 | 1 | 0 | 0 | 0 | bta-miR-425-3p | -0.53 |
| RRAD | ENST00000420652.1 | Ras-related associated with diabetes | 28 | 1 | 0 | 1 | 0 | 0 | bta-miR-425-3p | -0.53 |
| RASSF5 | ENST00000367117.3 | Ras association (RalGDS/AF-6) domain family member 5 | 27 | 2 | 0 | 1 | 1 | 0 | bta-miR-2403 | -0.53 |
| AL359878.1 | ENST00000381466.1 | Uncharacterized protein | 5 | 1 | 0 | 1 | 0 | 0 | bta-miR-2403 | -0.53 |
| CYP8B1 | ENST00000316161.4 | cytochrome P450, family 8, subfamily B, polypeptide 1 | 5 | 1 | 1 | 0 | 0 | 0 | bta-miR-2403 | -0.52 |
| F11R | ENST00000368026.6 | F11 receptor | 2844 | 1 | 1 | 0 | 0 | 0 | bta-miR-2403 | -0.52 |
| PCK1 | ENST00000319441.4 | phosphoenolpyruvate carboxykinase 1 (soluble) | 5 | 1 | 1 | 0 | 0 | 0 | bta-miR-425-3p | -0.52 |
| CACUL1 | ENST00000369151.3 | CDK2-associated, cullin domain 1 | 213 | 1 | 1 | 0 | 0 | 0 | bta-miR-425-3p | -0.52 |
| TRABD2A | ENST00000409133.1 | TraB domain containing 2A | 94 | 1 | 0 | 0 | 1 | 0 | bta-miR-425-3p | -0.51 |
| SCML2 | ENST00000251900.4 | sex comb on midleg-like 2 (Drosophila) | 7 | 1 | 1 | 0 | 0 | 0 | bta-miR-2403 | -0.51 |
| CCDC103 | ENST00000417826.2 | coiled-coil domain containing 103 | 72 | 1 | 1 | 0 | 0 | 0 | bta-miR-2403 | -0.5 |
| HSPB2 | ENST00000537382.1 | Homo sapiens heat shock 27kDa protein 2 (HSPB2), mRNA. | 5 | 1 | 0 | 0 | 1 | 0 | bta-miR-2403 | -0.49 |
| RAX | ENST00000256852.7 | retina and anterior neural fold homeobox | 5 | 1 | 1 | 0 | 0 | 0 | bta-miR-2403 | -0.49 |
| LALBA | ENST00000301046.2 | lactalbumin, alpha- | 5 | 1 | 0 | 0 | 1 | 0 | bta-miR-425-3p | -0.49 |
| DDAH1 | ENST00000535924.2 | dimethylarginine dimethylaminohydrolase 1 | 46 | 2 | 0 | 1 | 1 | 0 | bta-miR-2403 | -0.49 |
| UPK3A | ENST00000216211.4 | uroplakin 3A | 5 | 1 | 0 | 1 | 0 | 0 | bta-miR-425-3p | -0.49 |
| NFIA | ENST00000403491.3 | nuclear factor I/A | 5 | 2 | 1 | 0 | 1 | 0 | bta-miR-2403 | -0.48 |
| TSEN34 | ENST00000302937.4 | TSEN34 tRNA splicing endonuclease subunit | 2691 | 1 | 0 | 1 | 0 | 0 | bta-miR-2403 | -0.48 |
| FOXD1 | ENST00000499003.3 | forkhead box D1 | 423 | 1 | 0 | 0 | 1 | 2 | bta-miR-2403 | -0.48 |
| GRAMD1B | ENST00000529750.1 | GRAM domain containing 1B | 35 | 1 | 1 | 0 | 0 | 0 | bta-miR-2403 | -0.47 |
| C2orf50 | ENST00000381585.3 | chromosome 2 open reading frame 50 | 5 | 1 | 0 | 0 | 1 | 0 | bta-miR-2403 | -0.47 |
| GPR114 | ENST00000340339.4 | G protein-coupled receptor 114 | 5 | 1 | 1 | 0 | 0 | 0 | bta-miR-2403 | -0.47 |
| GCSAM | ENST00000308910.4 | germinal center-associated, signaling and motility | 5 | 1 | 0 | 0 | 1 | 0 | bta-miR-425-3p | -0.47 |
| ERN2 | ENST00000457008.2 | endoplasmic reticulum to nucleus signaling 2 | 5 | 1 | 0 | 0 | 1 | 0 | bta-miR-2403 | -0.47 |
| WDR26 | ENST00000414423.2 | WD repeat domain 26 | 1229 | 2 | 2 | 0 | 0 | 0 | bta-miR-2403 | -0.47 |
| FLNB | ENST00000357272.4 | filamin B, beta | 467 | 1 | 1 | 0 | 0 | 1 | bta-miR-2403 | -0.46 |
| LCE3E | ENST00000368789.1 | late cornified envelope 3E | 5 | 1 | 0 | 1 | 0 | 0 | bta-miR-425-3p | -0.46 |
| PHF2 | ENST00000375376.4 | PHD finger protein 2 | 151 | 1 | 1 | 0 | 0 | 0 | bta-miR-2403 | -0.46 |
| CDKL5 | ENST00000379996.3 | cyclin-dependent kinase-like 5 | 5 | 1 | 0 | 0 | 1 | 0 | bta-miR-2403 | -0.46 |
| PPCDC | ENST00000342932.3 | phosphopantothenoylcysteine decarboxylase | 26 | 1 | 0 | 1 | 0 | 1 | bta-miR-425-3p | -0.46 |
| SULF1 | ENST00000458141.2 | sulfatase 1 | 11 | 1 | 1 | 0 | 0 | 0 | bta-miR-425-3p | -0.45 |
| C3orf80 | ENST00000326474.3 | chromosome 3 open reading frame 80 | 296 | 1 | 1 | 0 | 0 | 0 | bta-miR-2403 | -0.45 |
| FOXQ1 | ENST00000296839.2 | forkhead box Q1 | 181 | 1 | 0 | 0 | 1 | 0 | bta-miR-425-3p | -0.45 |
| RAB5A | ENST00000273047.4 | RAB5A, member RAS oncogene family | 68 | 1 | 0 | 0 | 1 | 0 | bta-miR-2403 | -0.45 |
| KISS1 | ENST00000367194.4 | KiSS-1 metastasis-suppressor | 14 | 1 | 0 | 0 | 1 | 0 | bta-miR-425-3p | -0.44 |
| PRX | ENST00000291825.7 | periaxin | 17 | 2 | 0 | 0 | 2 | 1 | bta-miR-425-3p | -0.44 |
| ITGA2B | ENST00000377068.3 | integrin, alpha 2b (platelet glycoprotein IIb of IIb/IIIa complex, antigen CD41) | 5 | 1 | 0 | 1 | 0 | 0 | bta-miR-425-3p | -0.44 |
| GCLC | ENST00000229416.6 | glutamate-cysteine ligase, catalytic subunit | 1001 | 1 | 1 | 0 | 0 | 0 | bta-miR-2403 | -0.43 |
| TUBB1 | ENST00000217133.1 | tubulin, beta 1 class VI | 5 | 1 | 1 | 0 | 0 | 0 | bta-miR-2403 | -0.43 |
| COLQ | ENST00000383785.2 | collagen-like tail subunit (single strand of homotrimer) of asymmetric acetylcholinesterase | 8 | 1 | 1 | 0 | 0 | 0 | bta-miR-2403 | -0.43 |
| MLANA | ENST00000381477.3 | melan-A | 5 | 1 | 0 | 1 | 0 | 0 | bta-miR-425-3p | -0.43 |
| ADAMTS2 | ENST00000251582.7 | ADAM metallopeptidase with thrombospondin type 1 motif, 2 | 2767 | 1 | 1 | 0 | 0 | 0 | bta-miR-2403 | -0.43 |
| DUSP1 | ENST00000239223.3 | dual specificity phosphatase 1 | 37211 | 1 | 0 | 1 | 0 | 0 | bta-miR-2403 | -0.42 |
| ACOT7 | ENST00000608083.1 | acyl-CoA thioesterase 7 | 141 | 1 | 0 | 0 | 1 | 0 | bta-miR-425-3p | -0.42 |
| KIAA1549 | ENST00000440172.1 | KIAA1549 | 508 | 2 | 0 | 0 | 2 | 2 | bta-miR-425-3p | -0.42 |
| ABCC5 | ENST00000334444.6 | ATP-binding cassette, sub-family C (CFTR/MRP), member 5 | 453 | 1 | 0 | 1 | 0 | 1 | bta-miR-425-3p | -0.42 |
| SGCA | ENST00000262018.3 | sarcoglycan, alpha (50kDa dystrophin-associated glycoprotein) | 14 | 1 | 0 | 1 | 0 | 0 | bta-miR-425-3p | -0.41 |
| MYO18B | ENST00000335473.7 | myosin XVIIIB | 39 | 1 | 0 | 1 | 0 | 1 | bta-miR-2403 | -0.41 |
| NUTF2 | ENST00000219169.4 | nuclear transport factor 2 | 222 | 1 | 0 | 1 | 0 | 0 | bta-miR-2403 | -0.41 |
| FKBP14 | ENST00000222803.5 | FK506 binding protein 14, 22 kDa | 939 | 1 | 0 | 0 | 1 | 0 | bta-miR-2403 | -0.41 |
| PRPH2 | ENST00000230381.5 | peripherin 2 (retinal degeneration, slow) | 5 | 1 | 1 | 0 | 0 | 0 | bta-miR-2403 | -0.41 |
| RRAS | ENST00000246792.3 | related RAS viral (r-ras) oncogene homolog | 267 | 1 | 0 | 1 | 0 | 0 | bta-miR-2403 | -0.41 |
| RTN4RL2 | ENST00000335099.3 | reticulon 4 receptor-like 2 | 13 | 1 | 0 | 1 | 0 | 1 | bta-miR-2403 | -0.4 |
| NRGN | ENST00000412681.2 | neurogranin (protein kinase C substrate, RC3) | 60 | 1 | 0 | 1 | 0 | 0 | bta-miR-425-3p | -0.4 |
| RRNAD1 | ENST00000368218.4 | ribosomal RNA adenine dimethylase domain containing 1 | 367 | 1 | 0 | 0 | 1 | 0 | bta-miR-2403 | -0.4 |
| BIN1 | ENST00000376113.2 | bridging integrator 1 | 48 | 1 | 0 | 0 | 1 | 0 | bta-miR-2403 | -0.4 |
| SERPINB9 | ENST00000380698.4 | serpin peptidase inhibitor, clade B (ovalbumin), member 9 | 154 | 1 | 0 | 1 | 0 | 1 | bta-miR-425-3p | -0.4 |
| SPAG11B | ENST00000297498.2 | sperm associated antigen 11B | 5 | 1 | 0 | 1 | 0 | 0 | bta-miR-2403 | -0.4 |
| TSPYL2 | ENST00000375442.4 | TSPY-like 2 | 270 | 1 | 0 | 0 | 1 | 0 | bta-miR-425-3p | -0.4 |
| SETD6 | ENST00000394266.4 | SET domain containing 6 | 121 | 1 | 0 | 0 | 1 | 0 | bta-miR-2403 | -0.4 |
| RPL17-C18orf32 | ENST00000584895.1 | RPL17-C18orf32 readthrough | 733 | 1 | 0 | 0 | 1 | 0 | bta-miR-425-3p | -0.4 |
| MACROD2 | ENST00000217246.4 | MACRO domain containing 2 | 5 | 2 | 0 | 1 | 1 | 1 | bta-miR-2403 | -0.4 |
| RAB30 | ENST00000533486.1 | RAB30, member RAS oncogene family | 274 | 1 | 0 | 1 | 0 | 1 | bta-miR-2403 | -0.39 |
| CHD9 | ENST00000566029.1 | chromodomain helicase DNA binding protein 9 | 447 | 1 | 1 | 0 | 0 | 0 | bta-miR-2403 | -0.39 |
| TGFB1I1 | ENST00000361773.3 | transforming growth factor beta 1 induced transcript 1 | 164 | 1 | 0 | 0 | 1 | 0 | bta-miR-425-3p | -0.39 |
| AL441883.1 | ENST00000600057.1 | Uncharacterized protein | 5 | 1 | 0 | 0 | 1 | 0 | bta-miR-425-3p | -0.38 |
| BRSK2 | ENST00000382179.1 | BR serine/threonine kinase 2 | 15 | 1 | 0 | 0 | 1 | 0 | bta-miR-2403 | -0.38 |
| INSC | ENST00000528567.1 | inscuteable homolog (Drosophila) | 27 | 1 | 0 | 0 | 1 | 0 | bta-miR-2403 | -0.38 |
| CDON | ENST00000392693.3 | cell adhesion associated, oncogene regulated | 173 | 1 | 1 | 0 | 0 | 1 | bta-miR-425-3p | -0.37 |
| SDCCAG3 | ENST00000357365.3 | serologically defined colon cancer antigen 3 | 787 | 1 | 0 | 1 | 0 | 0 | bta-miR-425-3p | -0.37 |
| SYPL2 | ENST00000369872.3 | synaptophysin-like 2 | 69 | 1 | 0 | 0 | 1 | 0 | bta-miR-2403 | -0.37 |
| C11orf96 | ENST00000528572.1 | chromosome 11 open reading frame 96 | 480 | 1 | 0 | 0 | 1 | 0 | bta-miR-425-3p | -0.37 |
| ACSS1 | ENST00000323482.4 | acyl-CoA synthetase short-chain family member 1 | 12 | 1 | 0 | 0 | 1 | 1 | bta-miR-2403 | -0.37 |
| SCN8A | ENST00000354534.6 | sodium channel, voltage gated, type VIII, alpha subunit | 44 | 1 | 0 | 0 | 1 | 0 | bta-miR-425-3p | -0.36 |
| VEGFA | ENST00000372067.3 | vascular endothelial growth factor A | 907 | 1 | 0 | 1 | 0 | 1 | bta-miR-2403 | -0.36 |
| MYH9 | ENST00000216181.5 | myosin, heavy chain 9, non-muscle | 1317 | 1 | 0 | 0 | 1 | 1 | bta-miR-425-3p | -0.36 |
| ONECUT3 | ENST00000382349.4 | one cut homeobox 3 | 5 | 1 | 0 | 0 | 1 | 1 | bta-miR-425-3p | -0.36 |
| PIK3CG | ENST00000359195.3 | phosphatidylinositol-4,5-bisphosphate 3-kinase, catalytic subunit gamma | 5 | 1 | 1 | 0 | 0 | 0 | bta-miR-2403 | -0.36 |
| C2orf91 | ENST00000378711.2 | chromosome 2 open reading frame 91 | 5 | 1 | 0 | 1 | 0 | 0 | bta-miR-425-3p | -0.36 |
| EIF3C | ENST00000331666.6 | eukaryotic translation initiation factor 3, subunit C | 5 | 1 | 0 | 1 | 0 | 0 | bta-miR-425-3p | -0.36 |
| EIF3H | ENST00000521861.1 | eukaryotic translation initiation factor 3, subunit H | 27 | 1 | 1 | 0 | 0 | 1 | bta-miR-2403 | -0.36 |
| EIF3CL | ENST00000398944.3 | eukaryotic translation initiation factor 3, subunit C-like | 5 | 1 | 0 | 1 | 0 | 0 | bta-miR-425-3p | -0.36 |
| GLI2 | ENST00000452319.1 | GLI family zinc finger 2 | 73 | 1 | 1 | 0 | 0 | 0 | bta-miR-425-3p | -0.36 |
| RAB42 | ENST00000373826.3 | RAB42, member RAS oncogene family | 11 | 1 | 0 | 0 | 1 | 0 | bta-miR-2403 | -0.36 |
| RHBDF1 | ENST00000262316.6 | rhomboid 5 homolog 1 (Drosophila) | 365 | 1 | 0 | 1 | 0 | 0 | bta-miR-425-3p | -0.35 |
| TAB1 | ENST00000216160.6 | TGF-beta activated kinase 1/MAP3K7 binding protein 1 | 577 | 1 | 0 | 1 | 0 | 2 | bta-miR-2403 | -0.35 |
| CD276 | ENST00000318443.5 | CD276 molecule | 186 | 1 | 0 | 1 | 0 | 0 | bta-miR-425-3p | -0.35 |
| FCRLB | ENST00000336830.5 | Fc receptor-like B | 87 | 1 | 0 | 0 | 1 | 0 | bta-miR-425-3p | -0.35 |
| SIGLEC5 | ENST00000222107.4 | sialic acid binding Ig-like lectin 5 | 5 | 1 | 0 | 0 | 1 | 0 | bta-miR-425-3p | -0.35 |
| PRIMA1 | ENST00000393140.1 | proline rich membrane anchor 1 | 5 | 1 | 0 | 1 | 0 | 1 | bta-miR-425-3p | -0.35 |
| TIMP4 | ENST00000287814.4 | TIMP metallopeptidase inhibitor 4 | 38 | 1 | 0 | 0 | 1 | 1 | bta-miR-425-3p | -0.35 |
| ERMP1 | ENST00000381506.3 | endoplasmic reticulum metallopeptidase 1 | 53 | 1 | 0 | 1 | 0 | 0 | bta-miR-425-3p | -0.35 |
| CYB5R1 | ENST00000367249.4 | cytochrome b5 reductase 1 | 545 | 1 | 0 | 1 | 0 | 0 | bta-miR-425-3p | -0.35 |
| GHR | ENST00000230882.4 | growth hormone receptor | 102 | 1 | 0 | 0 | 1 | 0 | bta-miR-2403 | -0.35 |
| BCR | ENST00000305877.8 | breakpoint cluster region | 5 | 1 | 0 | 0 | 1 | 1 | bta-miR-425-3p | -0.34 |
| UBXN2A | ENST00000309033.4 | UBX domain protein 2A | 45 | 1 | 0 | 1 | 0 | 0 | bta-miR-2403 | -0.34 |
| NRARP | ENST00000356628.2 | NOTCH-regulated ankyrin repeat protein | 56 | 1 | 0 | 0 | 1 | 0 | bta-miR-425-3p | -0.34 |
| C12orf76 | ENST00000546651.2 | chromosome 12 open reading frame 76 | 62 | 1 | 0 | 0 | 1 | 0 | bta-miR-2403 | -0.34 |
| HECTD2 | ENST00000371667.1 | HECT domain containing E3 ubiquitin protein ligase 2 | 1514 | 1 | 0 | 1 | 0 | 0 | bta-miR-425-3p | -0.34 |
| SPATA8 | ENST00000328504.3 | spermatogenesis associated 8 | 10 | 1 | 0 | 0 | 1 | 0 | bta-miR-425-3p | -0.34 |
| GPR107 | ENST00000372410.3 | G protein-coupled receptor 107 | 1429 | 1 | 0 | 0 | 1 | 1 | bta-miR-2403 | -0.34 |
| PRMT8 | ENST00000382622.3 | protein arginine methyltransferase 8 | 5 | 1 | 0 | 1 | 0 | 1 | bta-miR-2403 | -0.34 |
| TMEM107 | ENST00000532998.1 | transmembrane protein 107 | 585 | 1 | 0 | 1 | 0 | 0 | bta-miR-425-3p | -0.34 |
| FAM217B | ENST00000358293.3 | family with sequence similarity 217, member B | 175 | 1 | 0 | 1 | 0 | 0 | bta-miR-425-3p | -0.34 |
| AQP8 | ENST00000219660.5 | aquaporin 8 | 7 | 1 | 0 | 1 | 0 | 0 | bta-miR-2403 | -0.34 |
| SERHL2 | ENST00000340239.4 | serine hydrolase-like 2 | 5 | 1 | 0 | 0 | 1 | 0 | bta-miR-425-3p | -0.34 |
| IP6K3 | ENST00000451316.1 | inositol hexakisphosphate kinase 3 | 8 | 1 | 0 | 1 | 0 | 0 | bta-miR-2403 | -0.34 |
| CDK5R1 | ENST00000313401.3 | cyclin-dependent kinase 5, regulatory subunit 1 (p35) | 335 | 1 | 0 | 0 | 1 | 1 | bta-miR-425-3p | -0.33 |
| KRT77 | ENST00000341809.3 | keratin 77 | 5 | 1 | 0 | 1 | 0 | 0 | bta-miR-425-3p | -0.33 |
| PRKRIP1 | ENST00000496391.1 | PRKR interacting protein 1 (IL11 inducible) | 656 | 1 | 0 | 0 | 1 | 0 | bta-miR-2403 | -0.33 |
| CLVS1 | ENST00000518592.1 | clavesin 1 | 5 | 1 | 0 | 0 | 1 | 0 | bta-miR-425-3p | -0.33 |
| MECP2 | ENST00000303391.6 | methyl CpG binding protein 2 (Rett syndrome) | 1474 | 1 | 0 | 1 | 0 | 0 | bta-miR-425-3p | -0.33 |
| DLGAP3 | ENST00000373347.1 | discs, large (Drosophila) homolog-associated protein 3 | 8 | 1 | 0 | 0 | 1 | 0 | bta-miR-425-3p | -0.33 |
| ZNF436 | ENST00000314011.4 | zinc finger protein 436 | 73 | 1 | 0 | 0 | 1 | 0 | bta-miR-2403 | -0.33 |
| GPR146 | ENST00000297468.3 | G protein-coupled receptor 146 | 5 | 1 | 0 | 0 | 1 | 0 | bta-miR-425-3p | -0.33 |
| MAL2 | ENST00000276681.6 | mal, T-cell differentiation protein 2 (gene/pseudogene) | 2056 | 1 | 0 | 0 | 1 | 0 | bta-miR-425-3p | -0.33 |
| FANCE | ENST00000229769.2 | Fanconi anemia, complementation group E | 1562 | 1 | 0 | 0 | 1 | 0 | bta-miR-425-3p | -0.33 |
| RAB3D | ENST00000222120.3 | RAB3D, member RAS oncogene family | 111 | 1 | 1 | 0 | 0 | 0 | bta-miR-2403 | -0.33 |
| GUCY2C | ENST00000261170.3 | guanylate cyclase 2C (heat stable enterotoxin receptor) | 5 | 1 | 0 | 0 | 1 | 0 | bta-miR-425-3p | -0.33 |
| UBE2Z | ENST00000360943.5 | ubiquitin-conjugating enzyme E2Z | 3688 | 1 | 0 | 0 | 1 | 0 | bta-miR-425-3p | -0.33 |
| HERC3 | ENST00000402738.1 | HECT and RLD domain containing E3 ubiquitin protein ligase 3 | 50 | 1 | 0 | 0 | 1 | 0 | bta-miR-425-3p | -0.33 |
| CLVS2 | ENST00000275162.5 | clavesin 2 | 19 | 1 | 0 | 0 | 1 | 0 | bta-miR-425-3p | -0.33 |
| SEPT5 | ENST00000438754.2 | septin 5 | 342 | 1 | 0 | 1 | 0 | 1 | bta-miR-425-3p | -0.33 |
| CWC25 | ENST00000225428.5 | CWC25 spliceosome-associated protein homolog (S. cerevisiae) | 300 | 1 | 0 | 0 | 1 | 0 | bta-miR-2403 | -0.33 |
| C17orf107 | ENST00000381365.3 | chromosome 17 open reading frame 107 | 11 | 1 | 0 | 0 | 1 | 0 | bta-miR-425-3p | -0.33 |
| PHKA2 | ENST00000379942.4 | phosphorylase kinase, alpha 2 (liver) | 588 | 1 | 1 | 0 | 0 | 0 | bta-miR-2403 | -0.33 |
| GDA | ENST00000358399.3 | guanine deaminase | 5 | 1 | 1 | 0 | 0 | 0 | bta-miR-425-3p | -0.33 |
| DNAJB6 | ENST00000429029.2 | DnaJ (Hsp40) homolog, subfamily B, member 6 | 420 | 1 | 0 | 1 | 0 | 0 | bta-miR-425-3p | -0.33 |
| NRF1 | ENST00000393230.2 | nuclear respiratory factor 1 | 321 | 1 | 0 | 0 | 1 | 0 | bta-miR-425-3p | -0.33 |
| LRRC3B | ENST00000396641.2 | leucine rich repeat containing 3B | 5 | 1 | 0 | 1 | 0 | 0 | bta-miR-2403 | -0.33 |
| KCNS3 | ENST00000304101.4 | potassium voltage-gated channel, delayed-rectifier, subfamily S, member 3 | 344 | 1 | 0 | 1 | 0 | 0 | bta-miR-425-3p | -0.32 |
| S100A7A | ENST00000368729.4 | S100 calcium binding protein A7A | 5 | 1 | 0 | 1 | 0 | 0 | bta-miR-2403 | -0.32 |
| COG5 | ENST00000347053.3 | component of oligomeric golgi complex 5 | 455 | 1 | 0 | 0 | 1 | 0 | bta-miR-425-3p | -0.32 |
| PRKCA | ENST00000413366.3 | protein kinase C, alpha | 726 | 1 | 0 | 1 | 0 | 0 | bta-miR-2403 | -0.32 |
| ZDHHC7 | ENST00000313732.4 | zinc finger, DHHC-type containing 7 | 4068 | 1 | 0 | 1 | 0 | 1 | bta-miR-425-3p | -0.32 |
| TNFRSF8 | ENST00000263932.2 | tumor necrosis factor receptor superfamily, member 8 | 5 | 1 | 1 | 0 | 0 | 0 | bta-miR-425-3p | -0.32 |
| IMPAD1 | ENST00000262644.4 | inositol monophosphatase domain containing 1 | 1488 | 1 | 0 | 0 | 1 | 0 | bta-miR-2403 | -0.32 |
| NR5A1 | ENST00000373588.4 | nuclear receptor subfamily 5, group A, member 1 | 5 | 1 | 0 | 0 | 1 | 0 | bta-miR-2403 | -0.32 |
| RBM20 | ENST00000369519.3 | RNA binding motif protein 20 | 7 | 1 | 1 | 0 | 0 | 0 | bta-miR-425-3p | -0.32 |
| ITGA7 | ENST00000553804.1 | integrin, alpha 7 | 100 | 1 | 0 | 0 | 1 | 0 | bta-miR-425-3p | -0.32 |
| PLEKHH1 | ENST00000329153.5 | pleckstrin homology domain containing, family H (with MyTH4 domain) member 1 | 125 | 1 | 0 | 0 | 1 | 0 | bta-miR-425-3p | -0.32 |
| OSCAR | ENST00000391761.1 | osteoclast associated, immunoglobulin-like receptor | 5 | 1 | 0 | 1 | 0 | 0 | bta-miR-2403 | -0.32 |
| VPS39 | ENST00000318006.5 | vacuolar protein sorting 39 homolog (S. cerevisiae) | 165 | 1 | 1 | 0 | 0 | 0 | bta-miR-2403 | -0.32 |
| PDE3A | ENST00000359062.3 | phosphodiesterase 3A, cGMP-inhibited | 1182 | 2 | 0 | 1 | 1 | 0 | bta-miR-425-3p | -0.32 |
| HES7 | ENST00000541682.2 | hairy and enhancer of split 7 (Drosophila) | 416 | 1 | 0 | 1 | 0 | 0 | bta-miR-2403 | -0.32 |
| MPP1 | ENST00000369534.3 | membrane protein, palmitoylated 1, 55kDa | 2530 | 1 | 0 | 1 | 0 | 0 | bta-miR-2403 | -0.31 |
| PDE4D | ENST00000340635.6 | phosphodiesterase 4D, cAMP-specific | 747 | 2 | 0 | 0 | 2 | 0 | bta-miR-2403 | -0.31 |
| KCNJ10 | ENST00000368089.3 | potassium inwardly-rectifying channel, subfamily J, member 10 | 5 | 1 | 0 | 1 | 0 | 1 | bta-miR-2403 | -0.31 |
| CDH24 | ENST00000397359.3 | cadherin 24, type 2 | 44 | 1 | 0 | 0 | 1 | 0 | bta-miR-425-3p | -0.31 |
| TMEM201 | ENST00000340305.5 | transmembrane protein 201 | 164 | 1 | 0 | 0 | 1 | 0 | bta-miR-2403 | -0.31 |
| DLG2 | ENST00000398309.2 | discs, large homolog 2 (Drosophila) | 15 | 1 | 1 | 0 | 0 | 0 | bta-miR-2403 | -0.31 |
| SLC25A24 | ENST00000565488.1 | solute carrier family 25 (mitochondrial carrier; phosphate carrier), member 24 | 1688 | 1 | 0 | 1 | 0 | 0 | bta-miR-425-3p | -0.31 |
| ARFGEF2 | ENST00000371917.4 | ADP-ribosylation factor guanine nucleotide-exchange factor 2 (brefeldin A-inhibited) | 111 | 1 | 0 | 0 | 1 | 0 | bta-miR-425-3p | -0.31 |
| ZBTB21 | ENST00000398505.3 | zinc finger and BTB domain containing 21 | 802 | 1 | 0 | 0 | 1 | 0 | bta-miR-2403 | -0.31 |
| RP11-17M16.1 | ENST00000532511.1 | Homo sapiens FLJ44313 protein (FLJ44313), mRNA. | 9 | 1 | 0 | 1 | 0 | 0 | bta-miR-2403 | -0.31 |
| PRELID2 | ENST00000334744.4 | PRELI domain containing 2 | 63 | 2 | 0 | 2 | 0 | 0 | bta-miR-2403 | -0.31 |
| NEIL2 | ENST00000284503.6 | nei endonuclease VIII-like 2 (E. coli) | 59 | 1 | 0 | 0 | 1 | 0 | bta-miR-2403 | -0.31 |
| GALNTL6 | ENST00000506823.1 | UDP-N-acetyl-alpha-D-galactosamine:polypeptide N-acetylgalactosaminyltransferase-like 6 | 26 | 1 | 0 | 0 | 1 | 0 | bta-miR-425-3p | -0.3 |
| NAGPA | ENST00000312251.3 | N-acetylglucosamine-1-phosphodiester alpha-N-acetylglucosaminidase | 297 | 1 | 0 | 1 | 0 | 0 | bta-miR-2403 | -0.3 |
| C1orf200 | ENST00000377320.3 | chromosome 1 open reading frame 200 | 5 | 1 | 0 | 0 | 1 | 0 | bta-miR-2403 | -0.3 |
| ZNF213 | ENST00000396878.3 | zinc finger protein 213 | 48 | 1 | 0 | 1 | 0 | 0 | bta-miR-2403 | -0.3 |
| MTCH1 | ENST00000373616.5 | mitochondrial carrier 1 | 71 | 1 | 0 | 1 | 0 | 0 | bta-miR-2403 | -0.3 |
| FAM109B | ENST00000321753.3 | family with sequence similarity 109, member B | 78 | 1 | 0 | 1 | 0 | 0 | bta-miR-425-3p | -0.3 |
| RBFOX1 | ENST00000355637.4 | RNA binding protein, fox-1 homolog (C. elegans) 1 | 5 | 1 | 0 | 1 | 0 | 0 | bta-miR-2403 | -0.3 |
| RAB11A | ENST00000569896.1 | RAB11A, member RAS oncogene family | 5781 | 1 | 0 | 0 | 1 | 0 | bta-miR-425-3p | -0.3 |
| HSF4 | ENST00000264009.8 | heat shock transcription factor 4 | 17 | 1 | 0 | 1 | 0 | 0 | bta-miR-2403 | -0.3 |
| ATXN7L3 | ENST00000454077.2 | ataxin 7-like 3 | 43 | 1 | 0 | 0 | 1 | 1 | bta-miR-2403 | -0.3 |
| PRRC2B | ENST00000372249.1 | proline-rich coiled-coil 2B | 97 | 1 | 0 | 0 | 1 | 0 | bta-miR-425-3p | -0.3 |
| AGPAT9 | ENST00000395226.2 | 1-acylglycerol-3-phosphate O-acyltransferase 9 | 89 | 1 | 0 | 1 | 0 | 0 | bta-miR-2403 | -0.3 |
| ST3GAL5 | ENST00000393808.3 | ST3 beta-galactoside alpha-2,3-sialyltransferase 5 | 5 | 1 | 0 | 0 | 1 | 0 | bta-miR-425-3p | -0.3 |
| GATA4 | ENST00000335135.4 | GATA binding protein 4 | 5 | 1 | 0 | 0 | 1 | 0 | bta-miR-2403 | -0.3 |
| GIPC3 | ENST00000322315.5 | GIPC PDZ domain containing family, member 3 | 48 | 1 | 1 | 0 | 0 | 0 | bta-miR-2403 | -0.3 |
| BDP1 | ENST00000380675.2 | B double prime 1, subunit of RNA polymerase III transcription initiation factor IIIB | 12 | 1 | 0 | 0 | 1 | 0 | bta-miR-2403 | -0.29 |
| SLC6A1 | ENST00000287766.4 | solute carrier family 6 (neurotransmitter transporter), member 1 | 5 | 1 | 0 | 1 | 0 | 0 | bta-miR-2403 | -0.29 |
| SLX4 | ENST00000294008.3 | SLX4 structure-specific endonuclease subunit | 48 | 1 | 0 | 0 | 1 | 0 | bta-miR-425-3p | -0.29 |
| OSBPL5 | ENST00000478260.1 | oxysterol binding protein-like 5 | 215 | 1 | 0 | 1 | 0 | 0 | bta-miR-2403 | -0.29 |
| SPATA13 | ENST00000382108.3 | spermatogenesis associated 13 | 900 | 2 | 0 | 1 | 1 | 0 | bta-miR-2403 | -0.29 |
| C7orf31 | ENST00000409280.1 | chromosome 7 open reading frame 31 | 40 | 1 | 0 | 1 | 0 | 0 | bta-miR-425-3p | -0.29 |
| KAL1 | ENST00000262648.3 | Kallmann syndrome 1 sequence | 11 | 1 | 1 | 0 | 0 | 0 | bta-miR-2403 | -0.29 |
| ABCC12 | ENST00000416054.1 | ATP-binding cassette, sub-family C (CFTR/MRP), member 12 | 5 | 1 | 1 | 0 | 0 | 0 | bta-miR-425-3p | -0.29 |
| RP11-849H4.2 | ENST00000529844.1 | Putative short transient receptor potential channel 2-like protein | 5 | 1 | 0 | 1 | 0 | 0 | bta-miR-425-3p | -0.29 |
| SIDT1 | ENST00000264852.4 | SID1 transmembrane family, member 1 | 5 | 1 | 0 | 1 | 0 | 0 | bta-miR-425-3p | -0.29 |
| ZMPSTE24 | ENST00000372759.3 | zinc metallopeptidase STE24 | 513 | 1 | 0 | 0 | 1 | 0 | bta-miR-425-3p | -0.29 |
| SH2D1B | ENST00000367929.2 | SH2 domain containing 1B | 5 | 1 | 0 | 1 | 0 | 0 | bta-miR-2403 | -0.29 |
| PML | ENST00000435786.2 | promyelocytic leukemia | 95 | 1 | 0 | 0 | 1 | 0 | bta-miR-2403 | -0.29 |
| BTBD7 | ENST00000334746.5 | BTB (POZ) domain containing 7 | 590 | 1 | 1 | 0 | 0 | 0 | bta-miR-2403 | -0.29 |
| MAP3K9 | ENST00000554752.2 | mitogen-activated protein kinase kinase kinase 9 | 44 | 1 | 0 | 1 | 0 | 2 | bta-miR-425-3p | -0.29 |
| CYP4B1 | ENST00000271153.4 | cytochrome P450, family 4, subfamily B, polypeptide 1 | 5 | 1 | 0 | 1 | 0 | 0 | bta-miR-425-3p | -0.29 |
| ABCB9 | ENST00000280560.8 | ATP-binding cassette, sub-family B (MDR/TAP), member 9 | 13 | 1 | 0 | 0 | 1 | 0 | bta-miR-425-3p | -0.29 |
| KCNAB1 | ENST00000302490.8 | potassium voltage-gated channel, shaker-related subfamily, beta member 1 | 5 | 1 | 0 | 1 | 0 | 0 | bta-miR-2403 | -0.28 |
| XPNPEP2 | ENST00000371106.3 | X-prolyl aminopeptidase (aminopeptidase P) 2, membrane-bound | 5 | 1 | 0 | 1 | 0 | 0 | bta-miR-425-3p | -0.28 |
| GPRIN2 | ENST00000374314.4 | G protein regulated inducer of neurite outgrowth 2 | 5 | 1 | 0 | 0 | 1 | 1 | bta-miR-2403 | -0.28 |
| ZCCHC24 | ENST00000372336.3 | zinc finger, CCHC domain containing 24 | 359 | 1 | 0 | 0 | 1 | 0 | bta-miR-425-3p | -0.28 |
| SEC14L4 | ENST00000381982.3 | SEC14-like 4 (S. cerevisiae) | 5 | 1 | 0 | 1 | 0 | 0 | bta-miR-2403 | -0.28 |
| HAPLN2 | ENST00000255039.1 | hyaluronan and proteoglycan link protein 2 | 5 | 1 | 0 | 1 | 0 | 0 | bta-miR-425-3p | -0.28 |
| ZNF24 | ENST00000589881.1 | zinc finger protein 24 | 1796 | 1 | 0 | 0 | 1 | 0 | bta-miR-2403 | -0.28 |
| ZFP36L1 | ENST00000555997.1 | ZFP36 ring finger protein-like 1 | 231 | 1 | 0 | 1 | 0 | 0 | bta-miR-425-3p | -0.28 |
| COG3 | ENST00000349995.5 | component of oligomeric golgi complex 3 | 53 | 1 | 0 | 0 | 1 | 0 | bta-miR-425-3p | -0.28 |
| ITGB6 | ENST00000283249.2 | integrin, beta 6 | 5 | 1 | 0 | 1 | 0 | 0 | bta-miR-425-3p | -0.28 |
| MICAL3 | ENST00000441493.2 | microtubule associated monooxygenase, calponin and LIM domain containing 3 | 58 | 1 | 0 | 1 | 0 | 0 | bta-miR-2403 | -0.28 |
| C1orf123 | ENST00000294360.4 | chromosome 1 open reading frame 123 | 558 | 1 | 0 | 1 | 0 | 0 | bta-miR-2403 | -0.28 |
| AC091801.1 | ENST00000402115.1 | LOC392621; Uncharacterized protein | 5 | 1 | 0 | 1 | 0 | 0 | bta-miR-425-3p | -0.28 |
| IMPDH1 | ENST00000419067.2 | IMP (inosine 5'-monophosphate) dehydrogenase 1 | 51 | 1 | 0 | 1 | 0 | 0 | bta-miR-425-3p | -0.28 |
| OTUD4 | ENST00000454497.2 | OTU domain containing 4 | 18 | 1 | 1 | 0 | 0 | 0 | bta-miR-2403 | -0.28 |
| PARPBP | ENST00000378128.3 | PARP1 binding protein | 456 | 1 | 0 | 1 | 0 | 0 | bta-miR-2403 | -0.28 |
| KIF3C | ENST00000264712.3 | kinesin family member 3C | 177 | 1 | 1 | 0 | 0 | 0 | bta-miR-2403 | -0.28 |
| CLDN9 | ENST00000445369.2 | claudin 9 | 5 | 1 | 0 | 1 | 0 | 0 | bta-miR-425-3p | -0.28 |
| FOXN4 | ENST00000355216.1 | forkhead box N4 | 16 | 1 | 1 | 0 | 0 | 0 | bta-miR-2403 | -0.28 |
| SOX1 | ENST00000330949.1 | SRY (sex determining region Y)-box 1 | 5 | 1 | 0 | 0 | 1 | 0 | bta-miR-425-3p | -0.28 |
| DERL3 | ENST00000404056.1 | derlin 3 | 129 | 1 | 0 | 0 | 1 | 0 | bta-miR-2403 | -0.27 |
| LIN28B | ENST00000345080.4 | lin-28 homolog B (C. elegans) | 118 | 1 | 0 | 0 | 1 | 0 | bta-miR-425-3p | -0.27 |
| PRRT2 | ENST00000300797.6 | proline-rich transmembrane protein 2 | 33 | 1 | 0 | 0 | 1 | 0 | bta-miR-425-3p | -0.27 |
| AMT | ENST00000458307.2 | aminomethyltransferase | 63 | 1 | 0 | 1 | 0 | 0 | bta-miR-2403 | -0.27 |
| NIPAL3 | ENST00000003912.3 | NIPA-like domain containing 3 | 775 | 1 | 0 | 1 | 0 | 0 | bta-miR-2403 | -0.27 |
| DAND5 | ENST00000585548.1 | DAN domain family member 5, BMP antagonist | 11 | 1 | 0 | 1 | 0 | 0 | bta-miR-2403 | -0.27 |
| KCNIP1 | ENST00000328939.4 | Kv channel interacting protein 1 | 5 | 1 | 0 | 0 | 1 | 0 | bta-miR-2403 | -0.27 |
| FGF9 | ENST00000382353.5 | fibroblast growth factor 9 | 136 | 1 | 0 | 0 | 1 | 0 | bta-miR-2403 | -0.27 |
| MFAP4 | ENST00000497081.2 | microfibrillar-associated protein 4 | 67 | 1 | 0 | 0 | 1 | 0 | bta-miR-425-3p | -0.27 |
| CMPK2 | ENST00000256722.5 | cytidine monophosphate (UMP-CMP) kinase 2, mitochondrial | 13 | 1 | 0 | 0 | 1 | 0 | bta-miR-2403 | -0.27 |
| ZIC4 | ENST00000383075.3 | Zic family member 4 | 5 | 1 | 0 | 1 | 0 | 0 | bta-miR-425-3p | -0.27 |
| WNT9A | ENST00000272164.5 | wingless-type MMTV integration site family, member 9A | 73 | 1 | 0 | 0 | 1 | 0 | bta-miR-2403 | -0.27 |
| SPNS2 | ENST00000329078.3 | spinster homolog 2 (Drosophila) | 7 | 1 | 0 | 0 | 1 | 0 | bta-miR-2403 | -0.27 |
| SGK2 | ENST00000373100.1 | serum/glucocorticoid regulated kinase 2 | 26 | 1 | 0 | 1 | 0 | 0 | bta-miR-425-3p | -0.27 |
| SGSM1 | ENST00000400358.4 | small G protein signaling modulator 1 | 25 | 2 | 0 | 2 | 0 | 0 | bta-miR-2403 | -0.27 |
| TTLL11 | ENST00000373776.3 | tubulin tyrosine ligase-like family, member 11 | 164 | 1 | 0 | 0 | 1 | 0 | bta-miR-425-3p | -0.27 |
| TBPL1 | ENST00000237264.4 | TBP-like 1 | 69 | 1 | 0 | 1 | 0 | 0 | bta-miR-425-3p | -0.27 |
| COG4 | ENST00000323786.5 | component of oligomeric golgi complex 4 | 2411 | 1 | 0 | 0 | 1 | 0 | bta-miR-2403 | -0.27 |
| SRGAP3 | ENST00000383836.3 | SLIT-ROBO Rho GTPase activating protein 3 | 38 | 1 | 0 | 1 | 0 | 1 | bta-miR-2403 | -0.27 |
| PCDH1 | ENST00000503492.1 | protocadherin 1 | 30 | 1 | 0 | 1 | 0 | 0 | bta-miR-2403 | -0.27 |
| THSD7A | ENST00000423059.4 | thrombospondin, type I, domain containing 7A | 5 | 1 | 0 | 0 | 1 | 0 | bta-miR-2403 | -0.27 |
| GJB4 | ENST00000339480.1 | gap junction protein, beta 4, 30.3kDa | 5 | 1 | 0 | 1 | 0 | 0 | bta-miR-2403 | -0.27 |
| SLC25A43 | ENST00000217909.7 | solute carrier family 25, member 43 | 5 | 1 | 0 | 1 | 0 | 0 | bta-miR-425-3p | -0.27 |
| ITGB5 | ENST00000296181.4 | integrin, beta 5 | 3180 | 1 | 0 | 1 | 0 | 0 | bta-miR-2403 | -0.26 |
| GP2 | ENST00000302555.5 | glycoprotein 2 (zymogen granule membrane) | 29 | 1 | 1 | 0 | 0 | 0 | bta-miR-2403 | -0.26 |
| TESK2 | ENST00000372084.1 | testis-specific kinase 2 | 11 | 1 | 0 | 1 | 0 | 0 | bta-miR-2403 | -0.26 |
| SUSD2 | ENST00000358321.3 | sushi domain containing 2 | 16 | 1 | 0 | 0 | 1 | 0 | bta-miR-2403 | -0.26 |
| CAMK4 | ENST00000282356.4 | calcium/calmodulin-dependent protein kinase IV | 73 | 2 | 0 | 2 | 0 | 0 | bta-miR-2403 | -0.26 |
| CYB561D1 | ENST00000496961.1 | cytochrome b561 family, member D1 | 105 | 1 | 0 | 0 | 1 | 0 | bta-miR-2403 | -0.26 |
| PFKFB2 | ENST00000367080.3 | 6-phosphofructo-2-kinase/fructose-2,6-biphosphatase 2 | 83 | 1 | 0 | 0 | 1 | 0 | bta-miR-2403 | -0.26 |
| HS3ST3A1 | ENST00000284110.1 | heparan sulfate (glucosamine) 3-O-sulfotransferase 3A1 | 148 | 1 | 0 | 1 | 0 | 0 | bta-miR-425-3p | -0.26 |
| SLC24A4 | ENST00000393265.2 | solute carrier family 24 (sodium/potassium/calcium exchanger), member 4 | 5 | 1 | 0 | 1 | 0 | 1 | bta-miR-2403 | -0.26 |
| ACSL5 | ENST00000354273.4 | acyl-CoA synthetase long-chain family member 5 | 104 | 1 | 0 | 0 | 1 | 1 | bta-miR-2403 | -0.26 |
| ANK1 | ENST00000289734.7 | ankyrin 1, erythrocytic | 5 | 1 | 0 | 0 | 1 | 0 | bta-miR-2403 | -0.26 |
| NEURL1B | ENST00000369800.5 | neuralized homolog 1B (Drosophila) | 5 | 1 | 0 | 1 | 0 | 1 | bta-miR-425-3p | -0.26 |
| GPR56 | ENST00000388812.4 | G protein-coupled receptor 56 | 44 | 1 | 0 | 0 | 1 | 0 | bta-miR-2403 | -0.26 |
| CTBS | ENST00000370630.5 | chitobiase, di-N-acetyl- | 33 | 1 | 0 | 1 | 0 | 0 | bta-miR-2403 | -0.26 |
| RUNX2 | ENST00000371432.3 | runt-related transcription factor 2 | 105 | 1 | 0 | 0 | 1 | 0 | bta-miR-425-3p | -0.26 |
| TFPI | ENST00000392365.1 | tissue factor pathway inhibitor (lipoprotein-associated coagulation inhibitor) | 10747 | 1 | 0 | 0 | 1 | 0 | bta-miR-2403 | -0.25 |
| AL450307.1 | ENST00000341866.3 | Uncharacterized protein; cDNA FLJ46300 fis, clone TESTI4035989 | 5 | 1 | 0 | 1 | 0 | 0 | bta-miR-425-3p | -0.25 |
| PKD1 | ENST00000262304.4 | polycystic kidney disease 1 (autosomal dominant) | 14 | 1 | 0 | 1 | 0 | 0 | bta-miR-2403 | -0.25 |
| MAFG | ENST00000357736.4 | v-maf avian musculoaponeurotic fibrosarcoma oncogene homolog G | 5 | 1 | 0 | 1 | 0 | 1 | bta-miR-2403 | -0.25 |
| RPS6KA2 | ENST00000265678.4 | ribosomal protein S6 kinase, 90kDa, polypeptide 2 | 1043 | 1 | 0 | 0 | 1 | 1 | bta-miR-425-3p | -0.25 |
| C10orf105 | ENST00000441508.2 | chromosome 10 open reading frame 105 | 5 | 1 | 0 | 1 | 0 | 0 | bta-miR-2403 | -0.25 |
| ADAM11 | ENST00000200557.6 | ADAM metallopeptidase domain 11 | 32 | 1 | 1 | 0 | 0 | 0 | bta-miR-2403 | -0.25 |
| FAM73B | ENST00000358369.4 | family with sequence similarity 73, member B | 651 | 1 | 0 | 0 | 1 | 1 | bta-miR-2403 | -0.25 |
| AP3M2 | ENST00000174653.3 | adaptor-related protein complex 3, mu 2 subunit | 792 | 1 | 0 | 1 | 0 | 0 | bta-miR-425-3p | -0.25 |
| C21orf59 | ENST00000382549.4 | chromosome 21 open reading frame 59 | 288 | 1 | 1 | 0 | 0 | 1 | bta-miR-2403 | -0.24 |
| NSMAF | ENST00000038176.3 | neutral sphingomyelinase (N-SMase) activation associated factor | 28 | 1 | 0 | 0 | 1 | 0 | bta-miR-2403 | -0.24 |
| CTNND1 | ENST00000524630.1 | catenin (cadherin-associated protein), delta 1 | 743 | 1 | 0 | 0 | 1 | 1 | bta-miR-2403 | -0.24 |
| MCC | ENST00000302475.4 | mutated in colorectal cancers | 115 | 1 | 1 | 0 | 0 | 0 | bta-miR-425-3p | -0.24 |
| ANKRD52 | ENST00000267116.7 | ankyrin repeat domain 52 | 2913 | 1 | 1 | 0 | 0 | 1 | bta-miR-2403 | -0.24 |
| MARCH4 | ENST00000273067.4 | membrane-associated ring finger (C3HC4) 4, E3 ubiquitin protein ligase | 68 | 1 | 0 | 1 | 0 | 0 | bta-miR-2403 | -0.24 |
| EPHA4 | ENST00000281821.2 | EPH receptor A4 | 64 | 1 | 0 | 0 | 1 | 0 | bta-miR-425-3p | -0.24 |
| FBLIM1 | ENST00000375771.1 | filamin binding LIM protein 1 | 581 | 1 | 0 | 1 | 0 | 0 | bta-miR-425-3p | -0.24 |
| SPRED2 | ENST00000356388.4 | sprouty-related, EVH1 domain containing 2 | 53 | 1 | 0 | 0 | 1 | 0 | bta-miR-2403 | -0.24 |
| DUSP7 | ENST00000495880.1 | dual specificity phosphatase 7 | 68 | 1 | 0 | 0 | 1 | 0 | bta-miR-425-3p | -0.24 |
| IL8 | ENST00000307407.3 | interleukin 8 | 227 | 1 | 0 | 1 | 0 | 0 | bta-miR-2403 | -0.24 |
| GPR26 | ENST00000284674.1 | G protein-coupled receptor 26 | 5 | 1 | 0 | 0 | 1 | 0 | bta-miR-425-3p | -0.24 |
| FN1 | ENST00000357009.2 | fibronectin 1 | 1064 | 1 | 0 | 1 | 0 | 0 | bta-miR-425-3p | -0.24 |
| CCDC62 | ENST00000253079.6 | coiled-coil domain containing 62 | 5 | 1 | 0 | 0 | 1 | 0 | bta-miR-425-3p | -0.24 |
| IQCE | ENST00000402050.2 | IQ motif containing E | 687 | 1 | 0 | 1 | 0 | 0 | bta-miR-2403 | -0.24 |
| MED29 | ENST00000315588.5 | mediator complex subunit 29 | 2453 | 1 | 1 | 0 | 0 | 0 | bta-miR-425-3p | -0.24 |
| GNA11 | ENST00000078429.4 | guanine nucleotide binding protein (G protein), alpha 11 (Gq class) | 1513 | 1 | 0 | 1 | 0 | 0 | bta-miR-2403 | -0.24 |
| PNO1 | ENST00000263657.2 | partner of NOB1 homolog (S. cerevisiae) | 554 | 1 | 0 | 0 | 1 | 0 | bta-miR-425-3p | -0.23 |
| AGK | ENST00000355413.4 | acylglycerol kinase | 186 | 1 | 0 | 0 | 1 | 0 | bta-miR-2403 | -0.23 |
| PMEPA1 | ENST00000341744.3 | prostate transmembrane protein, androgen induced 1 | 736 | 1 | 1 | 0 | 0 | 0 | bta-miR-2403 | -0.23 |
| C1orf198 | ENST00000366663.5 | chromosome 1 open reading frame 198 | 1484 | 1 | 0 | 1 | 0 | 0 | bta-miR-2403 | -0.23 |
| EPHA1 | ENST00000275815.3 | EPH receptor A1 | 13 | 1 | 0 | 1 | 0 | 0 | bta-miR-2403 | -0.23 |
| RSPO4 | ENST00000217260.4 | R-spondin 4 | 5 | 1 | 0 | 1 | 0 | 0 | bta-miR-2403 | -0.23 |
| FOXJ3 | ENST00000372571.1 | forkhead box J3 | 329 | 1 | 0 | 1 | 0 | 0 | bta-miR-425-3p | -0.23 |
| RTFDC1 | ENST00000395881.3 | replication termination factor 2 domain containing 1 | 15783 | 1 | 0 | 1 | 0 | 0 | bta-miR-425-3p | -0.23 |
| TLR6 | ENST00000436693.2 | toll-like receptor 6 | 5 | 1 | 0 | 1 | 0 | 0 | bta-miR-2403 | -0.23 |
| SPHK2 | ENST00000245222.4 | sphingosine kinase 2 | 142 | 1 | 0 | 0 | 1 | 0 | bta-miR-2403 | -0.23 |
| MFAP2 | ENST00000375534.3 | microfibrillar-associated protein 2 | 10 | 1 | 0 | 0 | 1 | 0 | bta-miR-2403 | -0.23 |
| KIAA0430 | ENST00000396368.3 | KIAA0430 | 461 | 1 | 1 | 0 | 0 | 0 | bta-miR-425-3p | -0.23 |
| AC011484.1 | ENST00000377652.3 | Uncharacterized protein; cDNA FLJ42076 fis, clone SYNOV2018921 | 5 | 1 | 0 | 1 | 0 | 0 | bta-miR-2403 | -0.23 |
| CHMP6 | ENST00000325167.5 | charged multivesicular body protein 6 | 227 | 1 | 0 | 1 | 0 | 0 | bta-miR-2403 | -0.22 |
| C1orf172 | ENST00000320567.5 | chromosome 1 open reading frame 172 | 190 | 1 | 0 | 1 | 0 | 0 | bta-miR-2403 | -0.22 |
| METTL8 | ENST00000375258.4 | methyltransferase like 8 | 124 | 1 | 0 | 0 | 1 | 0 | bta-miR-425-3p | -0.22 |
| DIEXF | ENST00000491415.2 | digestive organ expansion factor homolog (zebrafish) | 493 | 1 | 0 | 0 | 1 | 0 | bta-miR-425-3p | -0.22 |
| ESPN | ENST00000377828.1 | espin | 513 | 1 | 0 | 1 | 0 | 0 | bta-miR-2403 | -0.22 |
| DLX3 | ENST00000434704.2 | distal-less homeobox 3 | 5 | 1 | 0 | 1 | 0 | 0 | bta-miR-2403 | -0.22 |
| CHST10 | ENST00000264249.3 | carbohydrate sulfotransferase 10 | 33 | 1 | 0 | 1 | 0 | 0 | bta-miR-2403 | -0.22 |
| BCL2 | ENST00000398117.1 | B-cell CLL/lymphoma 2 | 55 | 1 | 0 | 0 | 1 | 0 | bta-miR-425-3p | -0.22 |
| ZFYVE20 | ENST00000253699.3 | zinc finger, FYVE domain containing 20 | 57 | 1 | 1 | 0 | 0 | 0 | bta-miR-2403 | -0.22 |
| GATS | ENST00000436886.2 | GATS, stromal antigen 3 opposite strand | 86 | 1 | 0 | 1 | 0 | 0 | bta-miR-2403 | -0.22 |
| FAM212B | ENST00000357260.5 | family with sequence similarity 212, member B | 211 | 1 | 0 | 0 | 1 | 0 | bta-miR-2403 | -0.22 |
| POFUT1 | ENST00000375749.3 | protein O-fucosyltransferase 1 | 647 | 1 | 0 | 0 | 1 | 0 | bta-miR-425-3p | -0.22 |
| FAM227A | ENST00000535113.1 | family with sequence similarity 227, member A | 16 | 1 | 1 | 0 | 0 | 0 | bta-miR-2403 | -0.22 |
| GRAMD4 | ENST00000361034.3 | GRAM domain containing 4 | 403 | 1 | 0 | 0 | 1 | 0 | bta-miR-425-3p | -0.22 |
| C17orf72 | ENST00000412177.1 | chromosome 17 open reading frame 72 | 5 | 1 | 0 | 1 | 0 | 0 | bta-miR-2403 | -0.22 |
| HOXD3 | ENST00000249440.3 | homeobox D3 | 23 | 1 | 0 | 0 | 1 | 0 | bta-miR-425-3p | -0.22 |
| ELMSAN1 | ENST00000394071.2 | ELM2 and Myb/SANT-like domain containing 1 | 15 | 1 | 0 | 1 | 0 | 0 | bta-miR-425-3p | -0.22 |
| ASAP1 | ENST00000357668.1 | ArfGAP with SH3 domain, ankyrin repeat and PH domain 1 | 185 | 1 | 0 | 0 | 1 | 0 | bta-miR-425-3p | -0.22 |
| ZNF398 | ENST00000420008.2 | zinc finger protein 398 | 305 | 1 | 0 | 1 | 0 | 1 | bta-miR-425-3p | -0.22 |
| C15orf27 | ENST00000388942.3 | chromosome 15 open reading frame 27 | 17 | 1 | 0 | 1 | 0 | 0 | bta-miR-2403 | -0.21 |
| XCR1 | ENST00000309285.3 | chemokine (C motif) receptor 1 | 5 | 1 | 0 | 1 | 0 | 0 | bta-miR-2403 | -0.21 |
| COL8A2 | ENST00000303143.4 | collagen, type VIII, alpha 2 | 15 | 1 | 0 | 0 | 1 | 0 | bta-miR-2403 | -0.21 |
| TANGO2 | ENST00000434570.2 | transport and golgi organization 2 homolog (Drosophila) | 119 | 1 | 1 | 0 | 0 | 0 | bta-miR-2403 | -0.21 |
| PER3 | ENST00000377532.3 | period circadian clock 3 | 16 | 1 | 0 | 1 | 0 | 0 | bta-miR-425-3p | -0.21 |
| AKAP12 | ENST00000402676.2 | A kinase (PRKA) anchor protein 12 | 913 | 1 | 0 | 1 | 0 | 0 | bta-miR-2403 | -0.21 |
| KREMEN1 | ENST00000400335.4 | kringle containing transmembrane protein 1 | 606 | 1 | 0 | 1 | 0 | 0 | bta-miR-425-3p | -0.21 |
| LIPH | ENST00000296252.4 | lipase, member H | 18 | 1 | 0 | 1 | 0 | 0 | bta-miR-2403 | -0.21 |
| FOXI2 | ENST00000388920.4 | forkhead box I2 | 5 | 1 | 0 | 1 | 0 | 0 | bta-miR-2403 | -0.21 |
| MN1 | ENST00000302326.4 | meningioma (disrupted in balanced translocation) 1 | 15 | 2 | 0 | 2 | 0 | 0 | bta-miR-2403 | -0.21 |
| WIZ | ENST00000389282.4 | widely interspaced zinc finger motifs | 1321 | 1 | 0 | 1 | 0 | 0 | bta-miR-2403 | -0.21 |
| CCND2 | ENST00000261254.3 | cyclin D2 | 66 | 1 | 0 | 1 | 0 | 1 | bta-miR-425-3p | -0.21 |
| KCNS1 | ENST00000306117.1 | potassium voltage-gated channel, delayed-rectifier, subfamily S, member 1 | 5 | 1 | 0 | 1 | 0 | 0 | bta-miR-425-3p | -0.21 |
| SIK2 | ENST00000304987.3 | salt-inducible kinase 2 | 378 | 2 | 0 | 1 | 1 | 0 | bta-miR-425-3p | -0.21 |
| SCN2B | ENST00000278947.5 | sodium channel, voltage-gated, type II, beta subunit | 5 | 1 | 0 | 1 | 0 | 0 | bta-miR-425-3p | -0.21 |
| DCT | ENST00000377028.5 | dopachrome tautomerase | 5 | 1 | 0 | 1 | 0 | 0 | bta-miR-425-3p | -0.21 |
| KCNJ12 | ENST00000583088.1 | potassium inwardly-rectifying channel, subfamily J, member 12 | 9 | 1 | 0 | 1 | 0 | 0 | bta-miR-2403 | -0.21 |
| PTCH1 | ENST00000430669.2 | patched 1 | 123 | 1 | 0 | 0 | 1 | 0 | bta-miR-2403 | -0.21 |
| FOXN3 | ENST00000345097.4 | forkhead box N3 | 42 | 1 | 0 | 0 | 1 | 0 | bta-miR-2403 | -0.21 |
| TUB | ENST00000305253.4 | tubby bipartite transcription factor | 743 | 1 | 0 | 1 | 0 | 0 | bta-miR-2403 | -0.21 |

**Predicted target mRNAs of mir-2462**

| Ortholog of target gene | Representative transcript | Gene name | 3P-seq tags + 5 | Total sites | 8mer sites | 7mer-m8 sites | 7mer-A1 sites | 6mer sites | Representative miRNA | Cumulative weighted context++ score |
| --- | --- | --- | --- | --- | --- | --- | --- | --- | --- | --- |
| AC079612.1 | ENST00000358775.1 | Uncharacterized protein; cDNA FLJ45964 fis, clone PLACE7014396 | 5 | 2 | 0 | 1 | 1 | 0 | bta-miR-2462 | -0.98 |
| CSN2 | ENST00000353151.3 | casein beta | 5 | 2 | 1 | 1 | 0 | 0 | bta-miR-2462 | -0.88 |
| CYP3A5 | ENST00000339843.2 | cytochrome P450, family 3, subfamily A, polypeptide 5 | 5 | 1 | 1 | 0 | 0 | 0 | bta-miR-2462 | -0.86 |
| CDR1as | CDR1as | circular RNA CDR1as | 1 | 1 | 0 | 1 | 0 | 0 | bta-miR-2462 | -0.84 |
| KPNA1 | ENST00000344337.6 | karyopherin alpha 1 (importin alpha 5) | 1030 | 3 | 2 | 0 | 1 | 1 | bta-miR-2462 | -0.8 |
| ICAM3 | ENST00000160262.5 | intercellular adhesion molecule 3 | 873 | 1 | 1 | 0 | 0 | 0 | bta-miR-2462 | -0.79 |
| C20orf85 | ENST00000371168.3 | chromosome 20 open reading frame 85 | 5 | 1 | 1 | 0 | 0 | 0 | bta-miR-2462 | -0.77 |
| LUZP2 | ENST00000533227.1 | leucine zipper protein 2 | 33 | 4 | 1 | 1 | 2 | 0 | bta-miR-2462 | -0.73 |
| TRA2A | ENST00000297071.4 | transformer 2 alpha homolog (Drosophila) | 1097 | 2 | 1 | 1 | 0 | 0 | bta-miR-2462 | -0.72 |
| PLEKHH3 | ENST00000591022.1 | pleckstrin homology domain containing, family H (with MyTH4 domain) member 3 | 5 | 1 | 1 | 0 | 0 | 0 | bta-miR-2462 | -0.66 |
| RP11-67H2.1 | ENST00000521500.2 | Uncharacterized protein | 5 | 2 | 0 | 2 | 0 | 0 | bta-miR-2462 | -0.64 |
| LYSMD3 | ENST00000509384.1 | LysM, putative peptidoglycan-binding, domain containing 3 | 92 | 4 | 1 | 1 | 2 | 1 | bta-miR-2462 | -0.64 |
| C10orf11 | ENST00000496424.2 | chromosome 10 open reading frame 11 | 5 | 1 | 1 | 0 | 0 | 4 | bta-miR-2462 | -0.62 |
| NEK7 | ENST00000367385.4 | NIMA-related kinase 7 | 12 | 2 | 2 | 0 | 0 | 0 | bta-miR-2462 | -0.61 |
| HORMAD1 | ENST00000368995.4 | HORMA domain containing 1 | 153 | 2 | 1 | 0 | 1 | 0 | bta-miR-2462 | -0.6 |
| GRP | ENST00000529320.2 | gastrin-releasing peptide | 5 | 1 | 1 | 0 | 0 | 0 | bta-miR-2462 | -0.6 |
| FAM221A | ENST00000344962.4 | family with sequence similarity 221, member A | 27 | 1 | 1 | 0 | 0 | 0 | bta-miR-2462 | -0.6 |
| SLC2A2 | ENST00000314251.3 | solute carrier family 2 (facilitated glucose transporter), member 2 | 5 | 3 | 1 | 2 | 0 | 0 | bta-miR-2462 | -0.6 |
| PPHLN1 | ENST00000449194.2 | periphilin 1 | 1823 | 1 | 1 | 0 | 0 | 0 | bta-miR-2462 | -0.59 |
| ZNF83 | ENST00000594682.2 | zinc finger protein 83 | 78 | 5 | 0 | 5 | 0 | 1 | bta-miR-2462 | -0.58 |
| GLTP | ENST00000318348.4 | glycolipid transfer protein | 5 | 1 | 1 | 0 | 0 | 0 | bta-miR-2462 | -0.58 |
| PEX2 | ENST00000357039.4 | peroxisomal biogenesis factor 2 | 662 | 1 | 1 | 0 | 0 | 0 | bta-miR-2462 | -0.56 |
| C8orf34 | ENST00000337103.4 | chromosome 8 open reading frame 34 | 38 | 1 | 1 | 0 | 0 | 0 | bta-miR-2462 | -0.56 |
| AC011294.3 | ENST00000451905.1 | Uncharacterized protein | 5 | 1 | 1 | 0 | 0 | 0 | bta-miR-2462 | -0.56 |
| UEVLD | ENST00000396197.3 | UEV and lactate/malate dehyrogenase domains | 39 | 2 | 1 | 0 | 1 | 1 | bta-miR-2462 | -0.54 |
| ZNF302 | ENST00000505365.2 | zinc finger protein 302 | 13 | 3 | 1 | 2 | 0 | 1 | bta-miR-2462 | -0.53 |
| CLEC6A | ENST00000382073.3 | C-type lectin domain family 6, member A | 5 | 1 | 1 | 0 | 0 | 0 | bta-miR-2462 | -0.53 |
| C7orf55-LUC7L2 | ENST00000354926.4 | C7orf55-LUC7L2 readthrough | 419 | 2 | 1 | 1 | 0 | 1 | bta-miR-2462 | -0.53 |
| C6orf120 | ENST00000332290.2 | chromosome 6 open reading frame 120 | 1415 | 2 | 1 | 1 | 0 | 0 | bta-miR-2462 | -0.53 |
| LUC7L2 | ENST00000541515.3 | LUC7-like 2 (S. cerevisiae) | 419 | 2 | 1 | 1 | 0 | 1 | bta-miR-2462 | -0.53 |
| PKD2L1 | ENST00000353274.3 | polycystic kidney disease 2-like 1 | 7 | 1 | 1 | 0 | 0 | 0 | bta-miR-2462 | -0.53 |
| INHBB | ENST00000295228.3 | inhibin, beta B | 75 | 3 | 0 | 1 | 2 | 0 | bta-miR-2462 | -0.52 |
| AICDA | ENST00000229335.6 | activation-induced cytidine deaminase | 5 | 1 | 1 | 0 | 0 | 1 | bta-miR-2462 | -0.52 |
| FAM199X | ENST00000493442.1 | family with sequence similarity 199, X-linked | 856 | 6 | 2 | 1 | 3 | 0 | bta-miR-2462 | -0.51 |
| AC012360.2 | ENST00000595531.1 | LOC644617 protein; Uncharacterized protein | 430 | 2 | 0 | 1 | 1 | 1 | bta-miR-2462 | -0.51 |
| RP11-192H23.4 | ENST00000534850.1 | Uncharacterized protein | 22 | 2 | 0 | 1 | 1 | 0 | bta-miR-2462 | -0.5 |
| PPP2R3C | ENST00000261475.5 | protein phosphatase 2, regulatory subunit B'', gamma | 105 | 1 | 1 | 0 | 0 | 0 | bta-miR-2462 | -0.5 |
| TIMM17A | ENST00000367287.4 | translocase of inner mitochondrial membrane 17 homolog A (yeast) | 5594 | 1 | 1 | 0 | 0 | 0 | bta-miR-2462 | -0.5 |
| LETMD1 | ENST00000380123.2 | LETM1 domain containing 1 | 1619 | 4 | 1 | 3 | 0 | 0 | bta-miR-2462 | -0.5 |
| CLDN14 | ENST00000399139.1 | claudin 14 | 121 | 1 | 1 | 0 | 0 | 0 | bta-miR-2462 | -0.49 |
| WNK3 | ENST00000375169.3 | WNK lysine deficient protein kinase 3 | 55 | 3 | 0 | 3 | 0 | 4 | bta-miR-2462 | -0.49 |
| LL22NC03-63E9.3 | ENST00000407120.1 | Uncharacterized protein | 5 | 1 | 0 | 1 | 0 | 0 | bta-miR-2462 | -0.49 |
| CXCL11 | ENST00000306621.3 | chemokine (C-X-C motif) ligand 11 | 5 | 1 | 1 | 0 | 0 | 0 | bta-miR-2462 | -0.49 |
| ZCCHC6 | ENST00000277141.6 | zinc finger, CCHC domain containing 6 | 20 | 3 | 0 | 3 | 0 | 0 | bta-miR-2462 | -0.48 |
| CXCL9 | ENST00000264888.5 | chemokine (C-X-C motif) ligand 9 | 5 | 1 | 0 | 0 | 1 | 2 | bta-miR-2462 | -0.48 |
| SELL | ENST00000236147.4 | selectin L | 5 | 1 | 1 | 0 | 0 | 2 | bta-miR-2462 | -0.47 |
| SGK494 | ENST00000301037.5 | uncharacterized serine/threonine-protein kinase SgK494 | 17 | 2 | 0 | 1 | 1 | 0 | bta-miR-2462 | -0.47 |
| GDI1 | ENST00000447750.2 | GDP dissociation inhibitor 1 | 21 | 2 | 2 | 0 | 0 | 0 | bta-miR-2462 | -0.47 |
| GOLT1B | ENST00000540141.1 | golgi transport 1B | 848 | 3 | 0 | 1 | 2 | 1 | bta-miR-2462 | -0.46 |
| SMYD3 | ENST00000541742.1 | SET and MYND domain containing 3 | 508 | 1 | 1 | 0 | 0 | 0 | bta-miR-2462 | -0.46 |
| PANK3 | ENST00000239231.6 | pantothenate kinase 3 | 522 | 3 | 1 | 2 | 0 | 1 | bta-miR-2462 | -0.46 |
| FAM78B | ENST00000354422.3 | family with sequence similarity 78, member B | 9 | 1 | 1 | 0 | 0 | 0 | bta-miR-2462 | -0.45 |
| ZNF611 | ENST00000543227.1 | zinc finger protein 611 | 7 | 1 | 1 | 0 | 0 | 0 | bta-miR-2462 | -0.45 |
| TMEM174 | ENST00000296776.5 | transmembrane protein 174 | 5 | 1 | 1 | 0 | 0 | 0 | bta-miR-2462 | -0.44 |
| ARHGAP36 | ENST00000370922.1 | Rho GTPase activating protein 36 | 5 | 1 | 1 | 0 | 0 | 0 | bta-miR-2462 | -0.44 |
| ARL6 | ENST00000463745.1 | ADP-ribosylation factor-like 6 | 275 | 2 | 1 | 0 | 1 | 0 | bta-miR-2462 | -0.44 |
| DHX40 | ENST00000251241.4 | DEAH (Asp-Glu-Ala-His) box polypeptide 40 | 538 | 1 | 1 | 0 | 0 | 0 | bta-miR-2462 | -0.44 |
| GFPT1 | ENST00000357308.4 | glutamine--fructose-6-phosphate transaminase 1 | 1313 | 3 | 1 | 1 | 1 | 2 | bta-miR-2462 | -0.44 |
| C1orf101 | ENST00000366534.4 | chromosome 1 open reading frame 101 | 5 | 1 | 1 | 0 | 0 | 0 | bta-miR-2462 | -0.43 |
| SPRY3 | ENST00000302805.2 | sprouty homolog 3 (Drosophila) | 5 | 5 | 0 | 3 | 2 | 0 | bta-miR-2462 | -0.43 |
| CSTF3 | ENST00000323959.4 | cleavage stimulation factor, 3' pre-RNA, subunit 3, 77kDa | 291 | 1 | 0 | 1 | 0 | 1 | bta-miR-2462 | -0.42 |
| RIPPLY2 | ENST00000369689.1 | ripply transcriptional repressor 2 | 14 | 1 | 0 | 1 | 0 | 0 | bta-miR-2462 | -0.42 |
| RNASE9 | ENST00000338904.3 | ribonuclease, RNase A family, 9 (non-active) | 5 | 2 | 0 | 0 | 2 | 0 | bta-miR-2462 | -0.42 |
| ADRB3 | ENST00000345060.3 | adrenoceptor beta 3 | 5 | 3 | 1 | 2 | 0 | 1 | bta-miR-2462 | -0.42 |
| SPRED1 | ENST00000299084.4 | sprouty-related, EVH1 domain containing 1 | 547 | 2 | 1 | 1 | 0 | 1 | bta-miR-2462 | -0.42 |
| FKTN | ENST00000223528.2 | fukutin | 153 | 2 | 1 | 1 | 0 | 0 | bta-miR-2462 | -0.41 |
| DDX4 | ENST00000505374.1 | DEAD (Asp-Glu-Ala-Asp) box polypeptide 4 | 5 | 1 | 1 | 0 | 0 | 1 | bta-miR-2462 | -0.41 |
| TMEM159 | ENST00000572258.1 | transmembrane protein 159 | 159 | 1 | 0 | 1 | 0 | 2 | bta-miR-2462 | -0.41 |
| C1orf95 | ENST00000366788.3 | chromosome 1 open reading frame 95 | 28 | 2 | 1 | 1 | 0 | 1 | bta-miR-2462 | -0.41 |
| KCNJ3 | ENST00000295101.2 | potassium inwardly-rectifying channel, subfamily J, member 3 | 21 | 3 | 1 | 2 | 0 | 2 | bta-miR-2462 | -0.41 |
| MDM4 | ENST00000391947.2 | Mdm4 p53 binding protein homolog (mouse) | 443 | 1 | 1 | 0 | 0 | 4 | bta-miR-2462 | -0.41 |
| GPX8 | ENST00000296734.6 | glutathione peroxidase 8 (putative) | 2107 | 1 | 1 | 0 | 0 | 2 | bta-miR-2462 | -0.4 |
| TM9SF2 | ENST00000376387.4 | transmembrane 9 superfamily member 2 | 5046 | 1 | 1 | 0 | 0 | 0 | bta-miR-2462 | -0.4 |
| B4GALT4 | ENST00000467604.1 | UDP-Gal:betaGlcNAc beta 1,4- galactosyltransferase, polypeptide 4 | 196 | 2 | 0 | 1 | 1 | 0 | bta-miR-2462 | -0.4 |
| RNASE1 | ENST00000397967.4 | ribonuclease, RNase A family, 1 (pancreatic) | 5 | 1 | 1 | 0 | 0 | 0 | bta-miR-2462 | -0.4 |
| DDO | ENST00000368924.3 | D-aspartate oxidase | 5 | 1 | 1 | 0 | 0 | 0 | bta-miR-2462 | -0.4 |
| SRSF6 | ENST00000244020.3 | serine/arginine-rich splicing factor 6 | 1914 | 2 | 1 | 0 | 1 | 2 | bta-miR-2462 | -0.4 |
| TMEM68 | ENST00000523073.1 | transmembrane protein 68 | 77 | 2 | 0 | 1 | 1 | 0 | bta-miR-2462 | -0.39 |
| ESCO2 | ENST00000305188.8 | establishment of sister chromatid cohesion N-acetyltransferase 2 | 157 | 1 | 1 | 0 | 0 | 0 | bta-miR-2462 | -0.39 |
| AHCYL1 | ENST00000369799.5 | adenosylhomocysteinase-like 1 | 1279 | 2 | 0 | 2 | 0 | 3 | bta-miR-2462 | -0.39 |
| MUT | ENST00000274813.3 | methylmalonyl CoA mutase | 49 | 1 | 1 | 0 | 0 | 0 | bta-miR-2462 | -0.39 |
| DKK1 | ENST00000373970.3 | dickkopf WNT signaling pathway inhibitor 1 | 2384 | 1 | 1 | 0 | 0 | 0 | bta-miR-2462 | -0.39 |
| PKIA | ENST00000396418.2 | protein kinase (cAMP-dependent, catalytic) inhibitor alpha | 542 | 3 | 1 | 0 | 2 | 2 | bta-miR-2462 | -0.39 |
| SIGLECL1 | ENST00000316401.7 | SIGLEC family like 1 | 5 | 1 | 1 | 0 | 0 | 1 | bta-miR-2462 | -0.39 |
| ALMS1 | ENST00000264448.6 | Alstrom syndrome 1 | 282 | 1 | 1 | 0 | 0 | 0 | bta-miR-2462 | -0.39 |
| METTL15 | ENST00000342303.5 | methyltransferase like 15 | 17 | 3 | 0 | 3 | 0 | 1 | bta-miR-2462 | -0.39 |
| TMEM154 | ENST00000304385.3 | transmembrane protein 154 | 5 | 2 | 1 | 1 | 0 | 0 | bta-miR-2462 | -0.38 |
| AC104472.1 | ENST00000399242.2 | CDNA FLJ26134 fis, clone TMS03713; Uncharacterized protein | 5 | 1 | 0 | 1 | 0 | 1 | bta-miR-2462 | -0.38 |
| KIAA1191 | ENST00000298569.4 | KIAA1191 | 23 | 1 | 1 | 0 | 0 | 0 | bta-miR-2462 | -0.38 |
| INSL5 | ENST00000304526.2 | insulin-like 5 | 5 | 1 | 0 | 1 | 0 | 0 | bta-miR-2462 | -0.38 |
| FSTL5 | ENST00000306100.5 | follistatin-like 5 | 5 | 1 | 1 | 0 | 0 | 0 | bta-miR-2462 | -0.38 |
| IL7 | ENST00000263851.4 | interleukin 7 | 5 | 1 | 1 | 0 | 0 | 0 | bta-miR-2462 | -0.38 |
| PITHD1 | ENST00000246151.4 | PITH (C-terminal proteasome-interacting domain of thioredoxin-like) domain containing 1 | 718 | 2 | 0 | 1 | 1 | 0 | bta-miR-2462 | -0.38 |
| NIPAL3 | ENST00000003912.3 | NIPA-like domain containing 3 | 775 | 1 | 1 | 0 | 0 | 0 | bta-miR-2462 | -0.38 |
| XKR4 | ENST00000327381.6 | XK, Kell blood group complex subunit-related family, member 4 | 5 | 4 | 0 | 3 | 1 | 9 | bta-miR-2462 | -0.38 |
| ZNF417 | ENST00000312026.5 | zinc finger protein 417 | 19 | 1 | 0 | 1 | 0 | 0 | bta-miR-2462 | -0.38 |
| CCDC160 | ENST00000517294.1 | coiled-coil domain containing 160 | 12 | 2 | 0 | 1 | 1 | 0 | bta-miR-2462 | -0.37 |
| GJB6 | ENST00000241124.6 | gap junction protein, beta 6, 30kDa | 5 | 1 | 1 | 0 | 0 | 0 | bta-miR-2462 | -0.37 |
| CCDC176 | ENST00000394009.3 | coiled-coil domain containing 176 | 61 | 2 | 0 | 2 | 0 | 1 | bta-miR-2462 | -0.37 |
| NBPF8 | ENST00000369372.4 | neuroblastoma breakpoint family, member 8 | 5 | 1 | 0 | 0 | 1 | 0 | bta-miR-2462 | -0.37 |
| RBM17 | ENST00000446108.1 | RNA binding motif protein 17 | 449 | 1 | 0 | 1 | 0 | 1 | bta-miR-2462 | -0.37 |
| MAGEB2 | ENST00000378988.4 | melanoma antigen family B, 2 | 255 | 1 | 1 | 0 | 0 | 0 | bta-miR-2462 | -0.37 |
| TBPL1 | ENST00000237264.4 | TBP-like 1 | 69 | 1 | 1 | 0 | 0 | 0 | bta-miR-2462 | -0.37 |
| FOXN2 | ENST00000340553.3 | forkhead box N2 | 441 | 2 | 0 | 1 | 1 | 1 | bta-miR-2462 | -0.37 |
| OR2D3 | ENST00000317834.3 | olfactory receptor, family 2, subfamily D, member 3 | 5 | 1 | 0 | 0 | 1 | 0 | bta-miR-2462 | -0.37 |
| ZNF677 | ENST00000599012.1 | zinc finger protein 677 | 51 | 1 | 0 | 1 | 0 | 0 | bta-miR-2462 | -0.36 |
| AL590452.1 | ENST00000596396.1 | Uncharacterized protein | 5 | 1 | 0 | 1 | 0 | 1 | bta-miR-2462 | -0.36 |
| STK3 | ENST00000419617.2 | serine/threonine kinase 3 | 25 | 1 | 1 | 0 | 0 | 1 | bta-miR-2462 | -0.36 |
| SUMO1 | ENST00000392246.2 | small ubiquitin-like modifier 1 | 180 | 1 | 0 | 1 | 0 | 0 | bta-miR-2462 | -0.36 |
| ENY2 | ENST00000521688.1 | enhancer of yellow 2 homolog (Drosophila) | 1814 | 1 | 0 | 1 | 0 | 0 | bta-miR-2462 | -0.36 |
| C1orf110 | ENST00000367912.2 | chromosome 1 open reading frame 110 | 5 | 1 | 1 | 0 | 0 | 0 | bta-miR-2462 | -0.36 |
| ANKRD49 | ENST00000544253.1 | ankyrin repeat domain 49 | 32 | 1 | 1 | 0 | 0 | 1 | bta-miR-2462 | -0.36 |
| GALNTL6 | ENST00000506823.1 | UDP-N-acetyl-alpha-D-galactosamine:polypeptide N-acetylgalactosaminyltransferase-like 6 | 26 | 1 | 1 | 0 | 0 | 0 | bta-miR-2462 | -0.36 |
| CTC-241N9.1 | ENST00000499601.2 | Uncharacterized protein | 41 | 1 | 0 | 1 | 0 | 0 | bta-miR-2462 | -0.36 |
| SEPT14 | ENST00000388975.3 | septin 14 | 5 | 1 | 1 | 0 | 0 | 0 | bta-miR-2462 | -0.36 |
| IFNA21 | ENST00000380225.1 | interferon, alpha 21 | 5 | 1 | 0 | 1 | 0 | 0 | bta-miR-2462 | -0.36 |
| BTG2 | ENST00000290551.4 | BTG family, member 2 | 758 | 1 | 1 | 0 | 0 | 0 | bta-miR-2462 | -0.36 |
| AKR1E2 | ENST00000298375.7 | aldo-keto reductase family 1, member E2 | 5 | 1 | 0 | 1 | 0 | 0 | bta-miR-2462 | -0.35 |
| GBE1 | ENST00000429644.2 | glucan (1,4-alpha-), branching enzyme 1 | 652 | 1 | 0 | 1 | 0 | 1 | bta-miR-2462 | -0.35 |
| SCGB1D2 | ENST00000244926.3 | secretoglobin, family 1D, member 2 | 5 | 1 | 0 | 1 | 0 | 0 | bta-miR-2462 | -0.35 |
| SCGB1D1 | ENST00000306238.3 | secretoglobin, family 1D, member 1 | 5 | 1 | 0 | 1 | 0 | 0 | bta-miR-2462 | -0.35 |
| SMIM10 | ENST00000330288.4 | small integral membrane protein 10 | 124 | 1 | 0 | 1 | 0 | 0 | bta-miR-2462 | -0.35 |
| CNOT6 | ENST00000393356.1 | CCR4-NOT transcription complex, subunit 6 | 300 | 2 | 1 | 1 | 0 | 1 | bta-miR-2462 | -0.35 |
| APBB2 | ENST00000295974.8 | amyloid beta (A4) precursor protein-binding, family B, member 2 | 704 | 4 | 1 | 1 | 2 | 1 | bta-miR-2462 | -0.35 |
| NUDT4 | ENST00000337179.5 | nudix (nucleoside diphosphate linked moiety X)-type motif 4 | 41 | 1 | 1 | 0 | 0 | 1 | bta-miR-2462 | -0.35 |
| ZNF705G | ENST00000400156.4 | zinc finger protein 705G | 5 | 3 | 0 | 2 | 1 | 0 | bta-miR-2462 | -0.35 |
| OR51E1 | ENST00000396952.5 | olfactory receptor, family 51, subfamily E, member 1 | 5 | 1 | 1 | 0 | 0 | 1 | bta-miR-2462 | -0.35 |
| ZNRD1 | ENST00000332435.5 | zinc ribbon domain containing 1 | 442 | 1 | 0 | 1 | 0 | 0 | bta-miR-2462 | -0.35 |
| MGST1 | ENST00000540056.1 | microsomal glutathione S-transferase 1 | 397 | 1 | 0 | 1 | 0 | 0 | bta-miR-2462 | -0.35 |
| GHRH | ENST00000373614.2 | growth hormone releasing hormone | 5 | 1 | 0 | 1 | 0 | 0 | bta-miR-2462 | -0.35 |
| NID2 | ENST00000216286.5 | nidogen 2 (osteonidogen) | 4292 | 1 | 1 | 0 | 0 | 0 | bta-miR-2462 | -0.34 |
| MYO5A | ENST00000399231.3 | myosin VA (heavy chain 12, myoxin) | 573 | 1 | 1 | 0 | 0 | 3 | bta-miR-2462 | -0.34 |
| PCBP2 | ENST00000455667.3 | poly(rC) binding protein 2 | 24310 | 2 | 0 | 2 | 0 | 1 | bta-miR-2462 | -0.34 |
| C12orf75 | ENST00000443585.1 | chromosome 12 open reading frame 75 | 6680 | 1 | 0 | 1 | 0 | 0 | bta-miR-2462 | -0.34 |
| SUCNR1 | ENST00000362032.5 | succinate receptor 1 | 5 | 2 | 1 | 0 | 1 | 0 | bta-miR-2462 | -0.34 |
| UST | ENST00000367463.4 | uronyl-2-sulfotransferase | 933 | 2 | 2 | 0 | 0 | 1 | bta-miR-2462 | -0.34 |
| SRSF11 | ENST00000370950.3 | serine/arginine-rich splicing factor 11 | 1201 | 2 | 0 | 2 | 0 | 0 | bta-miR-2462 | -0.34 |
| RFTN2 | ENST00000295049.4 | raftlin family member 2 | 21 | 1 | 1 | 0 | 0 | 1 | bta-miR-2462 | -0.34 |
| ZFAND6 | ENST00000261749.6 | zinc finger, AN1-type domain 6 | 11 | 1 | 0 | 1 | 0 | 1 | bta-miR-2462 | -0.34 |
| E2F8 | ENST00000527884.1 | E2F transcription factor 8 | 66 | 1 | 1 | 0 | 0 | 0 | bta-miR-2462 | -0.34 |
| LANCL1 | ENST00000443314.1 | LanC lantibiotic synthetase component C-like 1 (bacterial) | 3395 | 1 | 1 | 0 | 0 | 0 | bta-miR-2462 | -0.34 |
| APEX1 | ENST00000557054.1 | APEX nuclease (multifunctional DNA repair enzyme) 1 | 15798 | 1 | 0 | 1 | 0 | 1 | bta-miR-2462 | -0.34 |
| AC004899.1 | ENST00000596947.1 | Uncharacterized protein | 5 | 1 | 0 | 1 | 0 | 0 | bta-miR-2462 | -0.34 |
| ZNF846 | ENST00000586293.1 | zinc finger protein 846 | 10 | 2 | 0 | 2 | 0 | 0 | bta-miR-2462 | -0.34 |
| LECT1 | ENST00000448904.2 | leukocyte cell derived chemotaxin 1 | 5 | 1 | 0 | 1 | 0 | 0 | bta-miR-2462 | -0.34 |
| KLHL6 | ENST00000341319.3 | kelch-like family member 6 | 5 | 3 | 1 | 1 | 1 | 1 | bta-miR-2462 | -0.34 |
| RNF144B | ENST00000259939.3 | ring finger protein 144B | 17 | 2 | 1 | 1 | 0 | 0 | bta-miR-2462 | -0.34 |
| ZNF345 | ENST00000589046.1 | zinc finger protein 345 | 24 | 1 | 0 | 1 | 0 | 2 | bta-miR-2462 | -0.34 |
| ST8SIA1 | ENST00000404299.3 | ST8 alpha-N-acetyl-neuraminide alpha-2,8-sialyltransferase 1 | 5 | 1 | 0 | 0 | 1 | 2 | bta-miR-2462 | -0.34 |
| MOAP1 | ENST00000298894.4 | modulator of apoptosis 1 | 79 | 2 | 0 | 1 | 1 | 0 | bta-miR-2462 | -0.34 |
| RALB | ENST00000272519.5 | v-ral simian leukemia viral oncogene homolog B | 527 | 2 | 0 | 2 | 0 | 0 | bta-miR-2462 | -0.34 |
| AC012215.1 | ENST00000437887.1 | Uncharacterized protein | 31 | 3 | 0 | 3 | 0 | 1 | bta-miR-2462 | -0.33 |
| RAB2A | ENST00000262646.7 | RAB2A, member RAS oncogene family | 788 | 2 | 0 | 2 | 0 | 2 | bta-miR-2462 | -0.33 |
| SFTPB | ENST00000519937.2 | surfactant protein B | 5 | 1 | 1 | 0 | 0 | 0 | bta-miR-2462 | -0.33 |
| SAMD5 | ENST00000367474.1 | sterile alpha motif domain containing 5 | 28 | 2 | 0 | 1 | 1 | 0 | bta-miR-2462 | -0.33 |
| ATP5J2 | ENST00000449683.1 | ATP synthase, H+ transporting, mitochondrial Fo complex, subunit F2 | 4357 | 1 | 0 | 1 | 0 | 0 | bta-miR-2462 | -0.33 |
| ATP6V1A | ENST00000273398.3 | ATPase, H+ transporting, lysosomal 70kDa, V1 subunit A | 180 | 2 | 1 | 0 | 1 | 1 | bta-miR-2462 | -0.33 |
| LMNB1 | ENST00000261366.5 | lamin B1 | 369 | 1 | 1 | 0 | 0 | 0 | bta-miR-2462 | -0.33 |
| ONECUT3 | ENST00000382349.4 | one cut homeobox 3 | 5 | 1 | 1 | 0 | 0 | 0 | bta-miR-2462 | -0.33 |
| VSIG1 | ENST00000415430.3 | V-set and immunoglobulin domain containing 1 | 5 | 1 | 1 | 0 | 0 | 0 | bta-miR-2462 | -0.33 |
| VHL | ENST00000256474.2 | von Hippel-Lindau tumor suppressor, E3 ubiquitin protein ligase | 2386 | 2 | 0 | 1 | 1 | 2 | bta-miR-2462 | -0.32 |
| FAM133B | ENST00000438306.1 | family with sequence similarity 133, member B | 32 | 1 | 1 | 0 | 0 | 1 | bta-miR-2462 | -0.32 |
| MPZL3 | ENST00000278949.4 | myelin protein zero-like 3 | 59 | 2 | 0 | 2 | 0 | 0 | bta-miR-2462 | -0.32 |
| CPVL | ENST00000265394.5 | carboxypeptidase, vitellogenic-like | 439 | 1 | 0 | 0 | 1 | 0 | bta-miR-2462 | -0.32 |
| COX7A2L | ENST00000378669.1 | cytochrome c oxidase subunit VIIa polypeptide 2 like | 289 | 1 | 0 | 1 | 0 | 0 | bta-miR-2462 | -0.32 |
| CHST11 | ENST00000549260.1 | carbohydrate (chondroitin 4) sulfotransferase 11 | 686 | 1 | 1 | 0 | 0 | 0 | bta-miR-2462 | -0.32 |
| PSMB4 | ENST00000290541.6 | proteasome (prosome, macropain) subunit, beta type, 4 | 192 | 1 | 0 | 1 | 0 | 0 | bta-miR-2462 | -0.32 |
| AGR2 | ENST00000419304.2 | anterior gradient 2 | 13 | 1 | 0 | 1 | 0 | 1 | bta-miR-2462 | -0.32 |
| ZNF709 | ENST00000428311.1 | | 90 | 3 | 0 | 0 | 3 | 0 | bta-miR-2462 | -0.32 |
| ATP6V1G2 | ENST00000376151.4 | ATPase, H+ transporting, lysosomal 13kDa, V1 subunit G2 | 16 | 1 | 0 | 1 | 0 | 1 | bta-miR-2462 | -0.32 |
| BPI | ENST00000451435.1 | bactericidal/permeability-increasing protein | 5 | 1 | 0 | 1 | 0 | 2 | bta-miR-2462 | -0.32 |
| C5orf51 | ENST00000381647.2 | chromosome 5 open reading frame 51 | 124 | 1 | 1 | 0 | 0 | 1 | bta-miR-2462 | -0.32 |
| CCSAP | ENST00000366687.1 | centriole, cilia and spindle-associated protein | 1210 | 1 | 1 | 0 | 0 | 0 | bta-miR-2462 | -0.32 |
| CCDC47 | ENST00000225726.5 | coiled-coil domain containing 47 | 1484 | 1 | 1 | 0 | 0 | 0 | bta-miR-2462 | -0.32 |
| GPRASP1 | ENST00000361600.5 | G protein-coupled receptor associated sorting protein 1 | 25 | 2 | 0 | 0 | 2 | 1 | bta-miR-2462 | -0.32 |
| ZNF770 | ENST00000356321.4 | zinc finger protein 770 | 52 | 1 | 1 | 0 | 0 | 1 | bta-miR-2462 | -0.32 |
| CMC1 | ENST00000466830.1 | COX assembly mitochondrial protein 1 homolog (S. cerevisiae) | 1203 | 2 | 0 | 0 | 2 | 1 | bta-miR-2462 | -0.31 |
| USP45 | ENST00000392738.2 | ubiquitin specific peptidase 45 | 491 | 2 | 0 | 1 | 1 | 0 | bta-miR-2462 | -0.31 |
| EXOC6 | ENST00000371552.4 | exocyst complex component 6 | 261 | 3 | 0 | 1 | 2 | 0 | bta-miR-2462 | -0.31 |
| CAPRIN2 | ENST00000395805.2 | caprin family member 2 | 17 | 2 | 1 | 0 | 1 | 0 | bta-miR-2462 | -0.31 |
| MAP4K5 | ENST00000013125.4 | mitogen-activated protein kinase kinase kinase kinase 5 | 634 | 1 | 1 | 0 | 0 | 0 | bta-miR-2462 | -0.31 |
| SYTL5 | ENST00000357972.5 | synaptotagmin-like 5 | 37 | 1 | 1 | 0 | 0 | 0 | bta-miR-2462 | -0.31 |
| C1orf51 | ENST00000369095.1 | chromosome 1 open reading frame 51 | 5 | 1 | 0 | 1 | 0 | 0 | bta-miR-2462 | -0.31 |
| ELAC1 | ENST00000269466.3 | elaC ribonuclease Z 1 | 7 | 1 | 0 | 1 | 0 | 1 | bta-miR-2462 | -0.31 |
| ARCN1 | ENST00000534182.2 | archain 1 | 1139 | 1 | 1 | 0 | 0 | 0 | bta-miR-2462 | -0.31 |
| PRG4 | ENST00000367483.4 | proteoglycan 4 | 5 | 1 | 1 | 0 | 0 | 1 | bta-miR-2462 | -0.31 |
| AC005008.2 | ENST00000431501.1 | Uncharacterized protein | 5 | 1 | 0 | 1 | 0 | 1 | bta-miR-2462 | -0.31 |
| TMEM71 | ENST00000356838.3 | transmembrane protein 71 | 5 | 1 | 0 | 1 | 0 | 3 | bta-miR-2462 | -0.31 |
| C9orf85 | ENST00000486911.2 | chromosome 9 open reading frame 85 | 57 | 1 | 0 | 1 | 0 | 0 | bta-miR-2462 | -0.3 |
| CHRNA7 | ENST00000306901.3 | cholinergic receptor, nicotinic, alpha 7 (neuronal) | 5 | 2 | 1 | 0 | 1 | 1 | bta-miR-2462 | -0.3 |
| HSD11B1L | ENST00000581893.1 | hydroxysteroid (11-beta) dehydrogenase 1-like | 211 | 1 | 0 | 1 | 0 | 0 | bta-miR-2462 | -0.3 |
| APOLD1 | ENST00000356591.4 | apolipoprotein L domain containing 1 | 27 | 1 | 1 | 0 | 0 | 2 | bta-miR-2462 | -0.3 |
| TCEAL7 | ENST00000332431.4 | transcription elongation factor A (SII)-like 7 | 15 | 1 | 0 | 1 | 0 | 0 | bta-miR-2462 | -0.3 |
| SCGB1D4 | ENST00000358585.1 | secretoglobin, family 1D, member 4 | 5 | 1 | 0 | 1 | 0 | 0 | bta-miR-2462 | -0.3 |
| PEA15 | ENST00000360472.4 | phosphoprotein enriched in astrocytes 15 | 5239 | 1 | 1 | 0 | 0 | 1 | bta-miR-2462 | -0.3 |
| GNGT1 | ENST00000248572.5 | guanine nucleotide binding protein (G protein), gamma transducing activity polypeptide 1 | 5 | 1 | 0 | 0 | 1 | 0 | bta-miR-2462 | -0.3 |
| ZKSCAN3 | ENST00000377255.3 | zinc finger with KRAB and SCAN domains 3 | 63 | 1 | 1 | 0 | 0 | 0 | bta-miR-2462 | -0.3 |
| GNAI3 | ENST00000369851.4 | guanine nucleotide binding protein (G protein), alpha inhibiting activity polypeptide 3 | 2439 | 1 | 1 | 0 | 0 | 0 | bta-miR-2462 | -0.3 |
| MARC2 | ENST00000366913.3 | mitochondrial amidoxime reducing component 2 | 213 | 1 | 1 | 0 | 0 | 0 | bta-miR-2462 | -0.3 |
| PPTC7 | ENST00000354300.3 | PTC7 protein phosphatase homolog (S. cerevisiae) | 817 | 1 | 1 | 0 | 0 | 1 | bta-miR-2462 | -0.3 |
| ZNF709 | ENST00000397732.3 | zinc finger protein 709 | 90 | 3 | 0 | 0 | 3 | 0 | bta-miR-2462 | -0.3 |
| AMFR | ENST00000290649.5 | autocrine motility factor receptor, E3 ubiquitin protein ligase | 1350 | 2 | 0 | 2 | 0 | 0 | bta-miR-2462 | -0.3 |
| AC090186.1 | ENST00000415643.1 | Uncharacterized protein | 5 | 1 | 0 | 0 | 1 | 0 | bta-miR-2462 | -0.3 |
| SNX3 | ENST00000230085.8 | sorting nexin 3 | 1843 | 1 | 0 | 1 | 0 | 0 | bta-miR-2462 | -0.3 |
| NIPSNAP1 | ENST00000216121.7 | nipsnap homolog 1 (C. elegans) | 2429 | 1 | 1 | 0 | 0 | 0 | bta-miR-2462 | -0.29 |
| TBX15 | ENST00000207157.3 | T-box 15 | 14 | 1 | 0 | 1 | 0 | 1 | bta-miR-2462 | -0.29 |
| MPZ | ENST00000533357.1 | myelin protein zero | 17 | 1 | 1 | 0 | 0 | 0 | bta-miR-2462 | -0.29 |
| C2orf83 | ENST00000409066.1 | chromosome 2 open reading frame 83 | 5 | 1 | 0 | 1 | 0 | 0 | bta-miR-2462 | -0.29 |
| CDH5 | ENST00000341529.3 | cadherin 5, type 2 (vascular endothelium) | 8 | 1 | 1 | 0 | 0 | 0 | bta-miR-2462 | -0.29 |
| SASS6 | ENST00000287482.5 | spindle assembly 6 homolog (C. elegans) | 796 | 1 | 1 | 0 | 0 | 1 | bta-miR-2462 | -0.29 |
| PPCS | ENST00000372556.3 | phosphopantothenoylcysteine synthetase | 491 | 2 | 0 | 2 | 0 | 1 | bta-miR-2462 | -0.29 |
| NHLRC3 | ENST00000470258.1 | NHL repeat containing 3 | 49 | 1 | 1 | 0 | 0 | 0 | bta-miR-2462 | -0.29 |
| SGTB | ENST00000381007.4 | small glutamine-rich tetratricopeptide repeat (TPR)-containing, beta | 205 | 1 | 1 | 0 | 0 | 1 | bta-miR-2462 | -0.29 |
| SLC46A3 | ENST00000266943.6 | solute carrier family 46, member 3 | 5 | 1 | 1 | 0 | 0 | 1 | bta-miR-2462 | -0.29 |
| ZC3HAV1L | ENST00000275766.1 | zinc finger CCCH-type, antiviral 1-like | 98 | 1 | 1 | 0 | 0 | 1 | bta-miR-2462 | -0.29 |
| GRB2 | ENST00000392563.1 | growth factor receptor-bound protein 2 | 2660 | 1 | 0 | 1 | 0 | 1 | bta-miR-2462 | -0.29 |
| PRRC2C | ENST00000367742.3 | proline-rich coiled-coil 2C | 5 | 1 | 1 | 0 | 0 | 0 | bta-miR-2462 | -0.28 |
| ZNF713 | ENST00000429591.2 | zinc finger protein 713 | 5 | 1 | 0 | 1 | 0 | 1 | bta-miR-2462 | -0.28 |
| CDK19 | ENST00000368911.3 | cyclin-dependent kinase 19 | 51 | 3 | 2 | 1 | 0 | 2 | bta-miR-2462 | -0.28 |
| VRK2 | ENST00000412104.2 | vaccinia related kinase 2 | 25 | 1 | 0 | 1 | 0 | 0 | bta-miR-2462 | -0.28 |
| PEX3 | ENST00000367591.4 | peroxisomal biogenesis factor 3 | 314 | 1 | 1 | 0 | 0 | 0 | bta-miR-2462 | -0.28 |
| TMEM170A | ENST00000357613.4 | transmembrane protein 170A | 70 | 3 | 0 | 0 | 3 | 1 | bta-miR-2462 | -0.28 |
| SCRG1 | ENST00000296506.3 | stimulator of chondrogenesis 1 | 5 | 2 | 0 | 0 | 2 | 1 | bta-miR-2462 | -0.28 |
| RTP2 | ENST00000358241.1 | receptor (chemosensory) transporter protein 2 | 5 | 1 | 0 | 1 | 0 | 0 | bta-miR-2462 | -0.28 |
| VPS13C | ENST00000249837.3 | vacuolar protein sorting 13 homolog C (S. cerevisiae) | 85 | 2 | 0 | 1 | 1 | 0 | bta-miR-2462 | -0.28 |
| ETNK1 | ENST00000266517.4 | ethanolamine kinase 1 | 1047 | 2 | 0 | 2 | 0 | 1 | bta-miR-2462 | -0.28 |
| KIF14 | ENST00000367350.4 | kinesin family member 14 | 451 | 1 | 1 | 0 | 0 | 0 | bta-miR-2462 | -0.28 |
| CTDSPL2 | ENST00000260327.4 | CTD (carboxy-terminal domain, RNA polymerase II, polypeptide A) small phosphatase like 2 | 457 | 2 | 2 | 0 | 0 | 0 | bta-miR-2462 | -0.28 |
| IAPP | ENST00000240652.3 | islet amyloid polypeptide | 5 | 1 | 0 | 1 | 0 | 1 | bta-miR-2462 | -0.28 |
| MAP2K7 | ENST00000397981.3 | mitogen-activated protein kinase kinase 7 | 164 | 1 | 1 | 0 | 0 | 0 | bta-miR-2462 | -0.28 |
| DCAF12L2 | ENST00000538699.1 | DDB1 and CUL4 associated factor 12-like 2 | 20 | 1 | 1 | 0 | 0 | 1 | bta-miR-2462 | -0.28 |
| XRCC4 | ENST00000511817.1 | X-ray repair complementing defective repair in Chinese hamster cells 4 | 573 | 1 | 0 | 1 | 0 | 0 | bta-miR-2462 | -0.28 |
| TTC6 | ENST00000476979.1 | tetratricopeptide repeat domain 6 | 5 | 1 | 0 | 1 | 0 | 0 | bta-miR-2462 | -0.28 |
| C9 | ENST00000263408.4 | complement component 9 | 5 | 1 | 1 | 0 | 0 | 0 | bta-miR-2462 | -0.28 |
| SLC1A6 | ENST00000598504.1 | solute carrier family 1 (high affinity aspartate/glutamate transporter), member 6 | 7 | 1 | 0 | 1 | 0 | 0 | bta-miR-2462 | -0.28 |
| PROX1 | ENST00000366958.4 | prospero homeobox 1 | 131 | 1 | 0 | 1 | 0 | 2 | bta-miR-2462 | -0.28 |
| ATG4C | ENST00000317868.4 | autophagy related 4C, cysteine peptidase | 82 | 2 | 0 | 2 | 0 | 0 | bta-miR-2462 | -0.27 |
| MYF6 | ENST00000228641.3 | myogenic factor 6 (herculin) | 5 | 1 | 0 | 1 | 0 | 1 | bta-miR-2462 | -0.27 |
| KIAA2026 | ENST00000399933.3 | KIAA2026 | 712 | 1 | 1 | 0 | 0 | 0 | bta-miR-2462 | -0.27 |
| TAL1 | ENST00000371884.2 | T-cell acute lymphocytic leukemia 1 | 5 | 2 | 1 | 1 | 0 | 1 | bta-miR-2462 | -0.27 |
| LETM1 | ENST00000302787.2 | leucine zipper-EF-hand containing transmembrane protein 1 | 198 | 2 | 0 | 1 | 1 | 0 | bta-miR-2462 | -0.27 |
| LRRC23 | ENST00000433346.1 | leucine rich repeat containing 23 | 202 | 1 | 0 | 0 | 1 | 0 | bta-miR-2462 | -0.27 |
| NME1 | ENST00000511355.1 | NME/NM23 nucleoside diphosphate kinase 1 | 25174 | 1 | 0 | 0 | 1 | 0 | bta-miR-2462 | -0.27 |
| DTX3L | ENST00000296161.4 | deltex 3-like (Drosophila) | 319 | 1 | 0 | 1 | 0 | 2 | bta-miR-2462 | -0.27 |
| MSN | ENST00000360270.5 | moesin | 5947 | 2 | 1 | 1 | 0 | 0 | bta-miR-2462 | -0.27 |
| TAF7L | ENST00000372907.3 | TAF7-like RNA polymerase II, TATA box binding protein (TBP)-associated factor, 50kDa | 51 | 2 | 0 | 0 | 2 | 0 | bta-miR-2462 | -0.27 |
| POU3F2 | ENST00000328345.5 | POU class 3 homeobox 2 | 274 | 2 | 0 | 1 | 1 | 1 | bta-miR-2462 | -0.27 |
| ARPC5L | ENST00000353214.2 | actin related protein 2/3 complex, subunit 5-like | 851 | 1 | 0 | 1 | 0 | 0 | bta-miR-2462 | -0.27 |
| PCYT1A | ENST00000292823.2 | phosphate cytidylyltransferase 1, choline, alpha | 731 | 2 | 1 | 1 | 0 | 1 | bta-miR-2462 | -0.27 |
| PRNP | ENST00000379440.4 | prion protein | 1136 | 1 | 1 | 0 | 0 | 0 | bta-miR-2462 | -0.27 |
| OOEP | ENST00000370363.1 | oocyte expressed protein | 22 | 1 | 0 | 0 | 1 | 0 | bta-miR-2462 | -0.27 |
| F9 | ENST00000218099.2 | coagulation factor IX | 5 | 1 | 0 | 0 | 1 | 2 | bta-miR-2462 | -0.27 |
| UBE2E3 | ENST00000410062.4 | ubiquitin-conjugating enzyme E2E 3 | 15 | 1 | 1 | 0 | 0 | 1 | bta-miR-2462 | -0.26 |
| MLTK | ENST00000338983.3 | Mitogen-activated protein kinase kinase kinase MLT | 766 | 1 | 1 | 0 | 0 | 2 | bta-miR-2462 | -0.26 |
| SNX25 | ENST00000504273.1 | sorting nexin 25 | 21 | 1 | 0 | 1 | 0 | 0 | bta-miR-2462 | -0.26 |
| CCDC141 | ENST00000409284.1 | coiled-coil domain containing 141 | 5 | 2 | 0 | 1 | 1 | 1 | bta-miR-2462 | -0.26 |
| LRRC63 | ENST00000595396.1 | leucine rich repeat containing 63 | 5 | 1 | 0 | 1 | 0 | 0 | bta-miR-2462 | -0.26 |
| PDCL3 | ENST00000264254.6 | phosducin-like 3 | 58 | 1 | 0 | 1 | 0 | 0 | bta-miR-2462 | -0.26 |
| FAM107B | ENST00000378470.1 | family with sequence similarity 107, member B | 403 | 1 | 1 | 0 | 0 | 2 | bta-miR-2462 | -0.26 |
| FAM183A | ENST00000410048.1 | family with sequence similarity 183, member A | 5 | 1 | 0 | 1 | 0 | 0 | bta-miR-2462 | -0.26 |
| CD14 | ENST00000302014.6 | CD14 molecule | 5 | 1 | 0 | 0 | 1 | 0 | bta-miR-2462 | -0.26 |
| ANKRD36 | ENST00000357042.4 | ankyrin repeat domain 36 | 5 | 1 | 0 | 1 | 0 | 0 | bta-miR-2462 | -0.26 |
| ANKRD46 | ENST00000335659.3 | ankyrin repeat domain 46 | 33 | 2 | 0 | 2 | 0 | 0 | bta-miR-2462 | -0.26 |
| ZNF131 | ENST00000505606.2 | zinc finger protein 131 | 1015 | 1 | 1 | 0 | 0 | 0 | bta-miR-2462 | -0.26 |
| H2AFY2 | ENST00000373255.4 | H2A histone family, member Y2 | 46 | 1 | 0 | 1 | 0 | 0 | bta-miR-2462 | -0.26 |
| NMU | ENST00000511469.1 | neuromedin U | 7 | 1 | 0 | 1 | 0 | 0 | bta-miR-2462 | -0.26 |
| LCP2 | ENST00000046794.5 | lymphocyte cytosolic protein 2 (SH2 domain containing leukocyte protein of 76kDa) | 5 | 3 | 0 | 2 | 1 | 3 | bta-miR-2462 | -0.26 |
| RAB27B | ENST00000262094.5 | RAB27B, member RAS oncogene family | 5 | 2 | 0 | 1 | 1 | 0 | bta-miR-2462 | -0.26 |
| TIAM2 | ENST00000461783.3 | T-cell lymphoma invasion and metastasis 2 | 192 | 1 | 1 | 0 | 0 | 0 | bta-miR-2462 | -0.26 |
| CLEC3A | ENST00000299642.4 | C-type lectin domain family 3, member A | 5 | 1 | 0 | 1 | 0 | 1 | bta-miR-2462 | -0.26 |
| CXCL5 | ENST00000296027.4 | chemokine (C-X-C motif) ligand 5 | 5 | 1 | 0 | 1 | 0 | 0 | bta-miR-2462 | -0.26 |
| KRTAP13-2 | ENST00000399889.2 | keratin associated protein 13-2 | 5 | 1 | 0 | 1 | 0 | 0 | bta-miR-2462 | -0.26 |
| SLC39A12 | ENST00000377369.2 | solute carrier family 39 (zinc transporter), member 12 | 5 | 1 | 0 | 1 | 0 | 0 | bta-miR-2462 | -0.26 |
| TTC33 | ENST00000337702.4 | tetratricopeptide repeat domain 33 | 676 | 1 | 1 | 0 | 0 | 2 | bta-miR-2462 | -0.25 |
| C10orf82 | ENST00000369210.3 | chromosome 10 open reading frame 82 | 5 | 1 | 0 | 1 | 0 | 0 | bta-miR-2462 | -0.25 |
| DBI | ENST00000355857.3 | diazepam binding inhibitor (GABA receptor modulator, acyl-CoA binding protein) | 623 | 1 | 0 | 1 | 0 | 0 | bta-miR-2462 | -0.25 |
| NINL | ENST00000278886.6 | ninein-like | 121 | 1 | 0 | 1 | 0 | 0 | bta-miR-2462 | -0.25 |
| CXCL6 | ENST00000226317.5 | chemokine (C-X-C motif) ligand 6 | 26 | 1 | 0 | 1 | 0 | 0 | bta-miR-2462 | -0.25 |
| CD79A | ENST00000221972.3 | CD79a molecule, immunoglobulin-associated alpha | 5 | 1 | 0 | 1 | 0 | 0 | bta-miR-2462 | -0.25 |
| LCORL | ENST00000326877.4 | ligand dependent nuclear receptor corepressor-like | 194 | 1 | 0 | 0 | 1 | 0 | bta-miR-2462 | -0.25 |
| POC1B | ENST00000378528.2 | POC1 centriolar protein B | 61 | 1 | 0 | 1 | 0 | 1 | bta-miR-2462 | -0.25 |
| PARP9 | ENST00000477522.2 | poly (ADP-ribose) polymerase family, member 9 | 456 | 1 | 0 | 1 | 0 | 0 | bta-miR-2462 | -0.25 |
| BLOC1S6 | ENST00000220531.3 | biogenesis of lysosomal organelles complex-1, subunit 6, pallidin | 1754 | 1 | 0 | 1 | 0 | 1 | bta-miR-2462 | -0.25 |
| C1orf158 | ENST00000288048.5 | chromosome 1 open reading frame 158 | 5 | 1 | 0 | 1 | 0 | 0 | bta-miR-2462 | -0.25 |
| SMNDC1 | ENST00000369603.5 | survival motor neuron domain containing 1 | 949 | 1 | 0 | 0 | 1 | 0 | bta-miR-2462 | -0.25 |
| GPR63 | ENST00000229955.3 | G protein-coupled receptor 63 | 5 | 2 | 1 | 1 | 0 | 0 | bta-miR-2462 | -0.25 |
| MUC15 | ENST00000455601.2 | mucin 15, cell surface associated | 7 | 2 | 0 | 2 | 0 | 0 | bta-miR-2462 | -0.25 |
| UMOD | ENST00000396134.2 | uromodulin | 5 | 1 | 0 | 1 | 0 | 0 | bta-miR-2462 | -0.25 |
| SNAI2 | ENST00000020945.1 | snail family zinc finger 2 | 1233 | 1 | 0 | 1 | 0 | 1 | bta-miR-2462 | -0.25 |
| RMND5A | ENST00000283632.4 | required for meiotic nuclear division 5 homolog A (S. cerevisiae) | 261 | 2 | 0 | 1 | 1 | 1 | bta-miR-2462 | -0.25 |
| CMTR2 | ENST00000338099.5 | cap methyltransferase 2 | 259 | 1 | 0 | 1 | 0 | 0 | bta-miR-2462 | -0.25 |
| QPCT | ENST00000338415.3 | glutaminyl-peptide cyclotransferase | 463 | 1 | 0 | 1 | 0 | 0 | bta-miR-2462 | -0.25 |
| NPTN | ENST00000345330.4 | neuroplastin | 2918 | 1 | 0 | 1 | 0 | 0 | bta-miR-2462 | -0.25 |
| ST6GALNAC3 | ENST00000328299.3 | ST6 (alpha-N-acetyl-neuraminyl-2,3-beta-galactosyl-1,3)-N-acetylgalactosaminide alpha-2,6-sialyltransferase 3 | 41 | 4 | 0 | 2 | 2 | 1 | bta-miR-2462 | -0.25 |
| ACTR5 | ENST00000243903.4 | ARP5 actin-related protein 5 homolog (yeast) | 41 | 1 | 0 | 1 | 0 | 0 | bta-miR-2462 | -0.25 |
| ZBED3 | ENST00000255198.2 | zinc finger, BED-type containing 3 | 399 | 2 | 1 | 1 | 0 | 0 | bta-miR-2462 | -0.25 |
| ADAMDEC1 | ENST00000256412.4 | ADAM-like, decysin 1 | 5 | 1 | 0 | 1 | 0 | 0 | bta-miR-2462 | -0.25 |
| SPRYD4 | ENST00000338146.5 | SPRY domain containing 4 | 1999 | 2 | 0 | 2 | 0 | 0 | bta-miR-2462 | -0.25 |
| TXNL4B | ENST00000268483.3 | thioredoxin-like 4B | 200 | 2 | 0 | 2 | 0 | 0 | bta-miR-2462 | -0.25 |
| PRDM5 | ENST00000515109.1 | PR domain containing 5 | 22 | 2 | 0 | 1 | 1 | 1 | bta-miR-2462 | -0.25 |
| ACSL6 | ENST00000379264.2 | acyl-CoA synthetase long-chain family member 6 | 7 | 2 | 1 | 1 | 0 | 0 | bta-miR-2462 | -0.24 |
| CHD8 | ENST00000430710.3 | chromodomain helicase DNA binding protein 8 | 191 | 1 | 0 | 1 | 0 | 0 | bta-miR-2462 | -0.24 |
| PPP1R17 | ENST00000342032.3 | protein phosphatase 1, regulatory subunit 17 | 5 | 1 | 0 | 1 | 0 | 0 | bta-miR-2462 | -0.24 |
| AHR | ENST00000242057.4 | aryl hydrocarbon receptor | 25 | 2 | 0 | 1 | 1 | 0 | bta-miR-2462 | -0.24 |
| FAM200B | ENST00000422728.2 | family with sequence similarity 200, member B | 213 | 1 | 0 | 1 | 0 | 1 | bta-miR-2462 | -0.24 |
| MYL1 | ENST00000341685.4 | myosin, light chain 1, alkali; skeletal, fast | 5 | 1 | 0 | 0 | 1 | 0 | bta-miR-2462 | -0.24 |
| ZNF148 | ENST00000360647.4 | zinc finger protein 148 | 230 | 4 | 0 | 2 | 2 | 1 | bta-miR-2462 | -0.24 |
| NIPAL2 | ENST00000341166.3 | NIPA-like domain containing 2 | 7 | 3 | 0 | 1 | 2 | 1 | bta-miR-2462 | -0.24 |
| FAM151B | ENST00000282226.4 | family with sequence similarity 151, member B | 35 | 1 | 0 | 1 | 0 | 0 | bta-miR-2462 | -0.24 |
| CRH | ENST00000276571.3 | corticotropin releasing hormone | 5 | 1 | 0 | 1 | 0 | 0 | bta-miR-2462 | -0.24 |
| CREG1 | ENST00000370509.4 | cellular repressor of E1A-stimulated genes 1 | 318 | 1 | 1 | 0 | 0 | 1 | bta-miR-2462 | -0.24 |
| SECTM1 | ENST00000269389.3 | secreted and transmembrane 1 | 30 | 1 | 0 | 0 | 1 | 0 | bta-miR-2462 | -0.24 |
| WFDC11 | ENST00000356562.2 | WAP four-disulfide core domain 11 | 5 | 1 | 0 | 1 | 0 | 0 | bta-miR-2462 | -0.24 |
| PANK1 | ENST00000322191.6 | pantothenate kinase 1 | 435 | 2 | 0 | 2 | 0 | 1 | bta-miR-2462 | -0.24 |
| RPP25 | ENST00000322177.5 | ribonuclease P/MRP 25kDa subunit | 546 | 2 | 1 | 1 | 0 | 0 | bta-miR-2462 | -0.24 |
| TNF | ENST00000449264.2 | tumor necrosis factor | 5 | 1 | 0 | 1 | 0 | 0 | bta-miR-2462 | -0.24 |
| GTF2E1 | ENST00000283875.5 | general transcription factor IIE, polypeptide 1, alpha 56kDa | 218 | 1 | 1 | 0 | 0 | 1 | bta-miR-2462 | -0.24 |
| ADRA1A | ENST00000380581.2 | adrenoceptor alpha 1A | 0 | 1 | 1 | 0 | 0 | 0 | bta-miR-2462 | -0.24 |
| PCTP | ENST00000576183.1 | phosphatidylcholine transfer protein | 476 | 1 | 1 | 0 | 0 | 0 | bta-miR-2462 | -0.24 |
| EGLN1 | ENST00000366641.3 | egl-9 family hypoxia-inducible factor 1 | 505 | 1 | 1 | 0 | 0 | 0 | bta-miR-2462 | -0.24 |
| PFKFB2 | ENST00000367080.3 | 6-phosphofructo-2-kinase/fructose-2,6-biphosphatase 2 | 83 | 3 | 2 | 0 | 1 | 1 | bta-miR-2462 | -0.24 |
| C12orf76 | ENST00000546651.2 | chromosome 12 open reading frame 76 | 62 | 1 | 0 | 1 | 0 | 0 | bta-miR-2462 | -0.24 |
| CD48 | ENST00000368045.3 | CD48 molecule | 5 | 1 | 0 | 1 | 0 | 1 | bta-miR-2462 | -0.24 |
| DCLK1 | ENST00000379892.4 | doublecortin-like kinase 1 | 9 | 2 | 1 | 1 | 0 | 1 | bta-miR-2462 | -0.24 |
| ORAI3 | ENST00000318663.4 | ORAI calcium release-activated calcium modulator 3 | 565 | 1 | 1 | 0 | 0 | 0 | bta-miR-2462 | -0.24 |
| PRR23C | ENST00000413199.1 | proline rich 23C | 5 | 1 | 0 | 1 | 0 | 1 | bta-miR-2462 | -0.24 |
| PROK2 | ENST00000353065.3 | prokineticin 2 | 7 | 1 | 0 | 0 | 1 | 1 | bta-miR-2462 | -0.24 |
| ARPP21 | ENST00000428373.1 | cAMP-regulated phosphoprotein, 21kDa | 5 | 2 | 0 | 0 | 2 | 0 | bta-miR-2462 | -0.24 |
| ZNF566 | ENST00000454319.1 | zinc finger protein 566 | 310 | 1 | 0 | 1 | 0 | 4 | bta-miR-2462 | -0.24 |
| SALL4 | ENST00000217086.4 | sal-like 4 (Drosophila) | 155 | 1 | 1 | 0 | 0 | 0 | bta-miR-2462 | -0.24 |
| IL33 | ENST00000381434.3 | interleukin 33 | 8 | 1 | 0 | 1 | 0 | 0 | bta-miR-2462 | -0.24 |
| CRCP | ENST00000415001.2 | CGRP receptor component | 1776 | 1 | 0 | 1 | 0 | 0 | bta-miR-2462 | -0.24 |
| SPATA8 | ENST00000328504.3 | spermatogenesis associated 8 | 10 | 1 | 0 | 1 | 0 | 0 | bta-miR-2462 | -0.24 |
| RBPMS2 | ENST00000560606.1 | RNA binding protein with multiple splicing 2 | 34 | 1 | 0 | 1 | 0 | 0 | bta-miR-2462 | -0.24 |
| FAHD1 | ENST00000427358.2 | fumarylacetoacetate hydrolase domain containing 1 | 130 | 1 | 0 | 1 | 0 | 0 | bta-miR-2462 | -0.23 |
| ZNF596 | ENST00000308811.4 | zinc finger protein 596 | 37 | 1 | 0 | 0 | 1 | 1 | bta-miR-2462 | -0.23 |
| SLC36A2 | ENST00000335244.4 | solute carrier family 36 (proton/amino acid symporter), member 2 | 5 | 1 | 1 | 0 | 0 | 0 | bta-miR-2462 | -0.23 |
| MS4A1 | ENST00000534668.1 | membrane-spanning 4-domains, subfamily A, member 1 | 5 | 2 | 0 | 1 | 1 | 1 | bta-miR-2462 | -0.23 |
| XIAP | ENST00000371199.3 | X-linked inhibitor of apoptosis | 153 | 4 | 1 | 2 | 1 | 1 | bta-miR-2462 | -0.23 |
| TPTE2 | ENST00000382978.1 | transmembrane phosphoinositide 3-phosphatase and tensin homolog 2 | 5 | 1 | 0 | 1 | 0 | 0 | bta-miR-2462 | -0.23 |
| UPP2 | ENST00000605860.1 | uridine phosphorylase 2 | 5 | 1 | 0 | 1 | 0 | 1 | bta-miR-2462 | -0.23 |
| BLOC1S5 | ENST00000397457.2 | biogenesis of lysosomal organelles complex-1, subunit 5, muted | 187 | 1 | 0 | 1 | 0 | 1 | bta-miR-2462 | -0.23 |
| NRGN | ENST00000412681.2 | neurogranin (protein kinase C substrate, RC3) | 60 | 1 | 0 | 1 | 0 | 0 | bta-miR-2462 | -0.23 |
| ZNF782 | ENST00000481138.1 | zinc finger protein 782 | 8 | 1 | 1 | 0 | 0 | 0 | bta-miR-2462 | -0.23 |
| CCDC50 | ENST00000392455.3 | coiled-coil domain containing 50 | 961 | 2 | 1 | 1 | 0 | 1 | bta-miR-2462 | -0.23 |
| UBE3A | ENST00000232165.3 | ubiquitin protein ligase E3A | 529 | 1 | 0 | 1 | 0 | 1 | bta-miR-2462 | -0.23 |
| FRMD3 | ENST00000304195.3 | FERM domain containing 3 | 14 | 1 | 1 | 0 | 0 | 0 | bta-miR-2462 | -0.23 |
| LSMEM1 | ENST00000312849.4 | leucine-rich single-pass membrane protein 1 | 10 | 1 | 0 | 1 | 0 | 0 | bta-miR-2462 | -0.23 |
| TOX | ENST00000361421.1 | thymocyte selection-associated high mobility group box | 139 | 1 | 0 | 1 | 0 | 0 | bta-miR-2462 | -0.23 |
| PAFAH1B2 | ENST00000527958.1 | platelet-activating factor acetylhydrolase 1b, catalytic subunit 2 (30kDa) | 180 | 2 | 0 | 2 | 0 | 0 | bta-miR-2462 | -0.23 |
| FGD4 | ENST00000427716.2 | FYVE, RhoGEF and PH domain containing 4 | 38 | 2 | 0 | 2 | 0 | 1 | bta-miR-2462 | -0.23 |
| NXT2 | ENST00000372106.1 | nuclear transport factor 2-like export factor 2 | 390 | 1 | 0 | 1 | 0 | 0 | bta-miR-2462 | -0.23 |
| GPHN | ENST00000478722.1 | gephyrin | 122 | 1 | 0 | 1 | 0 | 0 | bta-miR-2462 | -0.23 |
| TSGA10 | ENST00000393483.3 | testis specific, 10 | 8 | 1 | 1 | 0 | 0 | 1 | bta-miR-2462 | -0.23 |
| TUSC1 | ENST00000358022.3 | tumor suppressor candidate 1 | 987 | 1 | 0 | 1 | 0 | 1 | bta-miR-2462 | -0.23 |
| LLPH | ENST00000266604.2 | LLP homolog, long-term synaptic facilitation (Aplysia) | 88 | 1 | 0 | 0 | 1 | 0 | bta-miR-2462 | -0.23 |
| HRSP12 | ENST00000254878.3 | heat-responsive protein 12 | 209 | 1 | 0 | 1 | 0 | 0 | bta-miR-2462 | -0.23 |
| SOX4 | ENST00000244745.1 | SRY (sex determining region Y)-box 4 | 2736 | 1 | 0 | 1 | 0 | 1 | bta-miR-2462 | -0.23 |
| GHR | ENST00000230882.4 | growth hormone receptor | 102 | 1 | 1 | 0 | 0 | 2 | bta-miR-2462 | -0.23 |
| FCGR3A | ENST00000367969.3 | Fc fragment of IgG, low affinity IIIa, receptor (CD16a) | 5 | 1 | 0 | 1 | 0 | 1 | bta-miR-2462 | -0.23 |
| PXK | ENST00000463280.1 | PX domain containing serine/threonine kinase | 220 | 1 | 1 | 0 | 0 | 0 | bta-miR-2462 | -0.23 |
| PDGFRA | ENST00000257290.5 | platelet-derived growth factor receptor, alpha polypeptide | 39 | 2 | 1 | 1 | 0 | 1 | bta-miR-2462 | -0.23 |
| KDM4C | ENST00000381309.3 | lysine (K)-specific demethylase 4C | 160 | 1 | 1 | 0 | 0 | 0 | bta-miR-2462 | -0.23 |
| PARP8 | ENST00000503750.2 | poly (ADP-ribose) polymerase family, member 8 | 80 | 1 | 0 | 1 | 0 | 1 | bta-miR-2462 | -0.22 |
| APPL1 | ENST00000288266.3 | adaptor protein, phosphotyrosine interaction, PH domain and leucine zipper containing 1 | 908 | 1 | 1 | 0 | 0 | 0 | bta-miR-2462 | -0.22 |
| FCGR3B | ENST00000367964.2 | Fc fragment of IgG, low affinity IIIb, receptor (CD16b) | 5 | 1 | 0 | 1 | 0 | 1 | bta-miR-2462 | -0.22 |
| PLXDC2 | ENST00000377252.4 | plexin domain containing 2 | 506 | 3 | 2 | 1 | 0 | 2 | bta-miR-2462 | -0.22 |
| C16orf70 | ENST00000219139.3 | chromosome 16 open reading frame 70 | 485 | 1 | 0 | 1 | 0 | 0 | bta-miR-2462 | -0.22 |
| SNAPC3 | ENST00000380821.3 | small nuclear RNA activating complex, polypeptide 3, 50kDa | 2406 | 1 | 0 | 1 | 0 | 3 | bta-miR-2462 | -0.22 |
| ARL4C | ENST00000390645.2 | ADP-ribosylation factor-like 4C | 84 | 1 | 0 | 1 | 0 | 0 | bta-miR-2462 | -0.22 |
| ZNF586 | ENST00000396150.4 | zinc finger protein 586 | 137 | 3 | 0 | 3 | 0 | 1 | bta-miR-2462 | -0.22 |
| PDZD11 | ENST00000239666.4 | PDZ domain containing 11 | 144 | 1 | 0 | 0 | 1 | 0 | bta-miR-2462 | -0.22 |
| C18orf63 | ENST00000579455.1 | chromosome 18 open reading frame 63 | 5 | 2 | 1 | 1 | 0 | 0 | bta-miR-2462 | -0.22 |
| CRYL1 | ENST00000298248.7 | crystallin, lambda 1 | 158 | 1 | 0 | 0 | 1 | 0 | bta-miR-2462 | -0.22 |
| TXNDC15 | ENST00000358387.4 | thioredoxin domain containing 15 | 1181 | 1 | 0 | 1 | 0 | 0 | bta-miR-2462 | -0.22 |
| CCDC170 | ENST00000239374.7 | coiled-coil domain containing 170 | 15 | 4 | 0 | 2 | 2 | 0 | bta-miR-2462 | -0.22 |
| CTXN2 | ENST00000417307.2 | cortexin 2 | 5 | 1 | 0 | 0 | 1 | 0 | bta-miR-2462 | -0.22 |
| CNNM1 | ENST00000356713.4 | cyclin M1 | 45 | 1 | 0 | 1 | 0 | 1 | bta-miR-2462 | -0.22 |
| DYNLRB2 | ENST00000568035.1 | dynein, light chain, roadblock-type 2 | 21 | 1 | 0 | 1 | 0 | 0 | bta-miR-2462 | -0.22 |
| RYBP | ENST00000477973.2 | RING1 and YY1 binding protein | 342 | 1 | 0 | 1 | 0 | 3 | bta-miR-2462 | -0.22 |
| IFNA2 | ENST00000380206.2 | interferon, alpha 2 | 5 | 1 | 0 | 1 | 0 | 0 | bta-miR-2462 | -0.22 |
| IL36RN | ENST00000346807.3 | interleukin 36 receptor antagonist | 5 | 2 | 0 | 2 | 0 | 0 | bta-miR-2462 | -0.22 |
| RAVER2 | ENST00000371072.4 | ribonucleoprotein, PTB-binding 2 | 218 | 1 | 0 | 1 | 0 | 1 | bta-miR-2462 | -0.22 |
| C8orf37 | ENST00000286688.5 | chromosome 8 open reading frame 37 | 70 | 1 | 0 | 1 | 0 | 0 | bta-miR-2462 | -0.22 |
| C13orf35 | ENST00000356049.1 | chromosome 13 open reading frame 35 | 5 | 1 | 0 | 1 | 0 | 0 | bta-miR-2462 | -0.22 |
| RBM25 | ENST00000261973.7 | RNA binding motif protein 25 | 1190 | 2 | 0 | 0 | 2 | 1 | bta-miR-2462 | -0.22 |
| ATP2B1 | ENST00000261173.2 | ATPase, Ca++ transporting, plasma membrane 1 | 699 | 3 | 1 | 2 | 0 | 0 | bta-miR-2462 | -0.22 |
| USP15 | ENST00000353364.3 | ubiquitin specific peptidase 15 | 286 | 1 | 0 | 1 | 0 | 0 | bta-miR-2462 | -0.22 |
| STMND1 | ENST00000536551.1 | stathmin domain containing 1 | 45 | 1 | 0 | 1 | 0 | 0 | bta-miR-2462 | -0.22 |
| EPSTI1 | ENST00000313624.7 | epithelial stromal interaction 1 (breast) | 11 | 1 | 0 | 1 | 0 | 0 | bta-miR-2462 | -0.22 |
| SMIM21 | ENST00000579022.1 | small integral membrane protein 21 | 5 | 1 | 0 | 1 | 0 | 0 | bta-miR-2462 | -0.22 |
| SMIM17 | ENST00000598409.1 | small integral membrane protein 17 | 5 | 1 | 0 | 0 | 1 | 1 | bta-miR-2462 | -0.22 |
| EPHA6 | ENST00000470610.2 | EPH receptor A6 | 19 | 1 | 0 | 1 | 0 | 0 | bta-miR-2462 | -0.22 |
| SGCD | ENST00000435422.3 | sarcoglycan, delta (35kDa dystrophin-associated glycoprotein) | 39 | 5 | 1 | 2 | 2 | 1 | bta-miR-2462 | -0.22 |
| CABP5 | ENST00000293255.2 | calcium binding protein 5 | 5 | 2 | 0 | 2 | 0 | 0 | bta-miR-2462 | -0.22 |
| NTF4 | ENST00000451356.2 | neurotrophin 4 | 5 | 1 | 0 | 1 | 0 | 0 | bta-miR-2462 | -0.22 |
| RNF182 | ENST00000488300.1 | ring finger protein 182 | 1122 | 1 | 0 | 1 | 0 | 1 | bta-miR-2462 | -0.22 |
| TRIQK | ENST00000521988.1 | triple QxxK/R motif containing | 210 | 1 | 0 | 1 | 0 | 1 | bta-miR-2462 | -0.22 |
| ABCD2 | ENST00000308666.3 | ATP-binding cassette, sub-family D (ALD), member 2 | 5 | 1 | 1 | 0 | 0 | 4 | bta-miR-2462 | -0.21 |
| SRBD1 | ENST00000263736.4 | S1 RNA binding domain 1 | 33 | 1 | 0 | 1 | 0 | 0 | bta-miR-2462 | -0.21 |
| ZNF570 | ENST00000388801.3 | zinc finger protein 570 | 12 | 1 | 1 | 0 | 0 | 0 | bta-miR-2462 | -0.21 |
| AC115618.1 | ENST00000376775.2 | Uncharacterized protein; cDNA FLJ26048 fis, clone PRS02384 | 5 | 1 | 0 | 0 | 1 | 0 | bta-miR-2462 | -0.21 |
| LRIT3 | ENST00000594814.1 | leucine-rich repeat, immunoglobulin-like and transmembrane domains 3 | 5 | 1 | 0 | 1 | 0 | 1 | bta-miR-2462 | -0.21 |
| MAPRE3 | ENST00000233121.2 | microtubule-associated protein, RP/EB family, member 3 | 303 | 1 | 0 | 1 | 0 | 0 | bta-miR-2462 | -0.21 |
| MC2R | ENST00000327606.3 | melanocortin 2 receptor (adrenocorticotropic hormone) | 5 | 1 | 0 | 1 | 0 | 1 | bta-miR-2462 | -0.21 |
| METTL7B | ENST00000394252.3 | methyltransferase like 7B | 58 | 1 | 0 | 0 | 1 | 0 | bta-miR-2462 | -0.21 |
| EFHD1 | ENST00000264059.3 | EF-hand domain family, member D1 | 105 | 1 | 0 | 0 | 1 | 1 | bta-miR-2462 | -0.21 |
| OSBPL8 | ENST00000393249.2 | oxysterol binding protein-like 8 | 141 | 1 | 0 | 0 | 1 | 1 | bta-miR-2462 | -0.21 |
| GABARAP | ENST00000302386.5 | GABA(A) receptor-associated protein | 10137 | 1 | 0 | 1 | 0 | 0 | bta-miR-2462 | -0.21 |
| CCNH | ENST00000508855.1 | cyclin H | 250 | 2 | 0 | 1 | 1 | 0 | bta-miR-2462 | -0.21 |
| FAM135B | ENST00000395297.1 | family with sequence similarity 135, member B | 5 | 2 | 1 | 1 | 0 | 1 | bta-miR-2462 | -0.21 |
| HCAR1 | ENST00000432564.1 | hydroxycarboxylic acid receptor 1 | 38 | 2 | 0 | 2 | 0 | 0 | bta-miR-2462 | -0.21 |
| GALNT6 | ENST00000543196.2 | UDP-N-acetyl-alpha-D-galactosamine:polypeptide N-acetylgalactosaminyltransferase 6 (GalNAc-T6) | 83 | 1 | 0 | 1 | 0 | 1 | bta-miR-2462 | -0.21 |
| PICALM | ENST00000532317.1 | phosphatidylinositol binding clathrin assembly protein | 1173 | 2 | 1 | 1 | 0 | 0 | bta-miR-2462 | -0.21 |
| ELOVL6 | ENST00000394607.3 | ELOVL fatty acid elongase 6 | 901 | 1 | 0 | 1 | 0 | 4 | bta-miR-2462 | -0.21 |
| CRKL | ENST00000354336.3 | v-crk avian sarcoma virus CT10 oncogene homolog-like | 2142 | 1 | 0 | 1 | 0 | 0 | bta-miR-2462 | -0.21 |
| VSTM2A | ENST00000407838.3 | V-set and transmembrane domain containing 2A | 5 | 1 | 0 | 0 | 1 | 0 | bta-miR-2462 | -0.21 |
| ZDHHC15 | ENST00000373367.3 | zinc finger, DHHC-type containing 15 | 5 | 1 | 0 | 1 | 0 | 1 | bta-miR-2462 | -0.21 |
| KCNJ13 | ENST00000409779.1 | potassium inwardly-rectifying channel, subfamily J, member 13 | 5 | 1 | 0 | 1 | 0 | 3 | bta-miR-2462 | -0.21 |
| SMCO3 | ENST00000316048.2 | single-pass membrane protein with coiled-coil domains 3 | 5 | 1 | 0 | 1 | 0 | 0 | bta-miR-2462 | -0.21 |
| CASC4 | ENST00000299957.6 | cancer susceptibility candidate 4 | 5 | 1 | 1 | 0 | 0 | 0 | bta-miR-2462 | -0.21 |
| MRPL34 | ENST00000252602.1 | mitochondrial ribosomal protein L34 | 8223 | 1 | 0 | 1 | 0 | 0 | bta-miR-2462 | -0.21 |
| TUBB2A | ENST00000333628.3 | tubulin, beta 2A class IIa | 2337 | 1 | 0 | 0 | 1 | 0 | bta-miR-2462 | -0.21 |
| TGFBRAP1 | ENST00000393359.2 | transforming growth factor, beta receptor associated protein 1 | 435 | 2 | 0 | 1 | 1 | 1 | bta-miR-2462 | -0.21 |
| N4BP2L1 | ENST00000380139.4 | NEDD4 binding protein 2-like 1 | 92 | 1 | 0 | 1 | 0 | 0 | bta-miR-2462 | -0.21 |
| NKAPL | ENST00000343684.3 | NFKB activating protein-like | 5 | 1 | 0 | 1 | 0 | 0 | bta-miR-2462 | -0.21 |
| BCL7A | ENST00000538010.1 | B-cell CLL/lymphoma 7A | 96 | 1 | 1 | 0 | 0 | 0 | bta-miR-2462 | -0.21 |

**Predicted target mRNAs of mir-2359**

| Ortholog of target gene | Representative transcript | Gene name | 3P-seq tags + 5 | Total sites | 8mer sites | 7mer-m8 sites | 7mer-A1 sites | 6mer sites | Representative miRNA | Cumulative weighted context++ score |
| --- | --- | --- | --- | --- | --- | --- | --- | --- | --- | --- |
| RP4-758J18.2 | ENST00000444362.1 | HCG20425, isoform CRA_a; Uncharacterized protein; cDNA FLJ53815 | 283 | 2 | 1 | 1 | 0 | 0 | bta-miR-2359 | -1.17 |
| SOX6 | ENST00000316399.6 | SRY (sex determining region Y)-box 6 | 22 | 1* | 0 | 0 | 0 | 2 | bta-miR-2359 | -1 |
| CDC123 | ENST00000281141.4 | cell division cycle 123 | 9643 | 1 | 1 | 0 | 0 | 0 | bta-miR-2359 | -0.72 |
| SPINK13 | ENST00000512953.1 | serine peptidase inhibitor, Kazal type 13 (putative) | 725 | 1 | 1 | 0 | 0 | 0 | bta-miR-2359 | -0.71 |
| MRPS18B | ENST00000259873.4 | mitochondrial ribosomal protein S18B | 1667 | 2 | 1 | 0 | 1 | 0 | bta-miR-2359 | -0.68 |
| S100A7 | ENST00000368722.1 | S100 calcium binding protein A7 | 5 | 1 | 1 | 0 | 0 | 0 | bta-miR-2359 | -0.67 |
| CCT6B | ENST00000421975.3 | chaperonin containing TCP1, subunit 6B (zeta 2) | 203 | 1 | 1 | 0 | 0 | 0 | bta-miR-2359 | -0.64 |
| S100A7L2 | ENST00000368725.2 | S100 calcium binding protein A7-like 2 | 0 | 1 | 1 | 0 | 0 | 0 | bta-miR-2359 | -0.62 |
| COX6A2 | ENST00000287490.4 | cytochrome c oxidase subunit VIa polypeptide 2 | 5 | 1 | 1 | 0 | 0 | 0 | bta-miR-2359 | -0.61 |
| ELANE | ENST00000590230.1 | elastase, neutrophil expressed | 5 | 1 | 1 | 0 | 0 | 0 | bta-miR-2359 | -0.56 |
| OSTN | ENST00000445281.1 | osteocrin | 14 | 3 | 1 | 1 | 1 | 0 | bta-miR-2359 | -0.56 |
| RP11-67H2.1 | ENST00000521500.2 | Uncharacterized protein | 5 | 1 | 1 | 0 | 0 | 0 | bta-miR-2359 | -0.54 |
| TMEFF2 | ENST00000392314.1 | transmembrane protein with EGF-like and two follistatin-like domains 2 | 179 | 2 | 2 | 0 | 0 | 0 | bta-miR-2359 | -0.53 |
| LAMTOR3 | ENST00000499666.2 | late endosomal/lysosomal adaptor, MAPK and MTOR activator 3 | 67 | 1 | 1 | 0 | 0 | 1 | bta-miR-2359 | -0.52 |
| SERPINB5 | ENST00000382771.4 | serpin peptidase inhibitor, clade B (ovalbumin), member 5 | 295 | 2 | 1 | 0 | 1 | 0 | bta-miR-2359 | -0.51 |
| DCAF4L2 | ENST00000319675.3 | DDB1 and CUL4 associated factor 4-like 2 | 10 | 1 | 0 | 1 | 0 | 0 | bta-miR-2359 | -0.5 |
| TFEC | ENST00000265440.7 | transcription factor EC | 5 | 4 | 0 | 2 | 2 | 0 | bta-miR-2359 | -0.48 |
| CALD1 | ENST00000361388.2 | caldesmon 1 | 1013 | 6 | 1 | 1 | 4 | 1 | bta-miR-2359 | -0.47 |
| KCNJ8 | ENST00000240662.2 | potassium inwardly-rectifying channel, subfamily J, member 8 | 53 | 1 | 1 | 0 | 0 | 0 | bta-miR-2359 | -0.44 |
| SLC35B1 | ENST00000240333.6 | solute carrier family 35, member B1 | 119 | 1 | 1 | 0 | 0 | 0 | bta-miR-2359 | -0.44 |
| TRPM1 | ENST00000397795.2 | transient receptor potential cation channel, subfamily M, member 1 | 5 | 1 | 1 | 0 | 0 | 0 | bta-miR-2359 | -0.43 |
| PTHLH | ENST00000395872.1 | parathyroid hormone-like hormone | 32 | 1 | 1 | 0 | 0 | 0 | bta-miR-2359 | -0.43 |
| RGS7BP | ENST00000334025.2 | regulator of G-protein signaling 7 binding protein | 5 | 4 | 2 | 0 | 2 | 3 | bta-miR-2359 | -0.42 |
| TMEM229A | ENST00000455783.1 | transmembrane protein 229A | 5 | 1 | 1 | 0 | 0 | 0 | bta-miR-2359 | -0.42 |
| PRR26 | ENST00000381489.5 | proline rich 26 | 5 | 1 | 0 | 1 | 0 | 0 | bta-miR-2359 | -0.42 |
| IQUB | ENST00000466202.1 | IQ motif and ubiquitin domain containing | 5 | 1 | 1 | 0 | 0 | 0 | bta-miR-2359 | -0.41 |
| GADD45A | ENST00000370986.4 | growth arrest and DNA-damage-inducible, alpha | 689 | 1 | 1 | 0 | 0 | 0 | bta-miR-2359 | -0.41 |
| NUP62CL | ENST00000372461.3 | nucleoporin 62kDa C-terminal like | 73 | 1 | 1 | 0 | 0 | 1 | bta-miR-2359 | -0.41 |
| IL7 | ENST00000263851.4 | interleukin 7 | 5 | 1 | 1 | 0 | 0 | 0 | bta-miR-2359 | -0.41 |
| PTPN22 | ENST00000460620.1 | protein tyrosine phosphatase, non-receptor type 22 (lymphoid) | 5 | 1 | 1 | 0 | 0 | 0 | bta-miR-2359 | -0.4 |
| ZNF140 | ENST00000440550.2 | zinc finger protein 140 | 179 | 2 | 1 | 1 | 0 | 0 | bta-miR-2359 | -0.4 |
| ASCL2 | ENST00000331289.4 | achaete-scute complex homolog 2 (Drosophila) | 5 | 1 | 1 | 0 | 0 | 0 | bta-miR-2359 | -0.4 |
| CRTAC1 | ENST00000298819.4 | cartilage acidic protein 1 | 13 | 1 | 1 | 0 | 0 | 0 | bta-miR-2359 | -0.4 |
| EMD | ENST00000369842.4 | emerin | 1771 | 1 | 1 | 0 | 0 | 0 | bta-miR-2359 | -0.4 |
| BRD8 | ENST00000230901.5 | bromodomain containing 8 | 5 | 1 | 1 | 0 | 0 | 1 | bta-miR-2359 | -0.39 |
| PLAC9 | ENST00000372263.3 | placenta-specific 9 | 7 | 1 | 1 | 0 | 0 | 0 | bta-miR-2359 | -0.39 |
| RAB14 | ENST00000373840.4 | RAB14, member RAS oncogene family | 586 | 1 | 1 | 0 | 0 | 0 | bta-miR-2359 | -0.39 |
| ARL5C | ENST00000269586.7 | ADP-ribosylation factor-like 5C | 2 | 1 | 1 | 0 | 0 | 0 | bta-miR-2359 | -0.39 |
| CTSF | ENST00000310325.5 | cathepsin F | 382 | 1 | 1 | 0 | 0 | 0 | bta-miR-2359 | -0.38 |
| HEATR5A | ENST00000389961.3 | HEAT repeat containing 5A | 367 | 2 | 2 | 0 | 0 | 1 | bta-miR-2359 | -0.38 |
| IFI16 | ENST00000368131.4 | interferon, gamma-inducible protein 16 | 25 | 1 | 1 | 0 | 0 | 0 | bta-miR-2359 | -0.38 |
| WIF1 | ENST00000286574.4 | WNT inhibitory factor 1 | 28 | 1 | 1 | 0 | 0 | 0 | bta-miR-2359 | -0.38 |
| ABCD3 | ENST00000394233.2 | ATP-binding cassette, sub-family D (ALD), member 3 | 53 | 1 | 1 | 0 | 0 | 0 | bta-miR-2359 | -0.38 |
| ELL | ENST00000262809.4 | elongation factor RNA polymerase II | 250 | 1 | 1 | 0 | 0 | 0 | bta-miR-2359 | -0.37 |
| C1orf227 | ENST00000332912.3 | chromosome 1 open reading frame 227 | 5 | 1 | 0 | 1 | 0 | 0 | bta-miR-2359 | -0.37 |
| LGSN | ENST00000370658.5 | lengsin, lens protein with glutamine synthetase domain | 21 | 1 | 1 | 0 | 0 | 0 | bta-miR-2359 | -0.37 |
| CLEC1A | ENST00000315330.4 | C-type lectin domain family 1, member A | 5 | 1 | 1 | 0 | 0 | 0 | bta-miR-2359 | -0.37 |
| PTN | ENST00000393083.2 | pleiotrophin | 11 | 1 | 1 | 0 | 0 | 0 | bta-miR-2359 | -0.37 |
| MS4A14 | ENST00000395001.1 | membrane-spanning 4-domains, subfamily A, member 14 | 5 | 2 | 0 | 2 | 0 | 0 | bta-miR-2359 | -0.36 |
| CLEC9A | ENST00000355819.1 | C-type lectin domain family 9, member A | 5 | 1 | 1 | 0 | 0 | 0 | bta-miR-2359 | -0.36 |
| DOC2A | ENST00000350119.4 | double C2-like domains, alpha | 10 | 2 | 1 | 1 | 0 | 0 | bta-miR-2359 | -0.36 |
| OVCA2 | ENST00000572195.1 | ovarian tumor suppressor candidate 2 | 2807 | 1 | 1 | 0 | 0 | 0 | bta-miR-2359 | -0.36 |
| RFX1 | ENST00000254325.4 | regulatory factor X, 1 (influences HLA class II expression) | 44 | 1 | 1 | 0 | 0 | 1 | bta-miR-2359 | -0.35 |
| ADH7 | ENST00000437033.2 | alcohol dehydrogenase 7 (class IV), mu or sigma polypeptide | 5 | 1 | 1 | 0 | 0 | 0 | bta-miR-2359 | -0.34 |
| ELOVL6 | ENST00000394607.3 | ELOVL fatty acid elongase 6 | 901 | 2 | 1 | 0 | 1 | 0 | bta-miR-2359 | -0.34 |
| CAMK2D | ENST00000296402.5 | calcium/calmodulin-dependent protein kinase II delta | 938 | 2 | 0 | 2 | 0 | 1 | bta-miR-2359 | -0.34 |
| MRPS35 | ENST00000538315.1 | mitochondrial ribosomal protein S35 | 3092 | 1 | 1 | 0 | 0 | 0 | bta-miR-2359 | -0.34 |
| CCDC47 | ENST00000225726.5 | coiled-coil domain containing 47 | 1484 | 1 | 1 | 0 | 0 | 1 | bta-miR-2359 | -0.33 |
| CEP57L1 | ENST00000368968.2 | centrosomal protein 57kDa-like 1 | 90 | 1 | 1 | 0 | 0 | 1 | bta-miR-2359 | -0.33 |
| RPL36 | ENST00000347512.3 | ribosomal protein L36 | 1323 | 1 | 0 | 0 | 1 | 1 | bta-miR-2359 | -0.33 |
| CCDC73 | ENST00000531481.1 | coiled-coil domain containing 73 | 5 | 2 | 1 | 1 | 0 | 0 | bta-miR-2359 | -0.33 |
| MRPS28 | ENST00000521605.1 | mitochondrial ribosomal protein S28 | 2728 | 1 | 0 | 1 | 0 | 0 | bta-miR-2359 | -0.33 |
| PRMT6 | ENST00000370078.1 | protein arginine methyltransferase 6 | 270 | 2 | 1 | 0 | 1 | 1 | bta-miR-2359 | -0.33 |
| NFXL1 | ENST00000381538.3 | nuclear transcription factor, X-box binding-like 1 | 71 | 1 | 1 | 0 | 0 | 1 | bta-miR-2359 | -0.33 |
| ZNF674 | ENST00000523374.1 | zinc finger protein 674 | 7 | 1 | 1 | 0 | 0 | 1 | bta-miR-2359 | -0.32 |
| RGS13 | ENST00000391995.2 | regulator of G-protein signaling 13 | 5 | 1 | 0 | 1 | 0 | 2 | bta-miR-2359 | -0.32 |
| ARHGAP30 | ENST00000368016.3 | Rho GTPase activating protein 30 | 5 | 1 | 1 | 0 | 0 | 0 | bta-miR-2359 | -0.32 |
| CCL16 | ENST00000293275.3 | chemokine (C-C motif) ligand 16 | 5 | 1 | 0 | 1 | 0 | 0 | bta-miR-2359 | -0.32 |
| CAV1 | ENST00000405348.1 | caveolin 1, caveolae protein, 22kDa | 415 | 1 | 1 | 0 | 0 | 0 | bta-miR-2359 | -0.31 |
| S100A7A | ENST00000368729.4 | S100 calcium binding protein A7A | 5 | 1 | 1 | 0 | 0 | 0 | bta-miR-2359 | -0.31 |
| H2AFZ | ENST00000296417.5 | H2A histone family, member Z | 927 | 1 | 0 | 1 | 0 | 0 | bta-miR-2359 | -0.31 |
| LIN7C | ENST00000278193.2 | lin-7 homolog C (C. elegans) | 1701 | 3 | 1 | 1 | 1 | 2 | bta-miR-2359 | -0.31 |
| ST6GALNAC3 | ENST00000328299.3 | ST6 (alpha-N-acetyl-neuraminyl-2,3-beta-galactosyl-1,3)-N-acetylgalactosaminide alpha-2,6-sialyltransferase 3 | 41 | 6 | 1 | 3 | 2 | 0 | bta-miR-2359 | -0.31 |
| SERPINB10 | ENST00000238508.3 | serpin peptidase inhibitor, clade B (ovalbumin), member 10 | 5 | 1 | 0 | 1 | 0 | 0 | bta-miR-2359 | -0.3 |
| CCR2 | ENST00000400888.2 | chemokine (C-C motif) receptor 2 | 5 | 2 | 0 | 0 | 2 | 0 | bta-miR-2359 | -0.3 |
| FGF2 | ENST00000264498.3 | fibroblast growth factor 2 (basic) | 123 | 2 | 1 | 1 | 0 | 1 | bta-miR-2359 | -0.3 |
| RP11-366L20.2 | ENST00000356215.2 | Uncharacterized protein | 5 | 2 | 2 | 0 | 0 | 0 | bta-miR-2359 | -0.3 |
| SAMD12 | ENST00000409003.4 | sterile alpha motif domain containing 12 | 57 | 4 | 1 | 1 | 2 | 0 | bta-miR-2359 | -0.3 |
| ARL13B | ENST00000535334.1 | ADP-ribosylation factor-like 13B | 23 | 1 | 1 | 0 | 0 | 0 | bta-miR-2359 | -0.3 |
| OLFML1 | ENST00000329293.3 | olfactomedin-like 1 | 5 | 1 | 1 | 0 | 0 | 0 | bta-miR-2359 | -0.3 |
| DAP | ENST00000230895.6 | death-associated protein | 8552 | 1 | 1 | 0 | 0 | 0 | bta-miR-2359 | -0.3 |
| TES | ENST00000358204.4 | testis derived transcript (3 LIM domains) | 34 | 1 | 1 | 0 | 0 | 0 | bta-miR-2359 | -0.3 |
| C7orf41 | ENST00000324453.8 | chromosome 7 open reading frame 41 | 235 | 2 | 1 | 1 | 0 | 1 | bta-miR-2359 | -0.29 |
| EYS | ENST00000393380.2 | eyes shut homolog (Drosophila) | 5 | 2 | 1 | 1 | 0 | 1 | bta-miR-2359 | -0.29 |
| HIST1H2BF | ENST00000359985.1 | histone cluster 1, H2bf | 16 | 1 | 1 | 0 | 0 | 0 | bta-miR-2359 | -0.29 |
| TMSB15B | ENST00000540220.1 | thymosin beta 15B | 211 | 1 | 0 | 0 | 1 | 1 | bta-miR-2359 | -0.28 |
| C6orf120 | ENST00000332290.2 | chromosome 6 open reading frame 120 | 1415 | 3 | 0 | 2 | 1 | 0 | bta-miR-2359 | -0.28 |
| KBTBD3 | ENST00000534815.1 | kelch repeat and BTB (POZ) domain containing 3 | 5 | 1 | 1 | 0 | 0 | 0 | bta-miR-2359 | -0.28 |
| UBE2E3 | ENST00000410062.4 | ubiquitin-conjugating enzyme E2E 3 | 15 | 1 | 1 | 0 | 0 | 0 | bta-miR-2359 | -0.28 |
| BCL11A | ENST00000356842.4 | B-cell CLL/lymphoma 11A (zinc finger protein) | 401 | 1 | 1 | 0 | 0 | 0 | bta-miR-2359 | -0.28 |
| ZNF169 | ENST00000480716.1 | zinc finger protein 169 | 12 | 1 | 0 | 1 | 0 | 1 | bta-miR-2359 | -0.28 |
| ZNF788 | ENST00000430298.2 | zinc finger family member 788 | 16 | 1 | 0 | 1 | 0 | 1 | bta-miR-2359 | -0.28 |
| AMZ1 | ENST00000312371.4 | archaelysin family metallopeptidase 1 | 5 | 1 | 1 | 0 | 0 | 0 | bta-miR-2359 | -0.28 |
| MOB1B | ENST00000309395.2 | MOB kinase activator 1B | 54 | 2 | 1 | 1 | 0 | 0 | bta-miR-2359 | -0.28 |
| CSRP2 | ENST00000311083.5 | cysteine and glycine-rich protein 2 | 38 | 1 | 0 | 1 | 0 | 0 | bta-miR-2359 | -0.28 |
| RAD9B | ENST00000409425.1 | RAD9 homolog B (S. pombe) | 10 | 2 | 1 | 0 | 1 | 1 | bta-miR-2359 | -0.28 |
| ABCC12 | ENST00000416054.1 | ATP-binding cassette, sub-family C (CFTR/MRP), member 12 | 5 | 1 | 1 | 0 | 0 | 1 | bta-miR-2359 | -0.28 |
| NDRG3 | ENST00000373803.2 | NDRG family member 3 | 960 | 1 | 1 | 0 | 0 | 0 | bta-miR-2359 | -0.28 |
| CASS4 | ENST00000371336.3 | Cas scaffolding protein family member 4 | 49 | 1 | 1 | 0 | 0 | 0 | bta-miR-2359 | -0.28 |
| UGT2A1 | ENST00000503640.1 | UDP glucuronosyltransferase 2 family, polypeptide A1, complex locus | 5 | 1 | 0 | 0 | 1 | 1 | bta-miR-2359 | -0.27 |
| UGT2A2 | ENST00000457664.2 | UDP glucuronosyltransferase 2 family, polypeptide A2 | 5 | 1 | 0 | 0 | 1 | 1 | bta-miR-2359 | -0.27 |
| JAK2 | ENST00000381652.3 | Janus kinase 2 | 17 | 3 | 1 | 1 | 1 | 0 | bta-miR-2359 | -0.27 |
| SMIM3 | ENST00000526627.1 | small integral membrane protein 3 | 3180 | 1 | 0 | 1 | 0 | 0 | bta-miR-2359 | -0.27 |
| RARB | ENST00000437042.2 | retinoic acid receptor, beta | 48 | 1 | 0 | 1 | 0 | 0 | bta-miR-2359 | -0.27 |
| CTD-2370N5.3 | ENST00000578584.1 | | 5 | 1 | 0 | 1 | 0 | 1 | bta-miR-2359 | -0.27 |
| ZNF25 | ENST00000302609.7 | zinc finger protein 25 | 11 | 1 | 1 | 0 | 0 | 0 | bta-miR-2359 | -0.27 |
| TRAPPC2 | ENST00000453655.2 | trafficking protein particle complex 2 | 589 | 1 | 0 | 1 | 0 | 0 | bta-miR-2359 | -0.27 |
| AL627171.2 | ENST00000595378.1 | HCG1786899; PRO2610; Uncharacterized protein | 5 | 1 | 0 | 1 | 0 | 0 | bta-miR-2359 | -0.27 |
| C20orf85 | ENST00000371168.3 | chromosome 20 open reading frame 85 | 5 | 1 | 0 | 1 | 0 | 0 | bta-miR-2359 | -0.27 |
| PPP3CB | ENST00000360663.5 | protein phosphatase 3, catalytic subunit, beta isozyme | 105 | 2 | 1 | 1 | 0 | 0 | bta-miR-2359 | -0.27 |
| PCDHA1 | ENST00000504120.2 | protocadherin alpha 1 | 86 | 2 | 0 | 2 | 0 | 0 | bta-miR-2359 | -0.27 |
| SLCO1A2 | ENST00000307378.6 | solute carrier organic anion transporter family, member 1A2 | 5 | 1 | 1 | 0 | 0 | 0 | bta-miR-2359 | -0.27 |
| RAB9B | ENST00000243298.2 | RAB9B, member RAS oncogene family | 21 | 1 | 1 | 0 | 0 | 1 | bta-miR-2359 | -0.27 |
| CHCHD5 | ENST00000409719.1 | coiled-coil-helix-coiled-coil-helix domain containing 5 | 1577 | 2 | 0 | 0 | 2 | 0 | bta-miR-2359 | -0.26 |
| ENKUR | ENST00000376363.1 | enkurin, TRPC channel interacting protein | 7 | 1 | 0 | 1 | 0 | 0 | bta-miR-2359 | -0.26 |
| LRRC10B | ENST00000378075.2 | leucine rich repeat containing 10B | 5 | 1 | 1 | 0 | 0 | 0 | bta-miR-2359 | -0.26 |
| NKIRAS2 | ENST00000307641.5 | NFKB inhibitor interacting Ras-like 2 | 9685 | 1 | 1 | 0 | 0 | 0 | bta-miR-2359 | -0.26 |
| BHLHB9 | ENST00000372735.1 | basic helix-loop-helix domain containing, class B, 9 | 5 | 1 | 1 | 0 | 0 | 0 | bta-miR-2359 | -0.26 |
| PRRG1 | ENST00000378628.4 | proline rich Gla (G-carboxyglutamic acid) 1 | 1122 | 2 | 1 | 0 | 1 | 1 | bta-miR-2359 | -0.26 |
| ZSCAN12 | ENST00000361028.1 | zinc finger and SCAN domain containing 12 | 117 | 1 | 1 | 0 | 0 | 0 | bta-miR-2359 | -0.26 |
| AP1S2 | ENST00000329235.2 | adaptor-related protein complex 1, sigma 2 subunit | 357 | 2 | 0 | 2 | 0 | 0 | bta-miR-2359 | -0.25 |
| ARFIP1 | ENST00000451320.2 | ADP-ribosylation factor interacting protein 1 | 275 | 1 | 1 | 0 | 0 | 0 | bta-miR-2359 | -0.25 |
| CRYBA1 | ENST00000225387.3 | crystallin, beta A1 | 5 | 1 | 0 | 0 | 1 | 0 | bta-miR-2359 | -0.25 |
| CACNG8 | ENST00000270458.2 | calcium channel, voltage-dependent, gamma subunit 8 | 5 | 2 | 1 | 1 | 0 | 1 | bta-miR-2359 | -0.25 |
| ARHGEF33 | ENST00000409978.1 | Rho guanine nucleotide exchange factor (GEF) 33 | 9 | 1 | 1 | 0 | 0 | 0 | bta-miR-2359 | -0.25 |
| FUT9 | ENST00000302103.5 | fucosyltransferase 9 (alpha (1,3) fucosyltransferase) | 5 | 3 | 2 | 1 | 0 | 3 | bta-miR-2359 | -0.25 |
| RAB3GAP2 | ENST00000358951.2 | RAB3 GTPase activating protein subunit 2 (non-catalytic) | 461 | 2 | 1 | 1 | 0 | 0 | bta-miR-2359 | -0.25 |
| PTGR2 | ENST00000555661.1 | prostaglandin reductase 2 | 244 | 2 | 0 | 0 | 2 | 0 | bta-miR-2359 | -0.25 |
| ZNF705G | ENST00000400156.4 | zinc finger protein 705G | 5 | 1 | 1 | 0 | 0 | 1 | bta-miR-2359 | -0.25 |
| FAM216A | ENST00000377673.5 | family with sequence similarity 216, member A | 1234 | 1 | 0 | 0 | 1 | 0 | bta-miR-2359 | -0.25 |
| SPAG6 | ENST00000313311.6 | sperm associated antigen 6 | 23 | 1 | 1 | 0 | 0 | 0 | bta-miR-2359 | -0.25 |
| CNFN | ENST00000222032.5 | cornifelin | 40 | 1 | 0 | 0 | 1 | 0 | bta-miR-2359 | -0.25 |
| PTP4A2 | ENST00000602725.1 | protein tyrosine phosphatase type IVA, member 2 | 5 | 1 | 1 | 0 | 0 | 0 | bta-miR-2359 | -0.25 |
| WFDC6 | ENST00000600168.1 | WAP four-disulfide core domain 6 | 5 | 1 | 0 | 1 | 0 | 0 | bta-miR-2359 | -0.24 |
| CDK7 | ENST00000256443.3 | cyclin-dependent kinase 7 | 273 | 1 | 0 | 0 | 1 | 0 | bta-miR-2359 | -0.24 |
| KIF14 | ENST00000367350.4 | kinesin family member 14 | 451 | 1 | 1 | 0 | 0 | 0 | bta-miR-2359 | -0.24 |
| TNFSF13 | ENST00000338784.4 | tumor necrosis factor (ligand) superfamily, member 13 | 5 | 1 | 1 | 0 | 0 | 0 | bta-miR-2359 | -0.24 |
| CCNDBP1 | ENST00000300213.4 | cyclin D-type binding-protein 1 | 1136 | 1 | 0 | 1 | 0 | 0 | bta-miR-2359 | -0.24 |
| NDUFS2 | ENST00000367993.3 | NADH dehydrogenase (ubiquinone) Fe-S protein 2, 49kDa (NADH-coenzyme Q reductase) | 843 | 1 | 0 | 0 | 1 | 0 | bta-miR-2359 | -0.24 |
| TMEM17 | ENST00000335390.5 | transmembrane protein 17 | 10 | 1 | 0 | 0 | 1 | 0 | bta-miR-2359 | -0.24 |
| LMOD3 | ENST00000420581.2 | leiomodin 3 (fetal) | 5 | 1 | 1 | 0 | 0 | 0 | bta-miR-2359 | -0.24 |
| PGBD1 | ENST00000259883.3 | piggyBac transposable element derived 1 | 452 | 1 | 0 | 1 | 0 | 0 | bta-miR-2359 | -0.24 |
| AC010441.1 | ENST00000600109.1 | | 3180 | 1 | 0 | 1 | 0 | 0 | bta-miR-2359 | -0.23 |
| SLC2A12 | ENST00000275230.5 | solute carrier family 2 (facilitated glucose transporter), member 12 | 136 | 1 | 1 | 0 | 0 | 1 | bta-miR-2359 | -0.23 |
| NPM2 | ENST00000381530.5 | nucleophosmin/nucleoplasmin 2 | 136 | 2 | 0 | 1 | 1 | 0 | bta-miR-2359 | -0.23 |
| HOPX | ENST00000420433.1 | HOP homeobox | 55 | 1 | 0 | 1 | 0 | 0 | bta-miR-2359 | -0.23 |
| PLEKHO1 | ENST00000369124.4 | pleckstrin homology domain containing, family O member 1 | 144 | 1 | 0 | 1 | 0 | 0 | bta-miR-2359 | -0.23 |
| ZNF583 | ENST00000333201.9 | zinc finger protein 583 | 24 | 1 | 1 | 0 | 0 | 0 | bta-miR-2359 | -0.23 |
| GPR174 | ENST00000276077.1 | G protein-coupled receptor 174 | 5 | 1 | 0 | 1 | 0 | 0 | bta-miR-2359 | -0.23 |
| HMG20A | ENST00000336216.4 | high mobility group 20A | 391 | 1 | 1 | 0 | 0 | 0 | bta-miR-2359 | -0.23 |
| REG3G | ENST00000393897.2 | regenerating islet-derived 3 gamma | 5 | 1 | 0 | 0 | 1 | 0 | bta-miR-2359 | -0.23 |
| HSPBAP1 | ENST00000383659.1 | HSPB (heat shock 27kDa) associated protein 1 | 22 | 1 | 1 | 0 | 0 | 0 | bta-miR-2359 | -0.23 |
| RBM3 | ENST00000376759.3 | RNA binding motif (RNP1, RRM) protein 3 | 1325 | 1 | 0 | 1 | 0 | 0 | bta-miR-2359 | -0.23 |
| OR12D3 | ENST00000396806.3 | olfactory receptor, family 12, subfamily D, member 3 | 5 | 1 | 0 | 1 | 0 | 0 | bta-miR-2359 | -0.23 |
| DET1 | ENST00000564406.1 | de-etiolated homolog 1 (Arabidopsis) | 5 | 1 | 1 | 0 | 0 | 0 | bta-miR-2359 | -0.23 |
| HNRNPAB | ENST00000358344.3 | heterogeneous nuclear ribonucleoprotein A/B | 2641 | 1 | 0 | 1 | 0 | 0 | bta-miR-2359 | -0.23 |
| RSRC1 | ENST00000464171.1 | arginine/serine-rich coiled-coil 1 | 642 | 2 | 0 | 1 | 1 | 0 | bta-miR-2359 | -0.23 |
| ARPP21 | ENST00000428373.1 | cAMP-regulated phosphoprotein, 21kDa | 5 | 1 | 1 | 0 | 0 | 0 | bta-miR-2359 | -0.23 |
| GULP1 | ENST00000409843.1 | GULP, engulfment adaptor PTB domain containing 1 | 196 | 1 | 1 | 0 | 0 | 0 | bta-miR-2359 | -0.23 |
| ZIC3 | ENST00000287538.5 | Zic family member 3 | 9 | 1 | 0 | 1 | 0 | 1 | bta-miR-2359 | -0.22 |
| KIDINS220 | ENST00000256707.3 | kinase D-interacting substrate, 220kDa | 87 | 2 | 1 | 0 | 1 | 0 | bta-miR-2359 | -0.22 |
| TBC1D14 | ENST00000448507.1 | TBC1 domain family, member 14 | 1583 | 1 | 1 | 0 | 0 | 0 | bta-miR-2359 | -0.22 |
| TNFSF12-TNFSF13 | ENST00000293826.4 | TNFSF12-TNFSF13 readthrough | 5 | 1 | 1 | 0 | 0 | 0 | bta-miR-2359 | -0.22 |
| BTN3A2 | ENST00000396948.1 | butyrophilin, subfamily 3, member A2 | 5 | 1 | 0 | 1 | 0 | 0 | bta-miR-2359 | -0.22 |
| PPP1R2 | ENST00000328432.3 | protein phosphatase 1, regulatory (inhibitor) subunit 2 | 8 | 1 | 1 | 0 | 0 | 0 | bta-miR-2359 | -0.22 |
| C9orf170 | ENST00000375941.2 | chromosome 9 open reading frame 170 | 5 | 1 | 0 | 1 | 0 | 1 | bta-miR-2359 | -0.22 |
| LYRM1 | ENST00000396052.2 | LYR motif containing 1 | 405 | 1 | 0 | 1 | 0 | 0 | bta-miR-2359 | -0.22 |
| KLHL13 | ENST00000371882.1 | kelch-like family member 13 | 13 | 1 | 1 | 0 | 0 | 0 | bta-miR-2359 | -0.22 |
| MZT1 | ENST00000377818.3 | mitotic spindle organizing protein 1 | 157 | 1 | 0 | 1 | 0 | 0 | bta-miR-2359 | -0.22 |
| MYCL | ENST00000372815.1 | v-myc avian myelocytomatosis viral oncogene lung carcinoma derived homolog | 11 | 1 | 0 | 1 | 0 | 0 | bta-miR-2359 | -0.22 |
| STAC | ENST00000273183.3 | SH3 and cysteine rich domain | 172 | 1 | 1 | 0 | 0 | 0 | bta-miR-2359 | -0.22 |
| PXMP2 | ENST00000317479.3 | peroxisomal membrane protein 2, 22kDa | 3834 | 1 | 0 | 0 | 1 | 0 | bta-miR-2359 | -0.22 |
| ZNF597 | ENST00000301744.4 | zinc finger protein 597 | 159 | 1 | 0 | 1 | 0 | 1 | bta-miR-2359 | -0.22 |
| GIMAP1 | ENST00000307194.5 | GTPase, IMAP family member 1 | 5 | 1 | 0 | 1 | 0 | 0 | bta-miR-2359 | -0.22 |
| KLF12 | ENST00000377669.2 | Kruppel-like factor 12 | 251 | 4 | 1 | 1 | 2 | 3 | bta-miR-2359 | -0.22 |
| ARGLU1 | ENST00000400198.3 | arginine and glutamate rich 1 | 9450 | 1 | 0 | 1 | 0 | 1 | bta-miR-2359 | -0.22 |
| TADA1 | ENST00000367874.4 | transcriptional adaptor 1 | 160 | 1 | 0 | 0 | 1 | 1 | bta-miR-2359 | -0.21 |
| TPCN1 | ENST00000335509.6 | two pore segment channel 1 | 401 | 1 | 1 | 0 | 0 | 0 | bta-miR-2359 | -0.21 |
| JPH2 | ENST00000372980.3 | junctophilin 2 | 76 | 1 | 1 | 0 | 0 | 0 | bta-miR-2359 | -0.21 |
| AIM2 | ENST00000368130.4 | absent in melanoma 2 | 5 | 1 | 0 | 0 | 1 | 0 | bta-miR-2359 | -0.21 |
| FAM154A | ENST00000380534.4 | family with sequence similarity 154, member A | 5 | 1 | 0 | 0 | 1 | 0 | bta-miR-2359 | -0.21 |
| PXDC1 | ENST00000380283.4 | PX domain containing 1 | 1727 | 1 | 0 | 1 | 0 | 0 | bta-miR-2359 | -0.21 |
| HSPB7 | ENST00000411503.1 | heat shock 27kDa protein family, member 7 (cardiovascular) | 5 | 1 | 1 | 0 | 0 | 0 | bta-miR-2359 | -0.21 |
| KLK6 | ENST00000376851.3 | kallikrein-related peptidase 6 | 5 | 1 | 0 | 1 | 0 | 0 | bta-miR-2359 | -0.21 |
| MANEA | ENST00000358812.4 | mannosidase, endo-alpha | 41 | 3 | 1 | 1 | 1 | 0 | bta-miR-2359 | -0.21 |
| DCP2 | ENST00000389063.2 | decapping mRNA 2 | 155 | 2 | 1 | 1 | 0 | 1 | bta-miR-2359 | -0.21 |
| COPS2 | ENST00000388901.5 | COP9 signalosome subunit 2 | 11120 | 1 | 0 | 0 | 1 | 1 | bta-miR-2359 | -0.21 |

**Predicted target mRNAs of mir-2430**

| Ortholog of target gene | Representative transcript | Gene name | 3P-seq tags + 5 | Total sites | 8mer sites | 7mer-m8 sites | 7mer-A1 sites | 6mer sites | Representative miRNA | Cumulative weighted context++ score |
| --- | --- | --- | --- | --- | --- | --- | --- | --- | --- | --- |
| GIPC3 | ENST00000322315.5 | GIPC PDZ domain containing family, member 3 | 48 | 14 | 4 | 3 | 7 | 8 | bta-miR-2430 | -2.13 |
| LAIR1 | ENST00000391743.3 | leukocyte-associated immunoglobulin-like receptor 1 | 5 | 4 | 1 | 3 | 0 | 1 | bta-miR-2430 | -1.33 |
| ZNF831 | ENST00000371030.2 | zinc finger protein 831 | 5 | 1 | 1 | 0 | 0 | 4 | bta-miR-2430 | -1.17 |
| C15orf32 | ENST00000556865.1 | chromosome 15 open reading frame 32 | 5 | 1 | 1 | 0 | 0 | 0 | bta-miR-2430 | -1.13 |
| RHOJ | ENST00000316754.3 | ras homolog family member J | 118 | 2 | 1 | 1 | 0 | 1 | bta-miR-2430 | -1.12 |
| RP11-127H5.1 | ENST00000521923.1 | Uncharacterized protein | 5 | 1 | 1 | 0 | 0 | 0 | bta-miR-2430 | -0.95 |
| FOXJ3 | ENST00000372571.1 | forkhead box J3 | 329 | 4 | 1 | 1 | 2 | 0 | bta-miR-2430 | -0.9 |
| KLK14 | ENST00000391802.1 | kallikrein-related peptidase 14 | 5 | 2 | 0 | 2 | 0 | 0 | bta-miR-2430 | -0.86 |
| PRSS33 | ENST00000576886.1 | protease, serine, 33 | 5 | 3 | 2 | 1 | 0 | 0 | bta-miR-2430 | -0.86 |
| PNMA6A | ENST00000453825.2 | paraneoplastic Ma antigen family member 6A | 5 | 1 | 1 | 0 | 0 | 0 | bta-miR-2430 | -0.85 |
| TEX264 | ENST00000341333.5 | testis expressed 264 | 33 | 2 | 1 | 1 | 0 | 1 | bta-miR-2430 | -0.83 |
| PRAF2 | ENST00000376390.4 | PRA1 domain family, member 2 | 1921 | 2 | 1 | 0 | 1 | 2 | bta-miR-2430 | -0.82 |
| CTD-2228K2.5 | ENST00000342584.3 | Uncharacterized protein | 199 | 2 | 1 | 0 | 1 | 0 | bta-miR-2430 | -0.81 |
| LRRC6 | ENST00000519595.1 | leucine rich repeat containing 6 | 5 | 1 | 1 | 0 | 0 | 0 | bta-miR-2430 | -0.78 |
| AF196779.12 | ENST00000376358.3 | Uncharacterized protein; WD repeat domain phosphoinositide-interacting protein 4 | 1921 | 2 | 1 | 0 | 1 | 2 | bta-miR-2430 | -0.77 |
| WDR45 | ENST00000553851.1 | WD repeat domain 45 | 1921 | 2 | 1 | 0 | 1 | 2 | bta-miR-2430 | -0.77 |
| NPLOC4 | ENST00000572760.1 | nuclear protein localization 4 homolog (S. cerevisiae) | 1360 | 3 | 1 | 2 | 0 | 1 | bta-miR-2430 | -0.74 |
| RFXANK | ENST00000353145.1 | regulatory factor X-associated ankyrin-containing protein | 2548 | 2 | 1 | 0 | 1 | 0 | bta-miR-2430 | -0.74 |
| AMTN | ENST00000339336.4 | amelotin | 12 | 1 | 1 | 0 | 0 | 0 | bta-miR-2430 | -0.72 |
| PKIG | ENST00000372894.3 | protein kinase (cAMP-dependent, catalytic) inhibitor gamma | 1619 | 2 | 0 | 2 | 0 | 0 | bta-miR-2430 | -0.72 |
| RPL23A | ENST00000422514.2 | ribosomal protein L23a | 13 | 1 | 1 | 0 | 0 | 0 | bta-miR-2430 | -0.71 |
| POLR3C | ENST00000369294.1 | polymerase (RNA) III (DNA directed) polypeptide C (62kD) | 582 | 2 | 1 | 0 | 1 | 0 | bta-miR-2430 | -0.7 |
| SH2D3A | ENST00000437152.3 | SH2 domain containing 3A | 5 | 1 | 1 | 0 | 0 | 0 | bta-miR-2430 | -0.68 |
| RP11-455G16.1 | ENST00000326780.3 | Uncharacterized protein | 5 | 1 | 1 | 0 | 0 | 0 | bta-miR-2430 | -0.67 |
| STT3B | ENST00000295770.2 | STT3B, subunit of the oligosaccharyltransferase complex (catalytic) | 4458 | 2 | 2 | 0 | 0 | 0 | bta-miR-2430 | -0.67 |
| PRIM2 | ENST00000607273.1 | primase, DNA, polypeptide 2 (58kDa) | 32 | 2 | 1 | 0 | 1 | 0 | bta-miR-2430 | -0.67 |
| CCDC167 | ENST00000373408.3 | coiled-coil domain containing 167 | 1611 | 1 | 1 | 0 | 0 | 0 | bta-miR-2430 | -0.67 |
| GOLT1A | ENST00000308302.3 | golgi transport 1A | 805 | 1 | 1 | 0 | 0 | 0 | bta-miR-2430 | -0.66 |
| PPAPDC1A | ENST00000398248.1 | phosphatidic acid phosphatase type 2 domain containing 1A | 29 | 1 | 1 | 0 | 0 | 0 | bta-miR-2430 | -0.66 |
| ART5 | ENST00000397068.3 | ADP-ribosyltransferase 5 | 50 | 1 | 1 | 0 | 0 | 0 | bta-miR-2430 | -0.64 |
| LYRM5 | ENST00000556402.1 | LYR motif containing 5 | 40 | 1 | 1 | 0 | 0 | 0 | bta-miR-2430 | -0.64 |
| TIRAP | ENST00000392679.1 | toll-interleukin 1 receptor (TIR) domain containing adaptor protein | 82 | 3 | 0 | 2 | 1 | 0 | bta-miR-2430 | -0.64 |
| NKX2-8 | ENST00000258829.5 | NK2 homeobox 8 | 75 | 1 | 1 | 0 | 0 | 1 | bta-miR-2430 | -0.64 |
| L2HGDH | ENST00000267436.4 | L-2-hydroxyglutarate dehydrogenase | 893 | 2 | 1 | 1 | 0 | 0 | bta-miR-2430 | -0.63 |
| FAT3 | ENST00000298047.6 | FAT atypical cadherin 3 | 30 | 2 | 1 | 0 | 1 | 1 | bta-miR-2430 | -0.63 |
| CCNH | ENST00000508855.1 | cyclin H | 250 | 1 | 1 | 0 | 0 | 0 | bta-miR-2430 | -0.63 |
| B3GALT5 | ENST00000380620.4 | UDP-Gal:betaGlcNAc beta 1,3-galactosyltransferase, polypeptide 5 | 5 | 4 | 2 | 1 | 1 | 2 | bta-miR-2430 | -0.63 |
| FGF11 | ENST00000293829.4 | fibroblast growth factor 11 | 10 | 2 | 1 | 1 | 0 | 0 | bta-miR-2430 | -0.62 |
| KLK5 | ENST00000391809.2 | kallikrein-related peptidase 5 | 5 | 1 | 1 | 0 | 0 | 1 | bta-miR-2430 | -0.62 |
| CTXN2 | ENST00000417307.2 | cortexin 2 | 5 | 2 | 1 | 0 | 1 | 0 | bta-miR-2430 | -0.62 |
| C22orf46 | ENST00000402966.1 | chromosome 22 open reading frame 46 | 70 | 6 | 0 | 4 | 2 | 2 | bta-miR-2430 | -0.61 |
| MSH5 | ENST00000534153.4 | mutS homolog 5 | 661 | 2 | 1 | 1 | 0 | 1 | bta-miR-2430 | -0.61 |
| ALPL | ENST00000374840.3 | alkaline phosphatase, liver/bone/kidney | 251 | 3 | 1 | 2 | 0 | 0 | bta-miR-2430 | -0.6 |
| ALYREF | ENST00000331204.4 | Aly/REF export factor | 28673 | 1 | 1 | 0 | 0 | 0 | bta-miR-2430 | -0.6 |
| C1orf115 | ENST00000294889.5 | chromosome 1 open reading frame 115 | 180 | 3 | 1 | 1 | 1 | 1 | bta-miR-2430 | -0.6 |
| FAM19A4 | ENST00000295569.7 | family with sequence similarity 19 (chemokine (C-C motif)-like), member A4 | 5 | 1 | 1 | 0 | 0 | 2 | bta-miR-2430 | -0.6 |
| SAMD14 | ENST00000330175.4 | sterile alpha motif domain containing 14 | 88 | 5 | 1 | 4 | 0 | 0 | bta-miR-2430 | -0.59 |
| AARS | ENST00000261772.8 | alanyl-tRNA synthetase | 20942 | 1 | 1 | 0 | 0 | 1 | bta-miR-2430 | -0.59 |
| FKBP11 | ENST00000552878.1 | FK506 binding protein 11, 19 kDa | 37 | 1 | 1 | 0 | 0 | 0 | bta-miR-2430 | -0.59 |
| CHCHD10 | ENST00000520222.1 | coiled-coil-helix-coiled-coil-helix domain containing 10 | 1103 | 1 | 1 | 0 | 0 | 0 | bta-miR-2430 | -0.58 |
| PNMA6C | ENST00000421798.3 | paraneoplastic Ma antigen family member 6C | 5 | 1 | 1 | 0 | 0 | 0 | bta-miR-2430 | -0.58 |
| SCARA3 | ENST00000301904.3 | scavenger receptor class A, member 3 | 5318 | 2 | 1 | 1 | 0 | 0 | bta-miR-2430 | -0.58 |
| ENPP6 | ENST00000296741.2 | ectonucleotide pyrophosphatase/phosphodiesterase 6 | 5 | 2 | 1 | 1 | 0 | 2 | bta-miR-2430 | -0.57 |
| BOD1L2 | ENST00000585477.1 | biorientation of chromosomes in cell division 1-like 2 | 5 | 1 | 0 | 1 | 0 | 0 | bta-miR-2430 | -0.57 |
| BTF3L4 | ENST00000489308.2 | basic transcription factor 3-like 4 | 1245 | 2 | 1 | 0 | 1 | 0 | bta-miR-2430 | -0.57 |
| RBCK1 | ENST00000356286.5 | RanBP-type and C3HC4-type zinc finger containing 1 | 2123 | 2 | 0 | 2 | 0 | 0 | bta-miR-2430 | -0.56 |
| UQCC1 | ENST00000349714.5 | ubiquinol-cytochrome c reductase complex assembly factor 1 | 500 | 2 | 1 | 1 | 0 | 0 | bta-miR-2430 | -0.56 |
| HNF4A | ENST00000316099.4 | hepatocyte nuclear factor 4, alpha | 915 | 2 | 1 | 0 | 1 | 0 | bta-miR-2430 | -0.56 |
| REPS1 | ENST00000450536.2 | RALBP1 associated Eps domain containing 1 | 725 | 1 | 1 | 0 | 0 | 0 | bta-miR-2430 | -0.56 |
| HEYL | ENST00000372852.3 | hairy/enhancer-of-split related with YRPW motif-like | 37 | 5 | 1 | 3 | 1 | 0 | bta-miR-2430 | -0.55 |
| ZNF709 | ENST00000397732.3 | zinc finger protein 709 | 90 | 3 | 0 | 3 | 0 | 0 | bta-miR-2430 | -0.55 |
| HR | ENST00000381418.4 | hair growth associated | 248 | 2 | 2 | 0 | 0 | 1 | bta-miR-2430 | -0.55 |
| MZT1 | ENST00000377818.3 | mitotic spindle organizing protein 1 | 157 | 1 | 1 | 0 | 0 | 0 | bta-miR-2430 | -0.55 |
| STK11 | ENST00000326873.7 | serine/threonine kinase 11 | 377 | 2 | 1 | 0 | 1 | 0 | bta-miR-2430 | -0.54 |
| PCDHA1 | ENST00000504120.2 | protocadherin alpha 1 | 86 | 1 | 1 | 0 | 0 | 0 | bta-miR-2430 | -0.54 |
| SEMA7A | ENST00000261918.4 | semaphorin 7A, GPI membrane anchor (John Milton Hagen blood group) | 65 | 2 | 1 | 0 | 1 | 0 | bta-miR-2430 | -0.54 |
| NPAS1 | ENST00000439365.2 | neuronal PAS domain protein 1 | 317 | 1 | 1 | 0 | 0 | 0 | bta-miR-2430 | -0.54 |
| DERL2 | ENST00000572834.1 | derlin 2 | 261 | 3 | 0 | 1 | 2 | 0 | bta-miR-2430 | -0.54 |
| KPNA7 | ENST00000327442.6 | karyopherin alpha 7 (importin alpha 8) | 5 | 1 | 1 | 0 | 0 | 0 | bta-miR-2430 | -0.54 |
| TECTB | ENST00000369422.3 | tectorin beta | 5 | 2 | 2 | 0 | 0 | 0 | bta-miR-2430 | -0.54 |
| SYNJ2 | ENST00000449859.2 | synaptojanin 2 | 369 | 1 | 1 | 0 | 0 | 0 | bta-miR-2430 | -0.53 |
| VAMP8 | ENST00000263864.5 | vesicle-associated membrane protein 8 | 711 | 1 | 1 | 0 | 0 | 0 | bta-miR-2430 | -0.53 |
| KLF2 | ENST00000248071.5 | Kruppel-like factor 2 (lung) | 431 | 3 | 1 | 0 | 2 | 0 | bta-miR-2430 | -0.53 |
| MSANTD2 | ENST00000239614.4 | Myb/SANT-like DNA-binding domain containing 2 | 50 | 1 | 1 | 0 | 0 | 0 | bta-miR-2430 | -0.53 |
| C9orf170 | ENST00000375941.2 | chromosome 9 open reading frame 170 | 5 | 2 | 1 | 1 | 0 | 0 | bta-miR-2430 | -0.53 |
| VPS52 | ENST00000482399.1 | vacuolar protein sorting 52 homolog (S. cerevisiae) | 124 | 2 | 1 | 1 | 0 | 0 | bta-miR-2430 | -0.53 |
| EFNB3 | ENST00000226091.2 | ephrin-B3 | 5 | 2 | 1 | 0 | 1 | 0 | bta-miR-2430 | -0.53 |
| ZNF709 | ENST00000428311.1 | | 90 | 3 | 0 | 3 | 0 | 0 | bta-miR-2430 | -0.52 |
| GATA1 | ENST00000376670.3 | GATA binding protein 1 (globin transcription factor 1) | 5 | 1 | 1 | 0 | 0 | 0 | bta-miR-2430 | -0.52 |
| TPPP3 | ENST00000393957.2 | tubulin polymerization-promoting protein family member 3 | 5 | 2 | 1 | 0 | 1 | 0 | bta-miR-2430 | -0.52 |
| KDM4B | ENST00000159111.4 | lysine (K)-specific demethylase 4B | 547 | 1 | 1 | 0 | 0 | 0 | bta-miR-2430 | -0.52 |
| ARC | ENST00000356613.2 | activity-regulated cytoskeleton-associated protein | 5 | 2 | 1 | 1 | 0 | 2 | bta-miR-2430 | -0.52 |
| LGR6 | ENST00000367278.3 | leucine-rich repeat containing G protein-coupled receptor 6 | 18 | 1 | 1 | 0 | 0 | 0 | bta-miR-2430 | -0.52 |
| DDB2 | ENST00000378601.3 | damage-specific DNA binding protein 2, 48kDa | 198 | 2 | 1 | 0 | 1 | 1 | bta-miR-2430 | -0.52 |
| FOSL1 | ENST00000448083.2 | FOS-like antigen 1 | 276 | 1 | 1 | 0 | 0 | 1 | bta-miR-2430 | -0.52 |
| ABCA7 | ENST00000263094.6 | ATP-binding cassette, sub-family A (ABC1), member 7 | 218 | 1 | 1 | 0 | 0 | 0 | bta-miR-2430 | -0.52 |
| KLHDC8B | ENST00000332780.2 | kelch domain containing 8B | 321 | 2 | 1 | 0 | 1 | 0 | bta-miR-2430 | -0.51 |
| CNPY4 | ENST00000262932.3 | canopy FGF signaling regulator 4 | 24 | 1 | 1 | 0 | 0 | 0 | bta-miR-2430 | -0.51 |
| LRRC66 | ENST00000343457.3 | leucine rich repeat containing 66 | 5 | 1 | 1 | 0 | 0 | 0 | bta-miR-2430 | -0.51 |
| SSH2 | ENST00000582084.1 | slingshot protein phosphatase 2 | 190 | 2 | 0 | 2 | 0 | 0 | bta-miR-2430 | -0.51 |
| SH3GL2 | ENST00000380607.4 | SH3-domain GRB2-like 2 | 125 | 1 | 1 | 0 | 0 | 0 | bta-miR-2430 | -0.51 |
| RPL39 | ENST00000361575.3 | ribosomal protein L39 | 207 | 1 | 0 | 1 | 0 | 0 | bta-miR-2430 | -0.5 |
| CSNK2A2 | ENST00000262506.3 | casein kinase 2, alpha prime polypeptide | 99 | 1 | 1 | 0 | 0 | 0 | bta-miR-2430 | -0.5 |
| FKBP14 | ENST00000222803.5 | FK506 binding protein 14, 22 kDa | 939 | 1 | 1 | 0 | 0 | 0 | bta-miR-2430 | -0.5 |
| AC004466.1 | ENST00000599515.1 | Uncharacterized protein | 5 | 1 | 1 | 0 | 0 | 0 | bta-miR-2430 | -0.5 |
| KCND3 | ENST00000369697.1 | potassium voltage-gated channel, Shal-related subfamily, member 3 | 23 | 2 | 0 | 1 | 1 | 1 | bta-miR-2430 | -0.5 |
| RIC3 | ENST00000396677.2 | RIC3 acetylcholine receptor chaperone | 5 | 2 | 0 | 0 | 2 | 1 | bta-miR-2430 | -0.5 |
| EXOSC10 | ENST00000544779.1 | exosome component 10 | 138 | 1 | 1 | 0 | 0 | 0 | bta-miR-2430 | -0.5 |
| TOR2A | ENST00000458505.3 | torsin family 2, member A | 75 | 2 | 1 | 1 | 0 | 1 | bta-miR-2430 | -0.49 |
| SEPP1 | ENST00000514985.1 | selenoprotein P, plasma, 1 | 134 | 1 | 1 | 0 | 0 | 0 | bta-miR-2430 | -0.49 |
| CCBL2 | ENST00000370485.2 | cysteine conjugate-beta lyase 2 | 409 | 1 | 1 | 0 | 0 | 0 | bta-miR-2430 | -0.49 |
| PCDHAC1 | ENST00000253807.2 | protocadherin alpha subfamily C, 1 | 86 | 1 | 1 | 0 | 0 | 0 | bta-miR-2430 | -0.49 |
| TPPP | ENST00000360578.5 | tubulin polymerization promoting protein | 7 | 2 | 1 | 1 | 0 | 0 | bta-miR-2430 | -0.49 |
| SHE | ENST00000304760.2 | Src homology 2 domain containing E | 9 | 2 | 0 | 1 | 1 | 1 | bta-miR-2430 | -0.49 |
| PPP1R14D | ENST00000299174.5 | protein phosphatase 1, regulatory (inhibitor) subunit 14D | 5 | 1 | 1 | 0 | 0 | 0 | bta-miR-2430 | -0.49 |
| PLAC8 | ENST00000311507.4 | placenta-specific 8 | 212 | 1 | 0 | 1 | 0 | 1 | bta-miR-2430 | -0.49 |
| C2orf71 | ENST00000331664.5 | chromosome 2 open reading frame 71 | 5 | 2 | 1 | 1 | 0 | 4 | bta-miR-2430 | -0.49 |
| HOXB4 | ENST00000332503.5 | homeobox B4 | 214 | 1 | 1 | 0 | 0 | 1 | bta-miR-2430 | -0.49 |
| AL359878.1 | ENST00000381466.1 | Uncharacterized protein | 5 | 1 | 0 | 1 | 0 | 0 | bta-miR-2430 | -0.49 |
| PCDHAC2 | ENST00000289269.5 | protocadherin alpha subfamily C, 2 | 86 | 1 | 1 | 0 | 0 | 0 | bta-miR-2430 | -0.49 |
| PDGFRL | ENST00000251630.6 | platelet-derived growth factor receptor-like | 48 | 1 | 1 | 0 | 0 | 0 | bta-miR-2430 | -0.49 |
| ZNF346 | ENST00000503039.1 | zinc finger protein 346 | 5 | 1 | 1 | 0 | 0 | 1 | bta-miR-2430 | -0.48 |
| CREG2 | ENST00000324768.5 | cellular repressor of E1A-stimulated genes 2 | 38 | 4 | 0 | 2 | 2 | 0 | bta-miR-2430 | -0.48 |
| CNPY3 | ENST00000372836.4 | canopy FGF signaling regulator 3 | 837 | 1 | 1 | 0 | 0 | 0 | bta-miR-2430 | -0.48 |
| SPIN2A | ENST00000374908.1 | spindlin family, member 2A | 5 | 1 | 1 | 0 | 0 | 0 | bta-miR-2430 | -0.48 |
| SFN | ENST00000339276.4 | stratifin | 5 | 2 | 0 | 1 | 1 | 0 | bta-miR-2430 | -0.48 |
| PLK5 | ENST00000454744.2 | polo-like kinase 5 | 5 | 3 | 0 | 2 | 1 | 0 | bta-miR-2430 | -0.47 |
| TPBGL | ENST00000562197.2 | trophoblast glycoprotein-like | 8 | 3 | 1 | 0 | 2 | 0 | bta-miR-2430 | -0.47 |
| BLK | ENST00000259089.4 | B lymphoid tyrosine kinase | 5 | 1 | 1 | 0 | 0 | 0 | bta-miR-2430 | -0.47 |
| KCNJ14 | ENST00000391884.1 | potassium inwardly-rectifying channel, subfamily J, member 14 | 29 | 2 | 2 | 0 | 0 | 1 | bta-miR-2430 | -0.47 |
| AK4 | ENST00000545314.1 | adenylate kinase 4 | 116 | 2 | 1 | 1 | 0 | 1 | bta-miR-2430 | -0.47 |
| LINC00632 | ENST00000370535.3 | long intergenic non-protein coding RNA 632 | 5 | 2 | 0 | 1 | 1 | 0 | bta-miR-2430 | -0.47 |
| ADIPOQ | ENST00000412955.2 | adiponectin, C1Q and collagen domain containing | 5 | 2 | 1 | 0 | 1 | 1 | bta-miR-2430 | -0.47 |
| TMCO1 | ENST00000392129.6 | transmembrane and coiled-coil domains 1 | 4447 | 1 | 0 | 1 | 0 | 0 | bta-miR-2430 | -0.47 |
| BEX1 | ENST00000372728.3 | brain expressed, X-linked 1 | 11 | 1 | 0 | 1 | 0 | 0 | bta-miR-2430 | -0.46 |
| SLC41A1 | ENST00000367137.3 | solute carrier family 41 (magnesium transporter), member 1 | 300 | 1 | 1 | 0 | 0 | 4 | bta-miR-2430 | -0.46 |
| BBC3 | ENST00000341983.4 | BCL2 binding component 3 | 87 | 3 | 0 | 3 | 0 | 0 | bta-miR-2430 | -0.46 |
| ACE | ENST00000290866.4 | angiotensin I converting enzyme | 12 | 1 | 1 | 0 | 0 | 1 | bta-miR-2430 | -0.46 |
| ALDH9A1 | ENST00000354775.4 | aldehyde dehydrogenase 9 family, member A1 | 4094 | 1 | 1 | 0 | 0 | 0 | bta-miR-2430 | -0.46 |
| ADRA1D | ENST00000379453.4 | adrenoceptor alpha 1D | 17 | 1 | 1 | 0 | 0 | 0 | bta-miR-2430 | -0.46 |
| POLR2J | ENST00000292614.5 | polymerase (RNA) II (DNA directed) polypeptide J, 13.3kDa | 8349 | 1 | 0 | 1 | 0 | 0 | bta-miR-2430 | -0.46 |
| SRF | ENST00000265354.4 | serum response factor (c-fos serum response element-binding transcription factor) | 596 | 2 | 0 | 2 | 0 | 0 | bta-miR-2430 | -0.46 |
| CALML3 | ENST00000315238.1 | calmodulin-like 3 | 5 | 1 | 1 | 0 | 0 | 1 | bta-miR-2430 | -0.45 |
| CCDC24 | ENST00000372318.3 | coiled-coil domain containing 24 | 66 | 1 | 1 | 0 | 0 | 0 | bta-miR-2430 | -0.45 |
| MFNG | ENST00000416983.3 | MFNG O-fucosylpeptide 3-beta-N-acetylglucosaminyltransferase | 14 | 1 | 1 | 0 | 0 | 0 | bta-miR-2430 | -0.45 |
| OTOG | ENST00000399397.1 | otogelin | 5 | 2 | 0 | 1 | 1 | 0 | bta-miR-2430 | -0.45 |
| CERKL | ENST00000410087.3 | ceramide kinase-like | 8 | 1 | 1 | 0 | 0 | 0 | bta-miR-2430 | -0.45 |
| C17orf100 | ENST00000391428.2 | chromosome 17 open reading frame 100 | 39 | 1 | 0 | 1 | 0 | 0 | bta-miR-2430 | -0.45 |
| DEFB106B | ENST00000335479.2 | defensin, beta 106B | 5 | 1 | 0 | 1 | 0 | 0 | bta-miR-2430 | -0.45 |
| AC005481.5 | ENST00000409610.1 | Uncharacterized protein | 5 | 1 | 0 | 1 | 0 | 0 | bta-miR-2430 | -0.45 |
| TRMT112 | ENST00000535126.1 | tRNA methyltransferase 11-2 homolog (S. cerevisiae) | 283 | 1 | 0 | 1 | 0 | 0 | bta-miR-2430 | -0.45 |
| SARDH | ENST00000371872.4 | sarcosine dehydrogenase | 355 | 1 | 1 | 0 | 0 | 1 | bta-miR-2430 | -0.45 |
| DKFZP434E1119 | ENST00000598970.1 | | 5 | 1 | 1 | 0 | 0 | 2 | bta-miR-2430 | -0.45 |
| TMEM200B | ENST00000521452.1 | transmembrane protein 200B | 152 | 1 | 1 | 0 | 0 | 0 | bta-miR-2430 | -0.45 |
| POLH | ENST00000372226.1 | polymerase (DNA directed), eta | 450 | 2 | 0 | 2 | 0 | 0 | bta-miR-2430 | -0.45 |
| PAN2 | ENST00000425394.2 | PAN2 poly(A) specific ribonuclease subunit homolog (S. cerevisiae) | 310 | 1 | 1 | 0 | 0 | 0 | bta-miR-2430 | -0.45 |
| DEFB106A | ENST00000335186.2 | defensin, beta 106A | 5 | 1 | 0 | 1 | 0 | 0 | bta-miR-2430 | -0.45 |
| FAM163A | ENST00000341785.4 | family with sequence similarity 163, member A | 23 | 2 | 0 | 2 | 0 | 1 | bta-miR-2430 | -0.44 |
| AP5S1 | ENST00000379573.2 | adaptor-related protein complex 5, sigma 1 subunit | 492 | 1 | 0 | 1 | 0 | 3 | bta-miR-2430 | -0.44 |
| VTI1B | ENST00000554659.1 | vesicle transport through interaction with t-SNAREs 1B | 264 | 3 | 1 | 1 | 1 | 0 | bta-miR-2430 | -0.44 |
| EMP1 | ENST00000256951.5 | epithelial membrane protein 1 | 814 | 1 | 1 | 0 | 0 | 1 | bta-miR-2430 | -0.44 |
| GNG7 | ENST00000382159.3 | guanine nucleotide binding protein (G protein), gamma 7 | 92 | 4 | 1 | 3 | 0 | 2 | bta-miR-2430 | -0.44 |
| ASIC4 | ENST00000347842.3 | acid-sensing (proton-gated) ion channel family member 4 | 5 | 2 | 0 | 2 | 0 | 0 | bta-miR-2430 | -0.44 |
| WISP1 | ENST00000250160.6 | WNT1 inducible signaling pathway protein 1 | 5 | 3 | 1 | 1 | 1 | 2 | bta-miR-2430 | -0.44 |
| C11orf57 | ENST00000532163.1 | chromosome 11 open reading frame 57 | 207 | 1 | 1 | 0 | 0 | 0 | bta-miR-2430 | -0.44 |
| NT5C1A | ENST00000235628.1 | 5'-nucleotidase, cytosolic IA | 4 | 4 | 2 | 2 | 0 | 5 | bta-miR-2430 | -0.44 |
| ZKSCAN1 | ENST00000324306.6 | zinc finger with KRAB and SCAN domains 1 | 1314 | 2 | 1 | 1 | 0 | 1 | bta-miR-2430 | -0.43 |
| ACR | ENST00000216139.5 | acrosin | 5 | 1 | 0 | 1 | 0 | 0 | bta-miR-2430 | -0.43 |
| SPN | ENST00000360121.3 | sialophorin | 5 | 2 | 1 | 1 | 0 | 3 | bta-miR-2430 | -0.43 |
| CREB3L3 | ENST00000602147.1 | cAMP responsive element binding protein 3-like 3 | 42 | 2 | 0 | 2 | 0 | 0 | bta-miR-2430 | -0.43 |
| MST4 | ENST00000394334.2 | Serine/threonine-protein kinase MST4 | 47 | 1 | 1 | 0 | 0 | 1 | bta-miR-2430 | -0.43 |
| AC117395.1 | ENST00000593416.1 | LOC646903 protein; Uncharacterized protein | 22 | 1 | 1 | 0 | 0 | 0 | bta-miR-2430 | -0.43 |
| GUK1 | ENST00000366716.1 | guanylate kinase 1 | 3943 | 1 | 1 | 0 | 0 | 0 | bta-miR-2430 | -0.43 |
| CXCR5 | ENST00000292174.4 | chemokine (C-X-C motif) receptor 5 | 5 | 5 | 1 | 2 | 2 | 0 | bta-miR-2430 | -0.43 |
| AL049747.1 | ENST00000594060.1 | | 5 | 1 | 0 | 0 | 1 | 2 | bta-miR-2430 | -0.43 |
| VWA1 | ENST00000338660.5 | von Willebrand factor A domain containing 1 | 145 | 5 | 0 | 3 | 2 | 2 | bta-miR-2430 | -0.43 |
| CXorf24 | ENST00000357412.1 | chromosome X open reading frame 24 | 60 | 1 | 0 | 1 | 0 | 0 | bta-miR-2430 | -0.43 |
| PDRG1 | ENST00000202017.4 | p53 and DNA-damage regulated 1 | 116 | 1 | 1 | 0 | 0 | 0 | bta-miR-2430 | -0.43 |
| ZNF788 | ENST00000430298.2 | zinc finger family member 788 | 16 | 2 | 0 | 2 | 0 | 0 | bta-miR-2430 | -0.43 |
| RASA4B | ENST00000541662.1 | RAS p21 protein activator 4B | 5 | 2 | 1 | 1 | 0 | 0 | bta-miR-2430 | -0.42 |
| FBXO28 | ENST00000424254.2 | F-box protein 28 | 461 | 1 | 1 | 0 | 0 | 1 | bta-miR-2430 | -0.42 |
| MTMR11 | ENST00000406732.3 | myotubularin related protein 11 | 16 | 1 | 1 | 0 | 0 | 0 | bta-miR-2430 | -0.42 |
| HYAL4 | ENST00000223026.4 | hyaluronoglucosaminidase 4 | 5 | 2 | 0 | 1 | 1 | 0 | bta-miR-2430 | -0.42 |
| ZNF135 | ENST00000401053.4 | zinc finger protein 135 | 12 | 2 | 0 | 2 | 0 | 0 | bta-miR-2430 | -0.42 |
| FAIM2 | ENST00000320634.3 | Fas apoptotic inhibitory molecule 2 | 5 | 2 | 1 | 1 | 0 | 0 | bta-miR-2430 | -0.42 |
| CALB2 | ENST00000349553.5 | calbindin 2 | 5 | 1 | 1 | 0 | 0 | 0 | bta-miR-2430 | -0.42 |
| RNF11 | ENST00000242719.3 | ring finger protein 11 | 5881 | 1 | 0 | 1 | 0 | 0 | bta-miR-2430 | -0.42 |
| CRIP3 | ENST00000372569.3 | cysteine-rich protein 3 | 5 | 1 | 0 | 1 | 0 | 0 | bta-miR-2430 | -0.42 |
| POLR2H | ENST00000456318.1 | polymerase (RNA) II (DNA directed) polypeptide H | 3413 | 1 | 0 | 1 | 0 | 0 | bta-miR-2430 | -0.41 |
| ITPRIP | ENST00000337478.1 | inositol 1,4,5-trisphosphate receptor interacting protein | 1138 | 2 | 1 | 1 | 0 | 2 | bta-miR-2430 | -0.41 |
| PUSL1 | ENST00000379031.5 | pseudouridylate synthase-like 1 | 1343 | 1 | 0 | 1 | 0 | 0 | bta-miR-2430 | -0.41 |
| C17orf78 | ENST00000300618.4 | chromosome 17 open reading frame 78 | 5 | 1 | 1 | 0 | 0 | 0 | bta-miR-2430 | -0.41 |
| DUSP13 | ENST00000372702.3 | dual specificity phosphatase 13 | 5 | 2 | 0 | 1 | 1 | 0 | bta-miR-2430 | -0.41 |
| HDDC2 | ENST00000608295.1 | HD domain containing 2 | 1973 | 1 | 0 | 1 | 0 | 1 | bta-miR-2430 | -0.41 |
| C3orf27 | ENST00000356020.2 | chromosome 3 open reading frame 27 | 5 | 1 | 1 | 0 | 0 | 0 | bta-miR-2430 | -0.41 |
| HOXA2 | ENST00000222718.5 | homeobox A2 | 14 | 1 | 0 | 1 | 0 | 1 | bta-miR-2430 | -0.41 |
| P2RY13 | ENST00000325602.5 | purinergic receptor P2Y, G-protein coupled, 13 | 5 | 1 | 1 | 0 | 0 | 0 | bta-miR-2430 | -0.41 |
| SULT1B1 | ENST00000310613.3 | sulfotransferase family, cytosolic, 1B, member 1 | 5 | 1 | 1 | 0 | 0 | 0 | bta-miR-2430 | -0.4 |
| ZNF788 | ENST00000339302.4 | Zinc finger protein 788 | 16 | 2 | 0 | 2 | 0 | 0 | bta-miR-2430 | -0.4 |
| MCAT | ENST00000327555.5 | malonyl CoA:ACP acyltransferase (mitochondrial) | 133 | 2 | 1 | 1 | 0 | 0 | bta-miR-2430 | -0.4 |
| PCDHA2 | ENST00000526136.1 | protocadherin alpha 2 | 86 | 1 | 1 | 0 | 0 | 0 | bta-miR-2430 | -0.4 |
| RHNO1 | ENST00000489288.2 | RAD9-HUS1-RAD1 interacting nuclear orphan 1 | 786 | 1 | 0 | 1 | 0 | 0 | bta-miR-2430 | -0.4 |
| ERBB2 | ENST00000584450.1 | v-erb-b2 avian erythroblastic leukemia viral oncogene homolog 2 | 511 | 2 | 0 | 1 | 1 | 2 | bta-miR-2430 | -0.4 |
| SPDEF | ENST00000374037.3 | SAM pointed domain containing ETS transcription factor | 5 | 2 | 1 | 0 | 1 | 0 | bta-miR-2430 | -0.4 |
| ST3GAL5 | ENST00000393808.3 | ST3 beta-galactoside alpha-2,3-sialyltransferase 5 | 5 | 1 | 1 | 0 | 0 | 0 | bta-miR-2430 | -0.4 |
| FAM127A | ENST00000257013.7 | family with sequence similarity 127, member A | 4717 | 2 | 0 | 1 | 1 | 0 | bta-miR-2430 | -0.4 |
| ZNF395 | ENST00000344423.5 | zinc finger protein 395 | 1044 | 1 | 1 | 0 | 0 | 1 | bta-miR-2430 | -0.4 |
| NAIF1 | ENST00000373078.4 | nuclear apoptosis inducing factor 1 | 19 | 2 | 1 | 1 | 0 | 3 | bta-miR-2430 | -0.4 |
| TRIM39 | ENST00000396551.3 | tripartite motif containing 39 | 840 | 1 | 1 | 0 | 0 | 1 | bta-miR-2430 | -0.4 |
| DDA1 | ENST00000359866.4 | DET1 and DDB1 associated 1 | 76 | 2 | 0 | 2 | 0 | 0 | bta-miR-2430 | -0.39 |
| RP11-192H23.4 | ENST00000534850.1 | Uncharacterized protein | 22 | 1 | 1 | 0 | 0 | 1 | bta-miR-2430 | -0.39 |
| OLFML3 | ENST00000369551.1 | olfactomedin-like 3 | 24 | 1 | 0 | 1 | 0 | 0 | bta-miR-2430 | -0.39 |
| NANP | ENST00000304788.3 | N-acetylneuraminic acid phosphatase | 78 | 2 | 0 | 2 | 0 | 0 | bta-miR-2430 | -0.39 |
| SLC38A7 | ENST00000570101.1 | solute carrier family 38, member 7 | 635 | 2 | 0 | 1 | 1 | 0 | bta-miR-2430 | -0.39 |
| FSTL1 | ENST00000295633.3 | follistatin-like 1 | 6788 | 2 | 1 | 0 | 1 | 1 | bta-miR-2430 | -0.39 |
| KLF8 | ENST00000468660.1 | Kruppel-like factor 8 | 8 | 1 | 1 | 0 | 0 | 0 | bta-miR-2430 | -0.39 |
| RBM8A | ENST00000330165.8 | RNA binding motif protein 8A | 33 | 1 | 1 | 0 | 0 | 1 | bta-miR-2430 | -0.39 |
| CD3E | ENST00000361763.4 | CD3e molecule, epsilon (CD3-TCR complex) | 5 | 2 | 0 | 1 | 1 | 0 | bta-miR-2430 | -0.39 |
| HEPACAM | ENST00000298251.4 | hepatic and glial cell adhesion molecule | 5 | 2 | 1 | 0 | 1 | 0 | bta-miR-2430 | -0.39 |
| GORASP1 | ENST00000319283.3 | golgi reassembly stacking protein 1, 65kDa | 102 | 1 | 0 | 1 | 0 | 0 | bta-miR-2430 | -0.39 |
| RRBP1 | ENST00000377813.1 | ribosome binding protein 1 | 1290 | 2 | 0 | 2 | 0 | 0 | bta-miR-2430 | -0.39 |
| C21orf59 | ENST00000382549.4 | chromosome 21 open reading frame 59 | 288 | 1 | 1 | 0 | 0 | 0 | bta-miR-2430 | -0.39 |
| FAM120B | ENST00000476287.1 | family with sequence similarity 120B | 764 | 1 | 1 | 0 | 0 | 1 | bta-miR-2430 | -0.39 |
| KSR1 | ENST00000398988.3 | kinase suppressor of ras 1 | 256 | 3 | 0 | 2 | 1 | 2 | bta-miR-2430 | -0.39 |
| POU5F1B | ENST00000465342.2 | POU class 5 homeobox 1B | 5 | 1 | 0 | 0 | 1 | 1 | bta-miR-2430 | -0.39 |
| AQP1 | ENST00000311813.4 | aquaporin 1 | 5 | 1 | 1 | 0 | 0 | 0 | bta-miR-2430 | -0.39 |
| CIDEA | ENST00000320477.9 | cell death-inducing DFFA-like effector a | 5 | 1 | 0 | 1 | 0 | 0 | bta-miR-2430 | -0.39 |
| ATP6V0A1 | ENST00000343619.4 | ATPase, H+ transporting, lysosomal V0 subunit a1 | 290 | 2 | 0 | 1 | 1 | 3 | bta-miR-2430 | -0.39 |
| TMEM213 | ENST00000442682.2 | transmembrane protein 213 | 5 | 1 | 1 | 0 | 0 | 0 | bta-miR-2430 | -0.38 |
| VCPKMT | ENST00000395860.2 | valosin containing protein lysine (K) methyltransferase | 10 | 1 | 0 | 1 | 0 | 0 | bta-miR-2430 | -0.38 |
| KCNJ16 | ENST00000589377.1 | potassium inwardly-rectifying channel, subfamily J, member 16 | 5 | 1 | 0 | 0 | 1 | 2 | bta-miR-2430 | -0.38 |
| PABPC1L | ENST00000537323.1 | poly(A) binding protein, cytoplasmic 1-like | 137 | 2 | 0 | 1 | 1 | 1 | bta-miR-2430 | -0.38 |
| ATF2 | ENST00000487334.2 | activating transcription factor 2 | 123 | 2 | 0 | 1 | 1 | 0 | bta-miR-2430 | -0.38 |
| KLHL18 | ENST00000232766.5 | kelch-like family member 18 | 74 | 2 | 1 | 0 | 1 | 2 | bta-miR-2430 | -0.38 |
| MLLT10 | ENST00000377072.3 | myeloid/lymphoid or mixed-lineage leukemia (trithorax homolog, Drosophila); translocated to, 10 | 495 | 1 | 1 | 0 | 0 | 0 | bta-miR-2430 | -0.38 |
| CIAPIN1 | ENST00000565961.1 | cytokine induced apoptosis inhibitor 1 | 423 | 1 | 0 | 1 | 0 | 1 | bta-miR-2430 | -0.38 |
| SLC4A3 | ENST00000273063.6 | solute carrier family 4 (anion exchanger), member 3 | 32 | 1 | 1 | 0 | 0 | 0 | bta-miR-2430 | -0.38 |
| LTA | ENST00000454783.1 | lymphotoxin alpha | 5 | 2 | 0 | 1 | 1 | 0 | bta-miR-2430 | -0.38 |
| CBLL1 | ENST00000440859.3 | Cbl proto-oncogene-like 1, E3 ubiquitin protein ligase | 850 | 1 | 1 | 0 | 0 | 0 | bta-miR-2430 | -0.38 |
| TMPRSS5 | ENST00000536856.1 | transmembrane protease, serine 5 | 8 | 1 | 1 | 0 | 0 | 0 | bta-miR-2430 | -0.38 |
| FXYD6 | ENST00000540359.1 | FXYD domain containing ion transport regulator 6 | 42 | 1 | 1 | 0 | 0 | 0 | bta-miR-2430 | -0.38 |
| ATG9B | ENST00000605938.1 | autophagy related 9B | 5 | 3 | 1 | 1 | 1 | 2 | bta-miR-2430 | -0.38 |
| LTB | ENST00000446745.2 | lymphotoxin beta (TNF superfamily, member 3) | 11 | 1 | 1 | 0 | 0 | 0 | bta-miR-2430 | -0.38 |
| PYROXD2 | ENST00000370575.4 | pyridine nucleotide-disulphide oxidoreductase domain 2 | 15 | 1 | 0 | 1 | 0 | 0 | bta-miR-2430 | -0.38 |
| PCDHA7 | ENST00000525929.1 | protocadherin alpha 7 | 86 | 1 | 1 | 0 | 0 | 0 | bta-miR-2430 | -0.38 |
| SASH3 | ENST00000356892.3 | SAM and SH3 domain containing 3 | 5 | 1 | 1 | 0 | 0 | 0 | bta-miR-2430 | -0.38 |
| PCDHA4 | ENST00000530339.1 | protocadherin alpha 4 | 86 | 1 | 1 | 0 | 0 | 0 | bta-miR-2430 | -0.38 |
| OSBPL11 | ENST00000296220.5 | oxysterol binding protein-like 11 | 101 | 1 | 1 | 0 | 0 | 1 | bta-miR-2430 | -0.38 |
| PCDHA8 | ENST00000531613.1 | protocadherin alpha 8 | 86 | 1 | 1 | 0 | 0 | 0 | bta-miR-2430 | -0.38 |
| PCDHA5 | ENST00000529859.1 | protocadherin alpha 5 | 86 | 1 | 1 | 0 | 0 | 0 | bta-miR-2430 | -0.38 |
| PCDHA12 | ENST00000398631.2 | protocadherin alpha 12 | 86 | 1 | 1 | 0 | 0 | 0 | bta-miR-2430 | -0.38 |
| CYP27B1 | ENST00000228606.4 | cytochrome P450, family 27, subfamily B, polypeptide 1 | 11 | 4 | 1 | 2 | 1 | 0 | bta-miR-2430 | -0.37 |
| PCDHA6 | ENST00000529310.1 | protocadherin alpha 6 | 86 | 1 | 1 | 0 | 0 | 0 | bta-miR-2430 | -0.37 |
| PCDHA11 | ENST00000398640.2 | protocadherin alpha 11 | 86 | 1 | 1 | 0 | 0 | 0 | bta-miR-2430 | -0.37 |
| PCDHA3 | ENST00000522353.2 | protocadherin alpha 3 | 86 | 1 | 1 | 0 | 0 | 0 | bta-miR-2430 | -0.37 |
| PCDHA10 | ENST00000307360.5 | protocadherin alpha 10 | 86 | 1 | 1 | 0 | 0 | 0 | bta-miR-2430 | -0.37 |
| PCDHA13 | ENST00000289272.2 | protocadherin alpha 13 | 86 | 1 | 1 | 0 | 0 | 0 | bta-miR-2430 | -0.37 |
| PCDHA9 | ENST00000532602.1 | protocadherin alpha 9 | 86 | 1 | 1 | 0 | 0 | 0 | bta-miR-2430 | -0.37 |
| EPOR | ENST00000592375.2 | erythropoietin receptor | 96 | 1 | 1 | 0 | 0 | 2 | bta-miR-2430 | -0.37 |
| KRIT1 | ENST00000394507.1 | KRIT1, ankyrin repeat containing | 574 | 1 | 1 | 0 | 0 | 0 | bta-miR-2430 | -0.37 |
| BCMO1 | ENST00000258168.2 | beta-carotene 15,15'-monooxygenase 1 | 5 | 1 | 1 | 0 | 0 | 0 | bta-miR-2430 | -0.37 |
| LIMS2 | ENST00000409286.1 | LIM and senescent cell antigen-like domains 2 | 170 | 1 | 1 | 0 | 0 | 0 | bta-miR-2430 | -0.37 |
| CACNA2D4 | ENST00000382722.5 | calcium channel, voltage-dependent, alpha 2/delta subunit 4 | 16 | 1 | 0 | 1 | 0 | 1 | bta-miR-2430 | -0.37 |
| NDRG1 | ENST00000323851.7 | N-myc downstream regulated 1 | 2195 | 1 | 1 | 0 | 0 | 0 | bta-miR-2430 | -0.37 |
| SLC25A43 | ENST00000217909.7 | solute carrier family 25, member 43 | 5 | 2 | 0 | 0 | 2 | 0 | bta-miR-2430 | -0.37 |
| GNAO1 | ENST00000262493.6 | guanine nucleotide binding protein (G protein), alpha activating activity polypeptide O | 8 | 1 | 1 | 0 | 0 | 0 | bta-miR-2430 | -0.37 |
| C9orf163 | ENST00000354376.1 | chromosome 9 open reading frame 163 | 17 | 2 | 0 | 1 | 1 | 0 | bta-miR-2430 | -0.37 |
| UNC5B | ENST00000335350.6 | unc-5 homolog B (C. elegans) | 90 | 1 | 1 | 0 | 0 | 3 | bta-miR-2430 | -0.37 |
| PMFBP1 | ENST00000537465.1 | polyamine modulated factor 1 binding protein 1 | 5 | 1 | 0 | 1 | 0 | 1 | bta-miR-2430 | -0.37 |
| IDH3G | ENST00000370092.3 | isocitrate dehydrogenase 3 (NAD+) gamma | 391 | 1 | 1 | 0 | 0 | 0 | bta-miR-2430 | -0.37 |
| ACBD3 | ENST00000366812.5 | acyl-CoA binding domain containing 3 | 828 | 1 | 1 | 0 | 0 | 0 | bta-miR-2430 | -0.37 |
| SLC5A6 | ENST00000310574.3 | solute carrier family 5 (sodium/multivitamin and iodide cotransporter), member 6 | 1638 | 2 | 1 | 0 | 1 | 0 | bta-miR-2430 | -0.37 |
| TGM2 | ENST00000361475.2 | transglutaminase 2 | 731 | 3 | 0 | 2 | 1 | 1 | bta-miR-2430 | -0.37 |
| CXCL17 | ENST00000601181.1 | chemokine (C-X-C motif) ligand 17 | 8 | 1 | 0 | 1 | 0 | 0 | bta-miR-2430 | -0.36 |
| ZNF197 | ENST00000383745.2 | zinc finger protein 197 | 53 | 1 | 0 | 1 | 0 | 0 | bta-miR-2430 | -0.36 |
| FHL3 | ENST00000373016.3 | four and a half LIM domains 3 | 921 | 2 | 0 | 2 | 0 | 0 | bta-miR-2430 | -0.36 |
| CERCAM | ENST00000372842.1 | cerebral endothelial cell adhesion molecule | 184 | 4 | 0 | 4 | 0 | 2 | bta-miR-2430 | -0.36 |
| ZNF382 | ENST00000292928.2 | zinc finger protein 382 | 54 | 1 | 0 | 1 | 0 | 0 | bta-miR-2430 | -0.36 |
| B9D2 | ENST00000243578.3 | B9 protein domain 2 | 37 | 1 | 0 | 1 | 0 | 0 | bta-miR-2430 | -0.36 |
| SYCN | ENST00000318438.6 | syncollin | 5 | 1 | 0 | 1 | 0 | 0 | bta-miR-2430 | -0.36 |
| CTDSPL | ENST00000443503.2 | CTD (carboxy-terminal domain, RNA polymerase II, polypeptide A) small phosphatase-like | 372 | 4 | 0 | 1 | 3 | 0 | bta-miR-2430 | -0.36 |
| RSAD2 | ENST00000382040.3 | radical S-adenosyl methionine domain containing 2 | 5 | 1 | 1 | 0 | 0 | 1 | bta-miR-2430 | -0.36 |
| PCDH1 | ENST00000503492.1 | protocadherin 1 | 30 | 2 | 1 | 1 | 0 | 2 | bta-miR-2430 | -0.36 |
| PITX1 | ENST00000265340.7 | paired-like homeodomain 1 | 1502 | 2 | 1 | 1 | 0 | 1 | bta-miR-2430 | -0.36 |
| GID8 | ENST00000266069.3 | GID complex subunit 8 | 515 | 1 | 0 | 1 | 0 | 0 | bta-miR-2430 | -0.35 |
| DHPS | ENST00000210060.7 | deoxyhypusine synthase | 10521 | 1 | 0 | 1 | 0 | 0 | bta-miR-2430 | -0.35 |
| CBFA2T2 | ENST00000375279.2 | core-binding factor, runt domain, alpha subunit 2; translocated to, 2 | 107 | 5* | 1 | 0 | 3 | 1 | bta-miR-2430 | -0.35 |
| ARF3 | ENST00000256682.4 | ADP-ribosylation factor 3 | 1093 | 2 | 0 | 2 | 0 | 0 | bta-miR-2430 | -0.35 |
| MSC | ENST00000325509.4 | musculin | 27 | 1 | 1 | 0 | 0 | 0 | bta-miR-2430 | -0.35 |
| ZBTB44 | ENST00000525842.1 | zinc finger and BTB domain containing 44 | 236 | 2 | 1 | 0 | 1 | 0 | bta-miR-2430 | -0.35 |
| DERL3 | ENST00000404056.1 | derlin 3 | 129 | 3 | 1 | 1 | 1 | 0 | bta-miR-2430 | -0.35 |
| BATF2 | ENST00000301887.4 | basic leucine zipper transcription factor, ATF-like 2 | 5 | 2 | 0 | 1 | 1 | 1 | bta-miR-2430 | -0.35 |
| S100B | ENST00000291700.4 | S100 calcium binding protein B | 5 | 1 | 0 | 1 | 0 | 0 | bta-miR-2430 | -0.35 |
| CNIH3 | ENST00000272133.3 | cornichon family AMPA receptor auxiliary protein 3 | 28 | 1 | 1 | 0 | 0 | 0 | bta-miR-2430 | -0.35 |
| KIAA1279 | ENST00000361983.4 | KIAA1279 | 52 | 1 | 0 | 1 | 0 | 0 | bta-miR-2430 | -0.35 |
| TMEM235 | ENST00000586400.1 | transmembrane protein 235 | 5 | 1 | 1 | 0 | 0 | 0 | bta-miR-2430 | -0.35 |
| IPCEF1 | ENST00000265198.4 | interaction protein for cytohesin exchange factors 1 | 16 | 3 | 1 | 1 | 1 | 1 | bta-miR-2430 | -0.35 |
| NOP2 | ENST00000545200.1 | NOP2 nucleolar protein | 27 | 2 | 0 | 1 | 1 | 0 | bta-miR-2430 | -0.35 |
| THBS4 | ENST00000350881.2 | thrombospondin 4 | 642 | 3 | 0 | 0 | 3 | 0 | bta-miR-2430 | -0.35 |
| CHML | ENST00000366553.1 | choroideremia-like (Rab escort protein 2) | 140 | 1 | 1 | 0 | 0 | 0 | bta-miR-2430 | -0.35 |
| FRRS1L | ENST00000561981.2 | ferric-chelate reductase 1-like | 11 | 3 | 0 | 2 | 1 | 1 | bta-miR-2430 | -0.35 |
| CCR1 | ENST00000296140.3 | chemokine (C-C motif) receptor 1 | 10 | 2 | 0 | 2 | 0 | 0 | bta-miR-2430 | -0.35 |
| ZNF585A | ENST00000356958.4 | zinc finger protein 585A | 77 | 1 | 0 | 1 | 0 | 0 | bta-miR-2430 | -0.35 |
| TAS1R3 | ENST00000339381.5 | taste receptor, type 1, member 3 | 7 | 3 | 0 | 2 | 1 | 1 | bta-miR-2430 | -0.35 |
| TMEM176A | ENST00000004103.3 | transmembrane protein 176A | 1561 | 1 | 0 | 1 | 0 | 0 | bta-miR-2430 | -0.35 |
| PROSC | ENST00000328195.3 | proline synthetase co-transcribed homolog (bacterial) | 414 | 2 | 0 | 1 | 1 | 2 | bta-miR-2430 | -0.35 |
| PRR5L | ENST00000527487.1 | proline rich 5 like | 238 | 3 | 0 | 1 | 2 | 0 | bta-miR-2430 | -0.35 |
| ZBTB4 | ENST00000380599.4 | zinc finger and BTB domain containing 4 | 1039 | 1 | 1 | 0 | 0 | 0 | bta-miR-2430 | -0.35 |
| PLSCR2 | ENST00000336685.2 | phospholipid scramblase 2 | 5 | 1 | 0 | 1 | 0 | 0 | bta-miR-2430 | -0.35 |
| CD226 | ENST00000581982.1 | CD226 molecule | 8 | 1 | 0 | 1 | 0 | 1 | bta-miR-2430 | -0.35 |
| CABP4 | ENST00000438189.2 | calcium binding protein 4 | 5 | 1 | 0 | 1 | 0 | 1 | bta-miR-2430 | -0.34 |
| IL1RN | ENST00000361779.3 | interleukin 1 receptor antagonist | 19 | 1 | 0 | 1 | 0 | 2 | bta-miR-2430 | -0.34 |
| ZNF142 | ENST00000449707.1 | zinc finger protein 142 | 252 | 1 | 1 | 0 | 0 | 0 | bta-miR-2430 | -0.34 |
| TMA16 | ENST00000358572.5 | translation machinery associated 16 homolog (S. cerevisiae) | 34 | 1 | 1 | 0 | 0 | 0 | bta-miR-2430 | -0.34 |
| CYP19A1 | ENST00000396402.1 | cytochrome P450, family 19, subfamily A, polypeptide 1 | 5 | 1 | 1 | 0 | 0 | 1 | bta-miR-2430 | -0.34 |
| VSIG4 | ENST00000455586.2 | V-set and immunoglobulin domain containing 4 | 5 | 2 | 0 | 1 | 1 | 0 | bta-miR-2430 | -0.34 |
| GATA5 | ENST00000252997.2 | GATA binding protein 5 | 7 | 1 | 1 | 0 | 0 | 0 | bta-miR-2430 | -0.34 |
| PTPN3 | ENST00000412145.1 | protein tyrosine phosphatase, non-receptor type 3 | 374 | 1 | 0 | 1 | 0 | 2 | bta-miR-2430 | -0.34 |
| AC006946.15 | ENST00000441544.1 | Uncharacterized protein | 5 | 1 | 0 | 1 | 0 | 0 | bta-miR-2430 | -0.34 |
| HIST2H3C | ENST00000369158.1 | histone cluster 2, H3c | 5 | 1 | 0 | 1 | 0 | 0 | bta-miR-2430 | -0.34 |
| PRDX2 | ENST00000301522.2 | peroxiredoxin 2 | 7500 | 1 | 0 | 1 | 0 | 0 | bta-miR-2430 | -0.34 |
| BTN2A2 | ENST00000432533.2 | butyrophilin, subfamily 2, member A2 | 125 | 1 | 1 | 0 | 0 | 1 | bta-miR-2430 | -0.34 |
| PDAP1 | ENST00000350498.3 | PDGFA associated protein 1 | 208 | 1 | 0 | 1 | 0 | 3 | bta-miR-2430 | -0.34 |
| PAMR1 | ENST00000278360.3 | peptidase domain containing associated with muscle regeneration 1 | 40 | 1 | 1 | 0 | 0 | 1 | bta-miR-2430 | -0.34 |
| CSDC2 | ENST00000306149.7 | cold shock domain containing C2, RNA binding | 31 | 2 | 0 | 2 | 0 | 0 | bta-miR-2430 | -0.34 |
| B3GNT9 | ENST00000449549.3 | UDP-GlcNAc:betaGal beta-1,3-N-acetylglucosaminyltransferase 9 | 56 | 1 | 0 | 1 | 0 | 0 | bta-miR-2430 | -0.34 |
| ZNF557 | ENST00000414706.1 | zinc finger protein 557 | 294 | 1 | 1 | 0 | 0 | 1 | bta-miR-2430 | -0.34 |
| RELA | ENST00000525693.1 | v-rel avian reticuloendotheliosis viral oncogene homolog A | 1707 | 2 | 1 | 1 | 0 | 0 | bta-miR-2430 | -0.34 |
| RASGRF2 | ENST00000265080.4 | Ras protein-specific guanine nucleotide-releasing factor 2 | 140 | 2 | 0 | 2 | 0 | 1 | bta-miR-2430 | -0.34 |
| KLK4 | ENST00000324041.1 | kallikrein-related peptidase 4 | 5 | 1 | 0 | 1 | 0 | 0 | bta-miR-2430 | -0.34 |
| KRAS | ENST00000256078.4 | Kirsten rat sarcoma viral oncogene homolog | 40 | 2 | 1 | 1 | 0 | 0 | bta-miR-2430 | -0.34 |
| METTL14 | ENST00000388822.5 | methyltransferase like 14 | 520 | 1 | 0 | 1 | 0 | 2 | bta-miR-2430 | -0.34 |
| HIP1 | ENST00000336926.6 | huntingtin interacting protein 1 | 37 | 1 | 1 | 0 | 0 | 1 | bta-miR-2430 | -0.34 |
| GPBP1L1 | ENST00000290795.3 | GC-rich promoter binding protein 1-like 1 | 1737 | 1 | 0 | 1 | 0 | 0 | bta-miR-2430 | -0.34 |
| PPP2R5D | ENST00000485511.1 | protein phosphatase 2, regulatory subunit B', delta | 1755 | 3 | 1 | 1 | 1 | 0 | bta-miR-2430 | -0.34 |
| GBA | ENST00000368373.3 | glucosidase, beta, acid | 27 | 3 | 0 | 3 | 0 | 1 | bta-miR-2430 | -0.33 |
| MYADML2 | ENST00000409745.2 | myeloid-associated differentiation marker-like 2 | 5 | 2 | 0 | 1 | 1 | 1 | bta-miR-2430 | -0.33 |
| CRH | ENST00000276571.3 | corticotropin releasing hormone | 5 | 1 | 0 | 1 | 0 | 0 | bta-miR-2430 | -0.33 |
| ZNF558 | ENST00000601372.1 | zinc finger protein 558 | 24 | 1 | 1 | 0 | 0 | 1 | bta-miR-2430 | -0.33 |
| RPTN | ENST00000316073.3 | repetin | 5 | 2 | 0 | 1 | 1 | 0 | bta-miR-2430 | -0.33 |
| RS1 | ENST00000379984.3 | retinoschisin 1 | 5 | 2 | 1 | 0 | 1 | 0 | bta-miR-2430 | -0.33 |
| DACT3 | ENST00000300875.4 | dishevelled-binding antagonist of beta-catenin 3 | 59 | 2 | 0 | 1 | 1 | 0 | bta-miR-2430 | -0.33 |
| HAPLN4 | ENST00000291481.7 | hyaluronan and proteoglycan link protein 4 | 16 | 2 | 0 | 2 | 0 | 0 | bta-miR-2430 | -0.33 |
| DPYSL5 | ENST00000288699.6 | dihydropyrimidinase-like 5 | 315 | 3 | 2 | 1 | 0 | 2 | bta-miR-2430 | -0.33 |
| GPR39 | ENST00000329321.3 | G protein-coupled receptor 39 | 151 | 2 | 0 | 0 | 2 | 1 | bta-miR-2430 | -0.33 |
| TENC1 | ENST00000314250.6 | tensin like C1 domain containing phosphatase (tensin 2) | 240 | 2 | 0 | 2 | 0 | 0 | bta-miR-2430 | -0.33 |
| B4GALNT3 | ENST00000266383.5 | beta-1,4-N-acetyl-galactosaminyl transferase 3 | 5 | 2 | 0 | 1 | 1 | 0 | bta-miR-2430 | -0.33 |
| CCDC86 | ENST00000227520.5 | coiled-coil domain containing 86 | 37 | 1 | 0 | 1 | 0 | 2 | bta-miR-2430 | -0.33 |
| SPACA3 | ENST00000580599.1 | sperm acrosome associated 3 | 5 | 1 | 0 | 1 | 0 | 0 | bta-miR-2430 | -0.33 |
| IFI27 | ENST00000444961.1 | interferon, alpha-inducible protein 27 | 10 | 1 | 0 | 1 | 0 | 0 | bta-miR-2430 | -0.33 |
| TVP23B | ENST00000307767.8 | trans-golgi network vesicle protein 23 homolog B (S. cerevisiae) | 5 | 2 | 0 | 2 | 0 | 0 | bta-miR-2430 | -0.33 |
| POLR3D | ENST00000397802.4 | polymerase (RNA) III (DNA directed) polypeptide D, 44kDa | 678 | 1 | 1 | 0 | 0 | 1 | bta-miR-2430 | -0.33 |
| SLC31A2 | ENST00000259392.3 | solute carrier family 31 (copper transporter), member 2 | 110 | 1 | 0 | 1 | 0 | 0 | bta-miR-2430 | -0.33 |
| C16orf96 | ENST00000444310.4 | chromosome 16 open reading frame 96 | 7 | 1 | 0 | 1 | 0 | 0 | bta-miR-2430 | -0.33 |
| CA5B | ENST00000454127.2 | carbonic anhydrase VB, mitochondrial | 245 | 1 | 1 | 0 | 0 | 0 | bta-miR-2430 | -0.33 |
| HUNK | ENST00000270112.2 | hormonally up-regulated Neu-associated kinase | 85 | 2 | 1 | 0 | 1 | 2 | bta-miR-2430 | -0.33 |
| CD82 | ENST00000227155.4 | CD82 molecule | 1117 | 1 | 0 | 1 | 0 | 1 | bta-miR-2430 | -0.33 |
| FGF16 | ENST00000439435.1 | fibroblast growth factor 16 | 5 | 1 | 0 | 1 | 0 | 0 | bta-miR-2430 | -0.33 |
| HTR5A-AS1 | ENST00000395731.2 | HTR5A antisense RNA 1 | 5 | 1 | 0 | 1 | 0 | 0 | bta-miR-2430 | -0.33 |
| GDE1 | ENST00000353258.3 | glycerophosphodiester phosphodiesterase 1 | 2741 | 2 | 0 | 1 | 1 | 1 | bta-miR-2430 | -0.33 |
| CTD-2267D19.3 | ENST00000578774.1 | Uncharacterized protein | 110 | 2 | 1 | 0 | 1 | 1 | bta-miR-2430 | -0.33 |
| RARG | ENST00000425354.2 | retinoic acid receptor, gamma | 21 | 1 | 1 | 0 | 0 | 0 | bta-miR-2430 | -0.33 |
| APEX2 | ENST00000374987.3 | APEX nuclease (apurinic/apyrimidinic endonuclease) 2 | 972 | 1 | 0 | 1 | 0 | 0 | bta-miR-2430 | -0.33 |
| BCL2L12 | ENST00000246784.3 | BCL2-like 12 (proline rich) | 355 | 1 | 0 | 1 | 0 | 0 | bta-miR-2430 | -0.33 |
| NDRG2 | ENST00000298687.5 | NDRG family member 2 | 217 | 2 | 1 | 1 | 0 | 0 | bta-miR-2430 | -0.33 |
| ZNF563 | ENST00000293725.5 | zinc finger protein 563 | 5 | 1 | 0 | 1 | 0 | 0 | bta-miR-2430 | -0.33 |
| DRG2 | ENST00000395726.4 | developmentally regulated GTP binding protein 2 | 1552 | 2 | 0 | 2 | 0 | 1 | bta-miR-2430 | -0.32 |
| OSBPL2 | ENST00000358053.2 | oxysterol binding protein-like 2 | 283 | 1 | 1 | 0 | 0 | 0 | bta-miR-2430 | -0.32 |
| UBE2D2 | ENST00000398733.3 | ubiquitin-conjugating enzyme E2D 2 | 11458 | 1 | 0 | 1 | 0 | 0 | bta-miR-2430 | -0.32 |
| EGLN2 | ENST00000593726.1 | egl-9 family hypoxia-inducible factor 2 | 19 | 1 | 1 | 0 | 0 | 0 | bta-miR-2430 | -0.32 |
| SLC36A1 | ENST00000243389.3 | solute carrier family 36 (proton/amino acid symporter), member 1 | 236 | 4 | 1 | 2 | 1 | 1 | bta-miR-2430 | -0.32 |
| MAPK10 | ENST00000395169.3 | mitogen-activated protein kinase 10 | 5 | 1 | 1 | 0 | 0 | 2 | bta-miR-2430 | -0.32 |
| FAM227A | ENST00000535113.1 | family with sequence similarity 227, member A | 16 | 1 | 1 | 0 | 0 | 2 | bta-miR-2430 | -0.32 |
| ZNF154 | ENST00000512439.2 | zinc finger protein 154 | 5 | 2 | 0 | 2 | 0 | 1 | bta-miR-2430 | -0.32 |
| C14orf182 | ENST00000399206.1 | chromosome 14 open reading frame 182 | 5 | 1 | 0 | 1 | 0 | 0 | bta-miR-2430 | -0.32 |
| TMIE | ENST00000326431.3 | transmembrane inner ear | 26 | 2 | 0 | 1 | 1 | 0 | bta-miR-2430 | -0.32 |
| STAG3 | ENST00000317296.5 | stromal antigen 3 | 5 | 1 | 0 | 1 | 0 | 0 | bta-miR-2430 | -0.32 |
| AC112693.2 | ENST00000599897.1 | | 5 | 1 | 0 | 1 | 0 | 1 | bta-miR-2430 | -0.32 |
| KIF18A | ENST00000263181.6 | kinesin family member 18A | 152 | 1 | 0 | 1 | 0 | 0 | bta-miR-2430 | -0.32 |
| VWA5B1 | ENST00000289815.8 | von Willebrand factor A domain containing 5B1 | 5 | 1 | 1 | 0 | 0 | 0 | bta-miR-2430 | -0.32 |
| TCP11 | ENST00000412155.2 | t-complex 11, testis-specific | 5 | 1 | 0 | 1 | 0 | 0 | bta-miR-2430 | -0.32 |
| AC007375.1 | ENST00000600936.1 | Uncharacterized protein; cDNA FLJ43210 fis, clone FEBRA2020582 | 7 | 1 | 1 | 0 | 0 | 0 | bta-miR-2430 | -0.32 |
| DNAJB5 | ENST00000545841.1 | DnaJ (Hsp40) homolog, subfamily B, member 5 | 379 | 1 | 0 | 0 | 1 | 1 | bta-miR-2430 | -0.32 |
| ABHD14A | ENST00000273596.3 | abhydrolase domain containing 14A | 523 | 1 | 0 | 1 | 0 | 0 | bta-miR-2430 | -0.32 |
| KLF1 | ENST00000264834.4 | Kruppel-like factor 1 (erythroid) | 5 | 1 | 0 | 1 | 0 | 0 | bta-miR-2430 | -0.32 |
| GPN2 | ENST00000374135.4 | GPN-loop GTPase 2 | 97 | 2 | 0 | 2 | 0 | 0 | bta-miR-2430 | -0.32 |
| NT5DC1 | ENST00000319550.4 | 5'-nucleotidase domain containing 1 | 127 | 2 | 1 | 1 | 0 | 0 | bta-miR-2430 | -0.32 |
| KCNJ15 | ENST00000328656.4 | potassium inwardly-rectifying channel, subfamily J, member 15 | 5 | 1 | 1 | 0 | 0 | 0 | bta-miR-2430 | -0.32 |
| GNAS | ENST00000371075.3 | GNAS complex locus | 314 | 1 | 0 | 1 | 0 | 0 | bta-miR-2430 | -0.32 |
| GFOD1 | ENST00000603223.1 | glucose-fructose oxidoreductase domain containing 1 | 39 | 1 | 0 | 1 | 0 | 1 | bta-miR-2430 | -0.32 |
| ABCA12 | ENST00000272895.7 | ATP-binding cassette, sub-family A (ABC1), member 12 | 5 | 1 | 1 | 0 | 0 | 1 | bta-miR-2430 | -0.32 |
| RGSL1 | ENST00000294854.8 | regulator of G-protein signaling like 1 | 5 | 1 | 0 | 1 | 0 | 0 | bta-miR-2430 | -0.32 |
| MAP3K3 | ENST00000361357.3 | mitogen-activated protein kinase kinase kinase 3 | 144 | 1 | 0 | 1 | 0 | 3 | bta-miR-2430 | -0.32 |
| CDR2L | ENST00000337231.5 | cerebellar degeneration-related protein 2-like | 28 | 3 | 0 | 3 | 0 | 0 | bta-miR-2430 | -0.32 |
| SCAMP3 | ENST00000302631.3 | secretory carrier membrane protein 3 | 446 | 1 | 0 | 1 | 0 | 0 | bta-miR-2430 | -0.31 |
| SLC25A23 | ENST00000301454.4 | solute carrier family 25 (mitochondrial carrier; phosphate carrier), member 23 | 5 | 1 | 0 | 1 | 0 | 0 | bta-miR-2430 | -0.31 |
| CCNE2 | ENST00000520509.1 | cyclin E2 | 76 | 1 | 0 | 1 | 0 | 0 | bta-miR-2430 | -0.31 |
| MESP2 | ENST00000560219.1 | mesoderm posterior 2 homolog (mouse) | 8 | 1 | 0 | 1 | 0 | 0 | bta-miR-2430 | -0.31 |
| NR1D2 | ENST00000312521.4 | nuclear receptor subfamily 1, group D, member 2 | 154 | 2 | 1 | 1 | 0 | 0 | bta-miR-2430 | -0.31 |
| BMP8B | ENST00000372827.3 | bone morphogenetic protein 8b | 88 | 1 | 0 | 1 | 0 | 2 | bta-miR-2430 | -0.31 |
| WDR82 | ENST00000296490.3 | WD repeat domain 82 | 319 | 2 | 1 | 0 | 1 | 0 | bta-miR-2430 | -0.31 |
| PPP1R8 | ENST00000311772.5 | protein phosphatase 1, regulatory subunit 8 | 1066 | 1 | 1 | 0 | 0 | 0 | bta-miR-2430 | -0.31 |
| GCK | ENST00000403799.3 | glucokinase (hexokinase 4) | 5 | 1 | 1 | 0 | 0 | 0 | bta-miR-2430 | -0.31 |
| CSPP1 | ENST00000262210.5 | centrosome and spindle pole associated protein 1 | 101 | 1 | 0 | 1 | 0 | 0 | bta-miR-2430 | -0.31 |
| SLC38A11 | ENST00000303735.4 | solute carrier family 38, member 11 | 5 | 1 | 0 | 1 | 0 | 0 | bta-miR-2430 | -0.31 |
| MYF6 | ENST00000228641.3 | myogenic factor 6 (herculin) | 5 | 1 | 0 | 1 | 0 | 0 | bta-miR-2430 | -0.31 |
| GPR3 | ENST00000374024.3 | G protein-coupled receptor 3 | 5 | 1 | 0 | 1 | 0 | 0 | bta-miR-2430 | -0.31 |
| CXCL9 | ENST00000264888.5 | chemokine (C-X-C motif) ligand 9 | 5 | 1 | 0 | 1 | 0 | 0 | bta-miR-2430 | -0.31 |
| EEF2 | ENST00000309311.6 | eukaryotic translation elongation factor 2 | 25580 | 1 | 0 | 1 | 0 | 0 | bta-miR-2430 | -0.31 |
| ORM2 | ENST00000412657.1 | orosomucoid 2 | 5 | 1 | 0 | 1 | 0 | 0 | bta-miR-2430 | -0.31 |
| STIM1 | ENST00000527651.1 | stromal interaction molecule 1 | 1672 | 1 | 1 | 0 | 0 | 0 | bta-miR-2430 | -0.31 |
| SLC52A3 | ENST00000381944.3 | solute carrier family 52 (riboflavin transporter), member 3 | 5 | 2 | 0 | 1 | 1 | 2 | bta-miR-2430 | -0.31 |
| SHANK2 | ENST00000449833.2 | SH3 and multiple ankyrin repeat domains 2 | 26 | 2 | 1 | 1 | 0 | 4 | bta-miR-2430 | -0.31 |
| C10orf71 | ENST00000323868.4 | chromosome 10 open reading frame 71 | 5 | 3 | 0 | 3 | 0 | 1 | bta-miR-2430 | -0.31 |
| DGCR2 | ENST00000545799.1 | DiGeorge syndrome critical region gene 2 | 1754 | 1 | 1 | 0 | 0 | 0 | bta-miR-2430 | -0.31 |
| FNDC5 | ENST00000496770.1 | fibronectin type III domain containing 5 | 5 | 1 | 0 | 1 | 0 | 1 | bta-miR-2430 | -0.31 |
| CRX | ENST00000221996.7 | cone-rod homeobox | 5 | 1 | 1 | 0 | 0 | 0 | bta-miR-2430 | -0.31 |
| PHB | ENST00000300408.3 | prohibitin | 759 | 2 | 0 | 1 | 1 | 0 | bta-miR-2430 | -0.31 |
| SCRT1 | ENST00000332135.4 | scratch homolog 1, zinc finger protein (Drosophila) | 5 | 1 | 1 | 0 | 0 | 0 | bta-miR-2430 | -0.31 |
| SELP | ENST00000263686.6 | selectin P (granule membrane protein 140kDa, antigen CD62) | 5 | 1 | 1 | 0 | 0 | 0 | bta-miR-2430 | -0.31 |
| TEAD1 | ENST00000361905.4 | TEA domain family member 1 (SV40 transcriptional enhancer factor) | 740 | 2 | 0 | 2 | 0 | 1 | bta-miR-2430 | -0.31 |
| EMILIN1 | ENST00000380320.4 | elastin microfibril interfacer 1 | 5 | 1 | 1 | 0 | 0 | 0 | bta-miR-2430 | -0.31 |
| RAB31 | ENST00000578921.1 | RAB31, member RAS oncogene family | 726 | 2 | 0 | 2 | 0 | 1 | bta-miR-2430 | -0.31 |
| RBBP4 | ENST00000373493.5 | retinoblastoma binding protein 4 | 486 | 1 | 1 | 0 | 0 | 1 | bta-miR-2430 | -0.31 |
| C1orf87 | ENST00000450089.2 | chromosome 1 open reading frame 87 | 5 | 1 | 0 | 0 | 1 | 0 | bta-miR-2430 | -0.31 |
| G6PD | ENST00000393562.2 | glucose-6-phosphate dehydrogenase | 9 | 1 | 0 | 1 | 0 | 0 | bta-miR-2430 | -0.31 |
| HABP2 | ENST00000542051.1 | hyaluronan binding protein 2 | 34 | 3 | 0 | 0 | 3 | 1 | bta-miR-2430 | -0.31 |
| GBGT1 | ENST00000372043.3 | globoside alpha-1,3-N-acetylgalactosaminyltransferase 1 | 41 | 1 | 1 | 0 | 0 | 1 | bta-miR-2430 | -0.3 |
| MRPL17 | ENST00000288937.6 | mitochondrial ribosomal protein L17 | 1447 | 2 | 1 | 0 | 1 | 1 | bta-miR-2430 | -0.3 |
| ACHE | ENST00000302913.4 | acetylcholinesterase | 14 | 2 | 0 | 2 | 0 | 0 | bta-miR-2430 | -0.3 |
| GREM1 | ENST00000560677.1 | gremlin 1, DAN family BMP antagonist | 752 | 1 | 0 | 1 | 0 | 2 | bta-miR-2430 | -0.3 |
| TNFAIP8L1 | ENST00000327473.4 | tumor necrosis factor, alpha-induced protein 8-like 1 | 603 | 1 | 0 | 1 | 0 | 1 | bta-miR-2430 | -0.3 |
| CNOT10 | ENST00000328834.5 | CCR4-NOT transcription complex, subunit 10 | 3264 | 1 | 0 | 1 | 0 | 0 | bta-miR-2430 | -0.3 |
| FAM167A | ENST00000284486.4 | family with sequence similarity 167, member A | 85 | 3 | 0 | 0 | 3 | 0 | bta-miR-2430 | -0.3 |
| MFSD2A | ENST00000372811.5 | major facilitator superfamily domain containing 2A | 113 | 1 | 0 | 1 | 0 | 1 | bta-miR-2430 | -0.3 |
| ZNF791 | ENST00000446165.1 | zinc finger protein 791 | 242 | 1 | 0 | 1 | 0 | 0 | bta-miR-2430 | -0.3 |
| CLEC4F | ENST00000272367.2 | C-type lectin domain family 4, member F | 5 | 1 | 0 | 1 | 0 | 0 | bta-miR-2430 | -0.3 |
| MAPKAPK2 | ENST00000294981.4 | mitogen-activated protein kinase-activated protein kinase 2 | 178 | 1 | 1 | 0 | 0 | 0 | bta-miR-2430 | -0.3 |
| ARPC1A | ENST00000262942.5 | actin related protein 2/3 complex, subunit 1A, 41kDa | 99 | 1 | 0 | 1 | 0 | 0 | bta-miR-2430 | -0.3 |
| CHST8 | ENST00000438847.3 | carbohydrate (N-acetylgalactosamine 4-0) sulfotransferase 8 | 32 | 1 | 0 | 1 | 0 | 1 | bta-miR-2430 | -0.3 |
| PRICKLE1 | ENST00000455697.1 | prickle homolog 1 (Drosophila) | 146 | 2 | 0 | 2 | 0 | 0 | bta-miR-2430 | -0.3 |
| WNT9B | ENST00000290015.2 | wingless-type MMTV integration site family, member 9B | 28 | 3 | 1 | 1 | 1 | 2 | bta-miR-2430 | -0.3 |
| AMFR | ENST00000290649.5 | autocrine motility factor receptor, E3 ubiquitin protein ligase | 1350 | 1 | 1 | 0 | 0 | 0 | bta-miR-2430 | -0.3 |
| ISL1 | ENST00000230658.7 | ISL LIM homeobox 1 | 67 | 1 | 0 | 1 | 0 | 0 | bta-miR-2430 | -0.3 |
| MARCH1 | ENST00000274056.7 | membrane-associated ring finger (C3HC4) 1, E3 ubiquitin protein ligase | 5 | 3 | 0 | 0 | 3 | 0 | bta-miR-2430 | -0.3 |
| C3orf52 | ENST00000431717.2 | chromosome 3 open reading frame 52 | 402 | 1 | 0 | 1 | 0 | 0 | bta-miR-2430 | -0.3 |
| EHD3 | ENST00000322054.5 | EH-domain containing 3 | 206 | 3 | 1 | 1 | 1 | 2 | bta-miR-2430 | -0.3 |
| CDC42EP1 | ENST00000249014.4 | CDC42 effector protein (Rho GTPase binding) 1 | 4709 | 1 | 0 | 1 | 0 | 1 | bta-miR-2430 | -0.3 |
| ZNF57 | ENST00000306908.5 | zinc finger protein 57 | 33 | 2 | 1 | 1 | 0 | 0 | bta-miR-2430 | -0.3 |
| CRMP1 | ENST00000324989.7 | collapsin response mediator protein 1 | 252 | 1 | 0 | 1 | 0 | 0 | bta-miR-2430 | -0.3 |
| GZF1 | ENST00000338121.5 | GDNF-inducible zinc finger protein 1 | 252 | 1 | 1 | 0 | 0 | 1 | bta-miR-2430 | -0.3 |
| NFKBIZ | ENST00000394054.2 | nuclear factor of kappa light polypeptide gene enhancer in B-cells inhibitor, zeta | 236 | 1 | 0 | 1 | 0 | 0 | bta-miR-2430 | -0.3 |
| STX4 | ENST00000313843.3 | syntaxin 4 | 53 | 3 | 0 | 3 | 0 | 0 | bta-miR-2430 | -0.3 |
| NRXN1 | ENST00000342183.5 | neurexin 1 | 5 | 1 | 1 | 0 | 0 | 0 | bta-miR-2430 | -0.3 |
| TLR5 | ENST00000540964.1 | toll-like receptor 5 | 5 | 1 | 1 | 0 | 0 | 1 | bta-miR-2430 | -0.3 |
| NUP43 | ENST00000367403.3 | nucleoporin 43kDa | 113 | 2 | 0 | 1 | 1 | 0 | bta-miR-2430 | -0.3 |
| LANCL1 | ENST00000443314.1 | LanC lantibiotic synthetase component C-like 1 (bacterial) | 3395 | 1 | 0 | 1 | 0 | 1 | bta-miR-2430 | -0.3 |
| AIDA | ENST00000340020.6 | axin interactor, dorsalization associated | 48 | 2 | 0 | 2 | 0 | 0 | bta-miR-2430 | -0.29 |
| GRAPL | ENST00000344415.4 | GRB2-related adaptor protein-like | 5 | 1 | 0 | 1 | 0 | 0 | bta-miR-2430 | -0.29 |
| ANXA6 | ENST00000354546.5 | annexin A6 | 10462 | 1 | 0 | 1 | 0 | 1 | bta-miR-2430 | -0.29 |
| C10orf99 | ENST00000372126.3 | chromosome 10 open reading frame 99 | 5 | 1 | 0 | 0 | 1 | 1 | bta-miR-2430 | -0.29 |
| ARFRP1 | ENST00000440854.1 | ADP-ribosylation factor related protein 1 | 652 | 2 | 0 | 1 | 1 | 0 | bta-miR-2430 | -0.29 |
| AAR2 | ENST00000320849.4 | AAR2 splicing factor homolog (S. cerevisiae) | 74 | 1 | 1 | 0 | 0 | 1 | bta-miR-2430 | -0.29 |
| C1QTNF7 | ENST00000429690.1 | C1q and tumor necrosis factor related protein 7 | 5 | 1 | 1 | 0 | 0 | 0 | bta-miR-2430 | -0.29 |
| DSCAML1 | ENST00000527706.1 | Down syndrome cell adhesion molecule like 1 | 5 | 1 | 0 | 1 | 0 | 0 | bta-miR-2430 | -0.29 |
| C16orf89 | ENST00000474471.3 | chromosome 16 open reading frame 89 | 5 | 1 | 0 | 1 | 0 | 1 | bta-miR-2430 | -0.29 |
| SP6 | ENST00000342234.2 | Sp6 transcription factor | 5 | 2 | 0 | 1 | 1 | 1 | bta-miR-2430 | -0.29 |
| AMY1A | ENST00000370083.4 | amylase, alpha 1A (salivary) | 5 | 1 | 0 | 0 | 1 | 0 | bta-miR-2430 | -0.29 |
| CHD6 | ENST00000373233.3 | chromodomain helicase DNA binding protein 6 | 85 | 2 | 1 | 1 | 0 | 2 | bta-miR-2430 | -0.29 |
| FERMT3 | ENST00000345728.5 | fermitin family member 3 | 10 | 1 | 0 | 1 | 0 | 0 | bta-miR-2430 | -0.29 |
| FAM211A | ENST00000409083.3 | family with sequence similarity 211, member A | 23 | 2 | 0 | 1 | 1 | 2 | bta-miR-2430 | -0.29 |
| FERD3L | ENST00000275461.3 | Fer3-like (Drosophila) | 5 | 1 | 0 | 1 | 0 | 0 | bta-miR-2430 | -0.29 |
| CCNJ | ENST00000265992.5 | cyclin J | 5 | 1 | 1 | 0 | 0 | 1 | bta-miR-2430 | -0.29 |
| ZNF572 | ENST00000319286.5 | zinc finger protein 572 | 16 | 1 | 1 | 0 | 0 | 0 | bta-miR-2430 | -0.29 |
| ZNF445 | ENST00000425708.2 | zinc finger protein 445 | 370 | 3 | 0 | 2 | 1 | 1 | bta-miR-2430 | -0.29 |
| AMY1C | ENST00000370079.3 | amylase, alpha 1C (salivary) | 5 | 1 | 0 | 0 | 1 | 0 | bta-miR-2430 | -0.29 |
| RASA4 | ENST00000262940.7 | RAS p21 protein activator 4 | 5 | 2 | 1 | 1 | 0 | 0 | bta-miR-2430 | -0.29 |
| C15orf57 | ENST00000358005.3 | chromosome 15 open reading frame 57 | 79 | 1 | 0 | 1 | 0 | 0 | bta-miR-2430 | -0.29 |
| CD300E | ENST00000392619.1 | CD300e molecule | 5 | 1 | 1 | 0 | 0 | 0 | bta-miR-2430 | -0.29 |
| NUDT17 | ENST00000334513.5 | nudix (nucleoside diphosphate linked moiety X)-type motif 17 | 131 | 1 | 0 | 1 | 0 | 0 | bta-miR-2430 | -0.29 |
| TRIM55 | ENST00000315962.4 | tripartite motif containing 55 | 5 | 1 | 1 | 0 | 0 | 0 | bta-miR-2430 | -0.29 |
| CDC42BPG | ENST00000342711.5 | CDC42 binding protein kinase gamma (DMPK-like) | 68 | 1 | 1 | 0 | 0 | 0 | bta-miR-2430 | -0.29 |
| CXorf56 | ENST00000320339.4 | chromosome X open reading frame 56 | 279 | 1 | 0 | 1 | 0 | 0 | bta-miR-2430 | -0.29 |
| EDN1 | ENST00000379375.5 | endothelin 1 | 113 | 1 | 0 | 1 | 0 | 0 | bta-miR-2430 | -0.29 |
| C17orf103 | ENST00000399011.2 | chromosome 17 open reading frame 103 | 54 | 1 | 0 | 1 | 0 | 1 | bta-miR-2430 | -0.29 |
| C3orf65 | ENST00000296270.1 | chromosome 3 open reading frame 65 | 5 | 2 | 0 | 2 | 0 | 0 | bta-miR-2430 | -0.29 |
| ARMCX6 | ENST00000361910.4 | armadillo repeat containing, X-linked 6 | 5 | 1 | 0 | 1 | 0 | 0 | bta-miR-2430 | -0.29 |
| GOLPH3L | ENST00000271732.3 | golgi phosphoprotein 3-like | 274 | 1 | 1 | 0 | 0 | 1 | bta-miR-2430 | -0.29 |
| ACSL6 | ENST00000379264.2 | acyl-CoA synthetase long-chain family member 6 | 7 | 3 | 0 | 0 | 3 | 2 | bta-miR-2430 | -0.29 |
| RHPN1 | ENST00000289013.6 | rhophilin, Rho GTPase binding protein 1 | 97 | 1 | 0 | 1 | 0 | 1 | bta-miR-2430 | -0.29 |
| TUSC5 | ENST00000333813.3 | tumor suppressor candidate 5 | 5 | 2 | 0 | 1 | 1 | 0 | bta-miR-2430 | -0.29 |
| BLID | ENST00000560104.1 | BH3-like motif containing, cell death inducer | 5 | 1 | 0 | 0 | 1 | 0 | bta-miR-2430 | -0.29 |
| DNAH10OS | ENST00000514254.2 | dynein, axonemal, heavy chain 10 opposite strand | 89 | 3 | 0 | 1 | 2 | 1 | bta-miR-2430 | -0.29 |
| PANX1 | ENST00000227638.3 | pannexin 1 | 576 | 1 | 1 | 0 | 0 | 3 | bta-miR-2430 | -0.29 |
| TMEM41B | ENST00000528080.1 | transmembrane protein 41B | 191 | 1 | 0 | 1 | 0 | 0 | bta-miR-2430 | -0.28 |
| FAM214B | ENST00000378566.1 | family with sequence similarity 214, member B | 8 | 1 | 0 | 1 | 0 | 1 | bta-miR-2430 | -0.28 |
| SYS1 | ENST00000243918.5 | SYS1 Golgi-localized integral membrane protein homolog (S. cerevisiae) | 384 | 1 | 1 | 0 | 0 | 0 | bta-miR-2430 | -0.28 |
| LRRC3C | ENST00000377924.4 | leucine rich repeat containing 3C | 9 | 3 | 1 | 2 | 0 | 0 | bta-miR-2430 | -0.28 |
| TMCC3 | ENST00000261226.4 | transmembrane and coiled-coil domain family 3 | 70 | 1 | 1 | 0 | 0 | 1 | bta-miR-2430 | -0.28 |
| USH1G | ENST00000319642.1 | Usher syndrome 1G (autosomal recessive) | 5 | 1 | 1 | 0 | 0 | 0 | bta-miR-2430 | -0.28 |
| AMY2A | ENST00000414303.2 | amylase, alpha 2A (pancreatic) | 5 | 1 | 0 | 0 | 1 | 0 | bta-miR-2430 | -0.28 |
| N4BP3 | ENST00000274605.5 | NEDD4 binding protein 3 | 35 | 1 | 0 | 1 | 0 | 1 | bta-miR-2430 | -0.28 |
| NAV3 | ENST00000228327.6 | neuron navigator 3 | 277 | 2 | 1 | 0 | 1 | 0 | bta-miR-2430 | -0.28 |
| HMGXB3 | ENST00000503427.1 | HMG box domain containing 3 | 26 | 1 | 1 | 0 | 0 | 0 | bta-miR-2430 | -0.28 |
| CHRM1 | ENST00000306960.3 | cholinergic receptor, muscarinic 1 | 5 | 2 | 0 | 2 | 0 | 1 | bta-miR-2430 | -0.28 |
| PPME1 | ENST00000328257.8 | protein phosphatase methylesterase 1 | 343 | 1 | 1 | 0 | 0 | 1 | bta-miR-2430 | -0.28 |
| WBP2NL | ENST00000328823.9 | WBP2 N-terminal like | 5 | 1 | 0 | 1 | 0 | 0 | bta-miR-2430 | -0.28 |
| SUDS3 | ENST00000397564.2 | suppressor of defective silencing 3 homolog (S. cerevisiae) | 224 | 1 | 1 | 0 | 0 | 1 | bta-miR-2430 | -0.28 |
| FOXC2 | ENST00000320354.4 | forkhead box C2 (MFH-1, mesenchyme forkhead 1) | 665 | 1 | 0 | 1 | 0 | 0 | bta-miR-2430 | -0.28 |
| KCNN4 | ENST00000262888.3 | potassium intermediate/small conductance calcium-activated channel, subfamily N, member 4 | 142 | 1 | 0 | 1 | 0 | 0 | bta-miR-2430 | -0.28 |
| FAM184B | ENST00000265018.3 | family with sequence similarity 184, member B | 22 | 1 | 1 | 0 | 0 | 0 | bta-miR-2430 | -0.28 |
| PRCC | ENST00000271526.4 | papillary renal cell carcinoma (translocation-associated) | 8562 | 1 | 0 | 1 | 0 | 0 | bta-miR-2430 | -0.28 |
| GABRD | ENST00000378585.4 | gamma-aminobutyric acid (GABA) A receptor, delta | 5 | 1 | 0 | 1 | 0 | 0 | bta-miR-2430 | -0.28 |
| PADI2 | ENST00000375486.4 | peptidyl arginine deiminase, type II | 11 | 2 | 1 | 1 | 0 | 0 | bta-miR-2430 | -0.28 |
| UBFD1 | ENST00000395878.3 | ubiquitin family domain containing 1 | 6826 | 1 | 1 | 0 | 0 | 0 | bta-miR-2430 | -0.28 |
| TULP4 | ENST00000367097.3 | tubby like protein 4 | 93 | 2 | 0 | 2 | 0 | 0 | bta-miR-2430 | -0.28 |
| SPEF1 | ENST00000379756.3 | sperm flagellar 1 | 5 | 2 | 0 | 1 | 1 | 1 | bta-miR-2430 | -0.28 |
| GREB1L | ENST00000580732.2 | growth regulation by estrogen in breast cancer-like | 30 | 1 | 1 | 0 | 0 | 0 | bta-miR-2430 | -0.28 |
| RNASEH2A | ENST00000221486.4 | ribonuclease H2, subunit A | 1519 | 1 | 0 | 1 | 0 | 0 | bta-miR-2430 | -0.28 |
| NPFFR1 | ENST00000277942.6 | neuropeptide FF receptor 1 | 5 | 3 | 0 | 3 | 0 | 2 | bta-miR-2430 | -0.28 |
| FAM83H | ENST00000388913.3 | family with sequence similarity 83, member H | 39 | 2 | 1 | 1 | 0 | 0 | bta-miR-2430 | -0.28 |
| ANKRD54 | ENST00000215941.4 | ankyrin repeat domain 54 | 5 | 1 | 0 | 1 | 0 | 0 | bta-miR-2430 | -0.28 |
| ZNF662 | ENST00000541208.1 | zinc finger protein 662 | 11 | 1 | 0 | 1 | 0 | 0 | bta-miR-2430 | -0.28 |
| MTPN | ENST00000393085.3 | myotrophin | 1296 | 1 | 0 | 1 | 0 | 1 | bta-miR-2430 | -0.28 |
| KIAA0141 | ENST00000194118.4 | KIAA0141 | 1078 | 2 | 0 | 2 | 0 | 1 | bta-miR-2430 | -0.28 |
| GMDS | ENST00000530927.1 | GDP-mannose 4,6-dehydratase | 46 | 3 | 1 | 1 | 1 | 1 | bta-miR-2430 | -0.28 |
| EPS8L2 | ENST00000318562.8 | EPS8-like 2 | 1419 | 1 | 0 | 1 | 0 | 1 | bta-miR-2430 | -0.28 |
| HRK | ENST00000257572.5 | harakiri, BCL2 interacting protein (contains only BH3 domain) | 19 | 2 | 0 | 1 | 1 | 3 | bta-miR-2430 | -0.28 |
| FAM222B | ENST00000582266.1 | family with sequence similarity 222, member B | 100 | 2 | 0 | 1 | 1 | 2 | bta-miR-2430 | -0.28 |
| FCRL4 | ENST00000271532.1 | Fc receptor-like 4 | 5 | 1 | 0 | 1 | 0 | 1 | bta-miR-2430 | -0.28 |
| DENND5A | ENST00000328194.3 | DENN/MADD domain containing 5A | 294 | 1 | 1 | 0 | 0 | 0 | bta-miR-2430 | -0.28 |
| CABP7 | ENST00000216144.3 | calcium binding protein 7 | 12 | 2 | 0 | 2 | 0 | 1 | bta-miR-2430 | -0.28 |
| CD200 | ENST00000315711.8 | CD200 molecule | 5 | 1 | 0 | 1 | 0 | 1 | bta-miR-2430 | -0.28 |
| ST8SIA2 | ENST00000268164.3 | ST8 alpha-N-acetyl-neuraminide alpha-2,8-sialyltransferase 2 | 9 | 3 | 1 | 2 | 0 | 2 | bta-miR-2430 | -0.28 |
| PYCR2 | ENST00000343818.6 | pyrroline-5-carboxylate reductase family, member 2 | 1190 | 1 | 0 | 1 | 0 | 0 | bta-miR-2430 | -0.28 |
| PARP6 | ENST00000260376.7 | poly (ADP-ribose) polymerase family, member 6 | 867 | 1 | 0 | 1 | 0 | 0 | bta-miR-2430 | -0.27 |
| NUDCD3 | ENST00000355451.7 | NudC domain containing 3 | 659 | 2 | 1 | 0 | 1 | 0 | bta-miR-2430 | -0.27 |
| KREMEN1 | ENST00000400335.4 | kringle containing transmembrane protein 1 | 606 | 4 | 1 | 2 | 1 | 2 | bta-miR-2430 | -0.27 |
| MED27 | ENST00000357028.2 | mediator complex subunit 27 | 68 | 1 | 0 | 1 | 0 | 0 | bta-miR-2430 | -0.27 |
| PLAG1 | ENST00000316981.3 | pleiomorphic adenoma gene 1 | 58 | 1 | 1 | 0 | 0 | 2 | bta-miR-2430 | -0.27 |
| KIAA0513 | ENST00000566428.1 | KIAA0513 | 5 | 1 | 1 | 0 | 0 | 1 | bta-miR-2430 | -0.27 |
| TMEM97 | ENST00000226230.6 | transmembrane protein 97 | 10226 | 1 | 0 | 1 | 0 | 0 | bta-miR-2430 | -0.27 |
| MAPKBP1 | ENST00000457542.2 | mitogen-activated protein kinase binding protein 1 | 36 | 1 | 0 | 1 | 0 | 0 | bta-miR-2430 | -0.27 |
| MAGEA3 | ENST00000370278.3 | melanoma antigen family A, 3 | 5 | 1 | 1 | 0 | 0 | 0 | bta-miR-2430 | -0.27 |
| TCOF1 | ENST00000323668.7 | Treacher Collins-Franceschetti syndrome 1 | 325 | 2 | 1 | 1 | 0 | 0 | bta-miR-2430 | -0.27 |
| PSMB10 | ENST00000358514.4 | proteasome (prosome, macropain) subunit, beta type, 10 | 140 | 1 | 0 | 0 | 1 | 0 | bta-miR-2430 | -0.27 |
| DDX42 | ENST00000578681.1 | DEAD (Asp-Glu-Ala-Asp) box helicase 42 | 797 | 1 | 1 | 0 | 0 | 0 | bta-miR-2430 | -0.27 |
| OPN4 | ENST00000372071.2 | opsin 4 | 5 | 1 | 0 | 1 | 0 | 0 | bta-miR-2430 | -0.27 |
| RBBP8NL | ENST00000252998.1 | RBBP8 N-terminal like | 5 | 2 | 0 | 1 | 1 | 0 | bta-miR-2430 | -0.27 |
| FAM127B | ENST00000370775.2 | family with sequence similarity 127, member B | 647 | 2 | 0 | 1 | 1 | 0 | bta-miR-2430 | -0.27 |
| TAGLN | ENST00000532870.1 | transgelin | 225 | 1 | 0 | 1 | 0 | 0 | bta-miR-2430 | -0.27 |
| TAF7L | ENST00000372907.3 | TAF7-like RNA polymerase II, TATA box binding protein (TBP)-associated factor, 50kDa | 51 | 1 | 0 | 1 | 0 | 1 | bta-miR-2430 | -0.27 |
| RHOBTB3 | ENST00000379982.3 | Rho-related BTB domain containing 3 | 487 | 1 | 0 | 1 | 0 | 2 | bta-miR-2430 | -0.27 |
| C2CD4C | ENST00000332235.6 | C2 calcium-dependent domain containing 4C | 70 | 1 | 1 | 0 | 0 | 1 | bta-miR-2430 | -0.27 |
| UNC93A | ENST00000230256.3 | unc-93 homolog A (C. elegans) | 5 | 1 | 1 | 0 | 0 | 0 | bta-miR-2430 | -0.27 |
| PRPS1 | ENST00000372435.4 | phosphoribosyl pyrophosphate synthetase 1 | 2966 | 1 | 0 | 1 | 0 | 0 | bta-miR-2430 | -0.27 |
| TCF7L2 | ENST00000355717.4 | transcription factor 7-like 2 (T-cell specific, HMG-box) | 650 | 3 | 0 | 2 | 1 | 0 | bta-miR-2430 | -0.27 |
| SLC17A3 | ENST00000397060.4 | solute carrier family 17 (organic anion transporter), member 3 | 5 | 1 | 0 | 1 | 0 | 0 | bta-miR-2430 | -0.27 |
| CERS3 | ENST00000284382.4 | ceramide synthase 3 | 5 | 4 | 1 | 1 | 2 | 2 | bta-miR-2430 | -0.27 |
| CCM2L | ENST00000262659.8 | cerebral cavernous malformation 2-like | 7 | 1 | 1 | 0 | 0 | 0 | bta-miR-2430 | -0.27 |
| CARM1 | ENST00000327064.4 | coactivator-associated arginine methyltransferase 1 | 663 | 2 | 0 | 1 | 1 | 0 | bta-miR-2430 | -0.27 |
| ZNF551 | ENST00000282296.5 | zinc finger protein 551 | 49 | 2 | 0 | 2 | 0 | 0 | bta-miR-2430 | -0.27 |
| ARHGEF4 | ENST00000392953.3 | Rho guanine nucleotide exchange factor (GEF) 4 | 189 | 1 | 1 | 0 | 0 | 1 | bta-miR-2430 | -0.27 |
| COPS7B | ENST00000373608.3 | COP9 signalosome subunit 7B | 331 | 1 | 1 | 0 | 0 | 0 | bta-miR-2430 | -0.27 |
| PRRG2 | ENST00000246794.5 | proline rich Gla (G-carboxyglutamic acid) 2 | 8 | 1 | 0 | 1 | 0 | 1 | bta-miR-2430 | -0.27 |
| ST8SIA3 | ENST00000324000.3 | ST8 alpha-N-acetyl-neuraminide alpha-2,8-sialyltransferase 3 | 5 | 3 | 1 | 1 | 1 | 0 | bta-miR-2430 | -0.27 |
| FCAMR | ENST00000400962.3 | Fc receptor, IgA, IgM, high affinity | 5 | 1 | 0 | 1 | 0 | 1 | bta-miR-2430 | -0.27 |
| MAGEA8 | ENST00000535454.1 | melanoma antigen family A, 8 | 5 | 1 | 0 | 1 | 0 | 1 | bta-miR-2430 | -0.27 |
| ALDH1B1 | ENST00000377698.3 | aldehyde dehydrogenase 1 family, member B1 | 782 | 1 | 0 | 1 | 0 | 0 | bta-miR-2430 | -0.27 |
| KCNC1 | ENST00000379472.3 | potassium voltage-gated channel, Shaw-related subfamily, member 1 | 42 | 1 | 1 | 0 | 0 | 2 | bta-miR-2430 | -0.27 |
| MTERF | ENST00000419292.1 | mitochondrial transcription termination factor | 42 | 1 | 0 | 1 | 0 | 0 | bta-miR-2430 | -0.27 |
| AGAP1 | ENST00000304032.8 | ArfGAP with GTPase domain, ankyrin repeat and PH domain 1 | 109 | 2 | 0 | 0 | 2 | 3 | bta-miR-2430 | -0.27 |
| PLEKHH1 | ENST00000329153.5 | pleckstrin homology domain containing, family H (with MyTH4 domain) member 1 | 125 | 1 | 0 | 1 | 0 | 0 | bta-miR-2430 | -0.27 |
| KANSL2 | ENST00000550347.1 | KAT8 regulatory NSL complex subunit 2 | 68 | 1 | 0 | 1 | 0 | 0 | bta-miR-2430 | -0.27 |
| TMEM201 | ENST00000340305.5 | transmembrane protein 201 | 164 | 1 | 1 | 0 | 0 | 0 | bta-miR-2430 | -0.27 |
| ZNF687 | ENST00000368879.2 | zinc finger protein 687 | 1250 | 1 | 1 | 0 | 0 | 1 | bta-miR-2430 | -0.27 |
| AC009892.10 | ENST00000456337.1 | Uncharacterized protein | 5 | 1 | 0 | 1 | 0 | 0 | bta-miR-2430 | -0.27 |
| LCP1 | ENST00000398576.2 | lymphocyte cytosolic protein 1 (L-plastin) | 7 | 1 | 0 | 1 | 0 | 0 | bta-miR-2430 | -0.27 |
| PPM1L | ENST00000498165.1 | protein phosphatase, Mg2+/Mn2+ dependent, 1L | 59 | 1 | 0 | 1 | 0 | 1 | bta-miR-2430 | -0.27 |
| ADAT1 | ENST00000307921.3 | adenosine deaminase, tRNA-specific 1 | 597 | 2 | 0 | 1 | 1 | 1 | bta-miR-2430 | -0.27 |
| PLEKHM2 | ENST00000375799.3 | pleckstrin homology domain containing, family M (with RUN domain) member 2 | 349 | 2 | 1 | 1 | 0 | 0 | bta-miR-2430 | -0.27 |
| PPIH | ENST00000304979.3 | peptidylprolyl isomerase H (cyclophilin H) | 3787 | 1 | 0 | 0 | 1 | 0 | bta-miR-2430 | -0.27 |
| ZNF70 | ENST00000341976.3 | zinc finger protein 70 | 99 | 3 | 0 | 3 | 0 | 0 | bta-miR-2430 | -0.26 |
| BTN2A1 | ENST00000429381.1 | butyrophilin, subfamily 2, member A1 | 839 | 1 | 1 | 0 | 0 | 2 | bta-miR-2430 | -0.26 |
| KRTAP3-3 | ENST00000391586.1 | keratin associated protein 3-3 | 5 | 1 | 0 | 0 | 1 | 1 | bta-miR-2430 | -0.26 |
| UBE2J1 | ENST00000435041.2 | ubiquitin-conjugating enzyme E2, J1 | 1318 | 2 | 0 | 2 | 0 | 1 | bta-miR-2430 | -0.26 |
| SBSPON | ENST00000297354.6 | somatomedin B and thrombospondin, type 1 domain containing | 8 | 1 | 0 | 1 | 0 | 0 | bta-miR-2430 | -0.26 |
| HDLBP | ENST00000391975.1 | high density lipoprotein binding protein | 1755 | 1 | 0 | 1 | 0 | 0 | bta-miR-2430 | -0.26 |
| TRAPPC9 | ENST00000389328.4 | trafficking protein particle complex 9 | 251 | 2 | 1 | 1 | 0 | 2 | bta-miR-2430 | -0.26 |
| NPTXR | ENST00000333039.2 | neuronal pentraxin receptor | 578 | 3 | 1 | 1 | 1 | 3 | bta-miR-2430 | -0.26 |
| FOXA2 | ENST00000419308.2 | forkhead box A2 | 1207 | 1 | 1 | 0 | 0 | 0 | bta-miR-2430 | -0.26 |
| TSPYL6 | ENST00000317802.7 | TSPY-like 6 | 5 | 2 | 0 | 1 | 1 | 0 | bta-miR-2430 | -0.26 |
| BCL2L13 | ENST00000355028.3 | BCL2-like 13 (apoptosis facilitator) | 741 | 1 | 0 | 1 | 0 | 0 | bta-miR-2430 | -0.26 |
| KIRREL | ENST00000368172.1 | kin of IRRE like (Drosophila) | 493 | 3 | 0 | 3 | 0 | 1 | bta-miR-2430 | -0.26 |
| CD160 | ENST00000235933.6 | CD160 molecule | 5 | 1 | 0 | 1 | 0 | 0 | bta-miR-2430 | -0.26 |
| ATP5D | ENST00000215375.2 | ATP synthase, H+ transporting, mitochondrial F1 complex, delta subunit | 5503 | 1 | 0 | 1 | 0 | 0 | bta-miR-2430 | -0.26 |
| FANCE | ENST00000229769.2 | Fanconi anemia, complementation group E | 1562 | 1 | 1 | 0 | 0 | 0 | bta-miR-2430 | -0.26 |
| PLA2G2F | ENST00000375102.3 | phospholipase A2, group IIF | 5 | 1 | 1 | 0 | 0 | 1 | bta-miR-2430 | -0.26 |
| C4orf6 | ENST00000195455.2 | chromosome 4 open reading frame 6 | 5 | 1 | 0 | 0 | 1 | 0 | bta-miR-2430 | -0.26 |
| ECEL1 | ENST00000304546.1 | endothelin converting enzyme-like 1 | 6 | 1 | 1 | 0 | 0 | 0 | bta-miR-2430 | -0.26 |
| ZFP14 | ENST00000270001.7 | ZFP14 zinc finger protein | 109 | 2 | 1 | 0 | 1 | 0 | bta-miR-2430 | -0.26 |
| C1orf226 | ENST00000458626.2 | chromosome 1 open reading frame 226 | 369 | 4 | 1 | 2 | 1 | 0 | bta-miR-2430 | -0.26 |
| TMTC2 | ENST00000321196.3 | transmembrane and tetratricopeptide repeat containing 2 | 173 | 2 | 0 | 1 | 1 | 0 | bta-miR-2430 | -0.26 |
| CAP2 | ENST00000229922.2 | CAP, adenylate cyclase-associated protein, 2 (yeast) | 2952 | 1 | 0 | 1 | 0 | 0 | bta-miR-2430 | -0.26 |
| TNFRSF13C | ENST00000291232.3 | tumor necrosis factor receptor superfamily, member 13C | 36 | 4 | 1 | 2 | 1 | 1 | bta-miR-2430 | -0.26 |
| FNIP2 | ENST00000264433.6 | folliculin interacting protein 2 | 266 | 2 | 0 | 2 | 0 | 0 | bta-miR-2430 | -0.26 |
| SGSM1 | ENST00000400358.4 | small G protein signaling modulator 1 | 25 | 2 | 0 | 2 | 0 | 0 | bta-miR-2430 | -0.26 |
| ZNF646 | ENST00000300850.5 | zinc finger protein 646 | 5 | 1 | 1 | 0 | 0 | 1 | bta-miR-2430 | -0.26 |
| SIRPA | ENST00000400068.3 | signal-regulatory protein alpha | 1235 | 2 | 0 | 1 | 1 | 1 | bta-miR-2430 | -0.26 |
| NUDT5 | ENST00000491614.1 | nudix (nucleoside diphosphate linked moiety X)-type motif 5 | 808 | 1 | 1 | 0 | 0 | 0 | bta-miR-2430 | -0.26 |
| LNP1 | ENST00000383693.3 | leukemia NUP98 fusion partner 1 | 50 | 1 | 0 | 1 | 0 | 0 | bta-miR-2430 | -0.26 |
| GRM2 | ENST00000395052.3 | glutamate receptor, metabotropic 2 | 5 | 1 | 0 | 1 | 0 | 0 | bta-miR-2430 | -0.26 |
| ENO4 | ENST00000409522.1 | enolase family member 4 | 5 | 1 | 0 | 1 | 0 | 0 | bta-miR-2430 | -0.26 |
| NR0B2 | ENST00000254227.3 | nuclear receptor subfamily 0, group B, member 2 | 266 | 1 | 0 | 1 | 0 | 0 | bta-miR-2430 | -0.26 |
| RAD51D | ENST00000345365.6 | RAD51 paralog D | 73 | 2 | 2 | 0 | 0 | 0 | bta-miR-2430 | -0.26 |
| GADL1 | ENST00000454381.3 | glutamate decarboxylase-like 1 | 5 | 1 | 0 | 1 | 0 | 0 | bta-miR-2430 | -0.26 |
| TEX40 | ENST00000539943.1 | testis expressed 40 | 8 | 1 | 0 | 0 | 1 | 0 | bta-miR-2430 | -0.26 |
| FAM43A | ENST00000329759.4 | family with sequence similarity 43, member A | 287 | 1 | 0 | 1 | 0 | 0 | bta-miR-2430 | -0.26 |
| SYNGAP1 | ENST00000418600.2 | synaptic Ras GTPase activating protein 1 | 15 | 3 | 0 | 1 | 2 | 1 | bta-miR-2430 | -0.26 |
| MAF1 | ENST00000322428.5 | MAF1 homolog (S. cerevisiae) | 698 | 1 | 0 | 1 | 0 | 1 | bta-miR-2430 | -0.26 |
| DHX8 | ENST00000540306.1 | DEAH (Asp-Glu-Ala-His) box polypeptide 8 | 91 | 1 | 0 | 1 | 0 | 0 | bta-miR-2430 | -0.26 |
| FFAR2 | ENST00000599180.2 | free fatty acid receptor 2 | 5 | 1 | 0 | 1 | 0 | 0 | bta-miR-2430 | -0.26 |
| AGPAT4 | ENST00000366911.5 | 1-acylglycerol-3-phosphate O-acyltransferase 4 | 142 | 2 | 0 | 1 | 1 | 1 | bta-miR-2430 | -0.25 |
| TTC39C | ENST00000540918.2 | tetratricopeptide repeat domain 39C | 494 | 2 | 0 | 1 | 1 | 0 | bta-miR-2430 | -0.25 |
| CDH4 | ENST00000360469.5 | cadherin 4, type 1, R-cadherin (retinal) | 33 | 1 | 1 | 0 | 0 | 3 | bta-miR-2430 | -0.25 |
| ARHGEF16 | ENST00000378378.4 | Rho guanine nucleotide exchange factor (GEF) 16 | 72 | 1 | 0 | 1 | 0 | 0 | bta-miR-2430 | -0.25 |
| CRY2 | ENST00000443527.2 | cryptochrome 2 (photolyase-like) | 63 | 1 | 1 | 0 | 0 | 0 | bta-miR-2430 | -0.25 |
| C11orf80 | ENST00000360962.4 | chromosome 11 open reading frame 80 | 697 | 1 | 0 | 1 | 0 | 0 | bta-miR-2430 | -0.25 |
| GLP1R | ENST00000373256.4 | glucagon-like peptide 1 receptor | 5 | 1 | 1 | 0 | 0 | 0 | bta-miR-2430 | -0.25 |
| FAM168A | ENST00000064778.4 | family with sequence similarity 168, member A | 68 | 3 | 0 | 2 | 1 | 2 | bta-miR-2430 | -0.25 |
| FNDC7 | ENST00000271311.2 | fibronectin type III domain containing 7 | 5 | 2 | 0 | 1 | 1 | 0 | bta-miR-2430 | -0.25 |
| OBP2B | ENST00000372032.2 | odorant binding protein 2B | 5 | 1 | 0 | 0 | 1 | 0 | bta-miR-2430 | -0.25 |
| NRARP | ENST00000356628.2 | NOTCH-regulated ankyrin repeat protein | 56 | 1 | 0 | 1 | 0 | 0 | bta-miR-2430 | -0.25 |
| AL163636.6 | ENST00000553909.1 | Homo sapiens ribonuclease, RNase A family, 4 (RNASE4), transcript variant 4, mRNA. | 103 | 1 | 0 | 1 | 0 | 0 | bta-miR-2430 | -0.25 |
| SAMD7 | ENST00000428432.2 | sterile alpha motif domain containing 7 | 5 | 1 | 0 | 0 | 1 | 0 | bta-miR-2430 | -0.25 |
| RFX2 | ENST00000303657.5 | regulatory factor X, 2 (influences HLA class II expression) | 148 | 2 | 0 | 1 | 1 | 1 | bta-miR-2430 | -0.25 |
| TCP11X2 | ENST00000453326.2 | t-complex 11 family, X-linked 2 | 5 | 1 | 0 | 1 | 0 | 0 | bta-miR-2430 | -0.25 |
| RASAL2 | ENST00000448150.3 | RAS protein activator like 2 | 950 | 2 | 0 | 2 | 0 | 2 | bta-miR-2430 | -0.25 |
| CSDE1 | ENST00000339438.6 | cold shock domain containing E1, RNA-binding | 435 | 1 | 0 | 1 | 0 | 0 | bta-miR-2430 | -0.25 |
| C18orf25 | ENST00000282059.6 | chromosome 18 open reading frame 25 | 688 | 1 | 1 | 0 | 0 | 0 | bta-miR-2430 | -0.25 |
| C9orf114 | ENST00000361256.5 | chromosome 9 open reading frame 114 | 488 | 3 | 0 | 1 | 2 | 1 | bta-miR-2430 | -0.25 |
| COL6A2 | ENST00000300527.4 | collagen, type VI, alpha 2 | 9107 | 1 | 0 | 0 | 1 | 1 | bta-miR-2430 | -0.25 |
| CRP | ENST00000255030.5 | C-reactive protein, pentraxin-related | 5 | 1 | 0 | 1 | 0 | 0 | bta-miR-2430 | -0.25 |
| ZNFX1 | ENST00000371752.1 | zinc finger, NFX1-type containing 1 | 41 | 1 | 0 | 1 | 0 | 1 | bta-miR-2430 | -0.25 |
| MYBPC2 | ENST00000357701.5 | myosin binding protein C, fast type | 25 | 1 | 0 | 0 | 1 | 0 | bta-miR-2430 | -0.25 |
| SLC5A2 | ENST00000330498.3 | solute carrier family 5 (sodium/glucose cotransporter), member 2 | 5 | 1 | 0 | 1 | 0 | 0 | bta-miR-2430 | -0.25 |
| GIMAP1 | ENST00000307194.5 | GTPase, IMAP family member 1 | 5 | 1 | 0 | 1 | 0 | 0 | bta-miR-2430 | -0.25 |
| BCAR3 | ENST00000370247.3 | breast cancer anti-estrogen resistance 3 | 321 | 1 | 0 | 1 | 0 | 0 | bta-miR-2430 | -0.25 |
| MED29 | ENST00000315588.5 | mediator complex subunit 29 | 2453 | 5 | 1 | 2 | 2 | 5 | bta-miR-2430 | -0.25 |
| ARHGAP25 | ENST00000409202.3 | Rho GTPase activating protein 25 | 5 | 1 | 0 | 1 | 0 | 0 | bta-miR-2430 | -0.25 |
| BAI1 | ENST00000517894.1 | brain-specific angiogenesis inhibitor 1 | 5 | 1 | 0 | 1 | 0 | 1 | bta-miR-2430 | -0.25 |
| WDR81 | ENST00000437219.2 | WD repeat domain 81 | 1387 | 1 | 0 | 1 | 0 | 1 | bta-miR-2430 | -0.25 |
| NDUFV3 | ENST00000340344.4 | NADH dehydrogenase (ubiquinone) flavoprotein 3, 10kDa | 1449 | 1 | 0 | 1 | 0 | 1 | bta-miR-2430 | -0.25 |
| LAS1L | ENST00000312391.8 | LAS1-like (S. cerevisiae) | 26 | 3 | 1 | 1 | 1 | 1 | bta-miR-2430 | -0.25 |
| ZMYM4 | ENST00000314607.6 | zinc finger, MYM-type 4 | 280 | 1 | 0 | 1 | 0 | 0 | bta-miR-2430 | -0.25 |
| HSD17B4 | ENST00000256216.6 | hydroxysteroid (17-beta) dehydrogenase 4 | 204 | 1 | 0 | 0 | 1 | 1 | bta-miR-2430 | -0.25 |
| HDGFL1 | ENST00000510882.2 | hepatoma derived growth factor-like 1 | 5 | 1 | 0 | 1 | 0 | 1 | bta-miR-2430 | -0.25 |
| PHF2 | ENST00000375376.4 | PHD finger protein 2 | 151 | 1 | 0 | 1 | 0 | 1 | bta-miR-2430 | -0.25 |
| CACTIN | ENST00000429344.2 | cactin, spliceosome C complex subunit | 1422 | 3 | 0 | 2 | 1 | 0 | bta-miR-2430 | -0.25 |
| MCM9 | ENST00000316068.3 | minichromosome maintenance complex component 9 | 17 | 1 | 0 | 1 | 0 | 0 | bta-miR-2430 | -0.25 |
| ZNF787 | ENST00000270459.3 | zinc finger protein 787 | 191 | 1 | 1 | 0 | 0 | 0 | bta-miR-2430 | -0.25 |
| SYNGR1 | ENST00000328933.5 | synaptogyrin 1 | 101 | 2 | 0 | 2 | 0 | 3 | bta-miR-2430 | -0.25 |
| STOML1 | ENST00000564777.1 | stomatin (EPB72)-like 1 | 18 | 3 | 0 | 3 | 0 | 0 | bta-miR-2430 | -0.25 |
| GNA15 | ENST00000262958.3 | guanine nucleotide binding protein (G protein), alpha 15 (Gq class) | 5 | 1 | 0 | 1 | 0 | 0 | bta-miR-2430 | -0.25 |
| PACSIN3 | ENST00000298838.6 | protein kinase C and casein kinase substrate in neurons 3 | 55 | 2 | 0 | 2 | 0 | 0 | bta-miR-2430 | -0.25 |
| KLF13 | ENST00000307145.3 | Kruppel-like factor 13 | 5 | 3 | 0 | 3 | 0 | 2 | bta-miR-2430 | -0.25 |
| NOS1AP | ENST00000361897.5 | nitric oxide synthase 1 (neuronal) adaptor protein | 5 | 2 | 1 | 1 | 0 | 2 | bta-miR-2430 | -0.25 |
| DKC1 | ENST00000369550.5 | dyskeratosis congenita 1, dyskerin | 3493 | 1 | 0 | 1 | 0 | 0 | bta-miR-2430 | -0.25 |
| IL17A | ENST00000340057.1 | interleukin 17A | 5 | 1 | 0 | 1 | 0 | 0 | bta-miR-2430 | -0.25 |
| ZNF211 | ENST00000347302.3 | zinc finger protein 211 | 420 | 1 | 0 | 1 | 0 | 0 | bta-miR-2430 | -0.25 |
| CHDH | ENST00000315251.6 | choline dehydrogenase | 249 | 3 | 1 | 0 | 2 | 1 | bta-miR-2430 | -0.25 |
| MCU | ENST00000373053.3 | mitochondrial calcium uniporter | 2421 | 3 | 0 | 1 | 2 | 0 | bta-miR-2430 | -0.25 |
| MRPL35 | ENST00000337109.4 | mitochondrial ribosomal protein L35 | 860 | 1 | 0 | 0 | 1 | 2 | bta-miR-2430 | -0.25 |
| CYS1 | ENST00000381813.4 | cystin 1 | 22 | 2 | 0 | 2 | 0 | 0 | bta-miR-2430 | -0.25 |
| SLC4A9 | ENST00000506757.2 | solute carrier family 4, sodium bicarbonate cotransporter, member 9 | 5 | 1 | 0 | 1 | 0 | 0 | bta-miR-2430 | -0.25 |
| TLR4 | ENST00000355622.6 | toll-like receptor 4 | 47 | 1 | 1 | 0 | 0 | 1 | bta-miR-2430 | -0.25 |
| KRT79 | ENST00000330553.5 | keratin 79 | 5 | 1 | 0 | 1 | 0 | 1 | bta-miR-2430 | -0.25 |
| NUMA1 | ENST00000351960.6 | nuclear mitotic apparatus protein 1 | 7 | 1 | 0 | 1 | 0 | 0 | bta-miR-2430 | -0.25 |
| DAB1 | ENST00000371236.2 | Dab, reelin signal transducer, homolog 1 (Drosophila) | 490 | 1 | 0 | 1 | 0 | 1 | bta-miR-2430 | -0.25 |
| SLC12A4 | ENST00000422611.2 | solute carrier family 12 (potassium/chloride transporter), member 4 | 1618 | 1 | 0 | 1 | 0 | 0 | bta-miR-2430 | -0.25 |
| KIAA1715 | ENST00000272748.4 | KIAA1715 | 858 | 1 | 1 | 0 | 0 | 0 | bta-miR-2430 | -0.25 |
| AP001579.1 | ENST00000599569.1 | Uncharacterized protein | 47 | 2 | 0 | 1 | 1 | 1 | bta-miR-2430 | -0.25 |
| DUOXA1 | ENST00000558996.1 | dual oxidase maturation factor 1 | 5 | 1 | 0 | 1 | 0 | 0 | bta-miR-2430 | -0.24 |
| ITGA5 | ENST00000293379.4 | integrin, alpha 5 (fibronectin receptor, alpha polypeptide) | 5599 | 1 | 1 | 0 | 0 | 0 | bta-miR-2430 | -0.24 |
| RNF135 | ENST00000535306.2 | ring finger protein 135 | 65 | 1 | 0 | 1 | 0 | 0 | bta-miR-2430 | -0.24 |
| FYCO1 | ENST00000296137.2 | FYVE and coiled-coil domain containing 1 | 91 | 4 | 0 | 2 | 2 | 1 | bta-miR-2430 | -0.24 |
| PELI2 | ENST00000267460.4 | pellino E3 ubiquitin protein ligase family member 2 | 229 | 2 | 0 | 1 | 1 | 0 | bta-miR-2430 | -0.24 |
| ZNF264 | ENST00000263095.6 | zinc finger protein 264 | 412 | 1 | 0 | 1 | 0 | 3 | bta-miR-2430 | -0.24 |
| MLC1 | ENST00000395876.2 | megalencephalic leukoencephalopathy with subcortical cysts 1 | 5 | 1 | 0 | 1 | 0 | 1 | bta-miR-2430 | -0.24 |
| CAPN14 | ENST00000444918.2 | calpain 14 | 5 | 2 | 0 | 0 | 2 | 0 | bta-miR-2430 | -0.24 |
| C6orf47 | ENST00000375911.1 | chromosome 6 open reading frame 47 | 38 | 1 | 0 | 1 | 0 | 0 | bta-miR-2430 | -0.24 |
| EIF3F | ENST00000533626.1 | eukaryotic translation initiation factor 3, subunit F | 64 | 1 | 1 | 0 | 0 | 1 | bta-miR-2430 | -0.24 |
| HS3ST4 | ENST00000331351.5 | heparan sulfate (glucosamine) 3-O-sulfotransferase 4 | 5 | 1 | 0 | 1 | 0 | 0 | bta-miR-2430 | -0.24 |
| NAA60 | ENST00000360862.5 | N(alpha)-acetyltransferase 60, NatF catalytic subunit | 1339 | 1 | 0 | 1 | 0 | 0 | bta-miR-2430 | -0.24 |
| AQP3 | ENST00000297991.4 | aquaporin 3 (Gill blood group) | 70 | 1 | 0 | 1 | 0 | 0 | bta-miR-2430 | -0.24 |
| C1orf122 | ENST00000373042.4 | chromosome 1 open reading frame 122 | 435 | 1 | 0 | 0 | 1 | 1 | bta-miR-2430 | -0.24 |
| CD44 | ENST00000278386.6 | CD44 molecule (Indian blood group) | 1543 | 2 | 1 | 1 | 0 | 0 | bta-miR-2430 | -0.24 |
| CCDC103 | ENST00000417826.2 | coiled-coil domain containing 103 | 72 | 1 | 0 | 1 | 0 | 1 | bta-miR-2430 | -0.24 |
| MYLIP | ENST00000349606.4 | myosin regulatory light chain interacting protein | 452 | 1 | 1 | 0 | 0 | 1 | bta-miR-2430 | -0.24 |
| FAM86B1 | ENST00000448228.2 | family with sequence similarity 86, member B1 | 5 | 1 | 0 | 1 | 0 | 1 | bta-miR-2430 | -0.24 |
| RASGEF1C | ENST00000361132.4 | RasGEF domain family, member 1C | 5 | 1 | 0 | 1 | 0 | 0 | bta-miR-2430 | -0.24 |
| ODF3L1 | ENST00000332145.2 | outer dense fiber of sperm tails 3-like 1 | 5 | 1 | 0 | 0 | 1 | 0 | bta-miR-2430 | -0.24 |
| GRSF1 | ENST00000254799.6 | G-rich RNA sequence binding factor 1 | 4414 | 1 | 1 | 0 | 0 | 0 | bta-miR-2430 | -0.24 |
| NWD1 | ENST00000524140.2 | NACHT and WD repeat domain containing 1 | 5 | 1 | 1 | 0 | 0 | 0 | bta-miR-2430 | -0.24 |
| ITPKB | ENST00000272117.3 | inositol-trisphosphate 3-kinase B | 5 | 2 | 0 | 1 | 1 | 4 | bta-miR-2430 | -0.24 |
| LY6E | ENST00000517503.1 | lymphocyte antigen 6 complex, locus E | 342 | 1 | 0 | 1 | 0 | 0 | bta-miR-2430 | -0.24 |
| DNAJC11 | ENST00000377577.5 | DnaJ (Hsp40) homolog, subfamily C, member 11 | 361 | 1 | 0 | 0 | 1 | 1 | bta-miR-2430 | -0.24 |
| PLOD1 | ENST00000196061.4 | procollagen-lysine, 2-oxoglutarate 5-dioxygenase 1 | 5056 | 1 | 0 | 1 | 0 | 1 | bta-miR-2430 | -0.24 |
| UBE4A | ENST00000252108.3 | ubiquitination factor E4A | 29 | 1 | 1 | 0 | 0 | 0 | bta-miR-2430 | -0.24 |
| GALNT6 | ENST00000543196.2 | UDP-N-acetyl-alpha-D-galactosamine:polypeptide N-acetylgalactosaminyltransferase 6 (GalNAc-T6) | 83 | 2 | 1 | 0 | 1 | 1 | bta-miR-2430 | -0.24 |
| ZYG11B | ENST00000294353.6 | zyg-11 family member B, cell cycle regulator | 975 | 2 | 0 | 2 | 0 | 0 | bta-miR-2430 | -0.24 |
| PDPR | ENST00000568530.1 | pyruvate dehydrogenase phosphatase regulatory subunit | 69 | 2 | 0 | 2 | 0 | 0 | bta-miR-2430 | -0.24 |
| SPRY3 | ENST00000302805.2 | sprouty homolog 3 (Drosophila) | 5 | 2 | 0 | 1 | 1 | 0 | bta-miR-2430 | -0.24 |
| DGKH | ENST00000261491.5 | diacylglycerol kinase, eta | 46 | 3 | 1 | 1 | 1 | 4 | bta-miR-2430 | -0.24 |
| EXOC2 | ENST00000230449.4 | exocyst complex component 2 | 192 | 2 | 1 | 0 | 1 | 0 | bta-miR-2430 | -0.24 |
| MSR1 | ENST00000350896.3 | macrophage scavenger receptor 1 | 5 | 2 | 0 | 0 | 2 | 0 | bta-miR-2430 | -0.24 |
| RAD51B | ENST00000487270.1 | RAD51 paralog B | 24 | 1 | 0 | 1 | 0 | 0 | bta-miR-2430 | -0.24 |
| MAATS1 | ENST00000273390.5 | MYCBP-associated, testis expressed 1 | 5 | 1 | 0 | 1 | 0 | 0 | bta-miR-2430 | -0.24 |
| C7orf31 | ENST00000409280.1 | chromosome 7 open reading frame 31 | 40 | 1 | 0 | 0 | 1 | 1 | bta-miR-2430 | -0.24 |
| SPSB1 | ENST00000328089.6 | splA/ryanodine receptor domain and SOCS box containing 1 | 572 | 1 | 0 | 1 | 0 | 1 | bta-miR-2430 | -0.24 |
| DDX23 | ENST00000308025.3 | DEAD (Asp-Glu-Ala-Asp) box polypeptide 23 | 170 | 1 | 0 | 1 | 0 | 1 | bta-miR-2430 | -0.24 |
| PDXP | ENST00000215904.6 | pyridoxal (pyridoxine, vitamin B6) phosphatase | 58 | 1 | 0 | 1 | 0 | 1 | bta-miR-2430 | -0.24 |
| TSC22D2 | ENST00000361875.3 | TSC22 domain family, member 2 | 1062 | 1 | 0 | 1 | 0 | 0 | bta-miR-2430 | -0.24 |
| C15orf62 | ENST00000344320.6 | chromosome 15 open reading frame 62 | 8 | 1 | 1 | 0 | 0 | 0 | bta-miR-2430 | -0.24 |
| PDCD11 | ENST00000369797.3 | programmed cell death 11 | 1551 | 1 | 0 | 1 | 0 | 0 | bta-miR-2430 | -0.24 |
| SLC10A7 | ENST00000264986.3 | solute carrier family 10, member 7 | 77 | 1 | 0 | 1 | 0 | 0 | bta-miR-2430 | -0.24 |
| C19orf55 | ENST00000544099.1 | chromosome 19 open reading frame 55 | 59 | 2 | 1 | 1 | 0 | 0 | bta-miR-2430 | -0.24 |
| MRPL43 | ENST00000477279.1 | mitochondrial ribosomal protein L43 | 502 | 1 | 0 | 1 | 0 | 0 | bta-miR-2430 | -0.24 |
| JAK1 | ENST00000342505.4 | Janus kinase 1 | 139 | 2 | 0 | 1 | 1 | 0 | bta-miR-2430 | -0.24 |
| KCNIP2 | ENST00000348850.5 | Kv channel interacting protein 2 | 5 | 2 | 0 | 0 | 2 | 2 | bta-miR-2430 | -0.24 |
| ZNF624 | ENST00000311331.7 | zinc finger protein 624 | 167 | 1 | 0 | 1 | 0 | 1 | bta-miR-2430 | -0.24 |
| CHEK2 | ENST00000382566.1 | checkpoint kinase 2 | 5 | 1 | 0 | 1 | 0 | 0 | bta-miR-2430 | -0.24 |
| MIEF1 | ENST00000325301.2 | mitochondrial elongation factor 1 | 2319 | 1 | 1 | 0 | 0 | 3 | bta-miR-2430 | -0.24 |
| MT-ND5 | ENST00000361567.2 | mitochondrially encoded NADH dehydrogenase 5 | 675 | 1 | 0 | 0 | 1 | 1 | bta-miR-2430 | -0.24 |
| IQGAP2 | ENST00000274364.6 | IQ motif containing GTPase activating protein 2 | 588 | 1 | 1 | 0 | 0 | 0 | bta-miR-2430 | -0.24 |
| NECAB1 | ENST00000417640.2 | N-terminal EF-hand calcium binding protein 1 | 5 | 1 | 0 | 1 | 0 | 0 | bta-miR-2430 | -0.23 |
| ABCG8 | ENST00000272286.2 | ATP-binding cassette, sub-family G (WHITE), member 8 | 197 | 1 | 1 | 0 | 0 | 1 | bta-miR-2430 | -0.23 |
| SLC13A5 | ENST00000433363.2 | solute carrier family 13 (sodium-dependent citrate transporter), member 5 | 4848 | 1 | 0 | 1 | 0 | 0 | bta-miR-2430 | -0.23 |
| ZNF627 | ENST00000361113.5 | zinc finger protein 627 | 105 | 1 | 0 | 1 | 0 | 0 | bta-miR-2430 | -0.23 |
| COL25A1 | ENST00000399132.1 | collagen, type XXV, alpha 1 | 270 | 2 | 0 | 1 | 1 | 0 | bta-miR-2430 | -0.23 |
| FBXO31 | ENST00000311635.7 | F-box protein 31 | 48 | 5 | 0 | 3 | 2 | 0 | bta-miR-2430 | -0.23 |
| SPATA5 | ENST00000274008.4 | spermatogenesis associated 5 | 168 | 2 | 0 | 2 | 0 | 0 | bta-miR-2430 | -0.23 |
| FADS3 | ENST00000540820.1 | fatty acid desaturase 3 | 62 | 1 | 0 | 0 | 1 | 1 | bta-miR-2430 | -0.23 |
| AP4M1 | ENST00000359593.4 | adaptor-related protein complex 4, mu 1 subunit | 163 | 1 | 0 | 1 | 0 | 1 | bta-miR-2430 | -0.23 |
| SPSB3 | ENST00000566339.1 | splA/ryanodine receptor domain and SOCS box containing 3 | 827 | 1 | 0 | 1 | 0 | 0 | bta-miR-2430 | -0.23 |
| UBE2H | ENST00000355621.3 | ubiquitin-conjugating enzyme E2H | 855 | 1 | 0 | 1 | 0 | 0 | bta-miR-2430 | -0.23 |
| YES1 | ENST00000577961.1 | v-yes-1 Yamaguchi sarcoma viral oncogene homolog 1 | 168 | 1 | 1 | 0 | 0 | 0 | bta-miR-2430 | -0.23 |
| LRFN3 | ENST00000588831.1 | leucine rich repeat and fibronectin type III domain containing 3 | 207 | 2 | 1 | 1 | 0 | 0 | bta-miR-2430 | -0.23 |
| KIAA1456 | ENST00000524591.2 | KIAA1456 | 24 | 2 | 0 | 1 | 1 | 0 | bta-miR-2430 | -0.23 |
| SRSF10 | ENST00000343255.5 | serine/arginine-rich splicing factor 10 | 1920 | 1 | 0 | 1 | 0 | 3 | bta-miR-2430 | -0.23 |
| ARHGEF3 | ENST00000296315.3 | Rho guanine nucleotide exchange factor (GEF) 3 | 359 | 1 | 0 | 1 | 0 | 0 | bta-miR-2430 | -0.23 |
| IL4R | ENST00000395762.2 | interleukin 4 receptor | 239 | 1 | 0 | 1 | 0 | 0 | bta-miR-2430 | -0.23 |
| TNK2 | ENST00000381916.2 | tyrosine kinase, non-receptor, 2 | 7 | 1 | 0 | 1 | 0 | 0 | bta-miR-2430 | -0.23 |
| ZNF275 | ENST00000370251.3 | zinc finger protein 275 | 465 | 2 | 1 | 1 | 0 | 2 | bta-miR-2430 | -0.23 |
| TSFM | ENST00000540550.1 | Ts translation elongation factor, mitochondrial | 1340 | 1 | 0 | 0 | 1 | 0 | bta-miR-2430 | -0.23 |
| RALGAPA1 | ENST00000389698.3 | Ral GTPase activating protein, alpha subunit 1 (catalytic) | 82 | 2 | 0 | 1 | 1 | 1 | bta-miR-2430 | -0.23 |
| FAM212B | ENST00000357260.5 | family with sequence similarity 212, member B | 211 | 2 | 0 | 1 | 1 | 4 | bta-miR-2430 | -0.23 |
| RCAN2 | ENST00000330430.6 | regulator of calcineurin 2 | 32 | 1 | 0 | 1 | 0 | 0 | bta-miR-2430 | -0.23 |
| ZEB1 | ENST00000361642.5 | zinc finger E-box binding homeobox 1 | 59 | 1 | 0 | 1 | 0 | 1 | bta-miR-2430 | -0.23 |
| FMNL1 | ENST00000331495.3 | formin-like 1 | 33 | 1 | 0 | 1 | 0 | 0 | bta-miR-2430 | -0.23 |
| RASGRF1 | ENST00000558480.2 | Ras protein-specific guanine nucleotide-releasing factor 1 | 5 | 1 | 1 | 0 | 0 | 0 | bta-miR-2430 | -0.23 |
| TRIM26 | ENST00000437089.1 | tripartite motif containing 26 | 102 | 1 | 0 | 1 | 0 | 0 | bta-miR-2430 | -0.23 |
| CCDC28B | ENST00000421922.2 | coiled-coil domain containing 28B | 106 | 1 | 0 | 1 | 0 | 1 | bta-miR-2430 | -0.23 |
| MAP3K6 | ENST00000374040.3 | mitogen-activated protein kinase kinase kinase 6 | 25 | 1 | 0 | 1 | 0 | 0 | bta-miR-2430 | -0.23 |
| CDRT4 | ENST00000312177.6 | CMT1A duplicated region transcript 4 | 109 | 1 | 0 | 1 | 0 | 5 | bta-miR-2430 | -0.23 |
| DSE | ENST00000452085.3 | dermatan sulfate epimerase | 975 | 1 | 0 | 1 | 0 | 0 | bta-miR-2430 | -0.23 |
| ZNF844 | ENST00000439326.3 | zinc finger protein 844 | 11 | 1 | 0 | 1 | 0 | 0 | bta-miR-2430 | -0.23 |
| NTN1 | ENST00000173229.2 | netrin 1 | 12 | 1 | 1 | 0 | 0 | 2 | bta-miR-2430 | -0.23 |
| SKOR1 | ENST00000341418.5 | SKI family transcriptional corepressor 1 | 22 | 1 | 0 | 1 | 0 | 0 | bta-miR-2430 | -0.23 |
| NUDT18 | ENST00000522405.1 | nudix (nucleoside diphosphate linked moiety X)-type motif 18 | 40 | 1 | 0 | 1 | 0 | 0 | bta-miR-2430 | -0.23 |
| FOXB1 | ENST00000396057.4 | forkhead box B1 | 31 | 1 | 0 | 1 | 0 | 1 | bta-miR-2430 | -0.23 |
| NAA60 | ENST00000610180.1 | N-alpha-acetyltransferase 60 | 1339 | 1 | 0 | 1 | 0 | 0 | bta-miR-2430 | -0.23 |
| BARX2 | ENST00000281437.4 | BARX homeobox 2 | 5 | 1 | 0 | 1 | 0 | 0 | bta-miR-2430 | -0.23 |
| OGFRL1 | ENST00000370435.4 | opioid growth factor receptor-like 1 | 607 | 1 | 0 | 1 | 0 | 1 | bta-miR-2430 | -0.23 |
| SIGIRR | ENST00000531205.1 | single immunoglobulin and toll-interleukin 1 receptor (TIR) domain | 80 | 1 | 0 | 1 | 0 | 0 | bta-miR-2430 | -0.23 |
| STAT3 | ENST00000585517.1 | signal transducer and activator of transcription 3 (acute-phase response factor) | 478 | 1 | 1 | 0 | 0 | 0 | bta-miR-2430 | -0.23 |
| CERS1 | ENST00000427170.2 | ceramide synthase 1 | 167 | 2 | 0 | 2 | 0 | 0 | bta-miR-2430 | -0.23 |
| RASGRP4 | ENST00000586305.1 | RAS guanyl releasing protein 4 | 5 | 1 | 0 | 1 | 0 | 0 | bta-miR-2430 | -0.23 |
| ELOVL2 | ENST00000354666.3 | ELOVL fatty acid elongase 2 | 99 | 1 | 1 | 0 | 0 | 1 | bta-miR-2430 | -0.23 |
| FOXN3 | ENST00000345097.4 | forkhead box N3 | 42 | 1 | 0 | 1 | 0 | 3 | bta-miR-2430 | -0.23 |
| SMARCD1 | ENST00000394963.4 | SWI/SNF related, matrix associated, actin dependent regulator of chromatin, subfamily d, member 1 | 952 | 1 | 0 | 1 | 0 | 1 | bta-miR-2430 | -0.23 |
| GTPBP3 | ENST00000324894.8 | GTP binding protein 3 (mitochondrial) | 1451 | 1 | 0 | 1 | 0 | 0 | bta-miR-2430 | -0.23 |
| LRRC20 | ENST00000355790.4 | leucine rich repeat containing 20 | 159 | 2 | 1 | 0 | 1 | 1 | bta-miR-2430 | -0.23 |
| TIGIT | ENST00000486257.1 | T cell immunoreceptor with Ig and ITIM domains | 5 | 1 | 0 | 1 | 0 | 0 | bta-miR-2430 | -0.23 |
| CTRC | ENST00000375943.2 | chymotrypsin C (caldecrin) | 5 | 1 | 0 | 1 | 0 | 0 | bta-miR-2430 | -0.23 |
| SCRT2 | ENST00000246104.6 | scratch homolog 2, zinc finger protein (Drosophila) | 5 | 1 | 0 | 0 | 1 | 1 | bta-miR-2430 | -0.23 |
| PPP2R1B | ENST00000527614.1 | protein phosphatase 2, regulatory subunit A, beta | 341 | 2 | 1 | 1 | 0 | 0 | bta-miR-2430 | -0.23 |
| FAM76A | ENST00000373954.6 | family with sequence similarity 76, member A | 173 | 1 | 0 | 1 | 0 | 0 | bta-miR-2430 | -0.22 |
| LBH | ENST00000395323.3 | limb bud and heart development | 1956 | 2 | 0 | 2 | 0 | 0 | bta-miR-2430 | -0.22 |
| SNX11 | ENST00000393405.2 | sorting nexin 11 | 20 | 1 | 0 | 1 | 0 | 1 | bta-miR-2430 | -0.22 |
| TNFRSF19 | ENST00000382263.3 | tumor necrosis factor receptor superfamily, member 19 | 376 | 1 | 0 | 1 | 0 | 0 | bta-miR-2430 | -0.22 |
| CD177 | ENST00000378009.4 | CD177 molecule | 5 | 1 | 0 | 1 | 0 | 0 | bta-miR-2430 | -0.22 |
| TMOD2 | ENST00000249700.4 | tropomodulin 2 (neuronal) | 117 | 1 | 1 | 0 | 0 | 0 | bta-miR-2430 | -0.22 |
| CDK14 | ENST00000380050.3 | cyclin-dependent kinase 14 | 226 | 1 | 1 | 0 | 0 | 1 | bta-miR-2430 | -0.22 |
| SF3B4 | ENST00000271628.8 | splicing factor 3b, subunit 4, 49kDa | 8 | 1 | 0 | 0 | 1 | 0 | bta-miR-2430 | -0.22 |
| GATSL3 | ENST00000407689.3 | GATS protein-like 3 | 20 | 1 | 0 | 1 | 0 | 0 | bta-miR-2430 | -0.22 |
| CALHM1 | ENST00000329905.5 | calcium homeostasis modulator 1 | 5 | 1 | 0 | 1 | 0 | 0 | bta-miR-2430 | -0.22 |
| TRPM1 | ENST00000397795.2 | transient receptor potential cation channel, subfamily M, member 1 | 5 | 1 | 1 | 0 | 0 | 0 | bta-miR-2430 | -0.22 |
| LRRFIP1 | ENST00000308482.9 | leucine rich repeat (in FLII) interacting protein 1 | 525 | 1 | 0 | 1 | 0 | 0 | bta-miR-2430 | -0.22 |
| CHAD | ENST00000258969.4 | chondroadherin | 5 | 1 | 0 | 1 | 0 | 1 | bta-miR-2430 | -0.22 |
| LYRM9 | ENST00000460380.2 | LYR motif containing 9 | 191 | 1 | 0 | 1 | 0 | 1 | bta-miR-2430 | -0.22 |
| NANOS3 | ENST00000397555.2 | nanos homolog 3 (Drosophila) | 5 | 1 | 0 | 1 | 0 | 0 | bta-miR-2430 | -0.22 |
| C4orf19 | ENST00000381980.4 | chromosome 4 open reading frame 19 | 51 | 1 | 0 | 0 | 1 | 3 | bta-miR-2430 | -0.22 |
| FBXL20 | ENST00000394294.3 | F-box and leucine-rich repeat protein 20 | 455 | 2 | 0 | 1 | 1 | 3 | bta-miR-2430 | -0.22 |
| SAPCD2 | ENST00000409687.3 | suppressor APC domain containing 2 | 22 | 2 | 0 | 2 | 0 | 0 | bta-miR-2430 | -0.22 |
| CHST13 | ENST00000319340.2 | carbohydrate (chondroitin 4) sulfotransferase 13 | 1271 | 1 | 0 | 1 | 0 | 0 | bta-miR-2430 | -0.22 |
| DPP8 | ENST00000341861.5 | dipeptidyl-peptidase 8 | 1293 | 1 | 0 | 1 | 0 | 1 | bta-miR-2430 | -0.22 |
| PAFAH2 | ENST00000374282.3 | platelet-activating factor acetylhydrolase 2, 40kDa | 287 | 1 | 0 | 1 | 0 | 1 | bta-miR-2430 | -0.22 |
| SLC16A2 | ENST00000587091.1 | solute carrier family 16, member 2 (thyroid hormone transporter) | 22 | 1 | 1 | 0 | 0 | 0 | bta-miR-2430 | -0.22 |
| NUP214 | ENST00000359428.5 | nucleoporin 214kDa | 452 | 2 | 0 | 2 | 0 | 0 | bta-miR-2430 | -0.22 |
| PRUNE | ENST00000271620.3 | prune exopolyphosphatase | 41 | 1 | 0 | 1 | 0 | 0 | bta-miR-2430 | -0.22 |
| SEMA4A | ENST00000368282.1 | sema domain, immunoglobulin domain (Ig), transmembrane domain (TM) and short cytoplasmic domain, (semaphorin) 4A | 30 | 1 | 1 | 0 | 0 | 0 | bta-miR-2430 | -0.22 |
| TAF8 | ENST00000372977.3 | TAF8 RNA polymerase II, TATA box binding protein (TBP)-associated factor, 43kDa | 135 | 1 | 0 | 0 | 1 | 1 | bta-miR-2430 | -0.22 |
| SIRT1 | ENST00000212015.6 | sirtuin 1 | 220 | 1 | 1 | 0 | 0 | 0 | bta-miR-2430 | -0.22 |
| PGLYRP4 | ENST00000359650.5 | peptidoglycan recognition protein 4 | 5 | 1 | 0 | 1 | 0 | 1 | bta-miR-2430 | -0.22 |
| SAA4 | ENST00000278222.4 | serum amyloid A4, constitutive | 5 | 1 | 0 | 0 | 1 | 0 | bta-miR-2430 | -0.22 |
| FXYD7 | ENST00000270310.2 | FXYD domain containing ion transport regulator 7 | 5 | 1 | 0 | 1 | 0 | 0 | bta-miR-2430 | -0.22 |
| ALDH1A2 | ENST00000249750.4 | aldehyde dehydrogenase 1 family, member A2 | 401 | 1 | 0 | 1 | 0 | 0 | bta-miR-2430 | -0.22 |
| MAN1A2 | ENST00000356554.3 | mannosidase, alpha, class 1A, member 2 | 603 | 1 | 1 | 0 | 0 | 2 | bta-miR-2430 | -0.22 |
| NAT10 | ENST00000257829.3 | N-acetyltransferase 10 (GCN5-related) | 132 | 2 | 1 | 0 | 1 | 0 | bta-miR-2430 | -0.22 |
| RBM48 | ENST00000481551.1 | RNA binding motif protein 48 | 220 | 1 | 0 | 1 | 0 | 0 | bta-miR-2430 | -0.22 |
| PPP1R14C | ENST00000361131.4 | protein phosphatase 1, regulatory (inhibitor) subunit 14C | 173 | 1 | 0 | 1 | 0 | 0 | bta-miR-2430 | -0.22 |
| ELOVL1 | ENST00000372458.3 | ELOVL fatty acid elongase 1 | 188 | 1 | 0 | 1 | 0 | 0 | bta-miR-2430 | -0.22 |
| ACVRL1 | ENST00000550683.1 | activin A receptor type II-like 1 | 68 | 3 | 2 | 0 | 1 | 1 | bta-miR-2430 | -0.22 |
| FAM160B2 | ENST00000289921.7 | family with sequence similarity 160, member B2 | 803 | 2 | 1 | 1 | 0 | 0 | bta-miR-2430 | -0.22 |
| ALDH1A3 | ENST00000329841.5 | aldehyde dehydrogenase 1 family, member A3 | 8 | 1 | 0 | 1 | 0 | 0 | bta-miR-2430 | -0.22 |
| MAPK7 | ENST00000299612.7 | mitogen-activated protein kinase 7 | 617 | 1 | 0 | 1 | 0 | 0 | bta-miR-2430 | -0.22 |
| IMPG1 | ENST00000369963.3 | interphotoreceptor matrix proteoglycan 1 | 5 | 1 | 0 | 1 | 0 | 2 | bta-miR-2430 | -0.22 |
| C1orf110 | ENST00000367912.2 | chromosome 1 open reading frame 110 | 5 | 1 | 0 | 1 | 0 | 0 | bta-miR-2430 | -0.22 |
| ITK | ENST00000422843.3 | IL2-inducible T-cell kinase | 5 | 1 | 0 | 1 | 0 | 0 | bta-miR-2430 | -0.22 |
| TMUB2 | ENST00000590235.1 | transmembrane and ubiquitin-like domain containing 2 | 1043 | 1 | 0 | 1 | 0 | 0 | bta-miR-2430 | -0.22 |
| HELLS | ENST00000394036.1 | helicase, lymphoid-specific | 329 | 1 | 0 | 1 | 0 | 0 | bta-miR-2430 | -0.22 |
| UST | ENST00000367463.4 | uronyl-2-sulfotransferase | 933 | 2 | 0 | 0 | 2 | 1 | bta-miR-2430 | -0.22 |
| IQGAP1 | ENST00000268182.5 | IQ motif containing GTPase activating protein 1 | 5523 | 2 | 0 | 1 | 1 | 0 | bta-miR-2430 | -0.22 |
| NCKAP5L | ENST00000335999.6 | NCK-associated protein 5-like | 141 | 2 | 0 | 1 | 1 | 0 | bta-miR-2430 | -0.22 |
| ATP6V0E1 | ENST00000519374.1 | ATPase, H+ transporting, lysosomal 9kDa, V0 subunit e1 | 1195 | 1 | 0 | 0 | 1 | 0 | bta-miR-2430 | -0.22 |
| LIMD2 | ENST00000259006.3 | LIM domain containing 2 | 90 | 1 | 0 | 1 | 0 | 1 | bta-miR-2430 | -0.22 |
| AP1S1 | ENST00000337619.5 | adaptor-related protein complex 1, sigma 1 subunit | 750 | 1 | 0 | 1 | 0 | 0 | bta-miR-2430 | -0.22 |
| GPR17 | ENST00000272644.3 | G protein-coupled receptor 17 | 5 | 1 | 0 | 0 | 1 | 1 | bta-miR-2430 | -0.22 |
| XXbac-BPG32J3.20 | ENST00000461287.1 | | 1251 | 1 | 0 | 0 | 1 | 1 | bta-miR-2430 | -0.22 |
| ANAPC13 | ENST00000510994.1 | anaphase promoting complex subunit 13 | 443 | 1 | 0 | 0 | 1 | 2 | bta-miR-2430 | -0.22 |
| ADAMTS13 | ENST00000371916.1 | ADAM metallopeptidase with thrombospondin type 1 motif, 13 | 41 | 1 | 0 | 1 | 0 | 2 | bta-miR-2430 | -0.21 |
| TSPYL4 | ENST00000420283.1 | TSPY-like 4 | 5 | 1 | 1 | 0 | 0 | 0 | bta-miR-2430 | -0.21 |
| NRP2 | ENST00000360409.3 | neuropilin 2 | 146 | 1 | 1 | 0 | 0 | 3 | bta-miR-2430 | -0.21 |
| PKP3 | ENST00000331563.2 | plakophilin 3 | 167 | 1 | 0 | 1 | 0 | 0 | bta-miR-2430 | -0.21 |
| CSH2 | ENST00000336844.5 | chorionic somatomammotropin hormone 2 | 5 | 1 | 0 | 1 | 0 | 0 | bta-miR-2430 | -0.21 |
| ALPP | ENST00000392027.2 | alkaline phosphatase, placental | 5 | 1 | 0 | 0 | 1 | 2 | bta-miR-2430 | -0.21 |
| TCP11X1 | ENST00000333110.5 | t-complex 11 family, X-linked 1 | 5 | 1 | 0 | 1 | 0 | 0 | bta-miR-2430 | -0.21 |
| SMKR1 | ENST00000462322.2 | small lysine-rich protein 1 | 243 | 1 | 0 | 0 | 1 | 0 | bta-miR-2430 | -0.21 |
| MAN1C1 | ENST00000374332.4 | mannosidase, alpha, class 1C, member 1 | 60 | 2 | 1 | 1 | 0 | 2 | bta-miR-2430 | -0.21 |
| ARHGAP32 | ENST00000310343.9 | Rho GTPase activating protein 32 | 16 | 1 | 0 | 0 | 1 | 1 | bta-miR-2430 | -0.21 |
| SSR3 | ENST00000476217.1 | signal sequence receptor, gamma (translocon-associated protein gamma) | 6034 | 1 | 1 | 0 | 0 | 2 | bta-miR-2430 | -0.21 |
| PIP5K1A | ENST00000368890.4 | phosphatidylinositol-4-phosphate 5-kinase, type I, alpha | 3762 | 1 | 0 | 1 | 0 | 0 | bta-miR-2430 | -0.21 |
| MSRB1 | ENST00000399753.2 | methionine sulfoxide reductase B1 | 2102 | 1 | 0 | 0 | 1 | 1 | bta-miR-2430 | -0.21 |
| CDH5 | ENST00000341529.3 | cadherin 5, type 2 (vascular endothelium) | 8 | 1 | 1 | 0 | 0 | 0 | bta-miR-2430 | -0.21 |
| KIRREL3 | ENST00000525144.2 | kin of IRRE like 3 (Drosophila) | 16 | 1 | 1 | 0 | 0 | 1 | bta-miR-2430 | -0.21 |
| PIEZO2 | ENST00000302079.6 | piezo-type mechanosensitive ion channel component 2 | 158 | 1 | 1 | 0 | 0 | 0 | bta-miR-2430 | -0.21 |
| EHD2 | ENST00000263277.3 | EH-domain containing 2 | 2072 | 2 | 0 | 2 | 0 | 2 | bta-miR-2430 | -0.21 |
| TPCN2 | ENST00000294309.3 | two pore segment channel 2 | 629 | 2 | 1 | 0 | 1 | 1 | bta-miR-2430 | -0.21 |
| PDCD7 | ENST00000204549.4 | programmed cell death 7 | 64 | 1 | 0 | 1 | 0 | 0 | bta-miR-2430 | -0.21 |
| PTPRT | ENST00000373187.1 | protein tyrosine phosphatase, receptor type, T | 5 | 2 | 1 | 0 | 1 | 2 | bta-miR-2430 | -0.21 |
| WNT3 | ENST00000225512.5 | wingless-type MMTV integration site family, member 3 | 46 | 1 | 1 | 0 | 0 | 0 | bta-miR-2430 | -0.21 |
| TTC26 | ENST00000430935.1 | tetratricopeptide repeat domain 26 | 211 | 1 | 1 | 0 | 0 | 0 | bta-miR-2430 | -0.21 |
| NMNAT2 | ENST00000294868.4 | nicotinamide nucleotide adenylyltransferase 2 | 18 | 3 | 0 | 1 | 2 | 0 | bta-miR-2430 | -0.21 |
| LEMD1 | ENST00000367154.1 | LEM domain containing 1 | 5 | 1 | 0 | 1 | 0 | 0 | bta-miR-2430 | -0.21 |
| TMEM86A | ENST00000280734.2 | transmembrane protein 86A | 5 | 1 | 1 | 0 | 0 | 0 | bta-miR-2430 | -0.21 |
| ZNF583 | ENST00000333201.9 | zinc finger protein 583 | 24 | 1 | 0 | 1 | 0 | 0 | bta-miR-2430 | -0.21 |
| GSTO2 | ENST00000369707.2 | glutathione S-transferase omega 2 | 97 | 2 | 0 | 0 | 2 | 0 | bta-miR-2430 | -0.21 |
| MOCOS | ENST00000261326.5 | molybdenum cofactor sulfurase | 627 | 3 | 0 | 1 | 2 | 0 | bta-miR-2430 | -0.21 |
| GJB5 | ENST00000338513.1 | gap junction protein, beta 5, 31.1kDa | 5 | 1 | 0 | 1 | 0 | 0 | bta-miR-2430 | -0.21 |
| CLEC5A | ENST00000546910.1 | C-type lectin domain family 5, member A | 5 | 1 | 0 | 1 | 0 | 2 | bta-miR-2430 | -0.21 |
| KRT40 | ENST00000377755.4 | keratin 40 | 5 | 1 | 0 | 0 | 1 | 1 | bta-miR-2430 | -0.21 |
| KIAA0100 | ENST00000528896.2 | KIAA0100 | 12 | 1 | 0 | 1 | 0 | 0 | bta-miR-2430 | -0.21 |
| AC021218.2 | ENST00000377722.2 | Uncharacterized protein | 10 | 1 | 0 | 1 | 0 | 0 | bta-miR-2430 | -0.21 |
| ASB12 | ENST00000362002.2 | ankyrin repeat and SOCS box containing 12 | 5 | 1 | 0 | 1 | 0 | 0 | bta-miR-2430 | -0.21 |
| ZGLP1 | ENST00000403903.3 | zinc finger, GATA-like protein 1 | 7 | 1 | 0 | 1 | 0 | 0 | bta-miR-2430 | -0.21 |
| TMEM106A | ENST00000331615.3 | transmembrane protein 106A | 22 | 1 | 0 | 1 | 0 | 1 | bta-miR-2430 | -0.21 |
| BMP8A | ENST00000331593.5 | bone morphogenetic protein 8a | 13 | 1 | 0 | 1 | 0 | 3 | bta-miR-2430 | -0.21 |
| TMEM91 | ENST00000539627.1 | transmembrane protein 91 | 231 | 1 | 0 | 0 | 1 | 1 | bta-miR-2430 | -0.21 |
| SMO | ENST00000249373.3 | smoothened, frizzled family receptor | 142 | 1 | 1 | 0 | 0 | 0 | bta-miR-2430 | -0.21 |
| SPSB4 | ENST00000310546.2 | splA/ryanodine receptor domain and SOCS box containing 4 | 419 | 1 | 0 | 1 | 0 | 0 | bta-miR-2430 | -0.21 |
| THNSL2 | ENST00000343544.4 | threonine synthase-like 2 (S. cerevisiae) | 219 | 1 | 0 | 1 | 0 | 0 | bta-miR-2430 | -0.21 |
| MED1 | ENST00000300651.6 | mediator complex subunit 1 | 87 | 2 | 0 | 2 | 0 | 0 | bta-miR-2430 | -0.21 |
| NPAS2 | ENST00000335681.5 | neuronal PAS domain protein 2 | 559 | 2 | 0 | 1 | 1 | 0 | bta-miR-2430 | -0.21 |
| CDIP1 | ENST00000564828.1 | cell death-inducing p53 target 1 | 1857 | 1 | 0 | 1 | 0 | 1 | bta-miR-2430 | -0.21 |
| WDR33 | ENST00000322313.4 | WD repeat domain 33 | 537 | 2 | 1 | 0 | 1 | 0 | bta-miR-2430 | -0.21 |
| SLC2A4 | ENST00000317370.8 | solute carrier family 2 (facilitated glucose transporter), member 4 | 233 | 1 | 1 | 0 | 0 | 0 | bta-miR-2430 | -0.21 |
| MYRIP | ENST00000302541.6 | myosin VIIA and Rab interacting protein | 9 | 1 | 1 | 0 | 0 | 0 | bta-miR-2430 | -0.21 |
| POLR1D | ENST00000399697.3 | polymerase (RNA) I polypeptide D, 16kDa | 2372 | 1 | 0 | 0 | 1 | 1 | bta-miR-2430 | -0.21 |
| SLC25A53 | ENST00000357421.4 | solute carrier family 25, member 53 | 15 | 1 | 0 | 1 | 0 | 0 | bta-miR-2430 | -0.21 |
| SNTA1 | ENST00000217381.2 | syntrophin, alpha 1 | 360 | 1 | 0 | 1 | 0 | 0 | bta-miR-2430 | -0.21 |
| F13A1 | ENST00000264870.3 | coagulation factor XIII, A1 polypeptide | 5 | 2 | 0 | 2 | 0 | 0 | bta-miR-2430 | -0.21 |
| ADIG | ENST00000373348.3 | adipogenin | 5 | 1 | 0 | 0 | 1 | 0 | bta-miR-2430 | -0.21 |
| FBXL12 | ENST00000589626.1 | F-box and leucine-rich repeat protein 12 | 27 | 1 | 0 | 1 | 0 | 1 | bta-miR-2430 | -0.21 |
| ZDHHC20 | ENST00000320220.9 | zinc finger, DHHC-type containing 20 | 1486 | 1 | 0 | 1 | 0 | 0 | bta-miR-2430 | -0.21 |

**Predicted target mRNAs of mir-365**

| Ortholog of target gene | Representative transcript | Gene name | 3P-seq tags + 5 | Conserved sites total | Conserved 8mer sites | Conserved 7mer-m8 sites | Conserved 7mer-A1 sites | Poorly conserved sites total | Poorly conserved 8mer sites | Poorly conserved 7mer-m8 sites | Poorly conserved 7mer-A1 sites | 6mer sites | Representative miRNA | Cumulative weighted context++ score |
| --- | --- | --- | --- | --- | --- | --- | --- | --- | --- | --- | --- | --- | --- | --- |
| Best3 | ENSMUST00000020378.4 | bestrophin 3 | 174 | 1 | 1 | 0 | 0 | 0 | 0 | 0 | 0 | 0 | bta-miR-365-3p | -1.13 |
| Pde4d | ENSMUST00000120664.2 | phosphodiesterase 4D, cAMP specific | 1752 | 1 | 0 | 1 | 0 | 2 | 0 | 1 | 1 | 0 | bta-miR-365-3p | -1.1 |
| Hs3st1 | ENSMUST00000117944.1 | heparan sulfate (glucosamine) 3-O-sulfotransferase 1 | 137 | 2 | 0 | 2 | 0 | 1 | 0 | 1 | 0 | 0 | bta-miR-365-3p | -1.04 |
| Trp63 | ENSMUST00000115308.3 | transformation related protein 63 | 73 | 1 | 1 | 0 | 0 | 0 | 0 | 0 | 0 | 0 | bta-miR-365-3p | -1.03 |
| Rqcd1 | ENSMUST00000087215.6 | rcd1 (required for cell differentiation) homolog 1 (S. pombe) | 558 | 1 | 1 | 0 | 0 | 0 | 0 | 0 | 0 | 0 | bta-miR-365-3p | -0.97 |
| Traf3 | ENSMUST00000117269.2 | TNF receptor-associated factor 3 | 465 | 1 | 1 | 0 | 0 | 1 | 0 | 0 | 1 | 0 | bta-miR-365-3p | -0.95 |
| Entpd7 | ENSMUST00000081079.5 | ectonucleoside triphosphate diphosphohydrolase 7 | 146 | 1 | 1 | 0 | 0 | 1 | 0 | 0 | 1 | 0 | bta-miR-365-3p | -0.91 |
| Lmtk2 | ENSMUST00000041804.7 | lemur tyrosine kinase 2 | 931 | 1 | 1 | 0 | 0 | 0 | 0 | 0 | 0 | 0 | bta-miR-365-3p | -0.87 |
| Acvr1 | ENSMUST00000112599.2 | activin A receptor, type 1 | 687 | 2 | 1 | 1 | 0 | 0 | 0 | 0 | 0 | 1 | bta-miR-365-3p | -0.86 |
| Pate2 | ENSMUST00000118254.1 | prostate and testis expressed 2 | 10 | 1 | 1 | 0 | 0 | 0 | 0 | 0 | 0 | 0 | bta-miR-365-3p | -0.85 |
| Ahsa2 | ENSMUST00000109539.2 | AHA1, activator of heat shock protein ATPase 2 | 1274 | 1 | 1 | 0 | 0 | 0 | 0 | 0 | 0 | 0 | bta-miR-365-3p | -0.79 |
| Prpf40a | ENSMUST00000076313.7 | PRP40 pre-mRNA processing factor 40 homolog A (yeast) | 3927 | 1 | 1 | 0 | 0 | 2 | 0 | 1 | 1 | 1 | bta-miR-365-3p | -0.77 |
| Snrk | ENSMUST00000120173.2 | SNF related kinase | 6 | 1 | 0 | 1 | 0 | 0 | 0 | 0 | 0 | 1 | bta-miR-365-3p | -0.75 |
| Srgap1 | ENSMUST00000020322.6 | SLIT-ROBO Rho GTPase activating protein 1 | 109 | 1 | 0 | 1 | 0 | 0 | 0 | 0 | 0 | 0 | bta-miR-365-3p | -0.66 |
| Ormdl1 | ENSMUST00000027266.3 | ORM1-like 1 (S. cerevisiae) | 773 | 1 | 1 | 0 | 0 | 0 | 0 | 0 | 0 | 0 | bta-miR-365-3p | -0.65 |
| Usp48 | ENSMUST00000105840.2 | ubiquitin specific peptidase 48 | 3448 | 1 | 1 | 0 | 0 | 0 | 0 | 0 | 0 | 0 | bta-miR-365-3p | -0.64 |
| 9930021J03Rik | ENSMUST00000177155.2 | RIKEN cDNA 9930021J03 gene | 221 | 1 | 0 | 1 | 0 | 0 | 0 | 0 | 0 | 2 | bta-miR-365-3p | -0.64 |
| Arrb2 | ENSMUST00000102564.5 | arrestin, beta 2 | 705 | 1 | 1 | 0 | 0 | 0 | 0 | 0 | 0 | 0 | bta-miR-365-3p | -0.63 |
| Meis1 | ENSMUST00000068264.8 | Meis homeobox 1 | 223 | 1* | 0 | 0 | 0 | 0 | 0 | 0 | 0 | 0 | bta-miR-365-3p | -0.62 |
| Tfdp1 | ENSMUST00000170909.1 | transcription factor Dp 1 | 6372 | 1 | 0 | 1 | 0 | 0 | 0 | 0 | 0 | 0 | bta-miR-365-3p | -0.61 |
| Sgk1 | ENSMUST00000020145.6 | serum/glucocorticoid regulated kinase 1 | 5205 | 1 | 1 | 0 | 0 | 0 | 0 | 0 | 0 | 0 | bta-miR-365-3p | -0.61 |
| Syngr2 | ENSMUST00000177131.2 | synaptogyrin 2 | 1400 | 1 | 0 | 1 | 0 | 0 | 0 | 0 | 0 | 0 | bta-miR-365-3p | -0.59 |
| Trpm3 | ENSMUST00000037901.7 | transient receptor potential cation channel, subfamily M, member 3 | 21 | 1 | 0 | 1 | 0 | 0 | 0 | 0 | 0 | 1 | bta-miR-365-3p | -0.58 |
| Efemp1 | ENSMUST00000020759.6 | epidermal growth factor-containing fibulin-like extracellular matrix protein 1 | 345 | 1 | 0 | 1 | 0 | 0 | 0 | 0 | 0 | 1 | bta-miR-365-3p | -0.57 |
| Tada2a | ENSMUST00000018795.7 | transcriptional adaptor 2A | 71 | 1 | 0 | 1 | 0 | 1 | 0 | 1 | 0 | 0 | bta-miR-365-3p | -0.57 |
| Samd5 | ENSMUST00000100070.2 | sterile alpha motif domain containing 5 | 102 | 1 | 1 | 0 | 0 | 2 | 0 | 2 | 0 | 1 | bta-miR-365-3p | -0.56 |
| Zfp644 | ENSMUST00000112696.2 | zinc finger protein 644 | 601 | 1 | 1 | 0 | 0 | 0 | 0 | 0 | 0 | 0 | bta-miR-365-3p | -0.56 |
| Kcnj2 | ENSMUST00000042970.2 | potassium inwardly-rectifying channel, subfamily J, member 2 | 89 | 2 | 2 | 0 | 0 | 0 | 0 | 0 | 0 | 0 | bta-miR-365-3p | -0.55 |
| Rapgef4 | ENSMUST00000090826.6 | Rap guanine nucleotide exchange factor (GEF) 4 | 1334 | 1 | 1 | 0 | 0 | 0 | 0 | 0 | 0 | 0 | bta-miR-365-3p | -0.54 |
| Chfr | ENSMUST00000112519.3 | checkpoint with forkhead and ring finger domains | 1174 | 1 | 0 | 1 | 0 | 0 | 0 | 0 | 0 | 0 | bta-miR-365-3p | -0.54 |
| E2f2 | ENSMUST00000061721.5 | E2F transcription factor 2 | 59 | 1 | 1 | 0 | 0 | 0 | 0 | 0 | 0 | 0 | bta-miR-365-3p | -0.54 |
| Usp33 | ENSMUST00000026507.7 | ubiquitin specific peptidase 33 | 711 | 1 | 1 | 0 | 0 | 0 | 0 | 0 | 0 | 0 | bta-miR-365-3p | -0.54 |
| Qtrtd1 | ENSMUST00000023387.8 | queuine tRNA-ribosyltransferase domain containing 1 | 39 | 1 | 1 | 0 | 0 | 0 | 0 | 0 | 0 | 0 | bta-miR-365-3p | -0.53 |
| Avpr1a | ENSMUST00000020323.5 | arginine vasopressin receptor 1A | 286 | 1 | 1 | 0 | 0 | 0 | 0 | 0 | 0 | 1 | bta-miR-365-3p | -0.53 |
| Chp1 | ENSMUST00000119172.1 | calcineurin-like EF hand protein 1 | 4235 | 1 | 1 | 0 | 0 | 1 | 0 | 0 | 1 | 0 | bta-miR-365-3p | -0.51 |
| Dynll1 | ENSMUST00000009157.3 | dynein light chain LC8-type 1 | 81 | 1 | 0 | 1 | 0 | 0 | 0 | 0 | 0 | 0 | bta-miR-365-3p | -0.49 |
| Hhip | ENSMUST00000079038.2 | Hedgehog-interacting protein | 27 | 1 | 0 | 1 | 0 | 0 | 0 | 0 | 0 | 2 | bta-miR-365-3p | -0.49 |
| Adm | ENSMUST00000033054.8 | adrenomedullin | 265 | 1 | 0 | 1 | 0 | 0 | 0 | 0 | 0 | 0 | bta-miR-365-3p | -0.48 |
| Btg2 | ENSMUST00000020692.6 | B cell translocation gene 2, anti-proliferative | 3163 | 1 | 0 | 1 | 0 | 1 | 0 | 1 | 0 | 0 | bta-miR-365-3p | -0.48 |
| Esrra | ENSMUST00000025906.5 | estrogen related receptor, alpha | 1259 | 1 | 1 | 0 | 0 | 0 | 0 | 0 | 0 | 0 | bta-miR-365-3p | -0.48 |
| S100a14 | ENSMUST00000167598.3 | S100 calcium binding protein A14 | 18 | 1 | 0 | 1 | 0 | 0 | 0 | 0 | 0 | 0 | bta-miR-365-3p | -0.48 |
| Lct | ENSMUST00000073490.6 | lactase | 5 | 1 | 1 | 0 | 0 | 0 | 0 | 0 | 0 | 0 | bta-miR-365-3p | -0.47 |
| Sgk3 | ENSMUST00000168907.2 | serum/glucocorticoid regulated kinase 3 | 254 | 1 | 0 | 1 | 0 | 0 | 0 | 0 | 0 | 0 | bta-miR-365-3p | -0.47 |
| Cbfb | ENSMUST00000052209.3 | core binding factor beta | 6247 | 1 | 0 | 0 | 1 | 2 | 0 | 0 | 2 | 1 | bta-miR-365-3p | -0.47 |
| Oxr1 | ENSMUST00000022918.8 | oxidation resistance 1 | 554 | 1 | 1 | 0 | 0 | 0 | 0 | 0 | 0 | 0 | bta-miR-365-3p | -0.47 |
| Pax6 | ENSMUST00000111086.5 | paired box 6 | 15 | 1 | 0 | 1 | 0 | 0 | 0 | 0 | 0 | 0 | bta-miR-365-3p | -0.45 |
| Ehf | ENSMUST00000090475.4 | ets homologous factor | 151 | 1 | 1 | 0 | 0 | 0 | 0 | 0 | 0 | 0 | bta-miR-365-3p | -0.45 |
| Pias1 | ENSMUST00000098651.4 | protein inhibitor of activated STAT 1 | 811 | 2 | 1 | 0 | 1 | 0 | 0 | 0 | 0 | 0 | bta-miR-365-3p | -0.44 |
| Eya3 | ENSMUST00000081726.7 | eyes absent 3 homolog (Drosophila) | 473 | 1 | 0 | 1 | 0 | 0 | 0 | 0 | 0 | 1 | bta-miR-365-3p | -0.44 |
| Nr3c2 | ENSMUST00000109913.3 | nuclear receptor subfamily 3, group C, member 2 | 405 | 1 | 1 | 0 | 0 | 0 | 0 | 0 | 0 | 0 | bta-miR-365-3p | -0.44 |
| Rnf182 | ENSMUST00000059986.2 | ring finger protein 182 | 8 | 1 | 1 | 0 | 0 | 0 | 0 | 0 | 0 | 1 | bta-miR-365-3p | -0.44 |
| Arpc4 | ENSMUST00000156898.1 | actin related protein 2/3 complex, subunit 4 | 2340 | 1 | 1 | 0 | 0 | 0 | 0 | 0 | 0 | 0 | bta-miR-365-3p | -0.43 |
| Lysmd3 | ENSMUST00000049055.6 | LysM, putative peptidoglycan-binding, domain containing 3 | 719 | 1 | 1 | 0 | 0 | 0 | 0 | 0 | 0 | 0 | bta-miR-365-3p | -0.43 |
| Dlat | ENSMUST00000034567.3 | dihydrolipoamide S-acetyltransferase (E2 component of pyruvate dehydrogenase complex) | 7557 | 1 | 1 | 0 | 0 | 0 | 0 | 0 | 0 | 0 | bta-miR-365-3p | -0.43 |
| Tbk1 | ENSMUST00000020316.2 | TANK-binding kinase 1 | 111 | 2 | 0 | 0 | 2 | 0 | 0 | 0 | 0 | 0 | bta-miR-365-3p | -0.43 |
| Adcy6 | ENSMUST00000096224.4 | adenylate cyclase 6 | 43 | 1 | 0 | 1 | 0 | 1 | 1 | 0 | 0 | 1 | bta-miR-365-3p | -0.43 |
| Dock4 | ENSMUST00000037488.6 | dedicator of cytokinesis 4 | 596 | 1 | 1 | 0 | 0 | 0 | 0 | 0 | 0 | 0 | bta-miR-365-3p | -0.43 |
| D15Ertd621e | ENSMUST00000037270.3 | DNA segment, Chr 15, ERATO Doi 621, expressed | 2211 | 1 | 1 | 0 | 0 | 1 | 0 | 0 | 1 | 0 | bta-miR-365-3p | -0.42 |
| Igf1 | ENSMUST00000105300.3 | insulin-like growth factor 1 | 5701 | 1 | 0 | 1 | 0 | 0 | 0 | 0 | 0 | 2 | bta-miR-365-3p | -0.4 |
| Sypl | ENSMUST00000076698.6 | synaptophysin-like protein | 1404 | 1 | 1 | 0 | 0 | 0 | 0 | 0 | 0 | 0 | bta-miR-365-3p | -0.4 |
| P2ry1 | ENSMUST00000029331.1 | purinergic receptor P2Y, G-protein coupled 1 | 160 | 1 | 1 | 0 | 0 | 0 | 0 | 0 | 0 | 1 | bta-miR-365-3p | -0.4 |
| Klf3 | ENSMUST00000165536.2 | Kruppel-like factor 3 (basic) | 4159 | 1 | 1 | 0 | 0 | 0 | 0 | 0 | 0 | 0 | bta-miR-365-3p | -0.39 |
| Abhd12b | ENSMUST00000182927.2 | abhydrolase domain containing 12B | 7 | 1 | 1 | 0 | 0 | 0 | 0 | 0 | 0 | 0 | bta-miR-365-3p | -0.39 |
| Hmgcr | ENSMUST00000022176.9 | 3-hydroxy-3-methylglutaryl-Coenzyme A reductase | 1378 | 1 | 0 | 1 | 0 | 0 | 0 | 0 | 0 | 0 | bta-miR-365-3p | -0.39 |
| Dnajb2 | ENSMUST00000188931.1 | DnaJ (Hsp40) homolog, subfamily B, member 2 | 2295 | 1 | 1 | 0 | 0 | 0 | 0 | 0 | 0 | 0 | bta-miR-365-3p | -0.39 |
| Trhde | ENSMUST00000061632.7 | TRH-degrading enzyme | 58 | 1 | 1 | 0 | 0 | 0 | 0 | 0 | 0 | 3 | bta-miR-365-3p | -0.39 |
| Ammecr1 | ENSMUST00000041317.2 | Alport syndrome, mental retardation, midface hypoplasia and elliptocytosis chromosomal region gene 1 | 88 | 1 | 0 | 1 | 0 | 0 | 0 | 0 | 0 | 2 | bta-miR-365-3p | -0.38 |
| Tmod3 | ENSMUST00000072232.7 | tropomodulin 3 | 1070 | 1 | 1 | 0 | 0 | 0 | 0 | 0 | 0 | 0 | bta-miR-365-3p | -0.38 |
| Rictor | ENSMUST00000061656.6 | RPTOR independent companion of MTOR, complex 2 | 898 | 1 | 1 | 0 | 0 | 1 | 0 | 1 | 0 | 0 | bta-miR-365-3p | -0.38 |
| Rasd1 | ENSMUST00000062405.7 | RAS, dexamethasone-induced 1 | 26 | 1 | 1 | 0 | 0 | 0 | 0 | 0 | 0 | 0 | bta-miR-365-3p | -0.38 |
| Afap1l2 | ENSMUST00000122359.2 | actin filament associated protein 1-like 2 | 11 | 1 | 0 | 1 | 0 | 1 | 0 | 0 | 1 | 0 | bta-miR-365-3p | -0.38 |
| Ankrd11 | ENSMUST00000098334.6 | ankyrin repeat domain 11 | 15 | 1 | 0 | 1 | 0 | 0 | 0 | 0 | 0 | 0 | bta-miR-365-3p | -0.38 |
| Mylk | ENSMUST00000023538.8 | myosin, light polypeptide kinase | 4565 | 1 | 1 | 0 | 0 | 0 | 0 | 0 | 0 | 0 | bta-miR-365-3p | -0.37 |
| Fndc3b | ENSMUST00000046157.4 | fibronectin type III domain containing 3B | 367 | 1 | 0 | 1 | 0 | 0 | 0 | 0 | 0 | 2 | bta-miR-365-3p | -0.37 |
| Add3 | ENSMUST00000111741.3 | adducin 3 (gamma) | 2312 | 1 | 1 | 0 | 0 | 0 | 0 | 0 | 0 | 0 | bta-miR-365-3p | -0.36 |
| Nabp1 | ENSMUST00000027279.6 | nucleic acid binding protein 1 | 1026 | 1 | 0 | 1 | 0 | 0 | 0 | 0 | 0 | 0 | bta-miR-365-3p | -0.36 |
| Set | ENSMUST00000102866.4 | SET nuclear oncogene | 1369 | 1 | 0 | 1 | 0 | 0 | 0 | 0 | 0 | 0 | bta-miR-365-3p | -0.35 |
| A830080D01Rik | ENSMUST00000057180.7 | RIKEN cDNA A830080D01 gene | 420 | 1 | 1 | 0 | 0 | 0 | 0 | 0 | 0 | 0 | bta-miR-365-3p | -0.34 |
| Eif4e3 | ENSMUST00000032151.2 | eukaryotic translation initiation factor 4E member 3 | 317 | 1 | 0 | 1 | 0 | 0 | 0 | 0 | 0 | 1 | bta-miR-365-3p | -0.34 |
| Uhrf1bp1l | ENSMUST00000020112.5 | UHRF1 (ICBP90) binding protein 1-like | 1527 | 1 | 1 | 0 | 0 | 0 | 0 | 0 | 0 | 0 | bta-miR-365-3p | -0.34 |
| Creb5 | ENSMUST00000047450.7 | cAMP responsive element binding protein 5 | 82 | 1 | 0 | 1 | 0 | 0 | 0 | 0 | 0 | 0 | bta-miR-365-3p | -0.34 |
| Pgk1 | ENSMUST00000081593.7 | phosphoglycerate kinase 1 | 11 | 1 | 0 | 0 | 1 | 0 | 0 | 0 | 0 | 0 | bta-miR-365-3p | -0.33 |
| Nfib | ENSMUST00000107245.3 | nuclear factor I/B | 2888 | 2 | 0 | 1 | 1 | 0 | 0 | 0 | 0 | 2 | bta-miR-365-3p | -0.33 |
| Hoxa9 | ENSMUST00000114425.2 | homeobox A9 | 245 | 1 | 1 | 0 | 0 | 0 | 0 | 0 | 0 | 0 | bta-miR-365-3p | -0.33 |
| Plag1 | ENSMUST00000003369.4 | pleiomorphic adenoma gene 1 | 77 | 1 | 0 | 0 | 1 | 0 | 0 | 0 | 0 | 0 | bta-miR-365-3p | -0.33 |
| Dlx3 | ENSMUST00000092768.6 | distal-less homeobox 3 | 5 | 1 | 0 | 1 | 0 | 0 | 0 | 0 | 0 | 0 | bta-miR-365-3p | -0.33 |
| Mex3a | ENSMUST00000172699.1 | mex3 homolog A (C. elegans) | 442 | 1 | 1 | 0 | 0 | 0 | 0 | 0 | 0 | 1 | bta-miR-365-3p | -0.33 |
| Tram1 | ENSMUST00000027068.5 | translocating chain-associating membrane protein 1 | 2880 | 1 | 0 | 1 | 0 | 0 | 0 | 0 | 0 | 0 | bta-miR-365-3p | -0.32 |
| Pde4d | ENSMUST00000099179.2 | phosphodiesterase 4D, cAMP specific | 1608 | 1 | 0 | 1 | 0 | 0 | 0 | 0 | 0 | 0 | bta-miR-365-3p | -0.32 |
| Pip5k1a | ENSMUST00000107236.3 | phosphatidylinositol-4-phosphate 5-kinase, type 1 alpha | 1241 | 1 | 1 | 0 | 0 | 0 | 0 | 0 | 0 | 0 | bta-miR-365-3p | -0.32 |
| Nwd2 | ENSMUST00000159584.1 | NACHT and WD repeat domain containing 2 | 8 | 1 | 1 | 0 | 0 | 0 | 0 | 0 | 0 | 0 | bta-miR-365-3p | -0.32 |
| Ago3 | ENSMUST00000069097.7 | argonaute RISC catalytic subunit 3 | 292 | 2 | 1 | 1 | 0 | 0 | 0 | 0 | 0 | 2 | bta-miR-365-3p | -0.31 |
| Ank3 | ENSMUST00000182884.2 | ankyrin 3, epithelial | 1269 | 1 | 0 | 1 | 0 | 0 | 0 | 0 | 0 | 0 | bta-miR-365-3p | -0.31 |
| Rp2h | ENSMUST00000033372.7 | retinitis pigmentosa 2 homolog (human) | 416 | 1 | 1 | 0 | 0 | 0 | 0 | 0 | 0 | 0 | bta-miR-365-3p | -0.31 |
| Ago2 | ENSMUST00000044113.10 | argonaute RISC catalytic subunit 2 | 1138 | 1 | 0 | 1 | 0 | 2 | 0 | 1 | 1 | 2 | bta-miR-365-3p | -0.31 |
| Adam10 | ENSMUST00000067880.7 | a disintegrin and metallopeptidase domain 10 | 1662 | 1 | 1 | 0 | 0 | 0 | 0 | 0 | 0 | 0 | bta-miR-365-3p | -0.31 |
| Fam60a | ENSMUST00000054080.9 | family with sequence similarity 60, member A | 26 | 1 | 0 | 1 | 0 | 0 | 0 | 0 | 0 | 0 | bta-miR-365-3p | -0.31 |
| Vgll4 | ENSMUST00000032459.8 | vestigial like 4 (Drosophila) | 1729 | 1 | 0 | 1 | 0 | 0 | 0 | 0 | 0 | 0 | bta-miR-365-3p | -0.3 |
| Rgs9bp | ENSMUST00000069912.5 | regulator of G-protein signalling 9 binding protein | 25 | 1 | 1 | 0 | 0 | 1 | 0 | 0 | 1 | 0 | bta-miR-365-3p | -0.3 |
| Timp3 | ENSMUST00000020234.8 | tissue inhibitor of metalloproteinase 3 | 10779 | 1 | 0 | 1 | 0 | 0 | 0 | 0 | 0 | 0 | bta-miR-365-3p | -0.3 |
| Nlrp1a | ENSMUST00000048514.5 | NLR family, pyrin domain containing 1A | 0 | 1 | 1 | 0 | 0 | 0 | 0 | 0 | 0 | 0 | bta-miR-365-3p | -0.29 |
| Tasp1 | ENSMUST00000110079.3 | taspase, threonine aspartase 1 | 98 | 1 | 0 | 1 | 0 | 0 | 0 | 0 | 0 | 0 | bta-miR-365-3p | -0.29 |
| Phf12 | ENSMUST00000049167.8 | PHD finger protein 12 | 598 | 1 | 0 | 1 | 0 | 0 | 0 | 0 | 0 | 0 | bta-miR-365-3p | -0.29 |
| Slco5a1 | ENSMUST00000188454.1 | solute carrier organic anion transporter family, member 5A1 | 20 | 1 | 0 | 1 | 0 | 0 | 0 | 0 | 0 | 1 | bta-miR-365-3p | -0.29 |
| Kcnj3 | ENSMUST00000112632.1 | potassium inwardly-rectifying channel, subfamily J, member 3 | 181 | 2 | 0 | 0 | 2 | 0 | 0 | 0 | 0 | 2 | bta-miR-365-3p | -0.29 |
| Rnf44 | ENSMUST00000177950.2 | ring finger protein 44 | 2723 | 1 | 0 | 1 | 0 | 0 | 0 | 0 | 0 | 0 | bta-miR-365-3p | -0.29 |
| Hs3st3a1 | ENSMUST00000058652.5 | heparan sulfate (glucosamine) 3-O-sulfotransferase 3A1 | 12 | 1 | 1 | 0 | 0 | 0 | 0 | 0 | 0 | 0 | bta-miR-365-3p | -0.29 |
| Mapk1ip1l | ENSMUST00000164235.1 | mitogen-activated protein kinase 1 interacting protein 1-like | 1272 | 1 | 0 | 1 | 0 | 0 | 0 | 0 | 0 | 1 | bta-miR-365-3p | -0.29 |
| Gdf11 | ENSMUST00000026408.6 | growth differentiation factor 11 | 123 | 1 | 0 | 1 | 0 | 0 | 0 | 0 | 0 | 0 | bta-miR-365-3p | -0.29 |
| Arhgap12 | ENSMUST00000182066.2 | Rho GTPase activating protein 12 | 645 | 1 | 0 | 1 | 0 | 0 | 0 | 0 | 0 | 0 | bta-miR-365-3p | -0.29 |
| 8030462N17Rik | ENSMUST00000074653.4 | RIKEN cDNA 8030462N17 gene | 353 | 1 | 0 | 1 | 0 | 1 | 0 | 0 | 1 | 2 | bta-miR-365-3p | -0.29 |
| Impad1 | ENSMUST00000084949.2 | inositol monophosphatase domain containing 1 | 3917 | 1 | 0 | 1 | 0 | 1 | 0 | 0 | 1 | 0 | bta-miR-365-3p | -0.28 |
| Fli1 | ENSMUST00000016231.8 | Friend leukemia integration 1 | 141 | 1 | 0 | 1 | 0 | 0 | 0 | 0 | 0 | 0 | bta-miR-365-3p | -0.28 |
| Med12l | ENSMUST00000040325.8 | mediator complex subunit 12-like | 64 | 1 | 0 | 1 | 0 | 0 | 0 | 0 | 0 | 0 | bta-miR-365-3p | -0.28 |
| Tcp11l2 | ENSMUST00000020223.7 | t-complex 11 (mouse) like 2 | 1349 | 2 | 0 | 1 | 1 | 0 | 0 | 0 | 0 | 0 | bta-miR-365-3p | -0.28 |
| Ugcg | ENSMUST00000030074.7 | UDP-glucose ceramide glucosyltransferase | 811 | 1 | 0 | 1 | 0 | 0 | 0 | 0 | 0 | 0 | bta-miR-365-3p | -0.28 |
| Nr4a2 | ENSMUST00000028166.3 | nuclear receptor subfamily 4, group A, member 2 | 59 | 1 | 0 | 1 | 0 | 0 | 0 | 0 | 0 | 0 | bta-miR-365-3p | -0.28 |
| Rac1 | ENSMUST00000080537.8 | RAS-related C3 botulinum substrate 1 | 454 | 1 | 0 | 1 | 0 | 0 | 0 | 0 | 0 | 0 | bta-miR-365-3p | -0.28 |
| Socs5 | ENSMUST00000041369.6 | suppressor of cytokine signaling 5 | 2081 | 1 | 0 | 1 | 0 | 1 | 0 | 0 | 1 | 0 | bta-miR-365-3p | -0.27 |
| Ing3 | ENSMUST00000031680.4 | inhibitor of growth family, member 3 | 348 | 1 | 0 | 1 | 0 | 0 | 0 | 0 | 0 | 0 | bta-miR-365-3p | -0.27 |
| Synj1 | ENSMUST00000170853.2 | synaptojanin 1 | 641 | 1 | 0 | 1 | 0 | 0 | 0 | 0 | 0 | 0 | bta-miR-365-3p | -0.27 |
| Psmd8 | ENSMUST00000182328.2 | proteasome (prosome, macropain) 26S subunit, non-ATPase, 8 | 1799 | 1 | 1 | 0 | 0 | 0 | 0 | 0 | 0 | 0 | bta-miR-365-3p | -0.27 |
| Plce1 | ENSMUST00000169713.3 | phospholipase C, epsilon 1 | 216 | 1 | 0 | 1 | 0 | 0 | 0 | 0 | 0 | 0 | bta-miR-365-3p | -0.27 |
| Plcb4 | ENSMUST00000110109.2 | phospholipase C, beta 4 | 266 | 1 | 0 | 1 | 0 | 0 | 0 | 0 | 0 | 0 | bta-miR-365-3p | -0.27 |
| Bach1 | ENSMUST00000026703.5 | BTB and CNC homology 1 | 993 | 1 | 0 | 1 | 0 | 0 | 0 | 0 | 0 | 0 | bta-miR-365-3p | -0.27 |
| Mecp2 | ENSMUST00000100750.4 | methyl CpG binding protein 2 | 845 | 1 | 1 | 0 | 0 | 3 | 1 | 0 | 2 | 0 | bta-miR-365-3p | -0.26 |
| Slc30a7 | ENSMUST00000067485.3 | solute carrier family 30 (zinc transporter), member 7 | 257 | 1 | 1 | 0 | 0 | 1 | 0 | 0 | 1 | 2 | bta-miR-365-3p | -0.26 |
| Gxylt1 | ENSMUST00000057896.4 | glucoside xylosyltransferase 1 | 1508 | 1 | 0 | 1 | 0 | 0 | 0 | 0 | 0 | 1 | bta-miR-365-3p | -0.26 |
| Pik3r3 | ENSMUST00000030464.8 | phosphatidylinositol 3 kinase, regulatory subunit, polypeptide 3 (p55) | 310 | 1 | 0 | 1 | 0 | 0 | 0 | 0 | 0 | 1 | bta-miR-365-3p | -0.26 |
| Atp6v1g1 | ENSMUST00000035301.6 | ATPase, H+ transporting, lysosomal V1 subunit G1 | 94 | 1 | 0 | 0 | 1 | 0 | 0 | 0 | 0 | 0 | bta-miR-365-3p | -0.26 |
| Cnot6l | ENSMUST00000155901.2 | CCR4-NOT transcription complex, subunit 6-like | 632 | 2 | 0 | 2 | 0 | 1 | 0 | 0 | 1 | 1 | bta-miR-365-3p | -0.26 |
| Adamts1 | ENSMUST00000023610.9 | a disintegrin-like and metallopeptidase (reprolysin type) with thrombospondin type 1 motif, 1 | 597 | 1 | 1 | 0 | 0 | 0 | 0 | 0 | 0 | 0 | bta-miR-365-3p | -0.26 |
| Pgp | ENSMUST00000053024.6 | phosphoglycolate phosphatase | 1903 | 1 | 0 | 1 | 0 | 0 | 0 | 0 | 0 | 0 | bta-miR-365-3p | -0.26 |
| Uprt | ENSMUST00000087867.5 | uracil phosphoribosyltransferase (FUR1) homolog (S. cerevisiae) | 58 | 1 | 0 | 1 | 0 | 0 | 0 | 0 | 0 | 0 | bta-miR-365-3p | -0.25 |
| Fubp3 | ENSMUST00000055244.7 | far upstream element (FUSE) binding protein 3 | 672 | 1 | 0 | 1 | 0 | 0 | 0 | 0 | 0 | 0 | bta-miR-365-3p | -0.25 |
| Zfp148 | ENSMUST00000165418.2 | zinc finger protein 148 | 580 | 1 | 0 | 1 | 0 | 0 | 0 | 0 | 0 | 2 | bta-miR-365-3p | -0.25 |
| Etv1 | ENSMUST00000095767.4 | ets variant 1 | 907 | 1 | 0 | 1 | 0 | 0 | 0 | 0 | 0 | 0 | bta-miR-365-3p | -0.25 |
| Grpel2 | ENSMUST00000062991.7 | GrpE-like 2, mitochondrial | 87 | 1 | 0 | 1 | 0 | 0 | 0 | 0 | 0 | 0 | bta-miR-365-3p | -0.25 |
| Rnf144b | ENSMUST00000068891.5 | ring finger protein 144B | 828 | 1 | 0 | 1 | 0 | 0 | 0 | 0 | 0 | 0 | bta-miR-365-3p | -0.25 |
| Inpp4b | ENSMUST00000172031.2 | inositol polyphosphate-4-phosphatase, type II | 281 | 1 | 1 | 0 | 0 | 0 | 0 | 0 | 0 | 1 | bta-miR-365-3p | -0.25 |
| Ppp1r9a | ENSMUST00000035813.3 | protein phosphatase 1, regulatory (inhibitor) subunit 9A | 208 | 1 | 0 | 1 | 0 | 0 | 0 | 0 | 0 | 2 | bta-miR-365-3p | -0.24 |
| Epas1 | ENSMUST00000024954.9 | endothelial PAS domain protein 1 | 55091 | 1 | 0 | 1 | 0 | 0 | 0 | 0 | 0 | 0 | bta-miR-365-3p | -0.24 |
| Tpm4 | ENSMUST00000003575.9 | tropomyosin 4 | 2694 | 1 | 0 | 1 | 0 | 0 | 0 | 0 | 0 | 0 | bta-miR-365-3p | -0.24 |
| Dyrk2 | ENSMUST00000004281.8 | dual-specificity tyrosine-(Y)-phosphorylation regulated kinase 2 | 1322 | 1 | 0 | 1 | 0 | 0 | 0 | 0 | 0 | 1 | bta-miR-365-3p | -0.24 |
| Ubp1 | ENSMUST00000084885.6 | upstream binding protein 1 | 1113 | 1 | 0 | 1 | 0 | 0 | 0 | 0 | 0 | 0 | bta-miR-365-3p | -0.24 |
| Gm9979 | ENSMUST00000069457.1 | predicted gene 9979 | 5 | 1 | 0 | 0 | 1 | 0 | 0 | 0 | 0 | 1 | bta-miR-365-3p | -0.24 |
| Satb2 | ENSMUST00000042857.8 | special AT-rich sequence binding protein 2 | 205 | 1 | 0 | 1 | 0 | 1 | 0 | 1 | 0 | 0 | bta-miR-365-3p | -0.24 |
| Lin28b | ENSMUST00000079390.6 | lin-28 homolog B (C. elegans) | 191 | 1 | 0 | 1 | 0 | 0 | 0 | 0 | 0 | 1 | bta-miR-365-3p | -0.23 |
| Lin7c | ENSMUST00000028583.7 | lin-7 homolog C (C. elegans) | 1842 | 1 | 0 | 1 | 0 | 0 | 0 | 0 | 0 | 1 | bta-miR-365-3p | -0.23 |
| Six4 | ENSMUST00000043208.7 | sine oculis-related homeobox 4 | 266 | 1 | 0 | 1 | 0 | 0 | 0 | 0 | 0 | 1 | bta-miR-365-3p | -0.23 |
| Ppp2cb | ENSMUST00000009774.9 | protein phosphatase 2 (formerly 2A), catalytic subunit, beta isoform | 2724 | 1 | 0 | 0 | 1 | 0 | 0 | 0 | 0 | 0 | bta-miR-365-3p | -0.22 |
| Pcsk5 | ENSMUST00000050715.8 | proprotein convertase subtilisin/kexin type 5 | 74 | 1 | 1 | 0 | 0 | 0 | 0 | 0 | 0 | 0 | bta-miR-365-3p | -0.22 |
| Wnt5a | ENSMUST00000063465.5 | wingless-related MMTV integration site 5A | 231 | 1 | 1 | 0 | 0 | 0 | 0 | 0 | 0 | 0 | bta-miR-365-3p | -0.22 |
| Sema3a | ENSMUST00000030714.7 | sema domain, immunoglobulin domain (Ig), short basic domain, secreted, (semaphorin) 3A | 440 | 1 | 0 | 0 | 1 | 0 | 0 | 0 | 0 | 1 | bta-miR-365-3p | -0.22 |
| Lpar1 | ENSMUST00000055018.5 | lysophosphatidic acid receptor 1 | 1273 | 1 | 0 | 0 | 1 | 0 | 0 | 0 | 0 | 0 | bta-miR-365-3p | -0.22 |
| Sertad2 | ENSMUST00000093292.5 | SERTA domain containing 2 | 635 | 1 | 0 | 0 | 1 | 0 | 0 | 0 | 0 | 1 | bta-miR-365-3p | -0.21 |
| Ankrd28 | ENSMUST00000014640.7 | ankyrin repeat domain 28 | 256 | 1 | 0 | 1 | 0 | 0 | 0 | 0 | 0 | 2 | bta-miR-365-3p | -0.21 |
| Pdgfra | ENSMUST00000000476.9 | platelet derived growth factor receptor, alpha polypeptide | 1153 | 1 | 0 | 1 | 0 | 0 | 0 | 0 | 0 | 1 | bta-miR-365-3p | -0.21 |
| 4930402H24Rik | ENSMUST00000044766.9 | RIKEN cDNA 4930402H24 gene | 2877 | 1 | 0 | 1 | 0 | 1 | 0 | 1 | 0 | 1 | bta-miR-365-3p | -0.21 |
| Hdac4 | ENSMUST00000097644.3 | histone deacetylase 4 | 652 | 1 | 0 | 1 | 0 | 0 | 0 | 0 | 0 | 0 | bta-miR-365-3p | -0.21 |
| Tram2 | ENSMUST00000037998.5 | translocating chain-associating membrane protein 2 | 195 | 1 | 1 | 0 | 0 | 1 | 0 | 0 | 1 | 1 | bta-miR-365-3p | -0.21 |
| Abl2 | ENSMUST00000166172.3 | v-abl Abelson murine leukemia viral oncogene 2 (arg, Abelson-related gene) | 860 | 1 | 1 | 0 | 0 | 1 | 0 | 1 | 0 | 1 | bta-miR-365-3p | -0.21 |
