# Supplementary material for: Analysis of hepatic transcript profile and plasma lipid profile in early lactating dairy cows fed grape seed and grape marc meal extract
Source: BMC Genomics. 2017 Mar 23;18:253. doi: 10.1186/s12864-017-3638-1 (PMC5364584; doi:10.1186/s12864-017-3638-1)
Supplement: Supplementary file 3 — Concentrations of various lipid species in plasma (μM) of cows fed grape seed and grape marc meal extract (GSGME) and control cows at 1 week postpartum. (DOCX 45 kb) [file 12864_2017_3638_MOESM3_ESM.docx]

**Table S3 Sphingomyelin (SM) species in plasma (µM) of cows fed grape seed and grape marc meal extract (GSGME) and control cows at 1 week postpartum**

| Species | Control | GSGME |
| --- | --- | --- |
| SM 24:1 | n.d. | n.d. |
| SM 30:1 | 0.12 ± 0.13 | 0.12 ± 0.08 |
| SM 32:1 | 4.42 ± 1.47 | 4.56 ± 1.73 |
| SM 33:1 | 5.63 ± 1.79 | 6.01 ± 1.82 |
| SM 34:2 | 7.59 ± 2.70 | 7.94 ± 2.19 |
| SM 34:1 | 64.63 ± 14.94 | 64.35 ± 16.97 |
| SM 34:0 | 7.27 ± 1.93 | 6.36 ± 1.87 |
| SM 35:3 | 0.19 ± 0.21 | 0.18 ± 0.17 |
| SM 35:2 | 0.56 ± 0.47 | 0.55 ± 0.34 |
| SM 35:1 | 5.95 ± 1.65 | 5.80 ± 1.48 |
| SM 35:0 | 0.91 ± 0.32 | 0.76 ± 0.27 |
| SM 36:3 | 0.48 ± 0.32 | 0.66 ± 0.31 |
| SM 36:2 | 3.80 ± 0.81 | 3.73 ± 1.16 |
| SM 36:1 | 7.81 ± 1.37 | 7.62 ± 1.91 |
| SM 36:0 | 1.05 ± 0.51 | 1.03 ± 0.72 |
| SM 38:4 | 0.96 ± 0.46 | 1.03 ± 0.53 |
| SM 38:3 | 0.10 ± 0.14 | 0.13 ± 0.17 |
| SM 38:2 | 0.75 ± 0.73 | 0.53 ± 0.37 |
| SM 38:1 | 2.65 ± 1.37 | 1.34 ± 0.98 |
| SM 38:0 | 2.56 ± 1.67 | 1.81 ± 1.22 |
| SM 40:5 | 0.86 ± 0.43 | 0.99 ± 0.39 |
| SM 40:4 | 0.30 ± 0.34 | 0.11 ± 0.24 |
| SM 40:3 | 0.38 ± 0.65 | 0.79 ± 0.79 |
| SM 40:2 | 4.70 ± 2.07 | 5.21 ± 2.00 |
| SM 40:1 | 9.44 ± 2.36 | 9.91 ± 2.77 |
| SM 40:0 | 1.98 ± 1.61 | 2.85 ± 1.59 |
| SM 41:2 | 7.01± 1.61 | 7.07 ± 1.90 |
| SM 41:1 | 11.44 ± 2.03 | 12.07 ± 3.66 |
| SM 41:0 | 0.64 ± 0.33 | 0.58 ± 0.26 |
| SM 42:4 | 0.39 ± 0.50 | 0.59 ± 0.55 |
| SM 42:3 | 3.77 ± 1.31 | 3.98 ± 1.44 |
| SM 42:2 | 14.12 ± 2.61 | 13.58 ± 4.10 |
| SM 42:1 | 8.43 ± 1.56 | 8.66 ± 2.78 |
| SM 42:0 | 0.50 ± 0.31 | 0.56 ± 0.31 |
| SM 44:5 | 0.20 ± 0.35 | 0.07 ± 0.16 |
| SM 44:4 | 0.28 ± 0.41 | 0.22 ± 0.27 |
| SM 44:3 | 0.34 ± 0.33 | 0.46 ± 0.30 |
| SM 44:2 | 0.47 ± 0.38 | 0.35 ± 0.19 |
| SM 44:1 | 0.28 ± 0.19 | 0.25 ± 0.12 |
| SM 44:0 | 0.05 ± 0.07 | 0.06 ± 0.10 |
| SM 45:4 | n.d. | 0.05 ± 0.11 |
| SM 46:6 | 0.01 ± 0.04 | 0.04 ± 0.06 |
| SM 46:5 | 0.01 ± 0.04 | 0.01 ± 0.03 |
| SM 46:4 | 0.01 ± 0.04 | 0.01 ± 0.04 |
| SM 46:3 | n.d. ± n.d. | 0.02 ± 0.06 |
| ∑ SFA | 115.76 ± 27.54 | 115.65 ± 33.09 |
| ∑ MUFA | 44.39 ± 12.55 | 44.20 ± 13.40 |
| ∑ PUFA | 8.84 ± 6.05 | 9.89 ± 5.98 |
| ∑unsaturated | 53.23 ± 18.60 | 54.09± 19.38 |
| total | 168.99 ± 46.14 | 169.74 ± 52.47 |
| dihydro | 14.05 ± 6.43 | 13.25 ± 6.08 |

n.d., not detected; SFA, saturated fatty acids; MUFA, monounsaturated fatty acids; PUFA, polyunsaturated fatty acids.

**Table S4 Phosphatidylcholine (PC) species (µM) of cows fed grape seed and grape marc meal extract (GSGME) and control cows at 1 week postpartum**

| Species | Control | GSGME |
| --- | --- | --- |
| PC 26:0 | 1.23 ± 0.28 | 1.32 ± 0.33 |
| PC O-28:1 | 0.12 ± 0.12 | 0.10 ± 0.14 |
| PC O-28:0 | 0.16 ± 0.15 | 0.17 ± 0.12 |
| PC O-30:2 | 0.30 ± 0.19 | 0.35 ± 0.15 |
| PC O-30:1 | 1.17 ± 0.50 | 1.51 ± 0.71 |
| PC O-30:0 | 1.22 ± 0.34 | 1.17 ± 0.35 |
| PC 30:1 | 3.58 ± 1.75 | 4.62 ± 2.15 |
| PC 30:0 | 2.44 ± 1.07 | 2.61 ± 1.31 |
| PC O-32:3 | 2.23 ± 1.13 | 2.58 ± 1.36 |
| PC O-32:2 | 2.84 ± 1.17 | 3.05 ± 1.26 |
| PC O-32:1 | 2.19 ± 0.67 | 2.25 ± 0.65 |
| PC O-32:0 | 2.02 ± 0.75 | 2.16 ± 0.67 |
| PC 32:3 | 6.95 ± 3.45 | 7.69 ± 3.77 |
| PC 32:2 | 6.30 ± 3.25 | 6.61 ± 2.63 |
| PC 32:1 | 9.29 ± 3.42 | 8.89 ± 2.61 |
| PC 32:0 | 6.33 ± 1.54 | 5.68 ± 1.21 |
| PC O-34:3 | 8.30 ± 2.96 | 8.72 ± 3.60 |
| PC O-34:2 | 11.09 ± 3.72 | 10.98 ± 3.14 |
| PC O-34:1 | 10.54 ± 3.34 | 10.69 ± 2.66 |
| PC O-34:0 | 1.77 ± 0.88 | 1.78 ± 0.60 |
| PC 34:4 | 2.02 ± 1.12 | 1.98 ± 0.98 |
| PC 34:3 | 19.06 ± 6.30 | 19.86 ± 6.24 |
| PC 34:2 | 161.01 ± 43.95 | 159.98 ± 49.19 |
| PC 34:1 | 145.37 ± 47.77 | 145.83 ± 42.28 |
| PC 34:0 | 4.95 ± 0.99 | 5.29 ± 2.17 |
| PC O-36:5 | 3.68 ± 1.01 | 3.63 ± 1.19 |
| PC O-36:4 | 3.84 ± 1.35 | 3.74 ± 1.32 |
| PC O-36:3 | 5.02 ± 1.39 | 5.05 ± 1.80 |
| PC O-36:2 | 12.15 ± 3.51 | 11.97 ± 3.61 |
| PC O-36:1 | 11.31 ± 4.17 | 12.39 ± 3.52 |
| PC O-36:0 | 1.56 ± 0.62 | 1.53 ± 0.83 |
| PC 36:5 | 6.33 ± 1.97 | 6.97 ± 3.05 |
| PC 36:4 | 24.60 ± 6.04 | 24.55 ± 7.16 |
| PC 36:3 | 65.82 ± 2n.d. | 64.78 ± 20.62 |
| PC 36:2 | 178.89 ± 49.29 | 178.91 ± 62.25 |
| PC 36:1 | 116.31 ± 42.11 | 120.86 ± 41.39 |
| PC 36:0 | 6.21 ± 2.68 | 6.00 ± 1.69 |
| PC O-38:5 | 3.16 ± 0.89 | 3.19 ± 1.01 |
| PC O-38:4 | 2.58 ± 0.82 | 2.49 ± 0.78 |
| PC O-38:3 | 2.34 ± 0.86 | 2.15 ± 0.66 |
| PC O-38:2 | 1.88 ± 0.87 | 1.62 ± 0.68 |
| PC O-38:1 | 1.64 ± 0.76 | 1.56 ± 0.86 |
| PC 38:7 | 1.28 ± 0.60 | 1.49 ± 0.49 |
| PC 38:6 | 5.51 ± 2.52 | 5.69 ± 2.61 |
| PC 38:5 | 26.62 ± 8.65 | 26.94 ± 10.57 |
| PC 38:4 | 37.92 ± 13.28 | 36.28 ± 12.93 |
| PC 38:3 | 31.93 ± 17.28 | 27.86 ± 12.68 |
| PC 38:2 | 8.42 ± 4.03 | 8.97 ± 3.25 |
| PC 38:1 | 4.33 ± 2.25 | 4.20 ± 1.70 |
| PC 38:0 | 1.43 ± 0.63 | 1.56 ± 0.64 |
| PC O-40:6 | 0.99 ± 0.26 | 1.02 ± 0.37 |
| PC O-40:5 | 1.19 ± 0.52 | 1.29 ± 0.51 |
| PC O-40:4 | 0.57 ± 0.29 | 0.51 ± 0.22 |
| PC 40:7 | 0.55 ± 0.23 | 0.51 ± 0.26 |
| PC 40:6 | 2.82 ± 1.81 | 2.76 ± 0.97 |
| PC 40:5 | 8.06 ± 3.11 | 8.58 ± 2.45 |
| PC 40:4 | 2.08 ± 1.07 | 2.17 ± 0.81 |
| PC 40:3 | 0.42 ± 0.26 | 0.45 ± 0.25 |
| PC 40:2 | 0.13 ± 0.12 | 0.19 ± 0.12 |
| PC 40:1 | 0.07 ± 0.08 | 0.08 ± 0.10 |
| PC 40:0 | 0.10 ± 0.11 | 0.06 ± 0.08 |
| PC O-42:6 | 0.12 ± 0.13 | 0.14 ± 0.10 |
| PC O-42:5 | 0.13 ± 0.10 | 0.14 ± 0.11 |
| PC O-42:4 | 0.09 ± 0.12 | 0.07 ± 0.10 |
| PC 42:6 | 0.05 ± 0.07 | 0.05 ± 0.05 |
| PC 42:5 | 0.06 ± 0.08 | 0.09 ± 0.07 |
| PC 42:4 | n.d. | 0.02 ± 0.06 |
| PC 42:0 | 0.02 ± 0.03 | 0.05 ± 0.07 |
| PC O-44:6 | 0.01 ± 0.03 | n.d. ± n.d. |
| PC O-44:5 | 0.01 ± 0.04 | n.d. ± 0.01 |
| PC O-44:4 | n.d. ± n.d. | n.d. ± n.d. |
| PC 44:1 | n.d. ±n.d. | 0.01 ± 0.05 |
| PC O-46:6 | 1.06 ± 0.40 | 0.64 ± 0.49 |
| ∑ SFA | 22.76 ± 5.80 | 22.62 ± 5.96 |
| ∑ MUFA | 278.95 ± 91.84 | 284.50 ± 87.00 |
| ∑ PUFA | 597.87 ± 163.58 | 593.98 ± 180.98 |
| ∑ unsaturated | 876.82 ± 251.73 | 878.48 ± 260.77 |
| ∑ alkyl | 96.22 ± 30.31 | 98.01 ± 28.40 |
| total | 995.80 ± 284.92 | 999.11 ± 292.80 |

n.d., not detected; SFA, saturated fatty acids; MUFA, monounsaturated fatty acids; PUFA, polyunsaturated fatty acids.

**Table S5 Phosphatidylethanolamine (PE) species in plasma (µM) of cows fed grape seed and grape marc meal extract (GSGME) and control cows at 1 week postpartum**

| Species, µM | Control | GSGME |
| --- | --- | --- |
| PE 24:0 | n.d. | n.d. |
| PE 26:0 | n.d. | n.d. |
| PE O-30:0 | n.d. | n.d. |
| PE 30:3 | n.d. | n.d. |
| PE 30:2 | n.d. | n.d. |
| PE 30:1 | n.d. | n.d. |
| PE 30:0 | n.d. | n.d. |
| PE O-32:2 | n.d. | n.d. |
| PE O-32:1 | n.d. | n.d. |
| PE O-32:0 | n.d. | n.d. |
| PE 32:3 | n.d. | n.d. |
| PE 32:2 | 0.18 ± 0.07 | 0.22 ± 0.08 |
| PE 32:1 | 0.11± 0.02 | 0.13 ± 0.03 |
| PE 32:0 | 0.09 ± 0.03 | 0.08 ± 0.02 |
| PE O-34:3 | n.d. | n.d. |
| PE O-34:2 | n.d. | n.d. |
| PE O-34:1 | n.d. | n.d. |
| PE O-34:0 | n.d. | n.d. |
| PE 34:4 | n.d. | n.d. |
| PE 34:3 | 0.29 ± 0.12 | 0.31 ± 0.09 |
| PE 34:2 | 1.30 ± 0.63 | 1.32 ± 0.57 |
| PE 34:1 | 0.58 ± 0.22 | 0.57 ± 0.15 |
| PE 34:0 | 0.10 ± 0.05 | 0.08 ± 0.06 |
| PE O-36:5 | n.d. | n.d. |
| PE O-36:4 | n.d. | n.d. |
| PE O-36:3 | n.d. | n.d. |
| PE O-36:2 | n.d. | n.d. |
| PE O-36:1 | n.d. | n.d. |
| PE 36:6 | n.d. | n.d. |
| PE 36:5 | 0.08 ± 0.03 | 0.09 ± 0.03 |
| PE 36:4 | 0.19 ± 0.08 | 0.19 ± 0.06 |
| PE 36:3 | 0.29 ± 0.11 | 0.29 ± 0.09 |
| PE 36:2 | 0.90 ± 0.34 | 0.87 ± 0.30 |
| PE 36:1 | 0.22 ± 0.06 | 0.24 ± 0.07 |
| PE O-38:7 | 0.05 ± 0.02 | 0.05 ± 0.03 |
| PE O-38:6 | n.d. | n.d. |
| PE O-38:5 | n.d. | n.d. |
| PE O-38:4 | n.d. | n.d. |
| PE O-38:3 | n.d. | n.d. |
| PE 38:7 | n.d. | n.d. |
| PE 38:6 | 0.13 ± 0.05 | 0.12 ± 0.04 |
| PE 38:5 | 0.44 ± 0.17 | 0.42 ± 0.18 |
| PE 38:4 | 0.72 ± 0.25 | 0.68 ± 0.17 |
| PE 38:3 | 0.14 ± 0.06 | 0.13 ± 0.05 |
| PE 38:2 | 0.07 ± 0.02 | 0.06 ± 0.02 |
| PE 38:1 | 0.08 ± 0.02 | 0.09 ± 0.03 |
| PE O-40:7 | n.d. | n.d. |
| PE O-40:6 | n.d. | n.d. |
| PE O-40:5 | n.d. | n.d. |
| PE O-40:4 | n.d. | n.d. |
| PE 40:7 | 0.16 ± 0.02 | 0.16 ± 0.02 |
| PE 40:6 | 0.04 ± 0.02 | 0.04 ± 0.01 |
| PE 40:5 | 0.07 ± 0.03 | 0.08 ± 0.02 |
| PE 40:4 | 0.03 ± 0.01 | 0.03 ± 0.01 |
| PE 40:3 | 0.01 ± 0.01 | 0.02 ± n.d. |
| PE 40:2 | n.d. | n.d. |
| PE 40:1 | n.d. | n.d. |
| PE 40:0 | 5.27 ± 0.37 | 5.23 ± 0.33 |
| PE 42:7 | 0.03 ± 0.01 | 0.03 ± n.d. |
| PE 42:6 | 0.02 ± 0.01 | 0.02 ± 0.01 |
| PE 42:5 | 0.02 ± 0.01 | 0.02 ± 0.01 |
| ∑ SFA | 0.19 ± 0.07 | 0.17 ± 0.07 |
| ∑ MUFA | 0.99 ± 0.30 | 1.02 ± 0.25 |
| ∑ PUFA | 4.97 ± 1.66 | 4.95 ± 1.40 |
| ∑ unsaturated | 5.96 ± 1.92 | 5.97 ± 1.62 |
| ∑ alkyl | 0.22 ± 0.03 | 0.21 ± 0.03 |
| total | 6.36 ± 1.96 | 6.36 ± 1.64 |

n.d., not detected; SFA, saturated fatty acids; MUFA, monounsaturated fatty acids; PUFA, polyunsaturated fatty acids.

**Table S6 Phosphatidylethanolamine (PE) plasmalogen (P) species in plasma (µM) of cows fed grape seed and grape marc meal extract (GSGME) and control cows at 1 week postpartum**

| Species | Control | GSGME |
| --- | --- | --- |
| PE P-16:0/16:1 | 0.34 ± 0.04 | 0.31 ± 0.04 |
| PE P-16:0/16:0 | 0.31 ± 0.03 | 0.30 ± 0.06 |
| PE P-16:0/18:3 | 0.46 ± 0.07 | 0.48 ± 0.15 |
| PE P-16:0/18:2 | 1.57 ± 0.64 | 1.79 ± 0.83 |
| PE P-16:0/18:1 | 0.67 ± 0.23 | 0.78 ± 0.26 |
| PE P-16:0/18:0 | 0.30 ± 0.02 | 0.29 ± 0.04 |
| PE P-16:0/20:5 | 0.40 ± 0.05 | 0.42 ± 0.06 |
| PE P-16:0/20:4 | 0.68 ± 0.12 | 0.68 ± 0.18 |
| PE P-16:0/20:3 | 0.48 ± 0.20 | 0.43 ± 0.11 |
| PE P-16:0/22:6 | 0.39 ± 0.07 | 0.40 ± 0.12 |
| PE P-16:0/22:5 | 0.51 ± 0.13 | 0.51 ± 0.16 |
| PE P-16:0/22:4 | 0.32 ± 0.06 | 0.32 ± 0.06 |
| PE P-16:0/22:3 | 0.30 ± 0.04 | 0.27 ± 0.04 |
| PE P-18:1/16:1 | 0.30 ± 0.03 | 0.29 ± 0.05 |
| PE P-18:1/16:0 | 0.30 ± 0.02 | 0.30 ± 0.04 |
| PE P-18:1/18:3 | 0.33 ± 0.04 | 0.33 ± 0.07 |
| PE P-18:1/18:2 | 0.55 ± 0.18 | 0.61 ± 0.23 |
| PE P-18:1/18:1 | 0.47 ± 0.13 | 0.49 ± 0.10 |
| PE P-18:1/18:0 | 0.29 ± 0.03 | 0.26 ± 0.04 |
| PE P-18:1/20:5 | 0.33 ± 0.02 | 0.31± 0.05 |
| PE P-18:1/20:4 | 0.40 ± 0.06 | 0.39 ± 0.08 |
| PE P-18:1/20:3 | 0.32 ± 0.05 | 0.31 ± 0.05 |
| PE P-18:1/22:6 | 0.30 ± 0.03 | 0.29 ± 0.05 |
| PE P-18:1/22:5 | 0.30 ± 0.04 | 0.29 ± 0.05 |
| PE P-18:1/22:4 | 0.31 ± 0.03 | 0.27 ± 0.03 |
| PE P-18:1/22:3 | 0.28 ± 0.04 | 0.26 ± 0.04 |
| PE P-18:0/16:1 | 0.30 ± 0.03 | 0.28 ± 0.04 |
| PE P-18:0/16:0 | 0.29 ± 0.03 | 0.26 ± 0.03 |
| PE P-18:0/18:3 | 0.31 ± 0.04 | 0.30 ± 0.06 |
| PE P-18:0/18:2 | 0.48 ± 0.10 | 0.51 ± 0.16 |
| PE P-18:0/18:1 | 0.40 ± 0.09 | 0.39 ± 0.09 |
| PE P-18:0/18:0 | 0.28 ± 0.04 | 0.25 ± 0.03 |
| PE P-18:0/20:5 | 0.32 ± 0.03 | 0.29 ± 0.05 |
| PE P-18:0/20:4 | 0.44 ± 0.08 | 0.41 ± 0.09 |
| PE P-18:0/20:3 | 0.31 ± 0.05 | 0.29 ± 0.05 |
| PE P-18:0/22:6 | 0.33 ± 0.04 | 0.29 ± 0.06 |
| PE P-18:0/22:5 | 0.35 ± 0.03 | 0.32 ± 0.05 |
| PE P-18:0/22:4 | 0.29 ± 0.04 | 0.27 ± 0.04 |
| PE P-18:0/22:3 | n.d. | n.d. |
| ∑ SFA PE P-16:0 | 0.61 ± 0.05 | 0.59 ± 0.10 |
| ∑ MUFA PE P-16:0 | 1.01 ± 0.25 | 1.09 ± 0.29 |
| ∑ PUFA PE P-16:0 | 5.11 ± 1.06 | 5.31 ± 1.37 |
| ∑ total PE P-16:0 | 6.73 ± 1.27 | 6.99 ± 1.69 |
| ∑ SFA PE P-18:0 | 0.57 ± 0.06 | 0.51 ± 0.06 |
| ∑ MUFA PE P-18:0 | 0.70 ± 0.10 | 0.67 ± 0.11 |
| ∑ PUFA PE P-18:0 | 2.82 ± 0.27 | 2.69 ± 0.38 |
| ∑ total PE P-18:0 | 4.09 ± 0.39 | 3.87 ± 0.52 |
| ∑ SFA PE P-18:1 | 0.59 ± 0.04 | 0.56 ± 0.08 |
| ∑ MUFA PE P-18:1 | 0.77 ± 0.14 | 0.79 ± 0.13 |
| ∑ PUFA PE P-18:1 | 3.12 ± 0.29 | 3.06 ± 0.50 |
| ∑ total PE P-18:1 | 4.48 ± 0.40 | 4.41 ± 0.64 |

n.d., not detected; SFA, saturated fatty acids; MUFA, monounsaturated fatty acids; PUFA, polyunsaturated fatty acids.

**Table S7 Phosphatidylinositol (PI) species in plasma (µM) of cows fed grape seed and grape marc meal extract (GSGME) and control cows at 1 week postpartum**

| PI species | Control | GSGME |
| --- | --- | --- |
| PI 32:2 | n.d. | n.d. |
| PI 32:1 | n.d. | n.d. |
| PI 32:0 | 0.05 ± 0.01 | 0.05 ± 0.01 |
| PI 33:1 | n.d. | n.d. |
| PI 33:0 | n.d. | n.d. |
| PI 34:3 | 0.02 ± 0.01 | 0.02 ± 0.01 |
| PI 34:2 | 0.15 ± 0.03 | 0.15 ± 0.04 |
| PI 34:1 | 0.43 ± 0.11 | 0.43 ± 0.14 |
| PI 35:2 | n.d. | n.d. |
| PI 35:1 | n.d. | n.d. |
| PI 35:0 | n.d. | n.d. |
| PI 36:4 | 0.16 ± 0.03 | 0.16 ± 0.05 |
| PI 36:3 | 0.34 ± 0.08 | 0.35 ± 0.11 |
| PI 36:2 | 1.36 ± 0.32 | 1.41 ± 0.54 |
| PI 36:1 | 1.59 ± 0.64 | 1.66 ± 0.63 |
| PI 36:0 | 0.02 ± 0.01 | 0.02 ± 0.02 |
| PI 38:6 | 0.03 ± 0.01 | 0.03 ± 0,01 |
| PI 38:5 | 0.46 ± 0.11 | 0.51 ± 0.18 |
| PI 38:4 | 2.85 ± 0.56 | 2.70 ± 0.70 |
| PI 38:3 | 1.55 ± 0.68 | 1.45 ± 0.56 |
| PI 38:2 | 0.19 ± 0.13 | 0.19 ± 0.80 |
| PI 40:7 | n.d. | n.d. |
| PI 40:6 | 0.08 ± 0.02 | 0.07 ± 0.02 |
| PI 40:5 | 0.28 ± 0.10 | 0.30 ± 0.10 |
| PI 40:4 | 0.09 ± 0.04 | 0.08 ± 0.03 |
| PI 40:3 | 0.01 ± 0.01 | 0.01 ± 0.01 |
| ∑ SFA | 0.07 ± 0.01 | 0.06 ± 0.02 |
| ∑ MUFA | 2.02 ± 0.74 | 2.08 ± 0.76 |
| ∑ PUFA | 7.57 ± 1.68 | 7.44 ± 2.18 |
| ∑ unsaturated | 9.59 ± 2.34 | 9.52 ± 2.88 |
| total | 9.65 ± 2.34 | 9.58 ± 2.89 |

n.d., not detected; SFA, saturated fatty acids; MUFA, monounsaturated fatty acids; PUFA, polyunsaturated fatty acids.

**Table S8 Lysophosphatidylcholine (LPC) species in plasma (µM) of cows fed grape seed and grape marc meal extract (GSGME) and control cows at 1 week postpartum**

| Species | Control | GSGME |
| --- | --- | --- |
| LPC 15:0 | 0.97 ± 0.49 | 1.20 ± 0.39 |
| LPC 16:1 | 0.70 ± 0.25 | 0.74 ± 0.21 |
| LPC 16:0 | 30.68 ± 7.49 | 31.20 ± 7.94 |
| LPC 18:3 | 0.93 ± 0.39 | 1.05 ± 0.38 |
| LPC 18:2 | 8.11 ± 2.75 | 8.86 ± 3.15 |
| LPC 18:1 | 8.65 ± 3.02 | 9.26 ± 2.59 |
| LPC 18:0 | 22.76 ± 6.55 | 23.52 ± 6.97 |
| LPC 20:5 | 0.24 ± 0.08 | 0.30 ± 0.11 |
| LPC 20:4 | 1.06 ± 0.30 | 1.12 ± 0.35 |
| LPC 20:3 | 0.78 ± 0.48 | 0.76 ± 0.31 |
| LPC 20:0 | 0.59 ± 0.18 | 0.75 ± 0.19 |
| LPC 22:6 | 0.19 ± 0.10 | 0.22 ± 0.07 |
| LPC 22:5 | 0.52 ± 0.19 | 0.68 ± 0.18 |
| LPC 22:4 | 0.27 ± 0.10 | 0.29 ± 0.09 |
| LPC 22:0 | 0.57 ± 0.17 | 0.69 ± 0.15 |
| ∑ SFA | 55.56 ± 13.94 | 57.36 ± 14.98 |
| ∑ MUFA | 9.35 ± 3.25 | 10.01 ± 2.78 |
| ∑ PUFA | 12.10 ± 4.05 | 13.28 ± 4.18 |
| ∑ unsaturated | 21.45 ± 7.23 | 23.29 ± 6.67 |
| total | 77.02 ± 20.22 | 80.65 ± 21.23 |

SFA, saturated fatty acids; MUFA, monounsaturated fatty acids; PUFA, polyunsaturated fatty acids.

**Table S9 Free cholesterol (FC) and cholesterol ester (CE) species in plasma (µM) of cows fed grape seed and grape marc meal extract (GSGME) and control cows at 1 week postpartum**

| Species | Control | GSGME |
| --- | --- | --- |
| FC | 411.19 ± 110.87 | 444.48 ±124.44 |
| CE 14:1 | n.d. | n.d. |
| CE 14:0 | 12.17 ± 5.94 | 11.88 ± 3.93 |
| CE 15:1 | n.d. | n.d. |
| CE 15:0 | 20.93 ± 12.34 | 21.93 ± 7.98 |
| CE 16:3 | n.d. | n.d. |
| CE 16:2 | n.d. | n.d. |
| CE 16:1 | 71.98 ± 27.35 | 70.31 ± 23.15 |
| CE 16:0 | 97.86 ± 22.31 | 93.66 ±21.87 |
| CE 17:1 | n.d. | n.d. |
| CE 18:4 | n.d. | n.d. |
| CE 18:3 | 454.57 ± 136.15 | 474.16 ± 140.12 |
| CE 18:2 | 2073.68 ± 411.04 | 2074.39 ± 541.84 |
| CE 18:1 | 132.76 ± 32.35 | 133.28 ± 35.27 |
| CE 18:0 | 3.16 ± 0.89 | 3.10 ± 0.90 |
| CE 19:2 | n.d. | n.d. |
| CE 19:1 | n.d. | n.d. |
| CE 19:0 | n.d. | n.d. |
| CE 20:5 | 63.16 ± 23.19 | 64.62 ± 29.2 |
| CE 20:4 | 105.76 ± 30.47 | 100.51 ± 28.25 |
| CE 20:3 | 22.69 ± 10.35 | 19.73 ± 7.03 |
| CE 20:2 | 1.15 ± 0.43 | 0.92 ± 0.21 |
| CE 20:1 | 0.54 ± 0.16 | 0.45 ± 0.13 |
| CE 20:0 | 0.90 ± 0.14 | 0.86 ± 0.08 |
| CE 22:6 | 3.98 ± 1.98 | 3.91 ± 1.58 |
| CE 22:5 | 3.58 ± 0.99 | 3.52 ± 1.03 |
| CE 22:4 | 1.24 ± 0.37 | 1.25 ± 0.30 |
| CE 22:3 | n.d. | n.d. |
| CE 22:2 | n.d. | n.d. |
| CE 22:1 | 0.25 ± 0.06 | 0.24 ± 0.07 |
| CE 24:7 | n.d. | n.d. |
| CE 24:6 | n.d. | n.d. |
| CE 24:5 | n.d. | n.d. |
| CE 24:4 | n.d. | n.d. |
| CE 24:3 | n.d. | n.d. |
| CE 24:2 | n.d. | n.d. |
| CE 24:1 | n.d. | n.d. |
| CE 24:0 | n.d. | n.d. |
| ∑ SFA | 135.02 ± 38.75 | 131.43 ± 32.40 |
| ∑ MUFA | 205.53 ± 56.96 | 204.28 ± 57.66 |
| ∑ PUFA | 2729.83 ± 574.72 | 2743.01 ± 714.92 |
| ∑ unsaturated | 2935.36 ± 627.24 | 2947.29 ± 765.14 |
| total | 3070.38 ± 663.03 | 3078.72 ± 794.26 |
| FC | 411.19 ± 110.87 | 444.48 ± 124.44 |

n.d., not detected; SFA, saturated fatty acids; MUFA, monounsaturated fatty acids; PUFA, polyunsaturated fatty acids.
